# Chemoenzymatic glycan-selective remodeling of a therapeutic lysosomal enzyme with high-affinity M6P-glycan ligands. Enzyme substrate specificity is the name of the game†

**DOI:** 10.1039/d1sc03188k

**Published:** 2021-08-19

**Authors:** Xiao Zhang, Huiying Liu, Naresh Meena, Chao Li, Guanghui Zong, Nina Raben, Rosa Puertollano, Lai-Xi Wang

**Affiliations:** Department of Chemistry and Biochemistry, University of Maryland 8051 Regents Drive College Park Maryland 20742 USA wang518@umd.edu; Cell and Developmental Biology Center, National Heart, Lung, and Blood Institute, NIH Bethesda Maryland 20892 USA

## Abstract

Functionalization of therapeutic lysosomal enzymes with mannose-6-phosphate (M6P) glycan ligands represents a major strategy for enhancing the cation-independent M6P receptor (CI-MPR)-mediated cellular uptake, thus improving the overall therapeutic efficacy of the enzymes. However, the minimal high-affinity M6P-containing *N*-glycan ligands remain to be identified and their efficient and site-selective conjugation to therapeutic lysosomal enzymes is a challenging task. We report here the chemical synthesis of truncated M6P-glycan oxazolines and their use for enzymatic glycan remodeling of recombinant human acid α-glucosidase (rhGAA), an enzyme used for treatment of Pompe disease which is a disorder caused by a deficiency of the glycogen-degrading lysosomal enzyme. Structure–activity relationship studies identified M6P tetrasaccharide oxazoline as the minimal substrate for enzymatic transglycosylation yielding high-affinity M6P glycan ligands for the CI-MPR. Taking advantage of the substrate specificity of endoglycosidases Endo-A and Endo-F3, we found that Endo-A and Endo-F3 could efficiently deglycosylate the respective high-mannose and complex type *N*-glycans in rhGAA and site-selectively transfer the synthetic M6P *N*-glycan to the deglycosylated rhGAA without product hydrolysis. This discovery enabled a highly efficient one-pot deglycosylation/transglycosylation strategy for site-selective M6P-glycan remodeling of rhGAA to obtain a more homogeneous product. The Endo-A and Endo-F3 remodeled rhGAAs maintained full enzyme activity and demonstrated 6- and 20-fold enhanced binding affinities for CI-MPR receptor, respectively. Using an *in vitro* cell model system for Pompe disease, we demonstrated that the M6P-glycan remodeled rhGAA greatly outperformed the commercial rhGAA (Lumizyme) and resulted in the reversal of cellular pathology. This study provides a general and efficient method for site-selective M6P-glycan remodeling of recombinant lysosomal enzymes to achieve enhanced M6P receptor binding and cellular uptake, which could lead to improved overall therapeutic efficacy of enzyme replacement therapy.

## Abstract 2

An efficient one-pot M6P glycan remodeling of a multiply glycosylated lysosomal enzyme (Lumizyme) affords a new glycoengineered protein that shows greatly improved receptor binding, cellular uptake, and degradation of lysosomal glycogen in an *in vitro* model of Pompe disease.

## Graphical abstract

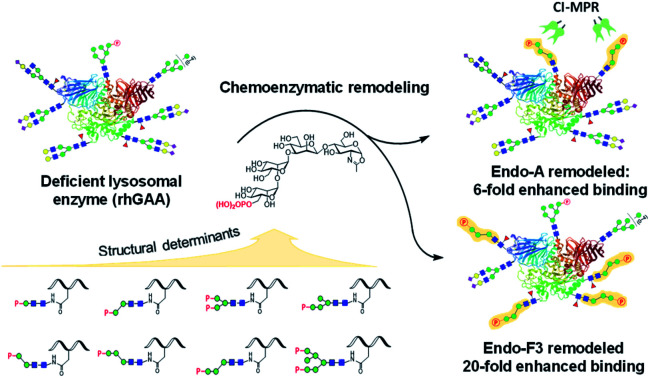

## Introduction

Lysosomal storage diseases (LSDs) are a group of inherited metabolic disorders caused by deficiency of respective hydrolases that are responsible for the degradation of substrates stored in lysosomes.^1^ The accumulation of undigested macromolecules leads to cell dysfunction and progressive clinical manifestations.^2^ A variety of therapeutic approaches have been attempted for LSDs,^3^ with intravenous enzyme replacement therapy (ERT) being the most prevalent.^4^ However, the effectiveness of ERT varies among different LSDs. For example, in the case of Pompe disease that is caused by a deficiency of the lysosomal glycogen-degrading enzyme, the acid α-glucosidase (GAA), a very high dose of the recombinant human acid α-glucosidase (rhGAA, Lumizyme) needs to be administered due to its relatively low cellular uptake and poor drug targeting.^5^ The cation-independent mannose-6-phosphate (M6P) receptor (CI-MPR), which continuously traffics between plasma membrane, late endosomes and trans-Golgi network (TGN), plays a critical role in cellular uptake and intracellular transport of enzymes to lysosomes by recognizing the M6P-containing *N*-glycans attached to the enzymes.^6–9^ Thus, enhancement of the CI-MPR-mediated endocytosis represents a major strategy to improve the overall efficiency of the ERT-based treatments.^10–12^ Toward this end, several approaches for increasing M6P modification of lysosomal enzymes have been attempted, including chemical conjugation of synthetic M6P-containing glycans,^13–22^ construction of non-mammalian based platforms to improve the M6P content,^23–26^ gene engineering of the glycosylation pathways,^27,28^ and a chemoenzymatic remodeling approach to introduce synthetic phosphorylated *N*-glycans.^29,30^ There are nice examples that the resulting modified enzymes show increased binding to CI-MPR and enhanced uptake by cells compared with the unmodified enzymes. Despite these promising studies, however, the essential structures of M6P-containing *N*-glycans that exhibit high affinity for the CI-MPR are to be identified and site-selective conjugation of the M6P glycan ligands to therapeutic lysosomal enzymes to achieve structurally well-defined products remains a challenging task.

**Fig. 1:**
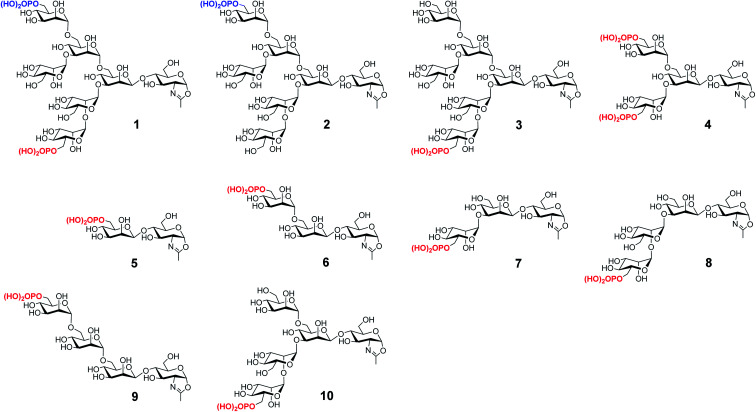
Structures of synthetic M6P-containing *N*-glycan oxazolines used for structure–activity relationship studies of enzymatic transglycosylation and M6P receptor binding.

We have previously reported the synthesis of several M6P-containing *N*-glycan oxazolines (compounds **1–4**, Fig. 1) and used them as donor substrates for endoglycosidase-catalyzed chemoenzymatic synthesis of M6P-containing glycoproteins, using ribonuclease B as a model glycoprotein.^30^ Binding studies have revealed that a single M6P moiety located at the low α-1,3-branch of the *N*-glycan context, derived from glycan oxazoline **3**, is sufficient for a high-affinity binding to CI-MPR, while the presence of a M6P moiety at the α-1,6-branch, as shown in glycan oxazolines **1** and **2**, appears dispensable.^30^ Despite these findings, the substrate specificity of the M6P-glycan oxazolines in enzymatic transglycosylation and the detailed structure–activity relationship of the M6P glycan ligands for receptor binding remain to be elucidated. In addition, it is not clear what constitutes the minimal M6P-glycan structures for high-affinity CI-MPR binding that will be feasible for a facile enzymatic glycan remodeling of therapeutic lysosomal enzymes. To address these questions, we report in this paper the chemical synthesis and evaluation of a series of truncated M6P-containing *N*-glycan oxazolines (compounds **5–10**, Fig. 1) as donor substrates for enzymatic transglycosylation and glycan remodeling of the commercial rhGAA, Lumizyme.^31^ Lumizyme is a therapeutic lysosomal enzyme that carries 7 different *N*-glycans and currently is used for the treatment of Pompe disease. The structure–activity relationship study revealed a Man6P-α1,2-Man disaccharide moiety in the synthetic *N*-glycans as a structural motif for high-affinity binding to the CI-MPR and identified a tetrasaccharide oxazoline as the minimal donor substrate for enzymatic transglycosylation to provide high-affinity M6P glycan ligands for the CI-MPR. This finding enabled a one-pot and site-selective glycan remodeling of the multiply glycosylated rhGAA to produce a more homogeneous glycoengineered enzyme that showed up to 20-fold enhanced binding affinities for the CI-MPR over the commercial Lumizyme. Moreover, by using a cell model system for Pompe disease, we demonstrated that the M6P-glycan remodeled rhGAA showed significantly enhanced cellular uptake and exhibited much more efficient degradation of glycogen in lysosomes than the commercial Lumizyme under the same conditions.

## Results and discussion

### Chemical synthesis of truncated high-mannose type phosphorylated *N*-glycan oxazolines

**Scheme 1:**
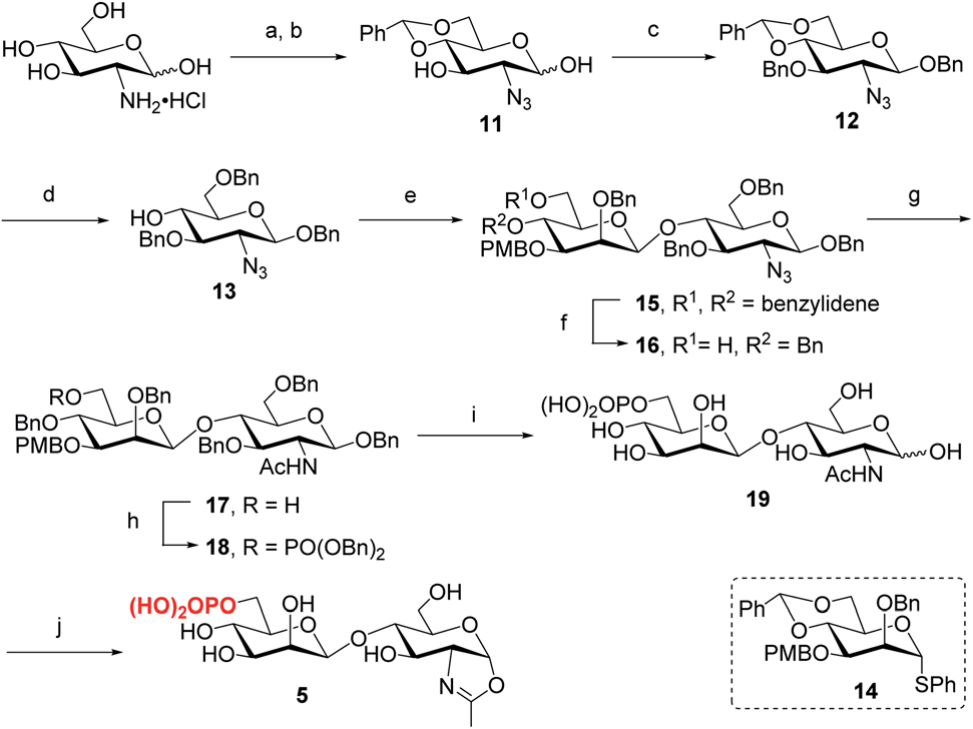
Synthesis of phosphorylated core disaccharide oxazoline **5**. Reagents and conditions: (a) TfN_3_, K_2_CO_3_, CuSO_4_, CH_2_Cl_2_/H_2_O, 0 °C–RT; (b) PhCH(OMe)_2_, CSA, MeCN, 73% for 2 steps; (c) BnBr, Ag_2_CO_3_, MeCN, 60 °C, then BnBr, NaH, DMF, 0 °C ∼RT, 75%; (d) Et_3_SiH, BF_3_·OEt_2_, DCM, 0 °C, 81%; (e) **14**, BSP, TTBP, Tf_2_O, CH_2_Cl_2_, 4 Å MS, −60 °C, 69%; (f) BH_3_·THF, Bu_2_BOTf, CH_2_Cl_2_, 0 °C, 91%; (g) AcSH, pyridine/CHCl_3_, RT, 84%; (h) (BnO)_2_PNiPr_2_, tetrazole, 4 Å MS, CH_2_Cl_2_, then mCPBA, −30 °C, 90%; (i) Pd/C, H_2_, THF/MeOH, then Pd(OH)_2_/C, H_2_, MeOH/H_2_O, 95%; (j) DMC, Et_3_N, H_2_O, 0 °C, 97%.

Following the previously reported synthesis of phosphorylated *N*-glycans,^18,29,30^ we first synthesized the selectively protected core disaccharide **15**. Starting from d-glucosamine hydrochloride, we prepared **11** in two steps following the reported procedure.^32^ Compound **11** was then treated with Ag_2_CO_3_ and BnBr to introduce a benzyl group to the anomeric position, followed by 3-*O*-benzylation with NaH and BnBr to yield **12** in 75% overall yield. The β-configuration was confirmed by the coupling constant of *J*_1,2_ = 7.8 Hz. Upon regioselective ring-opening of the benzylidene group, **13** was obtained in 81% yield, which was coupled with the known compound **14** (ref. 33) to give the core disaccharide **15** in 69% yield. Next, regioselective ring-opening reaction furnished **16** with a free OH at C6 position, and after the conversion of the 2-azido group into the 2-acetamido group with AcSH,^30^ the free OH was phosphorylated with dibenzyl *N*,*N*-diisopropylphosphoramidite, followed by oxidation with mCPBA^18^ to give **18** in 90% yield. Global deprotection of the Bn and PMB groups *via* a two-step catalytic hydrogenolysis^30^ gave the free disaccharide **19** in excellent yield. Finally, oxazoline formation was achieved in a single step by treatment with an excess amount of 2-chloro-1,3-dimethylimidazolinium chloride (DMC)^34^ in water in the presence of Et_3_N to afford the phosphorylated core disaccharide oxazoline **5** in 97% yield (Scheme 1).

**Scheme 2:**
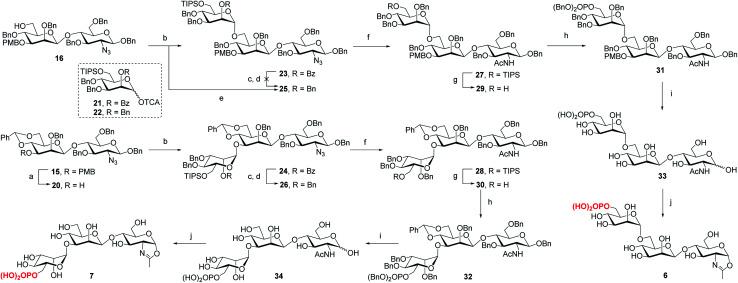
Synthesis of monophosphorylated trisaccharide oxazolines **6** and **7**. Reagents and conditions: (a) DDQ, CH_2_Cl_2_/H_2_O, 0 °C ∼RT, 90%; (b) **21**, TMSOTf, 4 Å MS, CH_2_Cl_2_, −30 °C, **23**, 94%, **24**, 92%; (c) CH_3_ONa, MeOH, 50 °C; (d) BnBr, NaH, DMF, 0 °C ∼RT, **26**, 90% for 2 steps; (e) **22**, TMSOTf, 4 Å MS, CH_2_Cl_2_, −30 °C, 72%; (f) AcSH, pyridine/CHCl_3_, 60 °C, **27**, 89%, **28**, 94%; (g) TBAF, THF, RT, **29**, 85%, **30**, 87%; (h) (BnO)_2_PNiPr_2_, tetrazole, 4 Å MS, CH_2_Cl_2_, then mCPBA, −30 °C, **31**, 75%, **32**, 90%; (i) Pd/C, H_2_, THF/MeOH, then Pd(OH)_2_/C, H_2_, MeOH/H_2_O, **33**, 96%, **34**, 80%; (j) DMC, Et_3_N, H_2_O, 0 °C, **6**, quant.,**7**, 95%.

To determine the role of the mannosyl branches, we synthesized trisaccharide oxazolines **6** and **7** with a phosphate group at the α-1,6 or α-1,3-branch, respectively (Scheme 2). Deprotection of the 3-*O*-PMB in **15** gave **20** as an acceptor, then **16** and **20** were glycosylated with glycosyl donor **21** (ref. 35) to give trisaccharides **23** and **24**, respectively. A two-step manipulation was conducted to convert the benzoyl group to permanent benzyl group, giving **26** in 90% yield. However, the benzylation step failed to afford **25** even under harsh conditions probably due to high steric hindrance, thus the 2-*O*-Bn imidate **22** (ref. 36) was used as the donor, which furnished **25** in 72% yield along with 15% of the β isomer. Next, upon the reduction of azido group to acetamido group followed by the selective deprotection of TIPS group with TBAF, **29** and **30** were obtained in 85% and 87% yield, respectively, which were ready for phosphorylation. Finally, the phosphate group was introduced at the C6 position to give the fully protected derivatives **31** and **32** in 75% and 90% yield, respectively. Global deprotection *via* hydrogenolysis removed all the Bn, PMB and benzylidene groups simultaneously, giving free trisaccharides **33** and **34**, which were converted into glycan oxazolines **6** and **7** respectively by reaction with DMC in a single step.

**Scheme 3:**
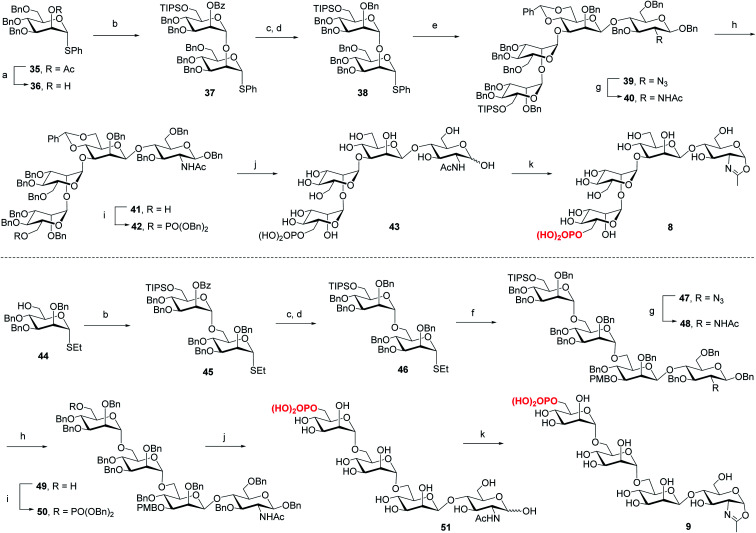
Synthesis of monophosphorylated tetrasaccharide oxazolines **8** and **9**. Reagents and conditions: (a) CH_3_ONa, MeOH/CH_2_Cl_2_, RT, 90%; (b) **21**, TMSOTf, 4 Å MS, CH_2_Cl_2_, −30 °C, **37**, 96%, **45**, 94%; (c) CH_3_ONa, MeOH, 50 °C; (d) BnBr, NaH, DMF, 0 °C∼RT, **38**, 83% for 2 steps, **46**, 89% for 2 steps; (e) **20**, NIS, TfOH, 4 Å MS, CH_2_Cl_2_, −30 °C, 74%; (f) **16**, NIS, AgOTf, 4 Å MS, CH_2_Cl_2_/Et_2_O, −40 °C, 72%; (g) AcSH, pyridine/CHCl_3_, 60 °C, **40**, 86%, **48**, 85%; (h) TBAF, THF, RT, **41**, 83%, **49**, 70%; (i) (BnO)_2_PNiPr_2_, tetrazole, 4 Å MS, CH_2_Cl_2_, then mCPBA, −30 °C, **42**, 79%, **50**, 88%; (j) Pd/C, H_2_, THF/MeOH, then Pd(OH)_2_/C, H_2_, MeOH/H_2_O, **43**, 95%, **51**, 91%; (k) DMC, Et_3_N, H_2_O, 0 °C, **8**, 87%, **9**, 90%.

For the synthesis of tetrasaccharides with only α-1,3 or α-1,6-branch, the disaccharide **37** was first synthesized by coupling of donor **21** with the known acceptor **36**.^37^ After the conversion of the benzoyl group to permanent benzyl group, the resulting disaccharide **38** was then used as the glycosyl donor for coupling with **20** under the promotion of NIS and TfOH to provide **39** in 74% yield, which was confirmed as the sole α isomer. After reduction of the azido group to acetamido group, followed by the selective deprotection of TIPS group, the C6 free OH was phosphorylated to give **42**. Finally, the global deprotection and oxazoline formation provided the oxazoline **8** in good yield (Scheme 3). In parallel, the 1,6-linked disaccharide **45** was prepared by the coupling of donor **21** with **44**,^38^ which furnished the glycosyl donor **46** upon the conversion of the benzoyl group to benzyl group. The [2 + 2] coupling of **46** and **16** afforded **47** in 72% yield along with 17% of β isomer. Conversion of the azido group to acetamido group, followed by deprotection of TIPS and standard phosphorylation gave **50** in good yield. Finally, global deprotection of **50** followed by DMC treatment gave the oxazoline **9** (Scheme 3).

**Scheme 4:**
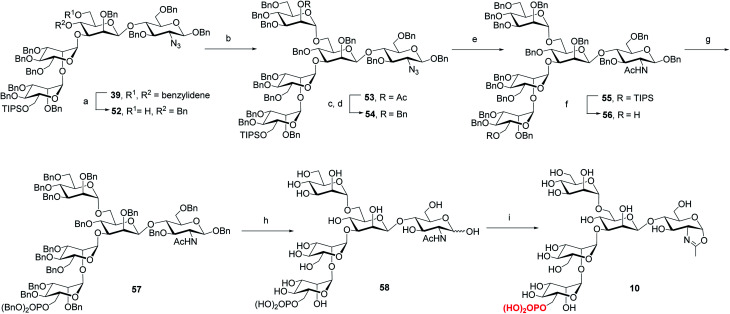
Synthesis of monophosphorylated pentasaccharide oxazoline **10**. Reagents and conditions: (a) Et_3_SiH, PhBCl_2_, 4 Å MS, CH_2_Cl_2_, −78 °C, 79%; (b) **35**, NIS, TfOH, 4 Å MS, CH_2_Cl_2_, −30 °C, 80%; (c) CH_3_ONa, CH_2_Cl_2_/MeOH, RT; (d) BnBr, NaH, DMF, 0 °C ∼RT, 85% for 2 steps; (e) AcSH, pyridine/CHCl_3_, 60 °C, 84%; (f) TBAF, THF, RT, 90%; (g) (BnO)_2_PNiPr_2_, tetrazole, 4 Å MS, CH_2_Cl_2_, then mCPBA, −30 °C, 90%; (h) Pd/C, H_2_, THF/MeOH then Pd(OH)_2_/C, H_2_, MeOH/H_2_O, 77%; (i) DMC, Et_3_N, H_2_O, 0 °C, 93%.

Previous studies have shown that not only the glycan structure determinants but also its optimal orientation is important for high-affinity binding to CI-MPR.^17,39,40^ Considering the critical role of the Man3 core for retaining the glycan conformation, we designed and synthesized the pentasaccharide oxazoline **10**. Starting with tetrasaccharide **39**, regioselective ring-opening reaction afforded **52** with a C6 free OH at the core mannosyl residue, then another mannosyl residue was installed at this position using glycosyl donor **35** (ref. 37) to give **53** in 80% yield. After the acetyl group was converted into benzyl group, oxazoline **10** was readily obtained as described for the synthesis of **5–9** by sequential reduction of azido group, deprotection of TIPS, phosphorylation, global deprotection and final oxazoline formation (Scheme 4).

### Evaluation of the synthetic M6P-glycan oxazolines as donor substrates for enzymatic transglycosylation

**Scheme 5:**
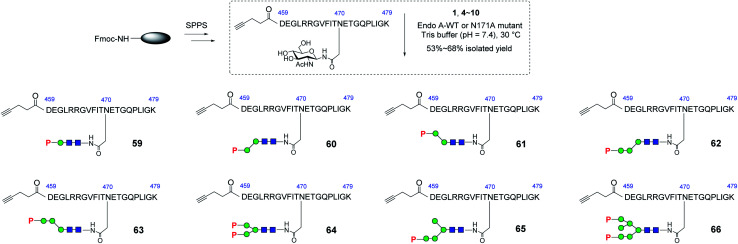
Synthesis of GlcNAc-peptide derived from rhGAA. Reagents and conditions: GlcNAc-peptide (2.0–3.0 mg), oxazoline (4–6 eq.) with Endo-A WT (for **59–65**) or Endo-A N171A (for **66**) was incubated in Tris buffer (100 mM, pH = 7.4) at 30 °C. **59**, 60%, **60**, 66%, **61**, 60%, **62**, 66%, **63**, 57%, **64**, 68%, **65**, 54%, **66**, 53%.

With these synthetic phosphorylated oxazolines in hand, we sought to test their activity as donor substrates for enzymatic transglycosylation. For that purpose, we selected a 21-mer glycopeptide (aa 459–479) derived from rhGAA as a model sequence, in which the N470 residue was selected to install a M6P-glycan,^16^ and the precursor GlcNAc-peptide was synthesized *via* automated solid-phase peptide synthesis (SPPS). Previous studies have shown that wild-type Endo-A, an endoglycosidase from *Arthrobacter protophormiae*,^41^ is efficient for the transglycosylation of truncated phosphorylated Man_3_GlcNAc oxazoline to GlcNAc-peptides,^29,30^ but not suitable for transferring large natural M6P high-mannose *N*-glycan oxazolines, due to its rapid hydrolysis of both the oxazoline donors and the resulting transglycosylation products.^30^ In this study, we found that the truncated structures (**4–10**) acted as good substrates of wild-type Endo-A, and the resulting products, once formed, were barely hydrolyzed by the enzyme, affording the desired glycopeptides (**59–65**) in good isolated yields. The newly formed phosphorylated products were eluted later than the GlcNAc-peptide under the reverse-phase HPLC condition, and the identities of the products were confirmed with ESI-MS (Fig. S1†). Notably, the transglycosylation yields could be driven to 80–90% if additional sugar oxazolines were added to the reaction. Finally, for the bis-phosphorylated Man_6_GlcNAc oxazoline **1**, Endo-A-N171A was used and the glycopeptide **66** was obtained in 53% yield after purification by HPLC (Scheme 5).

### Evaluation of the affinity of the synthetic M6P-glycopeptides for the M6P receptor (CI-MPR)

To determine the minimal M6P glycan structures that can still provide high-affinity for CI-MPR, we performed SPR binding studies with immobilized CI-MPR (Fig. S3† and Table 1). The results revealed that the glycopeptide **62** derived from tetrasaccharide oxazoline **8** with a Man6P-α1,2-Man disaccharide moiety at α1,3-arm of the core β-mannose residue showed strong binding affinity (70 nM) with CI-MPR, which was comparable to that of glycopeptide **66** with a bis-phosphorylated Man_6_GlcNAc_2_ ligand (54 nM). The glycopeptide **65** obtained from the pentasaccharide oxazoline **10** showed similar affinity (82 nM), suggesting that the presence of an additional α1,6-linked mannose moiety did not affect the affinity. Interestingly, when the M6P moiety was α1,3- or α1,6-linked to the next mannosyl residue in the *N*-glycan, the resulting glycopeptides (**60**, **61** and **63**) showed dramatically decreased affinity for CI-MPR. Taken together, the experimental data suggest that tetrasaccharide oxazoline **8** with a Man6P-α1,2-Man disaccharide moiety at α1,3-arm of the core β-mannose residue appears to be the minimal truncated *N*-glycan oxazoline derived from the natural structure to provide a high-affinity ligand for CI-MPR. The results further suggest that a Man6P-α1,2-Man disaccharide moiety constitutes an essential structural motif that retains strong binding affinity for the receptor.^39,40^

**Table 1:** SPR analysis of the binding of M6P-containing glycopeptides with the immobilized CI-MPR receptor^a^

Compounds	**59**	**60**	**61**	**62**	**63**	**64**	**65**	**66**
*K*_D_ (μM)	>50^b^	1.8^c^	>50^b^	0.070 ± 0.002	>50^b^	0.84^c^	0.082 ± 0.014	0.054 ± 0.006

a Serial 2-fold dilution of concentrations (7.8–4000 nM) were performed for the SPR analysis.

b No obvious binding was detected up to 50 μM.

c Estimated by steady state fitting because the kinetic fitting did not give reliable data. The standard deviations were obtained from three independent experiments.

#### Glycan remodeling of RNase B and the affinity of the glycan remodeled protein for CI-MPR

**Scheme 6:**
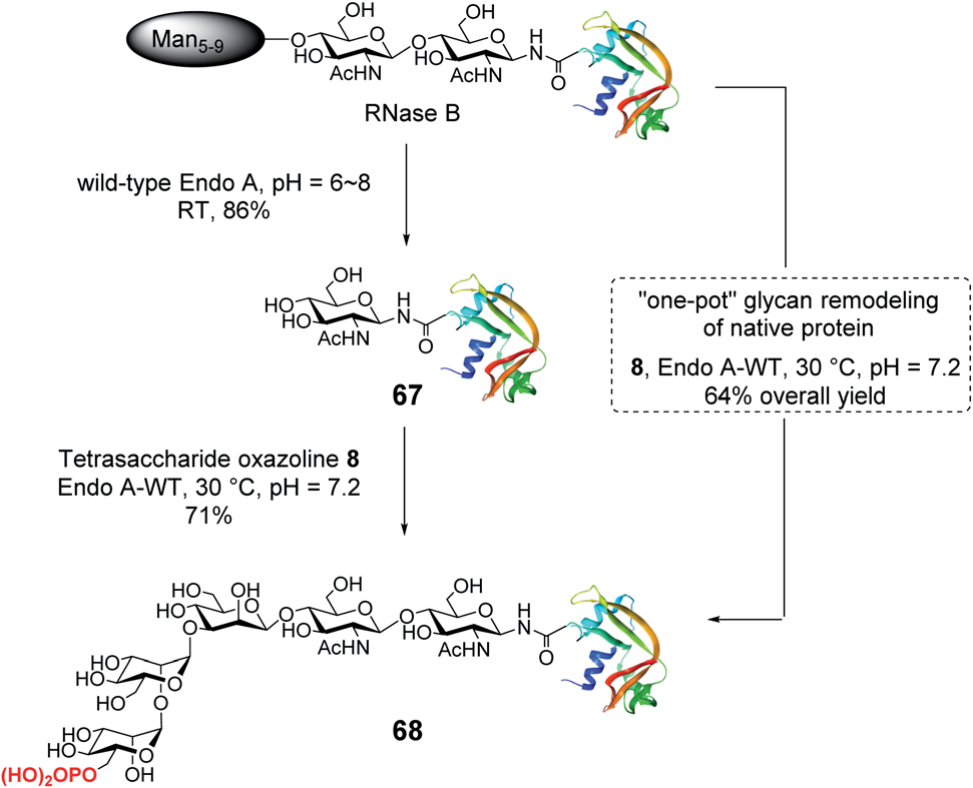
Stepwise and one-pot strategy to modify RNase B with the minimal tetrasaccharide oxazoline.

With the identification of the minimal high-affinity ligand (**8**), we next investigated the suitability of this M6P glycan oxazoline for glycan remodeling of glycoproteins, first using bovine RNase B as a model substrate, which carries a high-mannose type *N*-glycan at a single *N*-glycosylation site (Asn-34) (Scheme 6). RNase B was deglycosylated with wild-type Endo-A to give the homogeneous GlcNAc-RNase B (**67**). Then the enzymatic reaction between oxazoline **8** and GlcNAc-RNase B under the catalysis of the same enzyme smoothly afforded the phosphorylated glycoprotein **68** in 71% yield, the identity of which was confirmed by ESI-MS (calculated, *M* = 14 655; found, *M* = 14 656, deconvolution data). Although wild-type Endo-A was quite active for hydrolyzing natural Man5–Man9 *N*-glycan structures, we found that the M6P glycan oxazoline (**8**) was only very slowly hydrolyzed by this enzyme, and the resulting glycoprotein was resistant to hydrolysis under the reaction conditions, probably due to the truncation and phosphorylation of the *N*-glycan. The huge difference in the hydrolytic activities of Endo-A toward the starting glycoprotein and the resulting product, together with its excellent transglycosylation activity toward the truncated M6P-glycan oxazoline, prompted us to devise a one-pot strategy for enzymatic glycan remodeling. Thus, RNase B was incubated with wild-type Endo-A at 30 °C for 30 min before the addition of oxazoline **8** and the reaction mixture was incubated until the transglycosylation was complete. We found that the deglycosylation of RNase B proceeded quickly, followed by the formation of the desired phosphorylated glycoprotein **68** as a result of transglycosylation. The one-pot protocol simplified the procedure by omitting the purification of the intermediate after deglycosylation, thus improving the overall efficiency of the glycan remodeling approach. SPR binding experiments revealed that the M6P-containing RNase B (**68**) showed strong affinity for CI-MPR (*K*_D_ = 15.8 nM, Fig. S4†), which, again, was comparable to that of the glycoprotein with a large bis-M6P-Man_6_GlcNAc_2_ glycan.^30^ The results suggest that a simple M6P tetrasaccharide oxazoline is sufficient to enable the glycan remodeling of glycoprotein with high-affinity binding to CI-MPR in a one-pot manner.

### One-pot and glycan-selective remodeling of the recombinant human acid α-glucosidase (rhGAA, Lumizyme) using wild-type Endo-A and Endo-F3

**Scheme 7:**
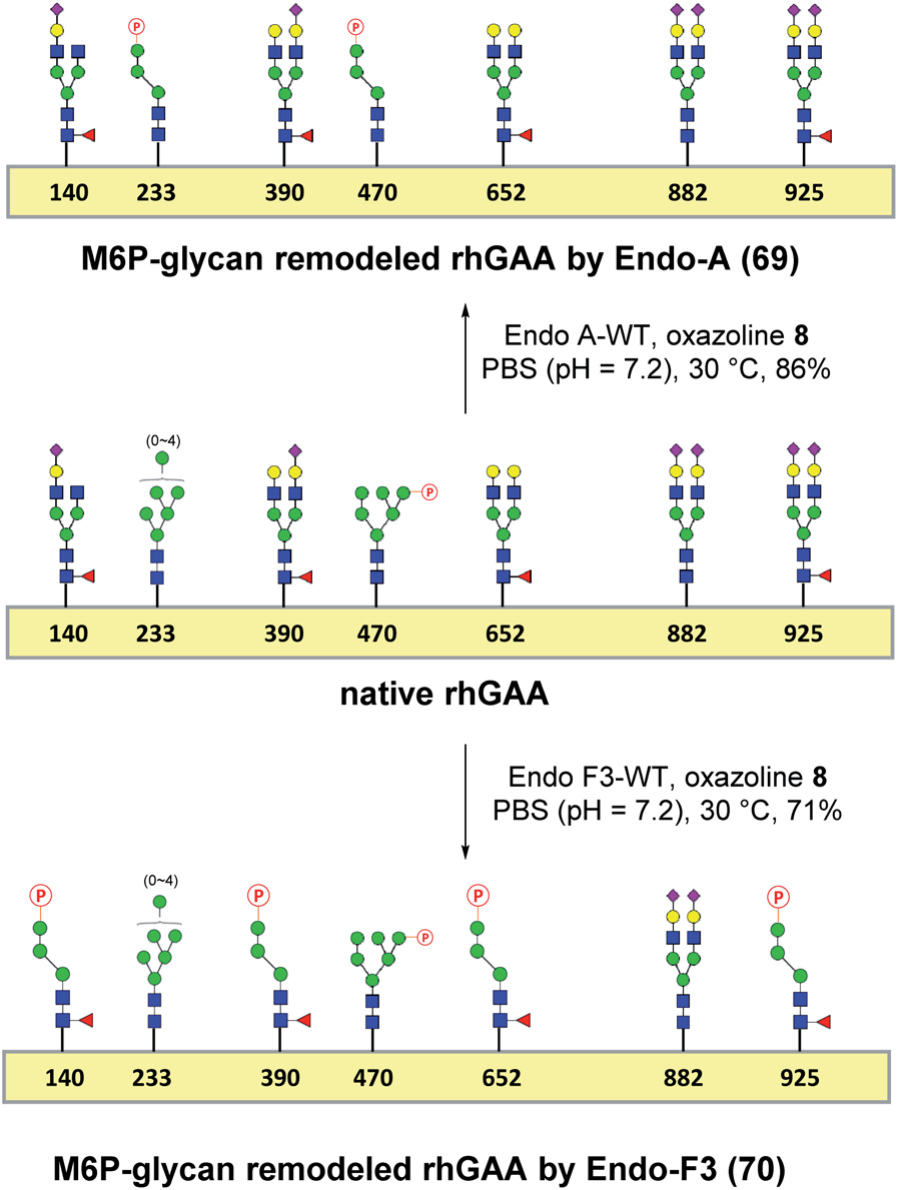
“One-pot” glycan remodeling of commercial rhGAA.

**Fig. 2:**
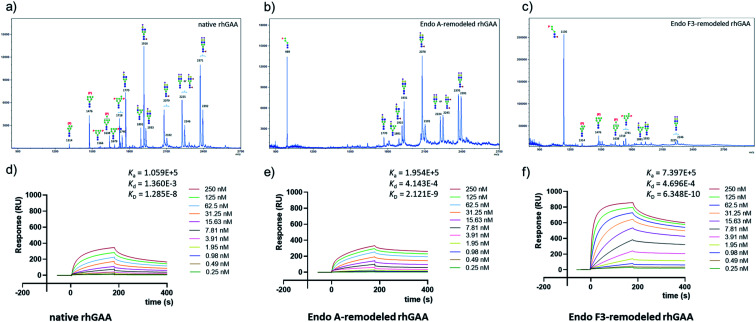
Glycan analysis (negative mode) and representative sensorgrams of three independent SPR binding experiments of native rhGAA and M6P glycan-remodeled rhGAA (**69** and **70**). (a) MALDI-TOF MS analysis of the *N*-glycans released from commercial rhGAA (Lumizyme). (b) MALDI-TOF MS analysis of the *N*-glycans released from the Endo-A remodeled rhGAA (**69**). (c) MALDI-TOF MS analysis of the *N*-glycans released from the Endo-F3 remodeled rhGAA (**70**). (d) SPR sensorgram of the binding between CI-MPR and the commercial rhGAA. (e) SPR sensorgram of the binding between CI-MPR and the Endo-A remodeled rhGAA (**69**). (f) SPR sensorgram of the binding between CI-MPR and the Endo-F3 remodeled rhGAA (**70**). The average *K*_D_ values for native rhGAA, **69** and **70** were 14.0 ± 3.7 nM, 2.3 ± 0.2 nM and 0.63 ± 0.07 nM, respectively.

Encouraged by the promising model study with RNase B, we next evaluated the feasibility of the one-pot enzymatic M6P-glycan remodeling on the recombinant human acid α-glucosidase (rhGAA; Lumizyme, Sanofi Genzyme), a therapeutic, multiply glycosylated lysosomal enzyme used for the treatment of Pompe disease.^22^ rhGAA produced in CHO cells has seven *N*-glycosylation sites,^31^ of which two sites (N233 and N470) reportedly contain high-mannose type glycans, while the rest are mainly occupied by core-fucosylated complex type *N*-glycans.^19^ Lumizyme, which is currently used for the enzyme replacement therapy of Pompe disease, contains relatively low amounts of high-mannose M6P-glycans, thus limiting its targeting and the overall therapeutic efficacy. The low M6P content partially explains why up to 20-fold higher dose is usually required for the treatment of Pompe disease than those of the lysosomal enzymes used for the treatment of other LSDs.^22^ Considering the substrate specificity of different endoglycosidases, we tested if we could selectively modify the high-mannose type *N*-glycans but leave the complex type *N*-glycans unchanged, or keep the high-mannose type *N*-glycans while acting on the complex type *N*-glycans selectively. Based on the success in modification of RNase B, wild-type Endo-A offers an excellent choice to selectively trim the high-mannose type glycans and install simultaneously the synthetic M6P-glycan *via* a one-pot strategy in view of its substrate specificity.^41^ Thus, the commercial rhGAA was treated with Endo-A, followed by addition of several portions of oxazoline **8** at 30 °C in one pot. The resulting reaction mixture was treated with Glutathione Agarose to remove the GST-tagged Endo-A, and the cleaved glycans and salts were removed by ultrafiltration (Scheme 7). Glycan analysis revealed the removal of high-mannose type *N*-glycans and introduction of the M6P-glycan after transglycosylation without affecting the complex type *N*-glycans (Fig. 2a and b and S5†). This result confirmed the efficiency of the site-selective glycan remodeling of a multiply glycosylated therapeutic protein.

Since the major glycoforms of rhGAA are core-fucosylated complex type *N*-glycans, we also sought to selectively remodel the core-fucosylated complex type *N*-glycans with M6P-glycan and to keep the original phosphorylated high-mannose type *N*-glycans unchanged. For this purpose, we turned our attention to Endo-F3, an endoglycosidase from *Elizabethkingia meningoseptica* that efficiently hydrolyzes core-fucosylated complex type *N*-glycans but is unable to cleave high-mannose type *N*-glycans.^42–45^ As an initial experiment, we found that, indeed, the Endo-F3 could efficiently transfer the minimal M6P-tetrasaccharide oxazoline (**8**) to a model core-fucosylated GlcNAc-peptide (Fucα1,6GlcNAc-CD52) and, interestingly, the resulting M6P-glycopeptide was resistant to hydrolysis by this enzyme (Fig. S6†). This preliminary study prompted us to test a one-pot strategy for selective glycan remodeling of rhGAA with Endo-F3. Thus, the commercial rhGAA was treated with Endo-F3 to selectively remove the core-fucosylated *N*-glycans, presumably at the N140, N390, N652, and N925 sites based on previous site-specific glycosylation profiling analysis,^16,19^ followed by three portions of tetrasaccharide oxazoline **8** every 2 hours at 30 °C (Scheme 7). The resulting reaction mixture was purified by HisTrap column to remove the His-tagged Endo-F3, and the cleaved glycans and extra salts were removed by ultrafiltration. Notably, we found that the Endo-F3-deglycosylated rhGAA was unstable and prone to precipitation. As a result, the one-pot simultaneous deglycosylation/transglycosylation strategy appeared to be much more efficient than a stepwise method that required an isolation of the deglycosylated intermediate. Glycan analysis confirmed the removal of complex type *N*-glycans and introduction of the M6P-glycan without affecting the high-mannose type *N*-glycans (Fig. 2c and S5†). The Endo-F3-based glycan remodeling method is complementary to the Endo-A-based glycan remodeling in terms of the *N*-glycan selectivity. Given the fact that most of the therapeutic enzymes used in ERTs are multiply glycosylated and usually carry both high-mannose type and complex type *N*-glycans, the present selective glycan remodeling method provides a general platform for a single-step, site- and glycan-selective M6P-glycan remodeling of most lysosomal enzymes with a more homogeneous product, which appears to be superior to the existing chemical conjugations with natural and synthetic M6P ligands.^16,24^

## CI-MPR binding of the M6P glycan-remodeled rhGAA

SPR experiments indicated that the native rhGAA had a notable affinity for CI-MPR (*K*_D_ = 14.0 nM) (Fig. 2d and S7†). Upon treatment with wild-type Endo-A to remove the high-mannose *N*-glycans including the M6P glycans, the deglycosylated rhGAA lost its binding affinity (Fig. S7†). However, after Endo-A catalyzed transglycosylation with the M6P-tetrasaccharide oxazoline (**8**), the remodeled rhGAA (**69**) exhibited a 6-fold enhanced affinity for the receptor (*K*_D_ = 2.3 nM, Fig. 2e). On the other hand, the Endo-F3-remodeled rhGAA (**70**) showed a 20-fold enhanced affinity (*K*_D_ = 0.63 nM, Fig. 2f). These results suggested that introduction of additional M6P-glycans into rhGAA resulted in further enhancement of affinity for the receptor. Previous studies have also shown that a novel rhGAA (designated as ATB200) carrying a higher M6P content that binds the CI-MPR with high affinity (apparent *K*_D_, ∼2–4 nM), exhibited enhanced cellular uptake and led to much improved glycogen reduction and reversal of muscle pathology in preclinical models.^46^ As a result, it was expected that the Endo-A remodeled rhGAA might exhibit comparable *in vivo* potency as ATB200, while the Endo-F3 remodeled enzyme might demonstrate much better therapeutic efficacy than ATB200 and commercial rhGAA, due to its much higher affinity for CI-MPR.

## Enzyme activity of the M6P glycan-remodeled rhGAA

**Fig. 3:**
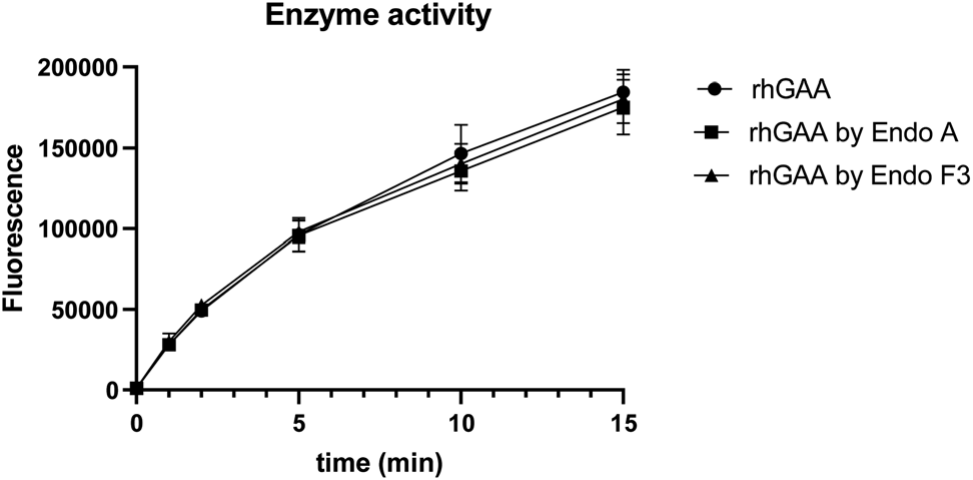
Comparison of the enzyme activity of commercial rhGAA and the M6P glycan-remodeled rhGAA (**69** and **70**).

To confirm if the remodeled enzymes still maintained their catalytic activity after M6P-glycan remodeling, we assessed the α-glucosidase activity of the commercial rhGAA, the Endo-A remodeled rhGAA (**69**), and the Endo-F3 remodeled rhGAA (**70**) using 4-methylumbelliferyl-α-d-glucopyranoside (4-MUG) as the substrate.^24^ The results indicated that the M6P glycan remodeled rhGAA maintained full enzyme activity as the parent rhGAA (Fig. 3). These data confirmed that the glycoengineering process was mild enough without denaturing the enzyme, and the resulting M6P-glycan remodeled enzyme was stable.

### Evaluation of the biological effect of the Endo-A and Endo-F3 remodeled rhGAA in an *in vitro* model of Pompe disease

Skeletal muscle is a major tissue affected in all forms of Pompe disease, and its response to the currently available therapy with the recombinant human GAA (rhGAA; Lumizyme) is not satisfactory.^47^ To evaluate the effect of the Endo-A (**69**) and Endo-F3 (**70**) remodeled rhGAA in the disease-relevant muscle cells, we used GAA-deficient multinucleated myotubes (KO) as an *in vitro* cell model system for Pompe disease.^48,49^ These myotubes are formed from conditionally immortalized myoblasts derived from the GAA knockout mice; unlike myoblasts, the differentiated myotubes replicate the primary defect of the disease, namely, the enlargement of glycogen-laden lysosomes. This physiologically relevant *in vitro* cell model system has been shown to closely replicate the pathogenic mechanisms of muscle tissue abnormalities in Pompe disease.^48,49^

**Fig. 4:**
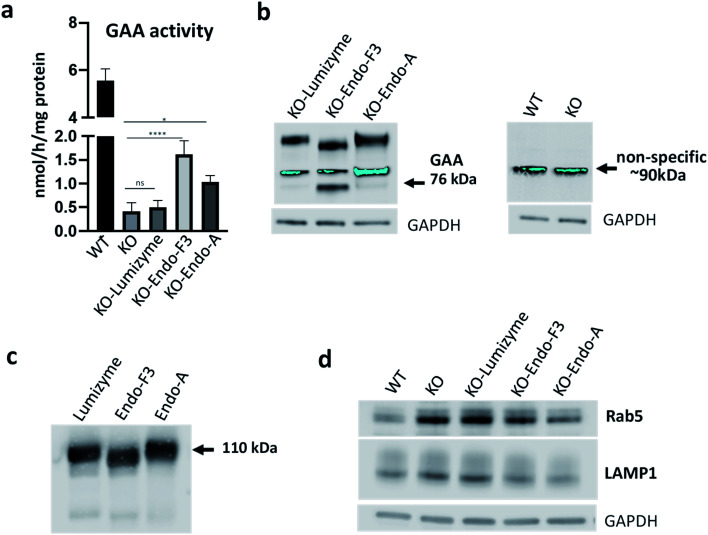
Effect of the therapeutic enzymes in GAA KO (KO) myotubes. (a) GAA activity was measured in cell lysates from untreated- (KO) and treated KO myotubes following incubation with Lumizyme (KO-Lumizyme), Endo-F3 remodeled rhGAA (KO-Endo-F3), and Endo-A remodeled rhGAA (KO-Endo-A). GAA activity in cell lysates of wild type (WT) myotubes was measured for comparison. The KO cells were treated for 24 hours with 5 μM of each of the recombinant enzyme; *n* = 5 for each condition. Statistical significance was determined by one-way ANOVA; graph represents mean ± SD. *p <0.05; ****p <0.0001; ns – statistically insignificant. (b) Western blot analysis (with anti-human GAA antibodies) of muscle cell lysates treated with the recombinant enzymes; GAPDH was used as loading control. (c) Western blot analysis of the recombinant enzymes with anti-human GAA antibodies. (d) Western blot analysis of WT, untreated- and treated muscle cell lysates with lysosomal (anti-LAMP1) and endosomal (anti-Rab5) antibodies. A decrease in the levels of both markers is observed in Endo-F3- and Endo-A treated cells.

Muscle cells were exposed to the commercial rhGAA (Lumizyme), the Endo-A remodeled rhGAA (**69**), and Endo-F3 remodeled rhGAA (**70**) (5 μM for 24 hours), which reach lysosomes *via* mannose 6-phosphate-mediated endocytosis. KO myotubes treated with Lumizyme and the glycoengineered proteins (**69** and **70**) were lysed, and the GAA activity was quantified using 4-Methylumbelliferyl-α-d-glucopyranoside, a fluorogenic substrate that is routinely used for the GAA assay in the diagnosis of Pompe disease. We found that GAA activity in the cell lysates increased significantly following incubation with both M6P-glycan remodeled proteins (**69** and **70**), whereas only a slight increase (statistically insignificant) was observed in Lumizyme-treated cells compared to the background level in the untreated cells (Fig. 4a). These results are consistent with the enhanced affinity of the M6P-glycan remodeled enzymes (**69** and **70**) to the CI-MPR receptor (Fig. 2). The effect of the Endo-F3 remodeled rhGAA (**70**) was more pronounced compared to that of Endo-A remodeled protein (**69**), suggesting its better cellular uptake (Fig. 4a). Western blot with anti-human GAA antibodies revealed the presence of the processed, mature lysosomal form of rhGAA (76 kDa) in cells treated with the Endo-F3 remodeled protein (**70**) (Fig. 4b, left panel); a weaker band was also detected in cell lysates following incubation with the Endo-A remodeled enzyme (**69**). In contrast, the 76 kDa form was barely detectable in cells treated with Lumizyme. The available anti-human GAA antibodies detected a strong non-specific band in mouse muscle cells as indicated by its presence in both WT and GAA-deficient cell (Fig. 4b, right panel). Previous studies have shown that the mature 76 kDa form of GAA has a higher affinity and activity towards glycogen.^31,50^

The immunoblot also showed that the molecular weight of the internalized GAA precursor appeared to be lower than the expected 110 kDa in the samples treated with the Endo-F3 remodeled rhGAA (**70**) compared to those treated with Lumizyme or the Endo-A remodeled rhGAA (**69**). The lower molecular weight of the Endo-F3 remodeled enzyme (**70**) was confirmed by Western analysis of the three recombinant proteins stained with anti-human GAA antibodies (Fig. 4c). These data were also consistent with the MALDI-TOF MS analysis of the glycan-remodeled rhGAA (**69** and **70**), which showed that the Endo-A remodeled rhGAA (**69**) had an average molecular mass of *ca.* 110 kDa, while the Endo-F3 remodeled rhGAA (**70**) had a molecular mass of *ca*.108 kDa (Fig. S8†). This observation was expected, as Endo-F3 based remodeling replaced most of the complex type *N*-glycans in the rhGAA with a much smaller (pentasaccharide) M6P *N*-glycan while the Endo-A based remodeling only changed the two high-mannose *N*-glycans to the M6P *N*-glycans. The efficient lysosomal trafficking of the remodeled proteins was associated with a significant reduction in the levels of both endosomal (Rab5) and lysosomal (LAMP1) markers, suggesting a reversal of lysosomal swelling; in contrast, the levels of these markers in Lumizyme-treated cells remained the same as in the untreated GAA-deficient cells (Fig. 4d).

**Fig. 5:**
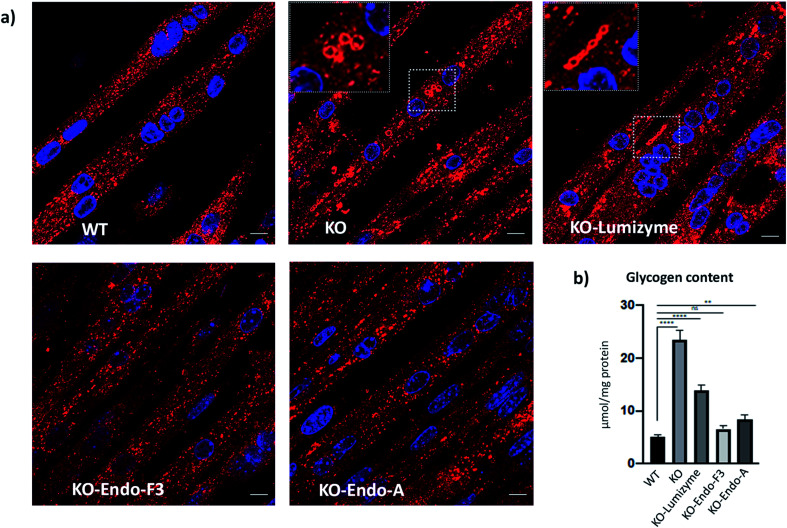
Effect of the therapeutic enzymes on lysosomal swelling and glycogen content in GAA KO (KO) myotubes. (a) Confocal images of WT, untreated- (KO) and treated KO myotubes following incubation with Lumizyme (KO-Lumizyme), Endo-F3 remodeled rhGAA (KO-Endo-F3), and Endo-A remodeled rhGAA (KO-Endo-A). Enlarged LAMP1-positive lysosomes (red) can be detected in untreated- (KO) and in Lumizyme-treated cells but not in WT or KO myotubes treated with the remodeled enzymes. Nuclei are stained with DAPI (blue). Bar: 10 μm. (b) Graph illustrates glycogen levels measured in cell lysates from WT, untreated- and treated KO myotubes. Endo F3 remodeled enzyme restored normal levels of glycogen in KO myotubes; *n* = 5 for each condition. Statistical significance was determined by one-way ANOVA; graph represents mean ± SD. **p <0.01; ****p <0.0001; ns – statistically insignificant.

The effect of the Endo-A and Endo-F3 remodeled rhGAA was confirmed by immunostaining of myotubes with Lamp1. Enlarged lysosomes were seen in cells treated with Lumizyme but not in those treated with the remodeled enzymes (**69** and **70**) (Fig. 5a; see also additional images in Fig. S9†). Finally, the degree of glycogen reduction achieved with Endo-A and Endo-F3 remodeled rhGAA (**69** and **70**) was significantly greater compared to that with the commercial rhGAA. Importantly, glycogen content in the diseased cells returned to the WT level following treatment with Endo-F3 remodeled rhGAA (**70**) (Fig. 5b). Taken together, these *in vitro* data indicated that upon efficient internalization, the M6P-glycan remodeled proteins, in particular the Endo-F3 remodeled rhGAA, could efficiently traffic to their correct cellular destination, the lysosome, and do their job there, *i.e.*, break down the accumulated glycogen. The therapeutic potential of the M6P glycan-remodeled rhGAA in lysosomal glycogen reduction and reversal of muscle pathology will be further evaluated in Pompe disease animal models in future studies.

## Conclusion

Here, we describe the chemical synthesis of an array of mannose-6-phosphate (M6P) containing *N*-glycan oxazolines and their use as donor substrates for chemoenzymatic synthesis of M6P-containing glycopeptides and for the glycan remodeling of a therapeutic lysosomal enzyme (rhGAA). The present study revealed a Man6P-α1,2-Man disaccharide as an essential structural motif for high-affinity binding to the M6P receptor (CI-MPR). Structure–activity relationship studies identified a tetrasaccharide oxazoline carrying this M6P disaccharide motif as the minimal donor substrate for efficient transglycosylation to give high-affinity M6P ligands. The discovery on the resistance of the M6P product to the hydrolysis by wild-type Endo-A and Endo-F3, coupled with the excellent hydrolysis activity of the wild-type enzymes on high-mannose and core-fucosylated complex type *N*-glycans, respectively, enabled a site-selective and one-pot conjugation of the high-affinity M6P glycan ligands either at the high-mannose or complex type *N*-glycosylation sites in the multiply glycosylated protein, giving structurally well-defined product. The Endo-A and Endo-F3 remodeled rhGAAs maintained full enzyme activities and demonstrated 6- and 20-fold enhanced binding affinities for CI-MPR, respectively. Moreover, by using an *in vitro* cell model system for Pompe disease, we demonstrated that the M6P-glycan remodeled rhGAA showed significantly enhanced cellular uptake over the commercial Lumizyme and exhibited much more efficient glycogen reduction in lysosomes than Lumizyme. While the therapeutic potential of the M6P glycan-remodeled rhGAA should be further evaluated in Pompe disease animal models, the present study provides a general and efficient method for site-selective M6P-glycan remodeling of recombinant lysosomal enzymes to achieve enhanced M6P receptor binding and cellular uptake, which holds a great promise for improved overall therapeutic efficacy of enzyme replacement therapy.

## Data availability

All data have been provided in the main text and the ESI.† Fig. S1–S9,† detailed synthetic procedures, compound characterization, and copies of NMR spectra are provided in the ESI.†

## Author contributions

X. Z. and L. X. W. designed the project; X. Z., H. L., C. L., and G. Z. performed the synthesis, characterization, enzymatic activity and binding assays; N. M., N. R., and R. P. performed the cell-based assays; L. X. W., N. R., and R. P. supervised the research; X. Z., N. R., and L. X. W. wrote the manuscript. All the authors analyzed the data, discussed the results, and commented on the manuscript.

## Conflicts of interest

L. X. W is the founder and a major shareholder of GlycoT Therapeutics. Other authors declare no potential conflicts of interest.

## Supplementary Material

SC-012-D1SC03188K-s001
