# Supplementary material for: Chemoenzymatic glycan-selective remodeling of a therapeutic lysosomal enzyme with high-affinity M6P-glycan ligands. Enzyme substrate specificity is the name of the game
Source: Chem Sci. 2021 Aug 19;12(37):12451–62. doi: 10.1039/d1sc03188k (PMC8480326; doi:10.1039/d1sc03188k)
Supplement: SC-012-D1SC03188K-s001 [file SC-012-D1SC03188K-s001.pdf]

Electronic Supplementary Material (ESI) for Chemical Science.
This journal is © The Royal Society of Chemistry 2021

Supporting Information

Chemoenzymatic glycan-selective remodeling of a therapeutic lysosomal enzyme with high-affinity M6P-glycan ligands. Enzyme substrate specificity is the name of the game

Xiao Zhang,[a] Huiying Liu,[a] Naresh Meena,[b] Chao Li,[a] Guanghui Zong,[a] Nina Raben,[b] Rosa Puertollano,[b] Lai-Xi Wang*[a]

[a] Department of Chemistry and Biochemistry, University of Maryland, 8051 Regents Drive, College Park, Maryland 20742, USA

[b] Cell and Developmental Biology Center, National Heart, Lung, and Blood Institute, NIH, Bethesda, MD 20892, USA

*Corresponding Author: wang518@umd.edu

Contents

1. Figure S1: HPLC and ESI-MS profiles of Glycopeptides……………………………..……..…….S3

2. Figure S2: HPLC and ESI-MS profiles of Glycoprotein…..……………………………………….S5

3. Figure S3: SPR Sensorgrams of the Binding with M6P Glycopeptides..………………………….S6

4. Figure S4: SPR Sensorgrams of the Binding with M6P Glycoprotein **68** ……………………..…….S7

5. Figure S5: Glycan Analysis of the M6P Glycan Remodeled rhGAA (**69** and **70**)……………….…….S8

6. Figure S6: Transglycosylation of CD52 with Endo-F3 ……..……….…………………………….S9

7. Figure S7: SPR Binding of the M6P Glycan Remodeled rhGAA (**69** and **70**)……………………S10

8. Figure S8: MALDI-TOF MS Analysis of the M6P Glycan Remodeled rhGAA (**69** and **70**)…….S11

9. Figure S9: Effect of the M6P Glycan Remodeled rhGAA on Lysosomal Swelling in GAA-Deficient (KO) Myotubes ………………………………………………………………………………..…S12

10. Materials and General Procedures for Chemical Synthesis…………………………………….S13

11. Synthesis of M6P Glycopeptides and Glycoproteins………..……………………….…………S41

12. Measurement of Surface Plasmon Resonance (SPR)…..……………………………….……..…S46

13. rhGAA Enzyme Activity Assay…………………………………………….……………..…S46

14. Muscle Cell Culture, Treatment, Processing, and Analysis………………………………….….S46

15. Measurement of the cell-associated GAA activity………………………………………………S47

16. Measurement of the glycogen content……….…………………………………………….….S47

17. References………………………..………………………………………………...……. S48

18. NMR Spectra……………...……………………………………………………S49 – S103

Figure S1: HPLC and ESI-MS profiles of Glycopeptides:

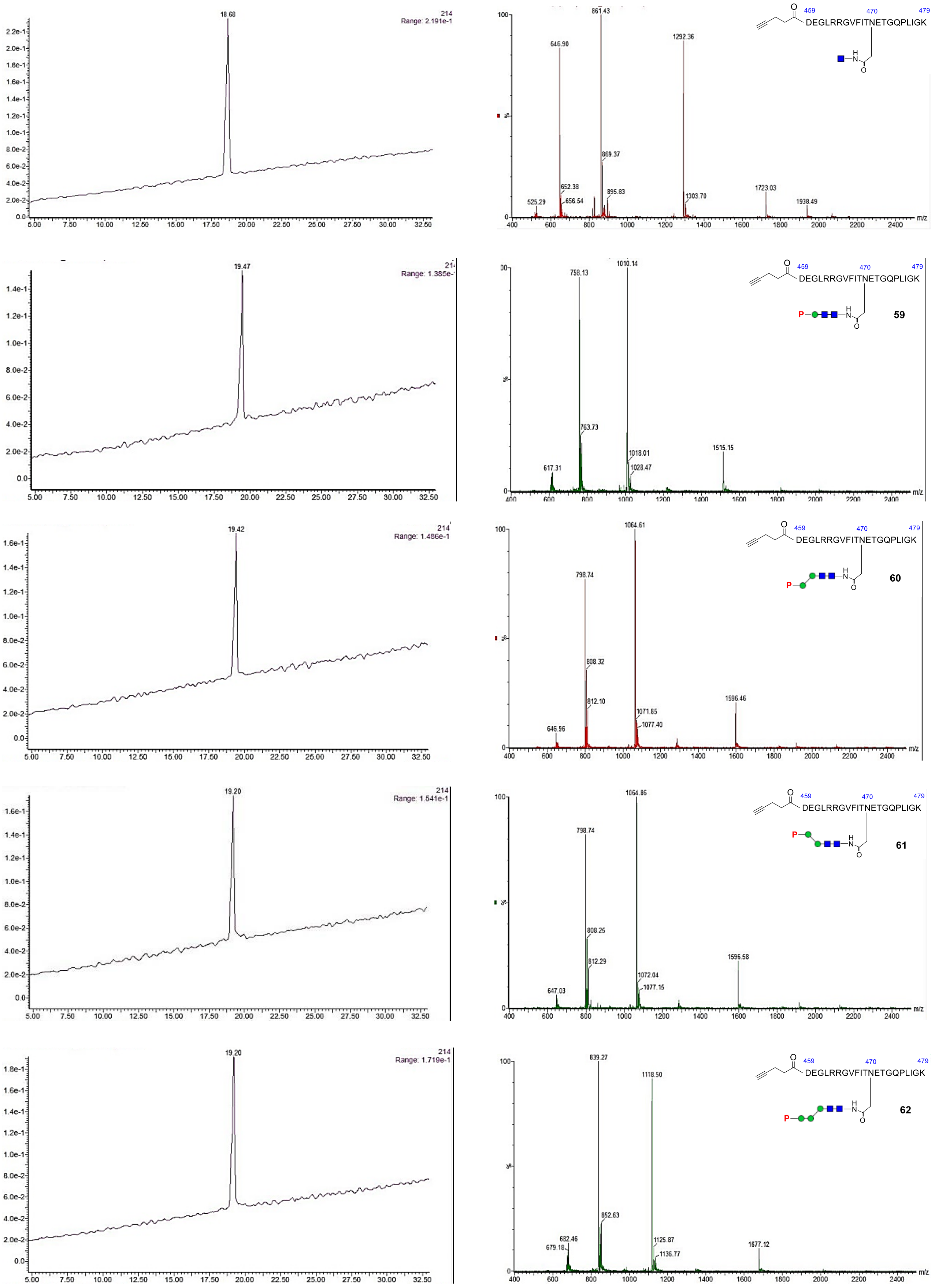

(continued)

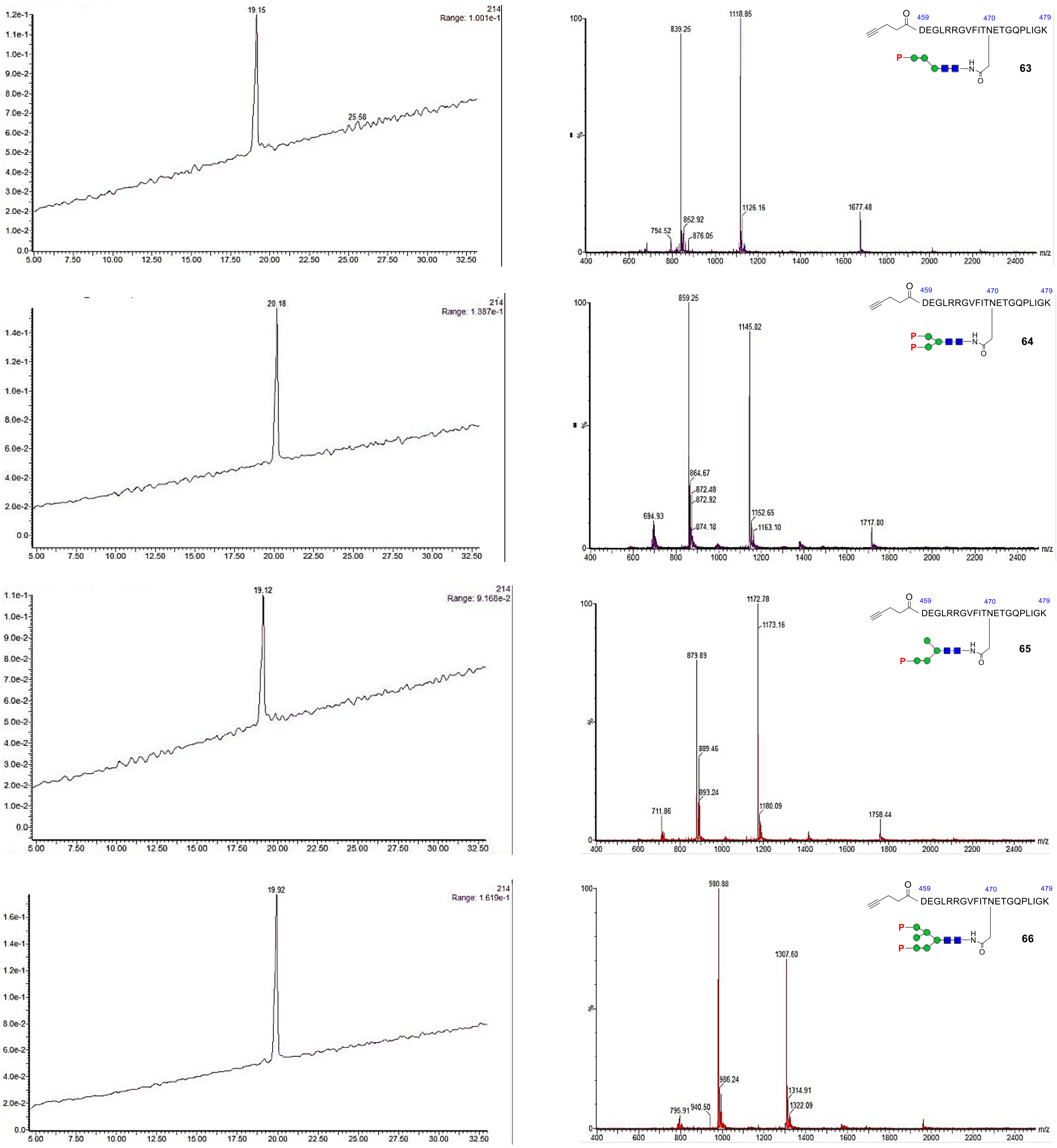

Figure S1. HPLC and ESI-MS profiles of Glycopeptides

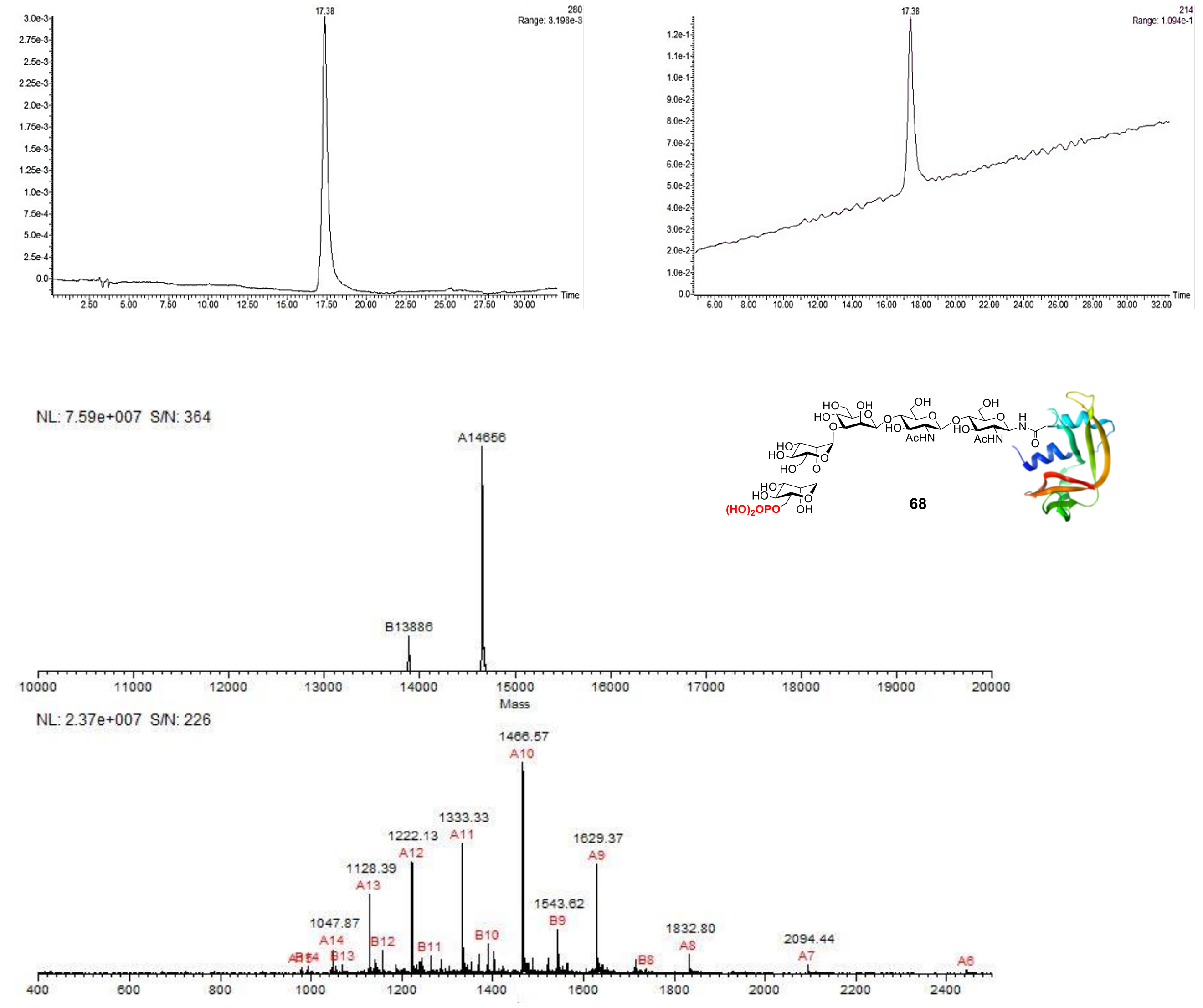

Figure S2. HPLC and ESI-MS profiles of Glycoprotein 68

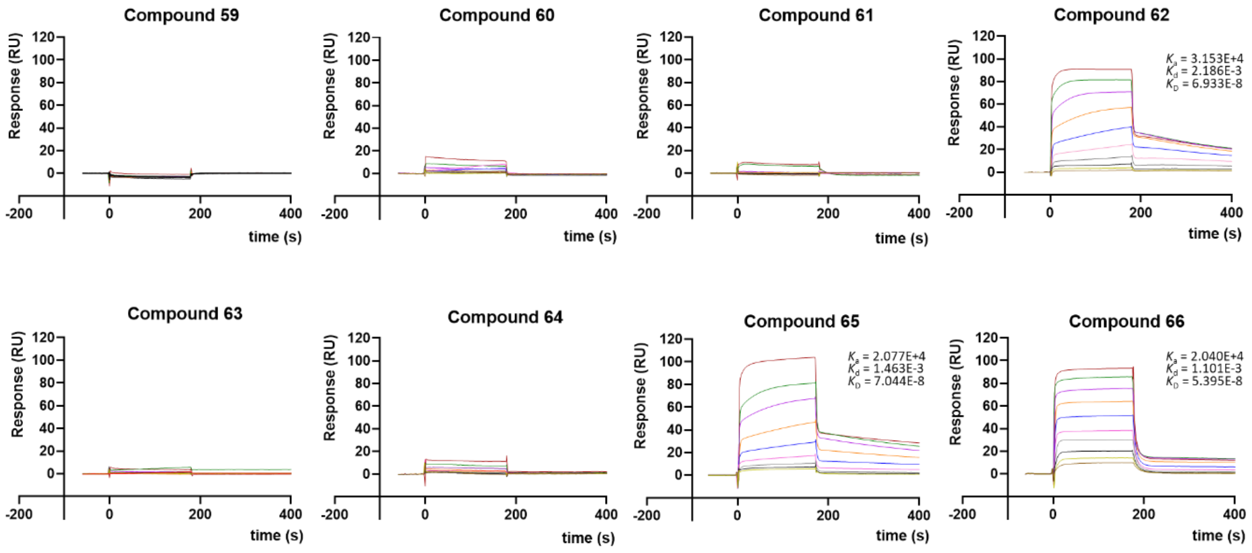

Figure S3. Representative SPR binding sensorgrams of CI-MPR with M6P-containing glycopeptides. K_D values obtained from three independent experiments for compounds **62**, **65** and **66** were 70.1 ± 2.1 nM, 82.4 ± 14.1 nM and 53.7 ± 5.6 nM, respectively.

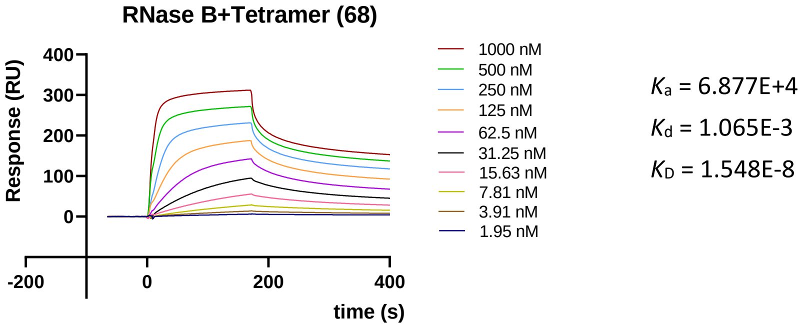

Figure S4. Representative SPR binding sensorgrams of CI-MPR with remodeled RNase B. Note: K_D value obtained from three independent experiments was 15.8 ± 1.9 nM.

Native rhGAA:

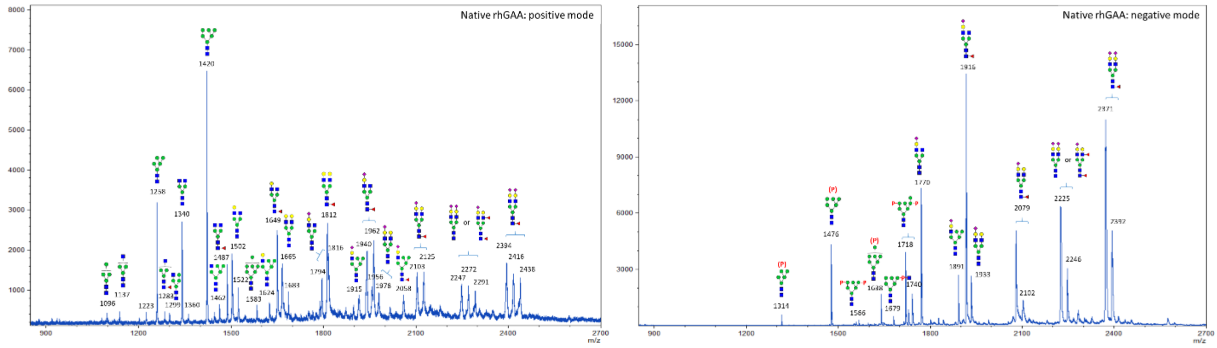

rhGAA treated with Endo-A:

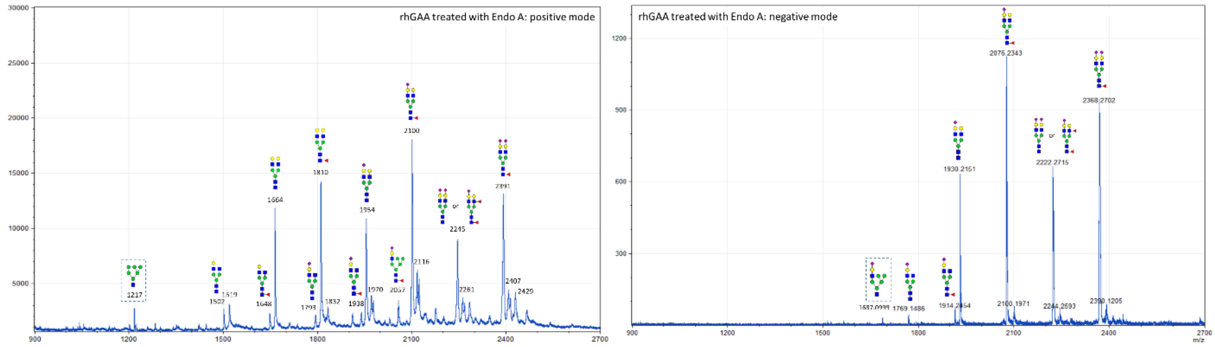

Endo-A remodeled rhGAA:

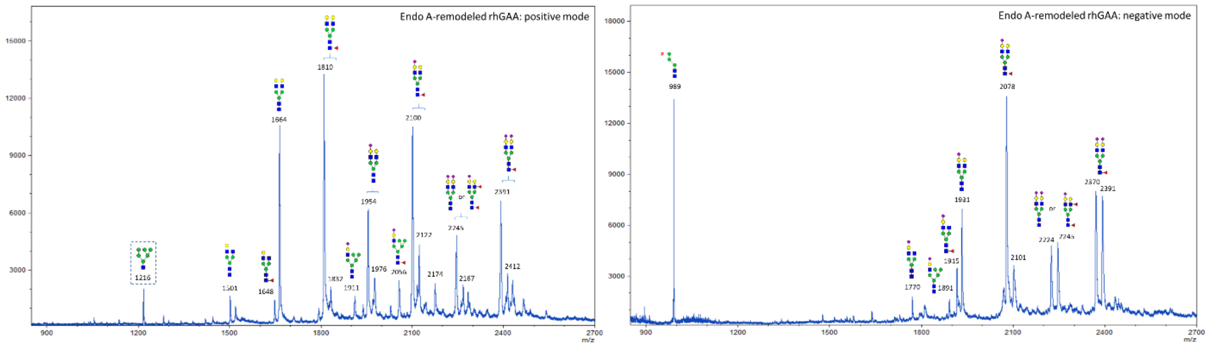

Endo-F3 remodeled rhGAA:

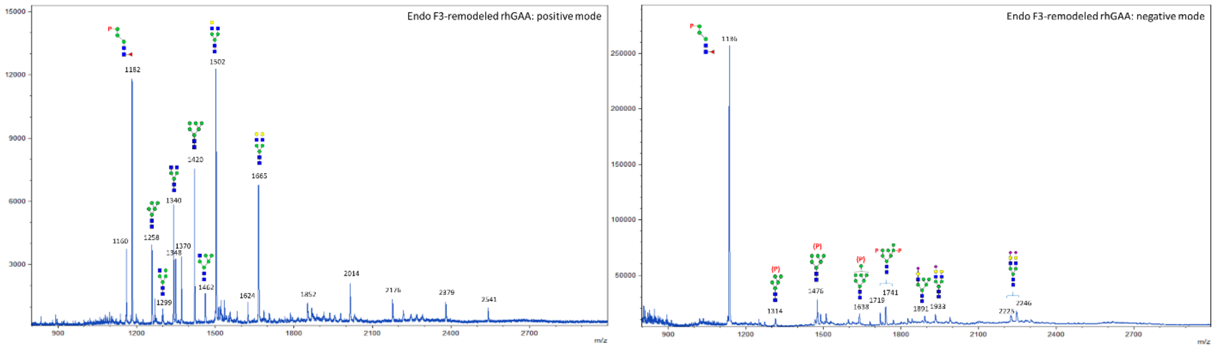

Figure S5. Glycan analysis of different stages of rhGAA. the structures marked with dotted boxes were the residual glycans that not completely removed by buffer exchange.

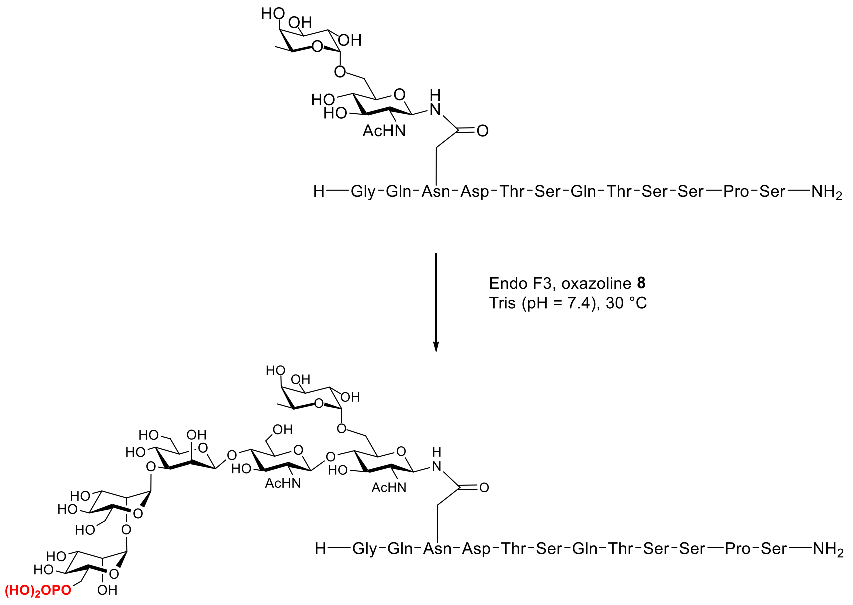

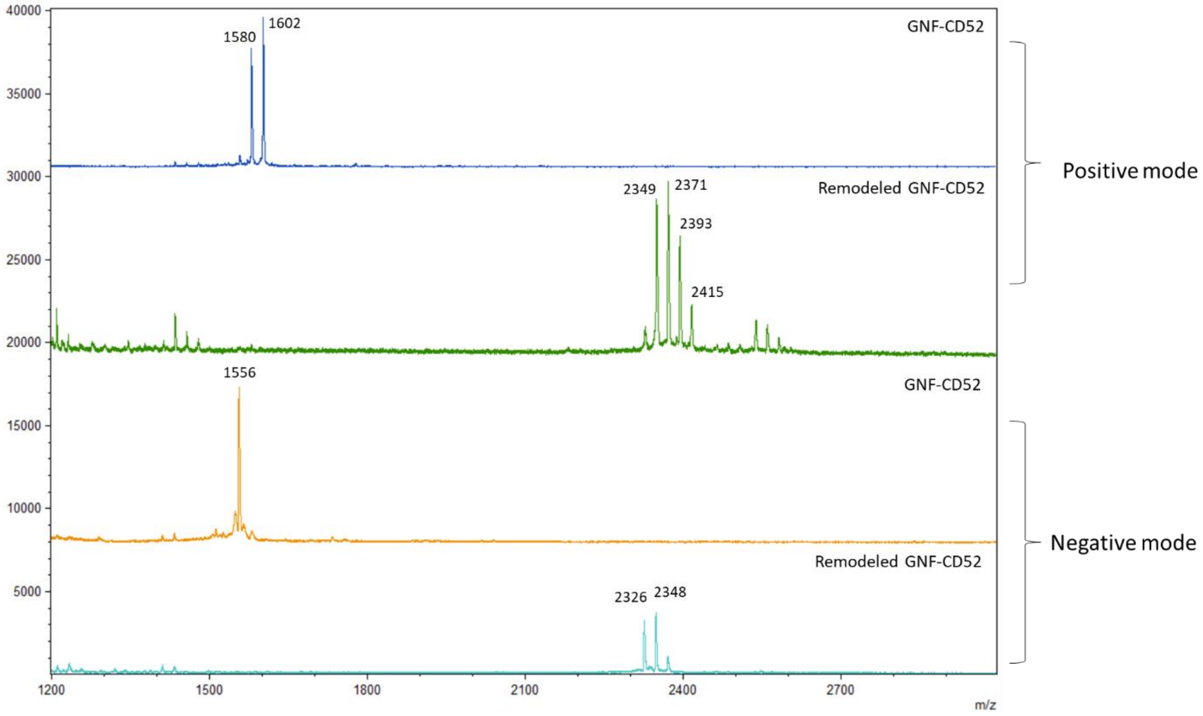

Figure S6. Transglycosylation of α1,6FucGlcNAc-CD52 with wild-type Endo F3. The reaction was complete within 30 min, and no hydrolysis of the product was observed after 5 h, indicating that Endo F3 would not cleave the newly formed glycosidic bond.

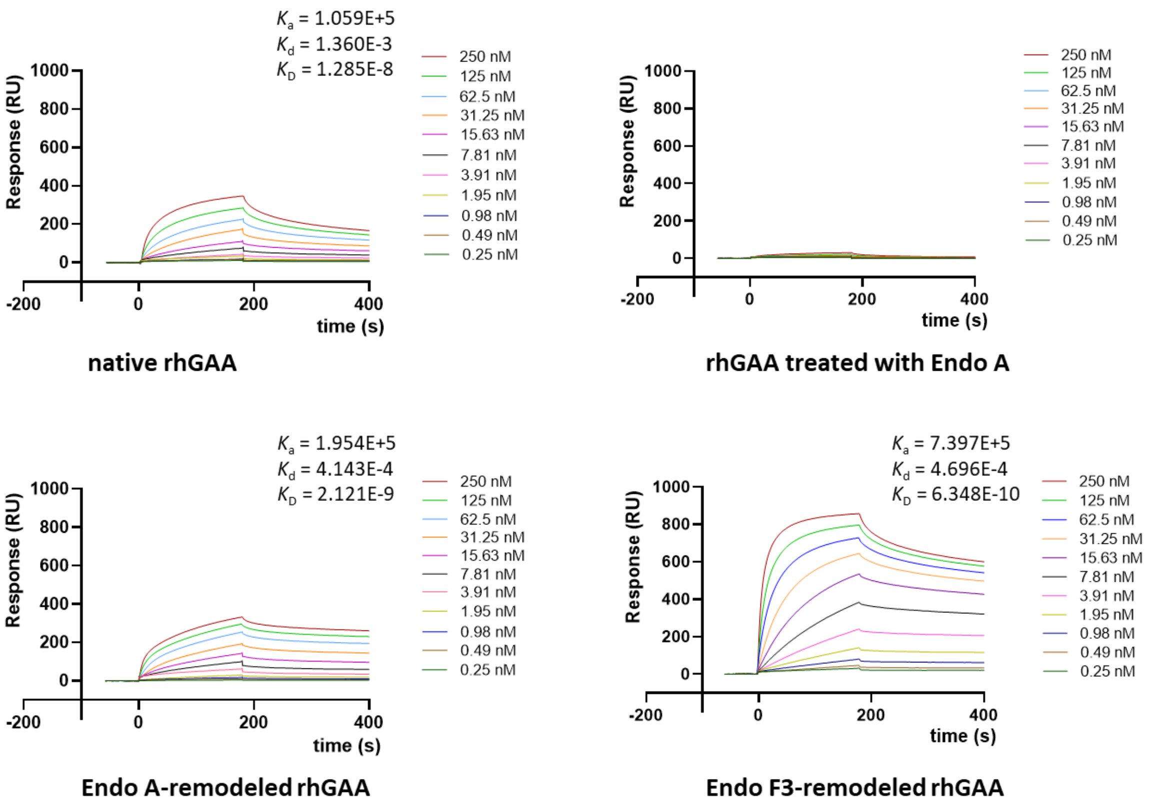

Figure S7. Representative SPR binding sensorgrams of different stages of rhGAA. Average K_D values for native rhGAA, Endo-A remodeled rhGAA (**69**) and Endo-F3 remodeled rhGAA (**70**) were 14.0 ± 3.7 nM, 2.3 ± 0.2 nM and 0.63 ± 0.07 nM, respectively.

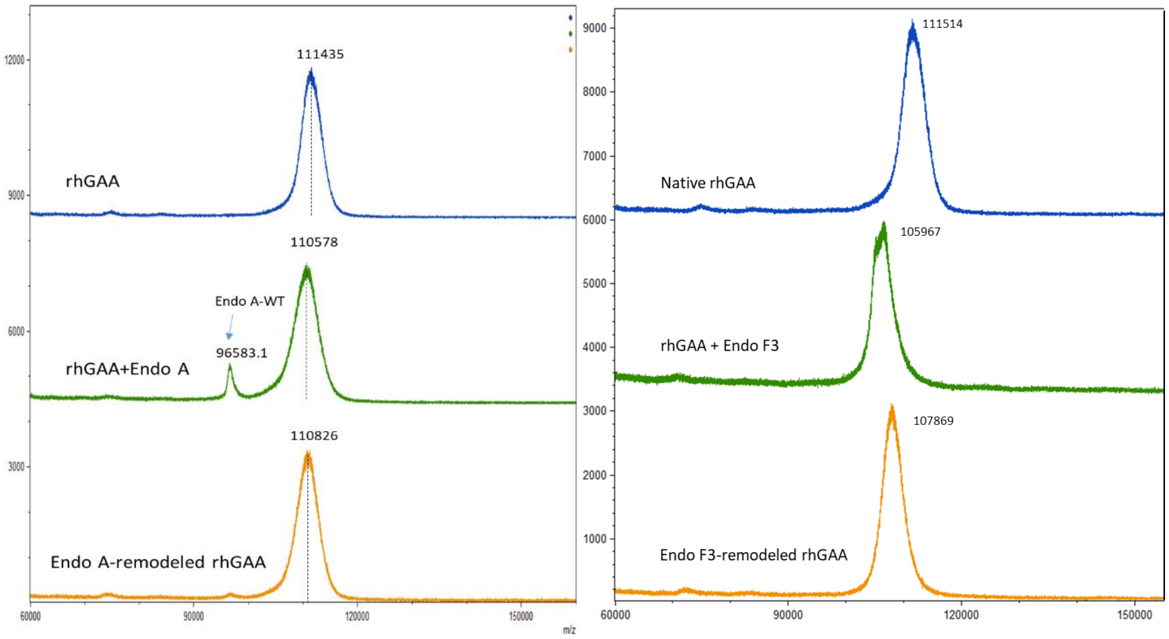

Figure S8. MALDI-TOF MS analysis of different glycoforms of rhGAA

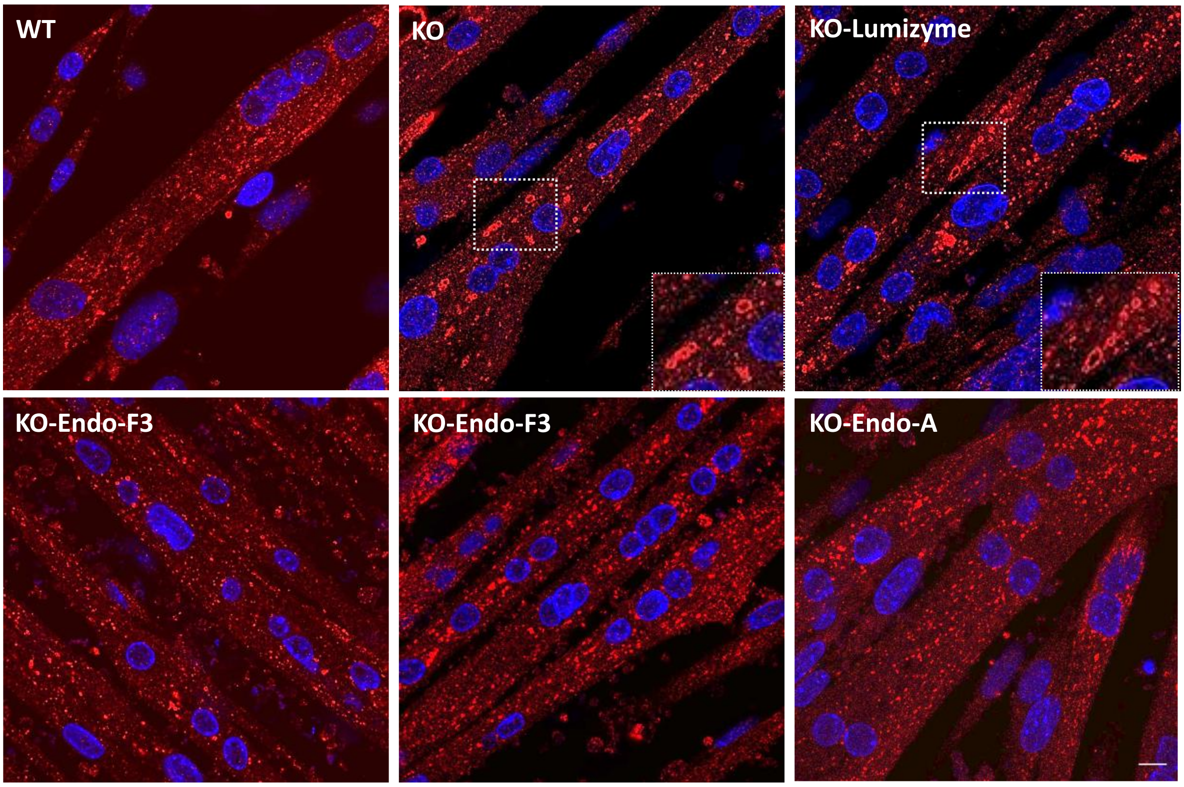

Figure S9. Effect of the therapeutic enzymes on lysosomal swelling in GAA-deficient (KO) myotubes. Confocal images of WT, untreated- (KO) and treated KO myotubes exposed to Lumizyme (KO-Lumizyme), Endo-F3 remodeled rhGAA (KO-Endo-F3), and Endo-A remodeled rhGAA (KO-Endo-A). Both untreated and Lumizyme-treated KO cells contain enlarged LAMP1-positive lysosomes (red); the structures of this size are not seen in WT and KO myotubes treated with the remodeled enzymes (KO-Endo-F3 and KO-Endo-A). Nuclei are stained with DAPI (blue). Bar:10µm for all images.

Materials and Methods.

All chemicals, reagents, and solvents were purchased from Sigma−Aldrich and TCI and unless specially noted applied in the reaction without further purification. TLC was performed using silica gel on glass plates (Sigma-Aldrich), and spots were detected under UV light (254 nm) then charring with 5 % (v/v) sulfuric acid in EtOH or cerium molybdate stain (CAM) followed by heating at 150 ºC. Silica gel (200−425 mesh) for flash chromatography was purchased from Sigma-Aldrich. NMR spectra were recorded on a 400 MHz spectrometer (Bruker, Tokyo, Japan) with $CDCl_3$ or D_2O as the solvent. The chemical shifts were assigned in ppm, and multiplicities are indicated by s (singlet), d (doublet), t (triplet), q (quartet), and m (multiplet). Coupling constants (J) are reported in Hertz. MALDI-TOF was performed on a Bruker Autoflex Speed Mass Spectrometer in positive reflectron mode with DHB (ACN/H_2O = 1:1) as the matrix. HRMS was performed on an Exactive Plus Orbitrap Mass Spectrometer (Thermo Scientific) equipped with a C18 column. Analytical RP-HPLC was performed on a Waters 626 HPLC instrument with a C18 column (3.5 μm, 4.6 × 250 mm) at 50 °C. The column was eluted with a linear gradient containing 0.1% FA for 30 min at the flow rate of 1.0 mL/min. Preparative HPLC was performed with a Waters 600 HPLC instrument and Waters C18 columns (5.0 μm, 10 × 250 mm; 7.0 μm, 19 × 300 mm). The column was eluted with a suitable gradient of MeCN−H_2O containing 0.1% TFA or FA at a flow rate of 4 mL/min or 10 mL/min. DIONEX HPAEC-PAD was performed on a Thermo Scientific Dionex ICS-6000 instrument and a PA200 column using a gradient of A (100 mM NaOH) and B (100 mM NaOH and 250 mM NaOAc) at a flow rate of 0.5 mL/min (0-60%B, 30min).

2-Azido-4,6-*O*-benzylidene-2-deoxy-αβ-D-glucopyranoside (11)

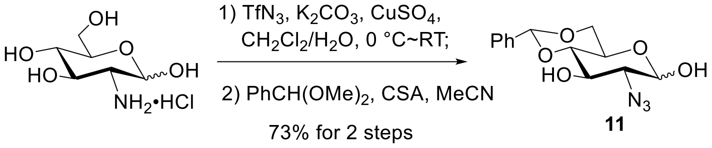

In situ preparation of TfN₃: To a vigorously stirring solution of NaN_3 (3.00 g, 46.1 mmol) in H_2O/CH_2Cl_2 (1:1, 10 mL) was dropwise added Tf_2O (1.50 mL, 8.95 mmol) at 0 °C. The resulting mixture turned into white cloudy solution and was stirred at 0 °C for a further 1.5 h. The organic layer was washed with water and saturated Na_2CO_3 (aq.), and the resulting solution of TfN_3 (in about 10 mL of CH_2Cl_2) was directly used in the next step without further purification.

Cu^{II}-catalyzed diazo transfer and protection with benzylidene: The freshly prepared solution of TfN_3 (in CH_2Cl_2) was added to a solution of D-glucosamine hydrochloride (1.00 g, 4.65 mmol), K_2CO_3 (0.75 g, 5.43 mmol), and a catalytic amount of $CuSO_4$ (10 mg) in water (5 mL) at 0 °C. Then, the ice bath was removed and MeOH was added to make the reaction homogeneous. After vigorous stirring at RT for 18 h, the mixture was passed through a pad of Celite and the filtrate was concentrated under reduced pressure to give a dry residue that was purified by a short column of silica gel (CH_2Cl_2/MeOH = 5:1~4:1) to give the crude azide product. To the crude azide product in MeCN (10 mL) was added (+)-10-Camphorsulfonic acid (195 mg, 0.84 mmol) and benzaldehyde dimethyl acetal (2.0 mL, 13.3 mmol), and the mixture was stirred at room temperature overnight. After the completion of the reaction as monitored by TLC, triethylamine was added to quench the reaction. Flash chromatography (hexanes/EtOAc = 2:1) afforded the product **11** as white solid (1.00 g, 73% for 2 steps, mixture of α/β isomers, 1.7:1). R_f = 0.20 (hexanes/EtOAc = 2:1). Spectroscopic data were in agreement with literature

values.[1]

Benzyl 2-azido-3-*O*-benzyl-4,6-*O*-benzylidene-2-deoxy-β-D-glucopyranoside (12)

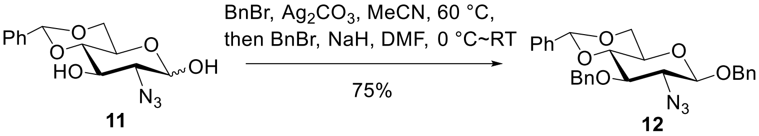

To a solution of compound **11** (952 mg, 3.25 mmol) in anhydrous acetonitrile (20 mL) was added Ag_2CO_3 (4.48 g, 16.24 mmol) and benzyl bromide (1.54 mL, 13.00 mmol), the mixture was kept in dark and heated to 60 °C overnight. When TLC showed the disappearance of the starting material, indicating the complete protection of the anomeric hydroxyl, the mixture was filtered through a pad of Celite and the filtrate was concentrated to dryness. The residue was dissolved in dry *N*,*N*-dimethylformamide (20 mL) and cooled to 0 °C, sodium hydride (325 mg, 8.13 mmol) and benzyl bromide (776 µL, 6.50 mmol) were added successively, and the mixture was slowly warmed to room temperature. After the completion of the reaction as monitored by TLC, MeOH (0.5 mL) was added to quench the excess sodium hydride. The reaction was diluted with CH_2Cl_2, successively washed with H_2O and brine and dried over anhydrous Na_2SO_4. Flash column chromatography (hexanes/EtOAc = 10:1~8:1) gave **12** (1.151 g, 75%) as white solid. R_f = 0.30 (hexanes/EtOAc = 10:1); ^{1}H NMR (400 MHz, $CDCl_3$) δ 7.54-7.52, 7.43-7.30 (15H, m, Ar-H), 5.63 (1H, s, PhC*H*), 4.97 (1H, d, PhC*H*₂, *J* = 11.8 Hz), 4.96 (1H, d, PhCH_2, *J* = 11.2 Hz), 4.84 (1H, d, PhCH_2, *J* = 11.2 Hz), 4.73 (1H, d, PhCH_2, *J* = 11.8 Hz), 4.48 (1H, d, H-1, *J* = 7.8 Hz), 4.42 (1H, dd, *J* = 10.5 Hz, *J* = 5.0 Hz), 3.86 (1H, dd, *J* = 10.3 Hz, *J* = 10.3 Hz), 3.77 (1H, dd, *J* = 9.2 Hz, *J* = 9.2 Hz), 3.61-3.56 (2H, m), 3.43 (1H, m); ^{13}C NMR (100 MHz, $CDCl_3$) δ 137.87, 137.17, 136.51, 129.10, 128.57, 128.41, 128.33, 128.23, 128.17, 128.07, 127.91, 126.04, 101.37, 101.14, 81.61, 79.06, 74.96, 71.43, 68.63, 66.25; MALDI-TOF: [M + Na]$^+$ calcd for $C_{27}H_{27}N_3NaO_5^+$, 496.18; found, 495.91.

Benzyl 2-azido-3,6-di-O-benzyl-2-deoxy-β-D-glucopyranoside (13)

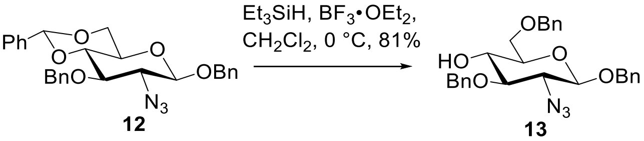

To a solution of compound **12** (500 mg, 1.056 mmol) in anhydrous CH_2Cl_2 (12 mL) was added triethylsilane (1.01 mL, 6.34 mmol) and BF_3·OEt_2 (0.67 mL, 5.28 mmol) at 0 °C, the mixture was stirred at this temperature for 3 h and quenched by triethylamine. The residue was concentrated and purified by flash column chromatography (hexanes/EtOAc = 10:1~4:1) to give **13** (406 mg, 81%) as colorless syrup. R_f = 0.30 (hexanes/EtOAc = 4:1); ^{1}H NMR (400 MHz, $CDCl_3$) δ 7.44-7.35 (15H, m, Ar-H), 4.98-4.93 (2H, m, PhCH_2), 4.81 (1H, d, PhCH_2, *J* = 11.3 Hz), 4.72 (1H, d, PhCH_2, *J* = 11.9 Hz), 4.68-4.59 (2H, m, PhCH_2), 4.40 (1H, d, H-1, *J* = 8.1 Hz), 3.79 (2H, m), 3.69 (1H, m), 3.52-3.42 (2H, m), 3.28 (1H, dd, *J* = 9.3 Hz, *J* = 9.3 Hz), 2.70 (1H, m); ^{13}C NMR (100 MHz, $CDCl_3$) δ 138.13, 137.76, 136.77, 128.64, 128.53, 128.51, 128.15, 128.07, 128.05, 128.02, 127.90, 127.77, 100.64, 82.62, 75.13, 74.06, 73.77, 71.96, 71.02, 70.17, 65.79; MALDI-TOF: [M + Na]$^+$ calcd for $C_{27}H_{29}N_3NaO_5^+$, 498.20; found, 498.18.

Benzyl 2-*O*-benzyl-4,6-*O*-benzylidene-3-*O*-*p*-methoxybenzyl-β-D-mannopyranosyl-(1→4)-2-

azido-3,6-di-*O*-benzyl-2-deoxy-β-D-glucopyranoside (15)

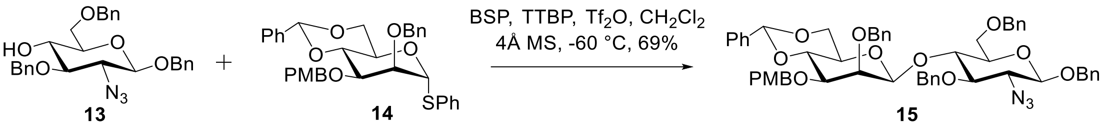

To a solution of compound **14** [2] (581 mg, 1.019 mmol) in anhydrous CH_2Cl_2 (18 mL) was added activated 4Å molecular sieves (2.0 g) under argon atmosphere, the mixture was stirred for 2 h at room temperature, then cooled to -60 °C. BSP (236 mg, 1.13 mmol) and TTBP (427 mg, 2.05 mmol) were added, and the solution was kept at -60 °C for 40 min before Tf_2O (206 μL, 1.23 mmol) was added. After 20 min, a solution of compound **13** (323 mg, 0.68 mmol) in CH_2Cl_2 (3.0 mL) was added and the mixture was stirred at -60 °C for 3 h. After the completion of the reaction as monitored by TLC, the mixture was filtered through a Celite pad. The filtrate was poured into saturated NaHCO3 and extracted with CH_2Cl_2. The organic layer was washed with brine, dried over Na_2SO_4, and concentrated. The residue was purified by column chromatography (hexanes/EtOAc = 10:1~5:1) to afford **15** (441 mg, 69%) as a colorless syrup. R_f = 0.40 (hexanes/EtOAc = 4:1); Spectroscopic data were in agreement with literature values.[3] MALDI-TOF: $[M + Na]^+$ calcd for $C_{55}H_{57}N_3NaO_{11}^+$, 958.39; found, 958.53.

Benzyl 2,4-di-*O*-benzyl-3-*O*-*p*-methoxybenzyl-β-D-mannopyranosyl-(1→4)-2-azido-3,6-di-*O*-benzyl-2-deoxy-β-D-glucopyranoside (16)

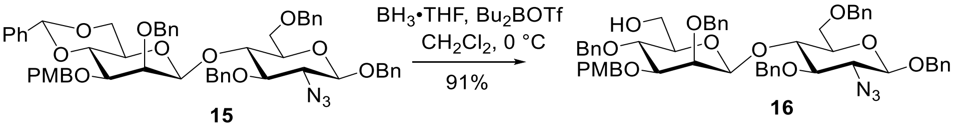

To a solution of compound **15** (300 mg, 0.321 mmol) in BH_3·THF (4.0 mL) was added a solution of Bu_2BOTf in CH_2Cl_2 (1 M, 642 µL) under argon atmosphere at 0 °C and the mixture was stirred at 0 °C for 40 min when TLC indicated the completion of the reaction. Et_3N (300 µL) was added to the reaction followed by careful addition of MeOH (600 µL). The mixture was co-evaporated with MeOH three times and the residue was purified by flash chromatography (hexanes/EtOAc = 5:1~2:1) to afford **16** (274 mg, 91%) as a colorless syrup. R_f = 0.30 (hexanes/EtOAc = 3:1); ^{1}H NMR (400 MHz, $CDCl_3$) δ 7.50-7.28 (27H, m, Ar-H), 6.93 (2H, m, Ar-H), 5.16 (1H, d, PhC*H*2, *J* = 10.8 Hz), 5.01 (1H, d, PhC*H*2, *J* = 12.1 Hz), 4.97-4.86 (3H, m, PhC*H*2), 4.78 (1H, d, PhC*H*2, *J* = 12.1 Hz), 4.75-4.64 (3H, m, PhC*H*2), 4.55-4.52 (3H, m, PhC*H*2), 4.40 (1H, d, *J* = 8.1 Hz), 4.01 (1H, dd, *J* = 9.3 Hz, *J* = 9.3 Hz), 3.89-3.75 (6H, m), 3.73-3.67 (2H, m), 3.57 (1H, dd, *J* = 8.2 Hz, *J* = 8.2 Hz), 3.45-3.39 (4H, m), 3.22-3.18 (1H, m), 1.97 (1H, s); ^{13}C NMR (100 MHz, $CDCl_3$) δ 159.31, 138.76, 138.52, 138.47, 137.75, 136.93, 130.38, 129.20, 128.64, 128.56, 128.45, 128.38, 128.26, 128.17, 128.06, 128.03, 127.91, 127.78, 127.69, 127.65, 127.51, 113.89, 100.76, 100.51, 82.34, 81.50, 77.08, 75.80, 75.31, 75.10, 74.90, 74.83, 74.57, 73.71, 71.65, 70.93, 68.54, 65.96, 62.22, 55.34; MALDI-TOF: $[M + Na]^+$ calcd for $C_{55}H_{59}N_3NaO_{11}^+$, 960.40; found, 959.98.

Benzyl 2,4-di-*O*-benzyl-3-*O*-*p*-methoxybenzyl-β-D-mannopyranosyl-(1→4)-2-acetamido-3,6-di-*O*-benzyl-2-deoxy-β-D-glucopyranoside (17)

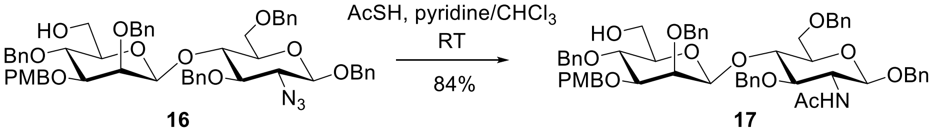

A solution of compound **16** (133.5 mg, 0.142 mmol) in a mixture of AcSH/pyridine/$CHCl_3$ (0.8 mL/0.6 mL/0.8 mL) was stirred at room temperature for 18 h. After the completion of the reaction as monitored by TLC, the resulting mixture was concentrated and subjected to flash chromatography on silica gel (hexanes/EtOAc = 4:1~3:2) to afford compound **17** (113.8 mg, 84%) as colorless syrup. R_f = 0.30 (hexanes/EtOAc = 2:1); ^{1}H NMR (400 MHz, $CDCl_3$) δ 7.44-7.43, 7.37-7.23 (27H, m, Ar-H), 6.88-6.86 (2H, m, Ar-H), 5.75 (1H, d, N*H*, *J* = 8.0 Hz), 4.98-4.81 (6H, m, PhC*H*2), 4.65-4.59 (4H, m, PhC*H*2), 4.53-4.47 (4H, m, PhC*H*2), 4.16 (1H, dd, *J* = 7.8 Hz, *J* = 7.8 Hz), 3.90 (1H, dd, *J* = 7.0 Hz, *J* = 7.0 Hz), 3.86-3.78 (6H, m), 3.73-3.69 (3H, m), 3.64-3.58 (1H, m), 3.53-3.48 (1H, m), 3.42-3.39 (1H, m), 3.22-3.18 (1H, m), 2.09 (1H, s), 1.76 (3H, s); ^{13}C NMR (100 MHz, $CDCl_3$) δ 170.54, 159.25, 138.78, 138.63, 138.37, 137.91, 137.60, 130.28, 129.17, 128.51, 128.39, 128.33, 128.26, 128.15, 128.02, 127.88, 127.74, 127.66, 127.61, 127.54, 113.84, 101.08, 99.14, 82.18, 77.78, 75.71, 75.47, 75.13, 75.08, 74.76, 74.62, 73.75, 73.57, 71.59, 70.71, 69.38, 62.28, 55.29, 23.38; MALDI-TOF: $[M + Na]^+$ calcd for $C_{57}H_{63}NNaO_{12}^+$, 976.42; found, 976.00.

Benzyl 2,4-di-*O*-benzyl-6-*O*-dibenzylphosphonato-3-*O*-*p*-methoxybenzyl-β-D-mannopyranosyl-(1→4)-2-acetamido-3,6-di-*O*-benzyl-2-deoxy-β-D-glucopyranoside (18)

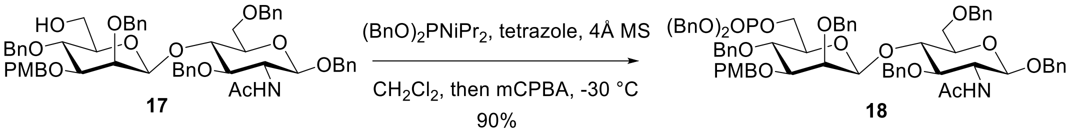

To a solution of compound **17** (50.0 mg, 0.052 mmol) in anhydrous CH_2Cl_2 (2.0 mL) was added activated 4Å molecular sieves (200 mg) and tetrazole (0.45 M in MeCN, 582 µL) and the mixture was stirred at room temperature for 1.5 h before $(BnO)_2PNiPr_2$ (70.6 µL) was added. The resulting mixture was further stirred overnight under argon atmosphere at room temperature until the complete disappearance of the starting material. Then the reaction was cooled to -30 °C, and mCPBA (77 wt %, 61.5 mg) was added, the reaction mixture was stirred at this temperature for 1 h and then filtered through a Celite pad. The filtrate was diluted with CH_2Cl_2, washed with saturated $NaHCO_3$ (aq.), dried over Na_2SO_4 and concentrated to dryness. The residue was purified by flash chromatography to give compound **18** (56.8 mg, 90%) as colorless syrup. R_f = 0.30 (hexanes/Acetone = 2:1); ^{1}H NMR (400 MHz, $CDCl_3$) δ 7.42-7.40, 7.34-7.20 (37H, m, Ar-H), 6.87-6.85 (2H, m, Ar-H), 5.96 (1H, d, N*H*, *J* = 8.3 Hz), 4.99-4.92 (4H, m, PhC*H*2), 4.89 (1H, d, *J* = 11.9 Hz, PhC*H*2), 4.88 (1H, d, *J* = 10.8 Hz, PhC*H*2), 4.85-4.82 (3H, m, PhC*H*2), 4.79 (1H, d, *J* = 11.8 Hz, PhC*H*2), 4.65 (1H, d, *J* = 11.8 Hz, PhC*H*2), 4.60-4.55 (3H, m), 4.52 (1H, d, *J* = 11.7 Hz, PhC*H*2), 4.47-4.41 (2H, m), 4.25-4.20 (1H, m), 4.19-4.12 (1H, m), 4.06 (1H, dd, *J* = 7.1 Hz, *J* = 7.1 Hz), 3.97 (1H, dd, *J* = 6.9 Hz, *J* = 6.9 Hz), 3.85-3.79 (6H, m), 3.77-3.71 (2H, m), 3.40 (1H, dd, *J* = 2.8 Hz, *J* = 9.3 Hz), 3.35-3.32 (1H, m), 1.69 (3H, s); ^{13}C NMR (100 MHz, $CDCl_3$) δ 170.07, 159.30, 138.87, 138.51, 138.17, 138.08, 137.80, 135.85, 135.78, 130.05, 129.24, 128.44, 128.39, 128.29, 128.24, 128.21, 128.11, 128.05, 128.03, 127.95, 127.93, 127.77, 127.73, 127.69, 127.58, 127.54, 127.38, 113.85, 100.92, 99.61, 81.98, 77.19, 77.13, 75.19, 75.11, 74.93, 74.54, 74.38, 74.30, 73.86, 73.49, 72.73, 71.49, 70.45, 69.68, 69.34, 69.29, 66.68, 66.63, 55.28, 53.58, 23.17; ^{31}P NMR (146 MHz, $CDCl_3$) δ -1.20; MALDI-TOF: $[M + Na]^+$ calcd for $C_{71}H_{76}NNaO_{15}P^+$, 1236.48; found, 1236.25.

6-*O*-phosphonato-β-D-mannopyranosyl-(1→4)-2-acetamido-2-deoxy-αβ-D-glucopyranoside (19)

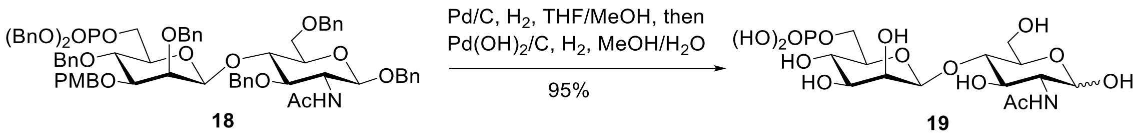

A mixture of compound **18** (56.8 mg, 0.047 mmol) and Pd/C (10 wt.% loading, 30 mg) in MeOH (2.0 mL) and THF (2.0 mL) was stirred under H_2 atmosphere for 21 h. The reaction mixture was filtered through a Celite pad, then concentrated to dryness. The mixture of the residue and $Pd(OH)_2/C$ (20 wt.% loading, 30 mg) in MeOH (2.5 mL) and H_2O (2.5 mL) was stirred under H_2 atmosphere for further 21 h. The reaction mixture was filtered through a Celite pad. The filtrate was concentrated to dryness then dissolved in H_2O and lyophilized. The crude product was purified on a Sephadex G-10 column by elution with H_2O. Fractions containing the product were pooled and lyophilized to give compound **19** (20.5 mg, 95%) as white solid. R_f = 0.50 (n-BuOH/EtOH/H_2O/AcOH = 1:1:1:0.05); ^{1}H NMR (400 MHz, D_2O) δ 5.15 (0.54H, d, J = 3.4 Hz), 4.65 (0.44H, d, J = 8.2 Hz), 4.05-3.97 (2.15H, m), 3.96-3.92 (1.07H, m), 3.90-3.86 (1.28H, m), 3.83-3.78 (1.12H, m), 3.77-3.74 (0.76H, m), 3.73-3.71 (0.53H, m), 3.71-3.68 (0.77H, m), 3.68-3.63 (2.64H, m), 3.62-3.59 (1.06H, m), 3.54-3.51 (0.44H, m), 3.45-3.41 (0.99H, m), 1.97 (3H, s); ^{13}C NMR (100 MHz, D_2O) δ 174.36, 174.06, 100.01, 99.86, 94.55, 90.04, 79.36, 78.89, 75.10, 75.05, 74.21, 72.10, 71.76, 70.20, 70.17, 69.63, 68.64, 65.72, 63.06, 59.97, 59.83, 55.80, 53.35, 21.81, 21.50; ^{31}P NMR (146 MHz, D_2O) δ 2.76 (overlapped signals); HRMS: $[M + H]^+$ calcd for C14H27NO14P$^+$, 464.1164; found, 464.1169.

2-Methyl-[6-*O*-phosphonato-β-D-mannopyranosyl-(1→4)-1,2-dideoxy-α-D-glucopyrano]-[2,1-*d*]-2-oxazoline (5).

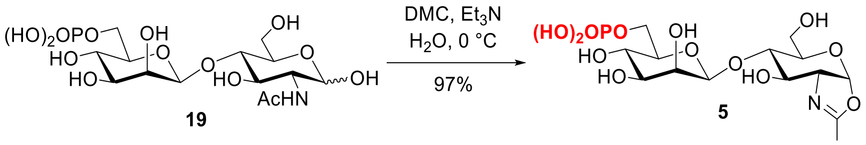

To a solution of compound **19** (5.0 mg, 0.011 mmol) in H_2O (300 µL) were added Et_3N (60.5 µL) and 2-chloro-1,3-dimethylimidazolinium chloride (DMC, 36.6 mg) at 0 °C. The reaction mixture was monitored by DIONEX HPAEC-PAD. After 2 h, the HPAEC analysis indicated that the free oligosaccharide was converted into a new oligosaccharide that was eluted earlier than the reducing sugar under the HPAEC condition (see general method). The product was purified by gel filtration on a Sephadex G-10 column that was eluted with 0.1% aq Et_3N to afford compound **5** (4.7 mg, 97%) as white solid after lyophilization with 5 mol.% of NaOH. ^{1}H NMR (400 MHz, D_2O) δ 6.01 (1H, d, J = 7.3 Hz), 4.63 (1H, m), 4.36-4.47 (1H, m), 4.13-4.11 (1H, m), 3.99-3.89 (3H, m), 3.72-3.59 (3H, m), 3.58-3.54 (2H, m), 3.38-3.32 (2H, m), 1.99 (3H, d, J = 1.7 Hz); ^{13}C NMR (100 MHz, D_2O) δ 168.70, 101.34, 99.88, 77.48, 75.88, 75.81, 72.57, 71.02, 70.50, 68.99, 66.54, 65.36, 63.25, 63.21, 61.82, 12.96; ^{31}P NMR (146 MHz, D_2O) δ 4.44. HRMS: $[M + H]^+$ calcd for C14H25NO13P$^+$, 446.1058; found, 446.1064.

Benzyl 2-*O*-benzyl-4,6-*O*-benzylidene-β-D-mannopyranosyl-(1→4)-2-azido-3,6-di-*O*-benzyl-2-deoxy-β-D-glucopyranoside (20).

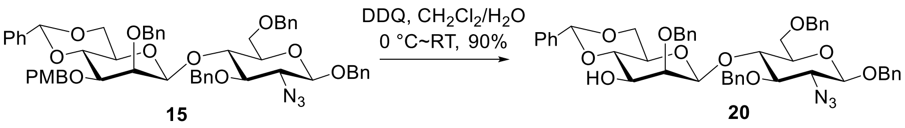

To a solution of **15** (454 mg, 0.485 mmol) in a mixture of CH_2Cl_2/H_2O (15 mL/1 mL) was added DDQ (252 mg, 1.11 mmol) at 0 °C. After 30 min, the reaction mixture was warmed to room temperature and further stirred for 1 h. The reaction mixture was diluted with CH_2Cl_2, washed with saturated $NaHCO_3$ (aq.) and brine, and dried over Na_2SO_4. Concentration and purification by column chromatography on silica gel (hexanes/EtOAc = 6:1~3:1) provided **20** (356 mg, 90%) as a white amorphous solid. R_f = 0.25 (hexanes/EtOAc = 3:1); Spectroscopic data were in agreement with literature values.[3] MALDI-TOF: $[M + Na]^+$ calcd for $C_{47}H_{49}N_3NaO_{10}^+$, 838.33; found, 838.47.

Benzyl 3,4-di-*O*-benzyl-2-*O*-benzoyl-6-*O*-triisopropylsilyl-α-D-mannopyranosyl-(1→6)-2,4-di-*O*-benzyl-3-*O*-*p*-methoxybenzyl-β-D-mannopyranosyl-(1→4)-2-azido-3,6-di-*O*-benzyl-2-deoxy-β-D-glucopyranoside (23)

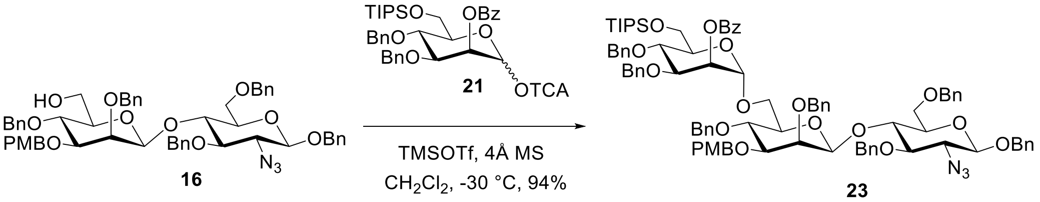

A mixture of trichloroacetimidate donor **21** [4] (100 mg, 0.13 mmol), acceptor **16** (63 mg, 0.067 mmol) and activated 4Å molecular sieves (200 mg) in anhydrous CH_2Cl_2 (2.0 mL) was stirred at room temperature under argon atmosphere for 1.5 h, and then cooled to -30 °C. TMSOTf (1.0 µL, 5.5 µmol) was added. After stirring at -30 °C for 50 min, the mixture was quenched with triethylamine (20 µL) and filtered then concentrated in vacuo. The residue was purified via silica gel chromatography (hexanes/EtOAc = 10:1~4:1) to give product **23** (97.6 mg, 94%) as white foam. R_f = 0.50 (hexanes/EtOAc = 3:1); ^{1}H NMR (400 MHz, $CDCl_3$) δ 8.10-8.08, 7.58, 7.45-7.13 (42H, m, Ar-H), 6.88-6.86 (2H, m, Ar-H), 5.66-5.64 (1H, m), 5.10 (1H, d, J = 11.4 Hz, PhCH_2), 4.95-4.77 (7H, m, PhCH_2), 4.70-4.57 (5H, m), 4.52-4.39 (4H, m), 4.34-4.31 (2H, m), 4.08 (1H, dd, J = 9.5 Hz, J = 9.5 Hz), 4.02-3.97 (2H, m), 3.91-3.86 (2H, m), 3.82-3.76 (5H, m), 3.72-3.68 (3H, m), 3.63-3.59 (2H, m), 3.52 (1H, dd, J = 8.3 Hz, J = 9.7 Hz), 3.43-3.33 (4H, m), 1.09 (21H, s); ^{13}C NMR (100 MHz, $CDCl_3$) δ 165.56, 159.28, 139.08, 138.90, 138.61, 138.51, 138.15, 138.01, 137.00, 132.97, 130.24, 130.16, 130.05, 129.29, 128.56, 128.46, 128.31, 128.21, 128.18, 128.14, 128.01, 127.93, 127.85, 127.78, 127.67, 127.61, 127.52, 127.43, 127.41, 127.36, 127.26, 113.85, 101.38, 100.73, 97.98, 82.63, 80.94, 78.23, 77.30, 75.04, 74.91, 74.85, 74.49, 74.44, 74.07, 73.87, 73.58, 72.75, 71.46, 71.31, 70.93, 69.05, 68.74, 66.73, 65.97, 62.30, 55.30, 18.10, 18.07, 12.06; MALDI-TOF: $[M + Na]^+$ calcd for $C_{91}H_{105}N_3NaO_{17}Si^+$, 1562.71; found, 1563.13.

Benzyl 3,4-di-*O*-benzyl-2-*O*-benzoyl-6-*O*-triisopropylsilyl-α-D-mannopyranosyl-(1→3)-2-*O*-benzyl-4,6-*O*-benzylidene-β-D-mannopyranosyl-(1→4)-2-azido-3,6-di-*O*-benzyl-2-deoxy-β-D-glucopyranoside (24)

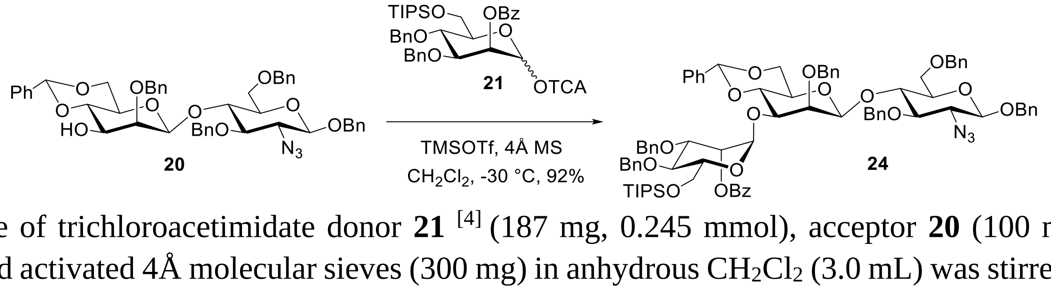

A mixture of trichloroacetimidate donor **21** [4] (187 mg, 0.245 mmol), acceptor **20** (100 mg, 0.123 mmol) and activated 4Å molecular sieves (300 mg) in anhydrous CH_2Cl_2 (3.0 mL) was stirred at room

temperature under argon atmosphere for 1.5 h, and then cooled to -30 °C. TMSOTf (2.1 μL, 12.3 μmol) was added. After stirring at -30 °C for 1 h, the mixture was quenched with triethylamine (20 μL) and filtered then concentrated in vacuo. The residue was purified via silica gel chromatography (hexanes/EtOAc = 15:1~5:1) to give product **23** (159.4 mg, 92%) as white foam. R_f = 0.30 (hexanes/EtOAc = 8:1); ^{1}H NMR (400 MHz, $CDCl_3$) δ 8.01-7.98, 7.49, 7.36-7.09 (40H, m, Ar-H), 5.74 (1H, m), 5.45 (1H, s), 5.30 (1H, m), 4.96 (1H, m), 4.85-4.82 (2H, m), 4.71-4.48 (8H, m), 4.38-4.35 (2H, m), 4.22-4.20 (1H, m), 4.04-3.88 (6H, m), 3.83-3.80 (1H, m), 3.72-3.70 (1H, m), 3.68-3.63 (2H, m), 3.60-3.57 (1H, m), 3.51-3.48 (1H, m), 3.42-3.35 (2H, m), 3.28-3.21 (2H, m), 2.99-2.96 (1H, m), 1.00 (21H, s); ^{13}C NMR (100 MHz, $CDCl_3$) δ 165.53, 138.85, 138.55, 138.27, 138.07, 137.66, 137.22, 136.87, 133.11, 130.00, 128.61, 128.51, 128.29, 128.17, 128.11, 128.05, 128.00, 127.97, 127.93, 127.84, 127.80, 127.72, 127.55, 125.90, 101.05, 101.01, 100.51, 98.76, 81.61, 78.79, 78.52, 78.16, 76.95, 75.66, 75.55, 75.23, 75.06, 74.98, 73.89, 73.69, 71.48, 70.90, 68.75, 68.38, 68.32, 67.03, 65.82, 62.76, 18.11, 12.07; MALDI-TOF: $[M + Na]^+$ calcd for $C_{91}H_{105}N_3NaO_{17}Si^+$, 1440.64; found, 1440.07.

Benzyl 2,3,4-tri-*O*-benzyl-6-*O*-triisopropylsilyl-α-D-mannopyranosyl-(1→6)-2,4-di-*O*-benzyl-3-*O*-*p*-methoxybenzyl-β-D-mannopyranosyl-(1→4)-2-azido-3,6-di-*O*-benzyl-2-deoxy-β-D-glucopyranoside (25)

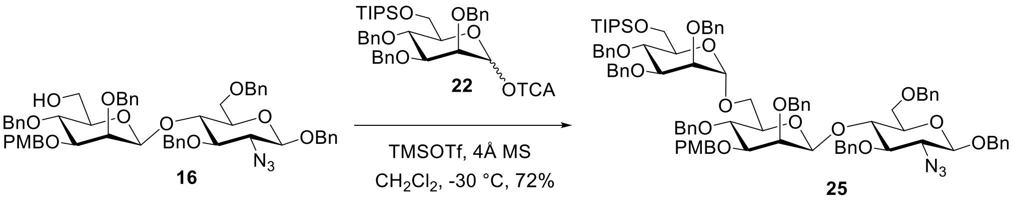

A mixture of trichloroacetimidate donor **22** [5] (100 mg, 0.133 mmol), acceptor **16** (63 mg, 0.067 mmol) and activated 4Å molecular sieves (200 mg) in anhydrous CH_2Cl_2 (2.0 mL) was stirred at room temperature under argon atmosphere for 1.5 h, and then cooled to -30 °C. TMSOTf (1.0 μL, 5.5 μmol) was added. After stirring at -30 °C for 50 min, the mixture was quenched with triethylamine (20 μL) and filtered then concentrated in vacuo. The residue was purified via silica gel chromatography (hexanes/EtOAc = 10:1~4:1) to give the desired α-isomer **25** (74.3 mg, 72%) as white foam. R_f = 0.40 (hexanes/EtOAc = 4:1); ^{1}H NMR (400 MHz, $CDCl_3$) δ 7.38-7.19 (42H, m, Ar-H), 6.87-6.85 (2H, m, Ar-H), 5.00 (1H, d, J = 11.7 Hz, PhCH_2), 4.96-4.79 (7H, m, PhCH_2), 4.69 (1H, d, J = 12.0 Hz, PhCH_2), 4.65-4.55 (4H, m), 4.50-4.40 (6H, m), 4.29 (1H, d, J = 8.0 Hz), 4.01 (1H, dd, J = 9.5 Hz, J = 9.5 Hz), 3.97-3.89 (2H, m), 3.87-3.74 (9H, m), 3.72-3.62 (2H, m), 3.58-3.53 (2H, m), 3.46 (1H, dd, J = 8.1 Hz, J = 9.7 Hz), 3.39 (1H, dd, J = 2.7 Hz, J = 9.4 Hz), 3.37-3.32 (2H, m), 3.27-3.24 (1H, m), 1.06 (21H, s); ^{13}C NMR (100 MHz, $CDCl_3$) δ 159.25, 139.17, 138.97, 138.92, 138.59, 138.56, 138.52, 137.99, 136.92, 130.28, 129.20, 128.51, 128.45, 128.28, 128.23, 128.22, 128.16, 128.14, 128.06, 127.92, 127.80, 127.77, 127.66, 127.61, 127.50, 127.46, 127.42, 127.32, 127.23, 127.11, 113.82, 101.61, 100.61, 98.19, 82.57, 80.75, 79.66, 77.60, 77.24, 75.42, 75.24, 74.92, 74.86, 74.75, 74.45, 74.28, 73.56, 73.41, 72.40, 71.52, 71.47, 70.90, 68.70, 66.12, 62.80, 55.28, 29.70, 18.05, 18.01, 12.04; MALDI-TOF: $[M + Na]^+$ calcd for $C_{91}H_{107}N_3NaO_{16}Si^+$, 1549.94; found, 1549.44.

Benzyl 2,3,4-tri-*O*-benzyl-6-*O*-triisopropylsilyl-α-D-mannopyranosyl-(1→3)-2-*O*-benzyl-4,6-*O*-benzylidene-β-D-mannopyranosyl-(1→4)-2-azido-3,6-di-*O*-benzyl-2-deoxy-β-D-glucopyranoside (26)

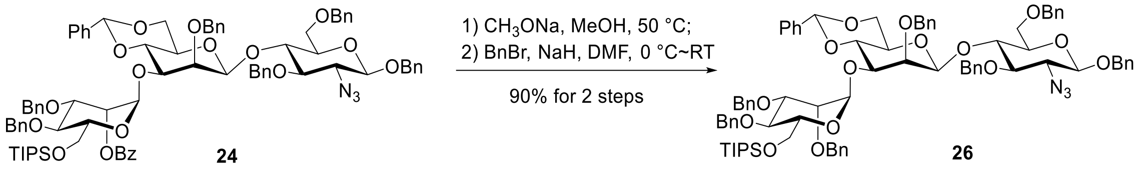

To a solution of compound **24** (90 mg, 0.0635 mmol) in MeOH (4.0 mL) was added sodium methoxide until pH = 10, the solution was heated to 50 °C and stirred overnight. After the complete disappearance of the starting material, the solution was concentrated to dryness and dissolved in dry *N*,*N*-dimethylformamide (3.0 mL) and cooled to 0 °C, sodium hydride (10.2 mg) and benzyl bromide (22.7 µL) were added successively, and the mixture was slowly warmed to room temperature. After the completion of the reaction as monitored by TLC, MeOH was added to quench the excess sodium hydride. The reaction was diluted with CH_2Cl_2, successively washed with H_2O and brine and dried over anhydrous Na_2SO_4. The residue was purified by flash column chromatography (hexanes/EtOAc = 10:1~6:1) to afford compound **26** (80.0 mg, 90% for 2 steps) as colorless syrup. R_f = 0.20 (hexanes/EtOAc = 6:1); ^{1}H NMR (400 MHz, $CDCl_3$) δ 7.48-7.46, 7.43-7.15, 7.10-7.08 (40H, m, Ar-H), 5.48 (1H, s), 5.34 (1H, m), 5.08 (1H, d, J = 10.5 Hz, PhCH_2), 4.99-4.94 (2H, m), 4.78-4.61 (7H, m), 4.54-4.48 (3H, m), 4.43 (1H, d, J = 12.4 Hz), 4.35-4.31 (2H, m), 4.07-3.98 (3H, m), 3.97-3.88 (4H, m), 3.85-3.82 (2H, m), 3.79-3.78 (1H, m), 3.73-3.68 (2H, m), 3.64-3.61 (1H, m), 3.53-3.46 (2H, m), 3.40-3.32 (2H, m), 3.10-3.03 (1H, m), 1.08-1.07 (21H, s); ^{13}C NMR (100 MHz, $CDCl_3$) δ 138.92, 138.58, 138.56, 138.37, 138.34, 137.76, 137.49, 136.85, 129.22, 128.57, 128.48, 128.29, 128.27, 128.21, 128.13, 128.02, 127.97, 127.94, 127.92, 127.79, 127.72, 127.60, 127.47, 127.44, 127.38, 127.20, 126.16, 101.75, 100.90, 100.46, 98.47, 81.64, 79.72, 79.07, 78.87, 77.22, 76.58, 75.69, 75.63, 75.03, 74.97, 74.81, 74.61, 74.38, 73.60, 71.82, 70.86, 68.49, 68.33, 66.89, 65.78, 63.46, 29.71, 18.09, 18.07, 12.05; MALDI-TOF: $[M + Na]^+$ calcd for $C_{83}H_{97}N_3NaO_{15}Si^+$, 1426.66; found, 1426.03.

Benzyl 2,3,4-tri-*O*-benzyl-6-*O*-triisopropylsilyl-α-D-mannopyranosyl-(1→6)-2,4-di-*O*-benzyl-3-*O*-*p*-methoxybenzyl-β-D-mannopyranosyl-(1→4)-2-acetamido-3,6-di-*O*-benzyl-2-deoxy-β-D-glucopyranoside (27)

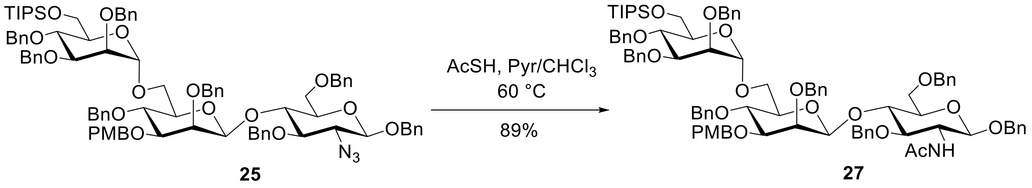

A solution of compound **25** (65.0 mg, 0.042 mmol) in a mixture of AcSH/pyridine/$CHCl_3$ (0.6 mL/0.4 mL/0.6 mL) was stirred at 60 °C for 18 h. After the completion of the reaction as monitored by TLC, the resulting mixture was concentrated and subjected to flash chromatography on silica gel (hexanes/EtOAc = 8:1~3:1) to afford compound **27** (58.1 mg, 89%) as colorless syrup. R_f = 0.30 (hexanes/EtOAc = 3:1); ^{1}H NMR (400 MHz, $CDCl_3$) δ 7.41-7.18 (42H, m, Ar-H), 6.86-6.84 (2H, m, Ar-H), 5.33 (1H, d, J = 7.9 Hz, NH), 4.95-4.83 (8H, m, PhCH_2), 4.63-4.53 (7H, m), 4.50-4.41 (6H, m), 4.06 (1H, dd, J = 9.5 Hz, J = 9.5 Hz), 4.03-3.97 (2H, m), 3.88-3.73 (11H, m), 3.66-3.63 (1H, m), 3.58-3.52 (4H, m), 3.44 (1H, dd, J = 2.8 Hz, J = 9.3 Hz), 3.33-3.29 (1H, m), 1.51 (3H, s), 1.04 (21H, s); ^{13}C NMR (100 MHz, $CDCl_3$) δ 169.48, 158.75, 138.81, 138.64, 138.44, 138.19, 138.00, 137.63, 137.39, 129.76, 128.69, 127.94, 127.78, 127.75, 127.71, 127.67, 127.58, 127.55, 127.47, 127.28, 127.19, 127.16, 127.07, 127.02, 126.95, 126.80, 126.77, 126.59, 113.31, 100.63, 98.84, 97.51, 82.08, 79.65,

75.19, 74.80, 74.60, 74.54, 74.45, 74.36, 74.29, 74.04, 73.98, 73.11, 73.02, 72.72, 71.91, 71.32, 70.99, 70.23, 68.98, 65.87, 62.09, 54.77, 29.19, 22.69, 17.53, 17.49, 11.56; MALDI-TOF: $[M + Na]^+$ calcd for $C_{93}H_{111}NNaO_{17}Si^+$, 1565.98; found, 1565.46.

Benzyl 2,3,4-tri-*O*-benzyl-6-*O*-triisopropylsilyl-α-D-mannopyranosyl-(1→3)-2-*O*-benzyl-4,6-*O*-benzylidene-β-D-mannopyranosyl-(1→4)-2-acetamido-3,6-di-*O*-benzyl-2-deoxy-β-D-glucopyranoside (28)

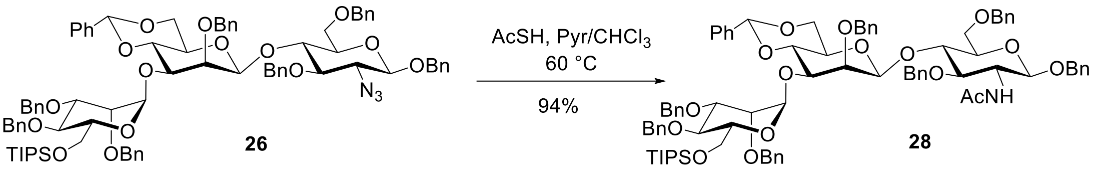

A solution of compound **26** (80.0 mg, 0.057 mmol) in a mixture of AcSH/pyridine/$CHCl_3$ (0.6 mL/0.4 mL/0.6 mL) was stirred at 60 °C for 18 h. After the completion of the reaction as monitored by TLC, the resulting mixture was concentrated and subjected to flash chromatography on silica gel (hexanes/EtOAc = 4:1~2:1) to afford compound **27** (76.5 mg, 94%) as colorless syrup. R_f = 0.25 (hexanes/EtOAc = 2:1); ^1H NMR (400 MHz, $CDCl_3$) δ 7.50-7.48, 7.38-7.16, 7.11-7.09 (40H, m, Ar-H), 5.97 (1H, d, *J* = 8.5 Hz, N*H*), 5.51 (1H, s), 5.36 (1H, m), 4.98-4.91 (3H, m), 4.81-4.77 (2H, m), 4.72 (1H, d, *J* = 11.3 Hz, PhCH_2), 4.69-4.62 (3H, m), 4.60-4.57 (2H, m), 4.53-4.47 (4H, m), 4.37 (1H, d, *J* = 12.4 Hz), 4.11-4.07 (1H, m), 4.06-3.82 (12H, m), 3.79-3.68 (3H, m), 3.59 (1H, dd, *J* = 10.2 Hz, *J* = 10.2 Hz), 3.19-3.13 (1H, m), 1.69 (3H, s), 1.08 (21H, s); ^{13}C NMR (100 MHz, $CDCl_3$) δ 170.04, 138.83, 138.67, 138.50, 138.28, 138.02, 137.64, 137.40, 129.27, 128.46, 128.35, 128.32, 128.28, 128.21, 128.07, 127.93, 127.86, 127.83, 127.80, 127.67, 127.59, 127.52, 127.49, 127.47, 127.44, 127.26, 126.17, 101.82, 101.52, 99.26, 98.47, 79.71, 79.00, 78.91, 77.22, 75.93, 75.58, 75.28, 75.02, 74.69, 74.59, 74.49, 73.46, 72.94, 71.81, 70.54, 69.56, 68.58, 66.90, 63.44, 53.16, 23.13, 18.09, 18.07, 12.03; MALDI-TOF: $[M + Na]^+$ calcd for $C_{85}H_{101}NNaO_{16}Si^+$, 1442.68; found, 1442.29.

Benzyl 2,3,4-tri-*O*-benzyl-α-D-mannopyranosyl-(1→6)-2,4-di-*O*-benzyl-3-*O*-*p*-methoxybenzyl-β-D-mannopyranosyl-(1→4)-2-acetamido-3,6-di-*O*-benzyl-2-deoxy-β-D-glucopyranoside (29)

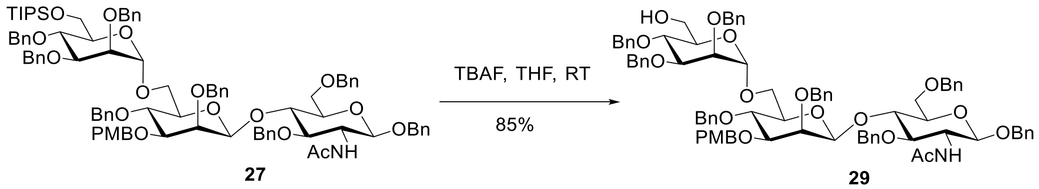

To a solution of compound **27** (65.0 mg, 0.042 mmol) in THF (2.0 mL) was added TBAF (1 M in THF, 210 µL), and the mixture was stirred at room temperature for 20 h. After the completion of the reaction as monitored by TLC, the resulting mixture was concentrated and subjected to flash chromatography on silica gel (hexanes/EtOAc = 5:1~1:2) to afford compound **29** (49.0 mg, 85%) as colorless syrup. R_f = 0.20 (hexanes/EtOAc = 1:1); ^1H NMR (400 MHz, $CDCl_3$) δ 7.43-7.41, 7.36-7.21 (42H, m, Ar-H), 6.89-6.87 (2H, m, Ar-H), 5.41 (1H, d, *J* = 8.0 Hz, N*H*), 5.07 (1H, m), 4.99-4.85 (7H, m, PhCH_2), 4.64-4.44 (12H, m), 4.17 (1H, dd, *J* = 7.4 Hz, *J* = 7.4 Hz), 4.08 (1H, dd, *J* = 7.2 Hz, *J* = 7.2 Hz), 3.96 (1H, dd, *J* = 9.4 Hz, *J* = 9.4 Hz), 3.89-3.80 (6H, m), 3.77-3.55 (9H, m), 3.47 (1H, dd, *J* = 2.9 Hz, *J* = 9.2 Hz), 3.39-3.35 (1H, m), 2.59 (1H, m), 1.56 (3H, s); ^{13}C NMR (100 MHz, $CDCl_3$) δ 170.46, 159.30, 139.14, 138.79, 138.61, 138.49, 138.36, 138.00, 137.84, 130.17, 129.24, 128.47, 128.40, 128.35,

128.31, 128.29, 128.27, 128.24, 128.11, 127.85, 127.84, 127.79, 127.76, 127.72, 127.65, 127.61, 127.56, 127.53, 127.40, 127.34, 113.86, 101.18, 99.24, 97.88, 82.41, 79.89, 75.59, 75.30, 75.19, 75.05, 74.99, 74.76, 74.69, 73.62, 73.56, 72.73, 72.33, 71.86, 71.49, 70.74, 69.46, 67.01, 61.99, 55.30, 23.21; MALDI-TOF: $[M + Na]^+$ calcd for $C_{84}H_{91}NNaO_{17}^+$, 1408.62; found, 1408.27.

Benzyl 2,3,4-tri-*O*-benzyl-α-D-mannopyranosyl-(1→3)-2-*O*-benzyl-4,6-*O*-benzylidene-β-D-mannopyranosyl-(1→4)-2-acetamido-3,6-di-*O*-benzyl-2-deoxy-β-D-glucopyranoside (30)

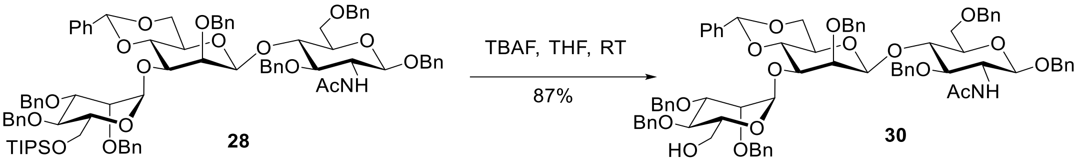

To a solution of compound **28** (76.5 mg, 0.054 mmol) in THF (2.0 mL) was added TBAF (1 M in THF, 269 µL), and the mixture was stirred at room temperature for 20 h. After the completion of the reaction as monitored by TLC, the resulting mixture was concentrated and subjected to flash chromatography on silica gel (hexanes/EtOAc = 3:1~2:3) to afford compound **30** (59.3 mg, 87%) as colorless syrup. R_f = 0.20 (hexanes/EtOAc = 1:1); ^{1}H NMR (400 MHz, $CDCl_3$) δ 7.52-7.49, 7.41-7.16, 7.10-7.08 (40H, m, Ar-H), 5.78 (1H, d, *J* = 8.0 Hz, N*H*), 5.52 (1H, s), 5.35 (1H, m), 5.01-4.91 (4H, m), 4.81-4.77 (2H, m), 4.78 (2H, m), 4.69-4.60 (5H, m), 4.53-4.47 (4H, m), 4.44-4.40 (1H, m), 4.17-3.95 (5H, m), 3.90-3.74 (7H, m), 3.71-3.58 (5H, m), 3.22-3.16 (1H, m), 1.77 (3H, s); ^{13}C NMR (100 MHz, $CDCl_3$) δ 170.19, 138.86, 138.54, 138.34, 138.06, 137.97, 137.58, 137.45, 129.35, 128.55, 128.38, 128.34, 128.21, 128.19, 127.98, 127.94, 127.88, 127.82, 127.77, 127.71, 127.57, 127.54, 127.50, 126.19, 101.89, 101.55, 99.19, 98.81, 79.64, 78.94, 78.56, 77.86, 75.64, 75.46, 75.15, 74.78, 74.43, 73.70, 73.57, 72.92, 72.16, 71.89, 70.77, 69.11, 68.61, 66.98, 62.52, 55.15, 23.32; MALDI-TOF: $[M + Na]^+$ calcd for $C_{76}H_{81}NNaO_{16}^+$, 1286.54; found, 1286.20.

Benzyl 2,3,4-tri-*O*-benzyl-6-*O*-dibenzylphosphonato-α-D-mannopyranosyl-(1→6)-2,4-di-*O*-benzyl-3-*O*-*p*-methoxybenzyl-β-D-mannopyranosyl-(1→4)-2-acetamido-3,6-di-*O*-benzyl-2-deoxy-β-D-glucopyranoside (31)

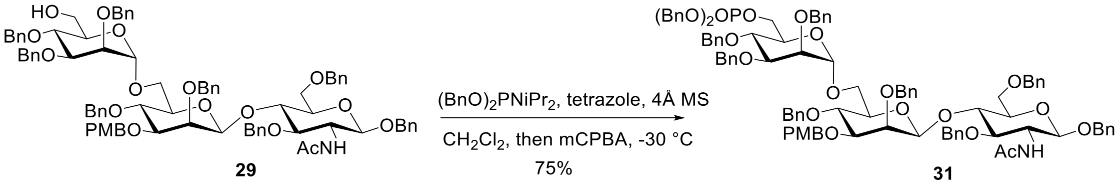

To a solution of compound **29** (38.0 mg, 0.027 mmol) in anhydrous CH_2Cl_2 (1.0 mL) was added activated 4Å molecular sieves (100 mg) and tetrazole (0.45 M in MeCN, 305 µL) and the mixture was stirred at room temperature for 1.5 h before $(BnO)_2PNiPr_2$ (37.4 µL) was added. The resulting mixture was further stirred overnight under argon atmosphere at room temperature until the complete disappearance of the starting material. Then the reaction was cooled to -30 °C, and mCPBA (77 wt %, 32.5 mg) was added, the reaction mixture was stirred at this temperature for 1 h and then filtered through a Celite pad. The filtrate was diluted with CH_2Cl_2, washed with saturated $NaHCO_3$ (aq.), dried over Na_2SO_4 and concentrated to dryness. The residue was purified by flash chromatography (hexanes/EtOAc = 4:1~2:3) to give compound **31** (33.9 mg, 75%) as colorless syrup. R_f = 0.20 (hexanes/EtOAc = 1:1); ^{1}H NMR (400 MHz, $CDCl_3$) δ 7.40-7.38, 7.32-7.15 (52H, m, Ar-H), 6.86-6.84 (2H, m, Ar-H), 5.30 (1H, d, *J* = 7.5 Hz, N*H*), 5.06-4.88 (8H, m), 4.85-4.82 (3H, m), 4.64-4.36

(12H, m), 4.11 (1H, dd, J = 8.2 Hz, J = 8.2 Hz), 4.05-3.96 (3H, m), 3.86-3.70 (11H, m), 3.62-3.56 (2H, m), 3.45 (1H, dd, J = 2.9 Hz, J = 9.3 Hz), 3.40-3.33 (2H, m), 1.53 (3H, s); ^{13}C NMR (100 MHz, $CDCl_3$) δ 170.27, 159.24, 139.14, 138.66, 138.62, 138.46, 138.40, 138.17, 137.92, 136.19, 136.12, 136.07, 136.00, 130.22, 129.20, 128.45, 128.43, 128.37, 128.34, 128.27, 128.24, 128.20, 128.18, 128.14, 128.02, 127.90, 127.81, 127.70, 127.65, 127.61, 127.51, 127.47, 127.39, 113.81, 100.75, 99.30, 97.91, 82.53, 80.11, 77.77, 77.25, 77.15, 75.36, 75.21, 75.12, 75.01, 74.93, 74.83, 74.74, 74.53, 74.37, 73.97, 73.55, 72.57, 71.92, 71.42, 70.89, 70.77, 69.38, 69.18, 69.13, 69.07, 69.01, 66.74, 66.46, 56.21, 55.29, 29.72, 23.27; ^{31}P NMR (146 MHz, $CDCl_3$) δ -1.24; MALDI-TOF: [M + Na]$^+$ calcd for $C_{98}H_{104}NNaO_{20}P^+$, 1668.68; found, 1669.05.

Benzyl 2,3,4-tri-*O*-benzyl-6-*O*-dibenzylphosphonato-α-D-mannopyranosyl-(1→3)-2-*O*-benzyl-4,6-*O*-benzylidene-β-D-mannopyranosyl-(1→4)-2-acetamido-3,6-di-*O*-benzyl-2-deoxy-β-D-glucopyranoside (32)

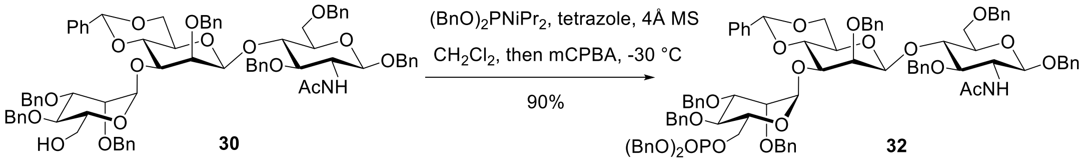

To a solution of compound **30** (58.0 mg, 0.046 mmol) in anhydrous CH_2Cl_2 (2.0 mL) was added activated 4Å molecular sieves (200 mg) and tetrazole (0.45 M in MeCN, 511 µL) and the mixture was stirred at room temperature for 1.5 h before $(BnO)_2PNiPr_2$ (62.5 µL) was added. The resulting mixture was further stirred overnight under argon atmosphere at room temperature until the complete disappearance of the starting material. Then the reaction was cooled to -30 °C, and mCPBA (77 wt %, 54.4 mg) was added, the reaction mixture was stirred at this temperature for 1 h and then filtered through a Celite pad. The filtrate was diluted with CH_2Cl_2, washed with saturated $NaHCO_3$ (aq.), dried over Na_2SO_4 and concentrated to dryness. The residue was purified by flash chromatography (hexanes/EtOAc = 4:1~2:3) to give compound **32** (63.2 mg, 90%) as colorless syrup. R_f = 0.20 (hexanes/EtOAc = 1:1); ^{1}H NMR (400 MHz, $CDCl_3$) δ 7.49-7.46, 7.40-7.17, 7.14-7.10, 7.04-7.02 (50H, m, Ar-H), 5.83 (1H, d, J = 8.1 Hz, N*H*), 5.49 (1H, s), 5.32 (1H, m), 5.10-5.00 (4H, m), 4.99-4.87 (4H, m), 4.76-4.72 (2H, m), 4.65-4.58 (5H, m), 4.51-4.45 (3H, m), 4.43-4.40 (1H, m), 4.37-4.42 (3H, m), 4.16-4.04 (2H, m), 4.01-3.93 (3H, m), 3.87-3.73 (6H, m), 3.70-3.64 (2H, m), 3.57 (1H, dd, J = 10.3 Hz, J = 10.3 Hz), 3.11-3.04 (1H, m), 1.76 (3H, s); ^{13}C NMR (100 MHz, $CDCl_3$) δ 170.20, 138.83, 138.43, 138.27, 138.09, 137.98, 137.62, 137.49, 135.96, 135.90, 129.31, 128.57, 128.53, 128.50, 128.46, 128.42, 128.35, 128.33, 128.20, 128.15, 128.03, 127.94, 127.91, 127.87, 127.78, 127.73, 127.65, 127.53, 127.47, 127.42, 126.20, 101.92, 101.24, 99.18, 98.64, 79.59, 78.94, 78.59, 75.64, 75.57, 75.25, 75.05, 74.32, 74.05, 73.51, 73.43, 71.98, 71.78, 70.68, 69.41, 69.35, 69.24, 69.19, 68.61, 66.74, 54.76, 23.29; ^{31}P NMR (146 MHz, $CDCl_3$) δ -1.08; MALDI-TOF: [M + Na]$^+$ calcd for $C_{90}H_{94}NNaO_{19}P^+$, 1546.60; found, 1546.01.

6-*O*-phosphonato-α-D-mannopyranosyl-(1→6)-β-D-mannopyranosyl-(1→4)-2-acetamido-2-deoxy-αβ-D-glucopyranoside (33)

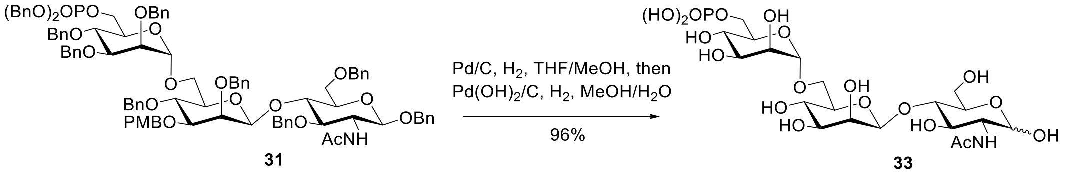

A mixture of compound **31** (60.2 mg, 0.036 mmol) and Pd/C (10 wt.% loading, 30 mg) in MeOH (2.0 mL) and THF (2.0 mL) was stirred under H_2 atmosphere for 21 h. The reaction mixture was filtered through a Celite pad, then concentrated to dryness. The mixture of the residue and $Pd(OH)_2/C$ (20 wt.% loading, 40 mg) in MeOH (2.5 mL) and H_2O (2.5 mL) was stirred under H_2 atmosphere for further 21 h. The reaction mixture was filtered through a Celite pad. The filtrate was concentrated to dryness then dissolved in H_2O and lyophilized. The crude product was purified on a Sephadex G-10 column by elution with H_2O. Fractions containing the product were pooled and lyophilized to give compound **33** (22.0 mg, 96%) as white solid. R_f = 0.40 (n-BuOH/EtOH/H_2O/AcOH = 1:1:1:0.05); ^{1}H NMR (400 MHz, D_2O) δ 5.14 (0.63H, d, J = 3.2 Hz), 4.84 (1.03H, m), 4.65 (0.39H, m), 4.03-3.98 (3.24H, m), 3.90-3.79 (5.69H, m), 3.75-3.62 (6.60H, m), 3.61-3.53 (4.03H, m), 1.99 (3H, s); ^{13}C NMR (100 MHz, D_2O) δ 174.92, 174.62, 100.55, 100.51, 99.88, 99.84, 94.91, 90.48, 80.28, 79.95, 74.45, 74.30, 72.78, 72.30, 71.88, 71.80, 70.51, 70.46, 70.19, 70.13, 69.93, 69.89, 69.08, 66.70, 66.27, 66.22, 63.59, 60.29, 60.17, 56.01, 53.61, 22.30, 21.98; ^{31}P NMR (146 MHz, D_2O) δ 1.91 (overlapped signals); HRMS: $[M + H]^+$ calcd for $C20H37NO19P^+$, 626.1692; found, 626.1690.

6-*O*-phosphonato-α-D-mannopyranosyl-(1→3)-β-D-mannopyranosyl-(1→4)-2-acetamido-2-deoxy-αβ-D-glucopyranoside (34)

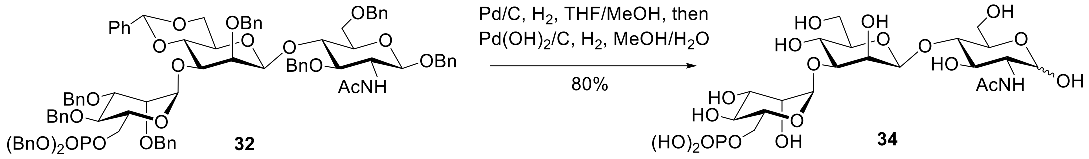

A mixture of compound **32** (53.0 mg, 0.034 mmol) and Pd/C (10 wt.% loading, 30 mg) in MeOH (2.0 mL) and THF (2.0 mL) was stirred under H_2 atmosphere for 21 h. The reaction mixture was filtered through a Celite pad, then concentrated to dryness. The mixture of the residue and $Pd(OH)_2/C$ (20 wt.% loading, 30 mg) in MeOH (2.5 mL) and H_2O (2.5 mL) was stirred under H_2 atmosphere for further 21 h. The reaction mixture was filtered through a Celite pad. The filtrate was concentrated to dryness then dissolved in H_2O and lyophilized. The crude product was purified on a Sephadex G-10 column by elution with H_2O. Fractions containing the product were pooled and lyophilized to give compound **34** (17.4 mg, 80%) as white solid. R_f = 0.35 (n-BuOH/EtOH/H_2O/AcOH = 1:1:1:0.05); ^{1}H NMR (400 MHz, D_2O) δ 5.14 (0.60H, d, J = 3.4 Hz), 5.06-5.05 (0.98H, m), 4.66-4.64 (0.51H, m), 4.15 (1.06H, m), 4.07-3.96 (3.20H, m), 3.89-3.80 (5.62H, m), 3.76-3.59 (7.68H, m), 3.53-3.49 (0.45H, m), 3.42-3.39 (0.96H, m), 1.98 (3H, s); ^{13}C NMR (100 MHz, D_2O) δ 174.31, 174.02, 102.02, 101.98, 99.44, 94.51, 90.11, 80.15, 80.01, 78.87, 78.49, 75.66, 74.29, 72.05, 71.87, 69.85, 69.80, 69.74, 69.60, 68.70, 66.01, 65.59, 65.53, 63.59, 60.47, 59.87, 59.76, 55.71, 53.26, 21.78, 21.49; ^{31}P NMR (146 MHz, D_2O) δ 1.71 (overlapped signals); HRMS: $[M + H]^+$ calcd for $C20H37NO19P^+$, 626.1692; found, 626.1690.

2-Methyl-[6-*O*-phosphonato-α-D-mannopyranosyl-(1→6)-β-D-mannopyranosyl-(1→4)-1,2-dideoxy-α-D-glucopyrano]-[2,1-*d*]-2-oxazoline (6)

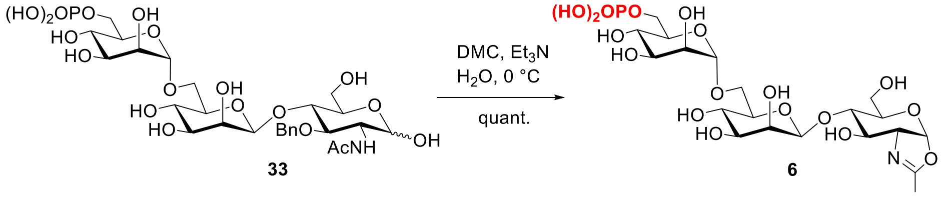

To a solution of compound **33** (8.0 mg, 0.013 mmol) in H_2O (300 μL) were added Et_3N (72.0 μL) and

2-chloro-1,3-dimethylimidazolinium chloride (DMC, 43.4 mg) at 0 °C. The reaction mixture was monitored by DIONEX HPAEC-PAD. After 2 h, the HPAEC analysis indicated that the free oligosaccharide was converted into a new oligosaccharide that was eluted earlier than the reducing sugar under the HPAEC condition (see general method). The product was purified by gel filtration on a Sephadex G-10 column that was eluted with 0.1% aq Et_3N to afford compound **6** (7.8 mg, quant.) as white solid after lyophilization with 5 mol.% of NaOH. ^{1}H NMR (400 MHz, D_2O) δ 6.02 (1H, d, J = 7.3 Hz), 4.87 (1H, m), 4.84-4.81 (1H, m), 4.64-4.62 (1H, m), 4.31-4.30 (1H, m), 4.15-4.12 (1H, m), 4.01-3.95 (2H, m), 3.92-3.86 (4H, m), 3.81-3.78 (2H, m), 3.80-3.76 (1H, m), 3.74-3.73 (1H, m), 3.71-3.70 (1H, m), 3.68-3.65 (3H, m), 3.61-3.54 (3H, m), 3.48-3.44 (1H, m), 3.36-3.34 (1H, m), 1.99 (3H, d, J = 1.8 Hz); ^{13}C NMR (100 MHz, D_2O) δ 168.55, 101.49, 99.90, 99.83, 77.63, 74.51, 72.96, 72.25, 70.92, 70.48, 70.18, 70.00, 69.06, 66.48, 66.03, 65.81, 65.16, 62.59, 61.77, 12.96; ^{31}P NMR (146 MHz, D_2O) δ 4.45; HRMS: $[M + H]^+$ calcd for C20H35NO18P$^+$, 608.1586; found, 608.1598.

2-Methyl-[6-*O*-phosphonato-α-D-mannopyranosyl-(1→3)-β-D-mannopyranosyl-(1→4)-1,2-dideoxy-α-D-glucopyrano]-[2,1-*d*]-2-oxazoline (7)

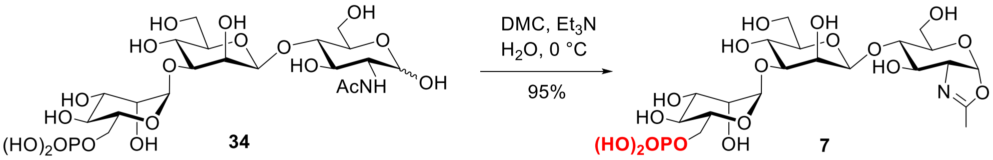

To a solution of compound **34** (8.0 mg, 0.013 mmol) in H_2O (300 µL) were added Et_3N (72.0 µL) and 2-chloro-1,3-dimethylimidazolinium chloride (DMC, 43.4 mg) at 0 °C. The reaction mixture was monitored by DIONEX HPAEC-PAD. After 2 h, the HPAEC analysis indicated that the free oligosaccharide was converted into a new oligosaccharide that was eluted earlier than the reducing sugar under the HPAEC condition (see general method). The product was purified by gel filtration on a Sephadex G-10 column that was eluted with 0.1% aq Et_3N to afford compound **7** (7.4 mg, 95%) as white solid after lyophilization with 5 mol.% of NaOH. ^{1}H NMR (400 MHz, D_2O) δ 6.00 (1H, d, J = 7.3 Hz), 5.02 (1H, m), 4.30-4.28 (1H, m), 4.11-4.08 (1H, m), 4.03-4.02 (1H, m), 3.98-3.94 (2H, m), 3.88-3.81 (4H, m), 3.78-3.76 (1H, m), 3.74-3.70 (2H, m), 3.68-3.64 (4H, m), 3.60-3.55 (2H, m), 3.37-3.33 (2H, m), 1.99 (3H, d, J = 1.5 Hz); ^{13}C NMR (100 MHz, D_2O) δ 168.61, 102.51, 101.10, 99.94, 80.35, 77.67, 76.12, 72.82, 72.74, 70.97, 70.13, 70.00, 69.34, 66.19, 66.06, 65.20, 62.65, 62.61, 61.56, 61.04, 13.00; ^{31}P NMR (146 MHz, D_2O) δ 4.45; HRMS: $[M + H]^+$ calcd for C20H35NO18P$^+$, 608.1586; found, 608.1599.

Phenyl 3,4-di-*O*-benzyl-2-*O*-benzoyl-6-*O*-triisopropylsilyl-α-D-mannopyranosyl-(1→2)-3,4,6-tri-*O*-benzyl-1-thio-α-D-mannopyranoside (37)

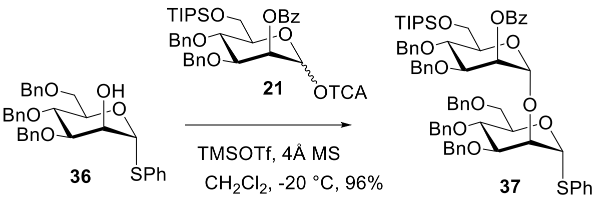

A mixture of trichloroacetimidate donor **21** [4] (895 mg, 1.17 mmol), acceptor **36** [5] (489 mg, 0.902 mmol) and activated 4Å molecular sieves (1.0 g) in anhydrous CH_2Cl_2 (10 mL) was stirred at room temperature under argon atmosphere for 1.5 h, and then cooled to -20 °C. TMSOTf (16.0 µL, 0.09 mmol) was added. After stirring at -20 °C for 0.5 h, the mixture was quenched with triethylamine (20

µL) and filtered then concentrated in vacuo. The residue was purified via silica gel chromatography (hexanes/EtOAc = 15:1~10:1) to give product **37** (995 mg, 96%) as colorless syrup. R_f = 0.60 (hexanes/EtOAc = 5:1); ^{1}H NMR (400 MHz, $CDCl_3$) δ 8.20-8.17, 7.67-7.63, 7.54-7.51, 7.45, 7.41-7.26 (35H, m, Ar-*H*), 5.82 (1H, m), 5.65 (1H, d, *J* = 1.5 Hz), 5.29 (1H, d, *J* = 1.6 Hz), 4.99-4.93, 4.84-4.78, 4.75-4.65, 4.57-4.53 (10H, m, PhC*H*$_2$), 4.37-4.34 (2H, m), 4.21-4.11 (3H, m), 4.05-3.98 (2H, m), 3.92-3.79 (4H, m), 1.15-1.11 (21H, m); ^{13}C NMR (100 MHz, $CDCl_3$) δ 165.63, 138.81, 138.52, 138.46, 138.27, 138.00, 134.44, 133.11, 131.52, 130.16, 130.07, 129.04, 128.54, 128.43, 128.34, 128.32, 128.26, 128.10, 128.09, 128.00, 127.95, 127.83, 127.72, 127.59, 127.56, 127.51, 127.46, 127.41, 99.61, 87.42, 80.25, 78.38, 77.31, 75.92, 75.31, 74.81, 74.04, 73.46, 73.27, 73.04, 72.30, 71.89, 69.39, 69.27, 62.39, 18.13, 18.08, 12.07; MALDI-TOF: $[M + Na]^+$ calcd for $C_{69}H_{80}NaO_{11}SSi^+$, 1167.51; found, 1167.73.

Phenyl 2,3,4-tri-*O*-benzyl-6-*O*-triisopropylsilyl-α-D-mannopyranosyl-(1→2)-3,4,6-tri-*O*-benzyl-1-thio-α-D-mannopyranoside (38)

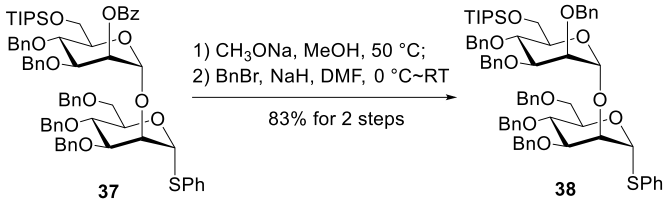

To a solution of compound **37** (1.30 g, 1.136 mmol) in MeOH (12.0 mL) was added sodium methoxide until pH = 10, the solution was heated to 50 °C and stirred overnight. After the complete disappearance of the starting material, the solution was concentrated to dryness and dissolved in dry *N*,*N*-dimethylformamide (10.0 mL) and cooled to 0 °C, sodium hydride (78.4 mg) and benzyl bromide (225 µL) were added successively, and the mixture was slowly warmed to room temperature. After the completion of the reaction as monitored by TLC, MeOH was added to quench the excess sodium hydride. The reaction was diluted with CH_2Cl_2, successively washed with H_2O and brine and dried over anhydrous Na_2SO_4. The residue was purified by flash column chromatography (hexanes/EtOAc = 15:1~10:1) to afford compound **38** (1.059 g, 83% for 2 steps) as colorless syrup. R_f = 0.60 (hexanes/EtOAc = 8:1); ^{1}H NMR (400 MHz, $CDCl_3$) δ 7.51-7.48, 7.39-7.27 (35H, m, Ar-*H*), 5.60 (1H, d, *J* = 1.4 Hz), 5.31 (1H, d, *J* = 2.3 Hz), 4.96-4.90 (2H, m), 4.74-4.69 (3H, m), 4.67 (1H, m), 4.64-4.58 (2H, m), 4.58-4.49 (5H, m), 4.39 (1H, m), 4.32 (1H, m), 4.05 (1H, dd, *J* = 9.4 Hz, *J* = 9.4 Hz), 4.00-3.82 (7H, m), 3.77-3.71 (2H, m), 1.15-1.13 (21H, m); ^{13}C NMR (100 MHz, $CDCl_3$) δ 138.84, 138.78, 138.54, 138.51, 138.42, 137.91, 134.42, 131.20, 128.54, 128.98, 128.56, 128.44, 128.38, 128.33, 128.29, 128.27, 128.15, 128.12, 128.08, 128.03, 128.00, 127.98, 127.78, 127.73, 127.54, 127.42, 127.39, 127.22, 98.91, 87.31, 80.76, 79.81, 77.28, 75.25, 75.11, 75.06, 74.97, 74.76, 74.35, 74.10, 73.29, 72.94, 72.45, 72.30, 72.04, 69.28, 63.01, 18.09, 18.05, 12.06; MALDI-TOF: $[M + Na]^+$ calcd for $C_{69}H_{82}NaO_{10}SSi^+$, 1153.53; found, 1152.91.

Benzyl 2,3,4-tri-*O*-benzyl-6-*O*-triisopropylsilyl-α-D-mannopyranosyl-(1→2)-3,4,6-tri-*O*-benzyl-α-D-mannopyranosyl-(1→3)-2-*O*-benzyl-4,6-*O*-benzylidene-β-D-mannopyranosyl-(1→4)-2-azido-3,6-di-*O*-benzyl-2-deoxy-β-D-glucopyranoside (39)

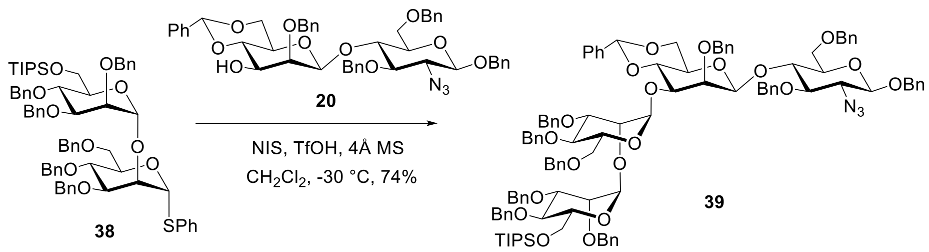

A mixture of compound **38** (180 mg, 0.159 mmol), acceptor **20** (100 mg, 0.123 mmol) and activated 4Å molecular sieves (450 mg) in anhydrous CH_2Cl_2 (4.5 mL) was stirred at room temperature under argon atmosphere for 1.5 h, and then cooled to -30 °C. N-iodosuccinimide (55.2 mg, 0.245 mmol) and TfOH (2.15 μL, 0.025 mmol) were successively added. After stirring at -30 °C for 2 h, the mixture was quenched with triethylamine (10 μL) and filtered then concentrated in vacuo. The residue was purified via silica gel chromatography (hexanes/EtOAc = 10:1~5:1) to give product **39** (166 mg, 74%) as colorless syrup. R_f = 0.50 (hexanes/EtOAc = 4:1); Spectroscopic data were in agreement with literature values. [7] MALDI-TOF: $[M + H]^+$ calcd for $C_{110}H_{126}N_3O_{20}Si^+$, 1836.87; found, 1836.44.

Benzyl 2,3,4-tri-*O*-benzyl-6-*O*-triisopropylsilyl-α-D-mannopyranosyl-(1→2)-3,4,6-tri-*O*-benzyl-α-D-mannopyranosyl-(1→3)-2-*O*-benzyl-4,6-*O*-benzylidene-β-D-mannopyranosyl-(1→4)-2-acetamido-3,6-di-*O*-benzyl-2-deoxy-β-D-glucopyranoside (40)

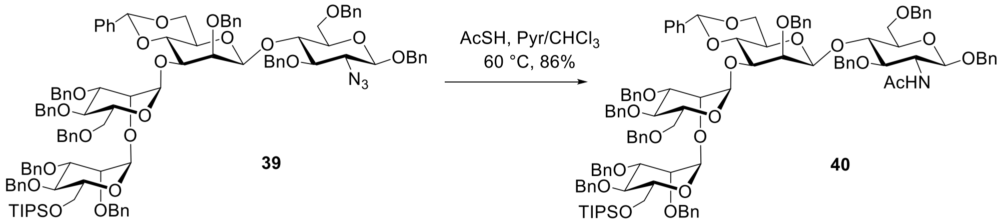

A solution of compound **39** (133.5 mg, 0.073 mmol) in a mixture of AcSH/pyridine/$CHCl_3$ (0.6 mL/0.4 mL/0.6 mL) was stirred at 60 °C for 18 h. After the completion of the reaction as monitored by TLC, the resulting mixture was concentrated and subjected to flash chromatography on silica gel (hexanes/EtOAc = 4:1~1:1) to afford compound **40** (115.8 mg, 86%) as colorless syrup. R_f = 0.30 (hexanes/EtOAc = 2:1); ^{1}H NMR (400 MHz, $CDCl_3$) δ 7.42-7.23 (54H, m), 7.03 (1H, t, J = 7.7 Hz), 5.86 (1H, d, J = 8.2 Hz), 5.51 (1H, s), 5.40 (1H, m), 5.29 (1H, m), 4.97-4.88 (4H, m), 4.86 (1H, d, J = 4.2 Hz), 4.83 (2H, m), 4.68-4.38 (15H, m), 4.31 (1H, m), 4.19-4.01 (5H, m), 3.98-3.85 (5H, m), 3.82 (1H, m), 3.79-3.53 (10H, m), 3.50 (1H, m), 3.09 (1H, m), 1.75 (3H, s), 1.32-1.28 (3H, m), 1.08 (18H, s); ^{13}C NMR (100 MHz, $CDCl_3$) δ 170.17, 139.18, 138.89, 138.78, 138.49, 138.46, 138.44, 138.20, 137.97, 137.89, 137.63, 137.16, 128.51, 128.47, 128.40, 128.37, 128.33, 128.28, 128.22, 128.16, 128.10, 128.05, 127.94, 127.90, 127.78, 127.74, 127.70, 127.57, 127.52, 127.34, 127.28, 125.75, 101.52, 101.40, 99.82, 99.20, 97.58, 79.74, 78.92, 78.64, 77.61, 77.27, 75.88, 75.32, 75.19, 75.01, 74.72, 74.57, 74.36, 74.30, 73.72, 73.44, 73.04, 72.26, 72.10, 71.53, 70.90, 70.67, 69.64, 69.26, 68.50, 67.01, 62.38, 60.42, 54.48, 29.73, 23.25, 18.12, 18.08, 12.10; MALDI-TOF: $[M + Na]^+$ calcd for $C_{112}H_{129}NNaO_{21}Si^+$, 1876.32; found, 1875.81.

Benzyl 2,3,4-tri-*O*-benzyl-α-D-mannopyranosyl-(1→2)-3,4,6-tri-*O*-benzyl-α-D-mannopyranosyl-(1→3)-2-*O*-benzyl-4,6-*O*-benzylidene-β-D-mannopyranosyl-(1→4)-2-acetamido-3,6-di-*O*-benzyl-2-deoxy-β-D-glucopyranoside (41)

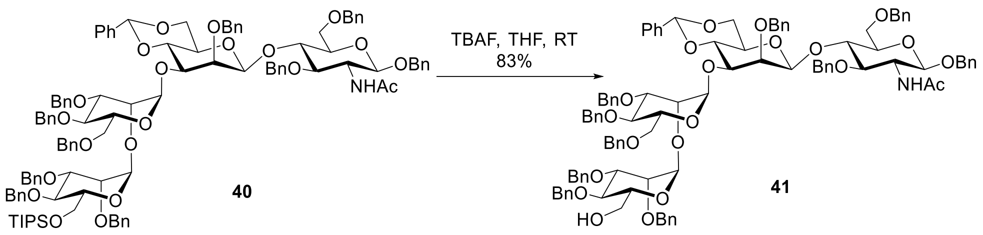

To a solution of compound **40** (115.8 mg, 0.063 mmol) in THF (2.0 mL) was added TBAF (1 M in THF, 313 µL), and the mixture was stirred at room temperature for 23 h. After the completion of the reaction as monitored by TLC, the resulting mixture was concentrated and subjected to flash chromatography on silica gel (hexanes/EtOAc = 2:1~2:3) to afford compound **41** (88.0 mg, 83%) as colorless syrup. R_f = 0.10 (hexanes/EtOAc = 3:2); ^{1}H NMR (400 MHz, $CDCl_3$) δ 7.46-7.21 (55H, m), 5.78 (1H, d, J = 8.0 Hz), 5.49 (1H, s), 5.25 (1H, d, J = 1.5 Hz), 5.04 (1H, m), 4.96 (1H, d, J = 6.7 Hz), 4.93-4.79 (6H, m), 4.65-4.48 (15H, m), 4.43 (1H, m), 4.10-4.04 (3H, m), 4.00-3.79 (9H, m), 3.78-3.69 (5H, m), 3.64-3.53 (5H, m), 3.29-3.26 (2H, m), 3.09 (1H, m), 1.75 (3H, s); ^{13}C NMR (100 MHz, $CDCl_3$) δ 170.19, 138.82, 138.65, 138.60, 138.51, 138.45, 138.37, 138.20, 138.16, 137.88, 137.61, 137.25, 129.12, 128.56, 128.47, 128.44, 128.38, 128.35, 128.29, 128.24, 128.22, 128.18, 128.05, 127.99, 127.95, 127.91, 127.85, 127.82, 127.77, 127.73, 127.65, 127.52, 127.48, 126.08, 101.67, 101.44, 99.97, 99.58, 99.17, 79.58, 78.88, 78.67, 77.70, 77.26, 75.78, 75.60, 75.25, 75.10, 74.92, 74.84, 74.77, 74.43, 73.63, 73.50, 73.38, 72.83, 72.41, 72.32, 72.21, 70.72, 69.55, 69.07, 68.53, 66.93, 61.84, 54.91, 29.73, 23.31; MALDI-TOF: $[M + Na]^+$ calcd for $C_{103}H_{109}NNaO_{21}^+$, 1719.98; found, 1719.60.

Benzyl 2,3,4-tri-*O*-benzyl-6-*O*-dibenzylphosphonato-α-D-mannopyranosyl-(1→2)-3,4,6-tri-*O*-benzyl-α-D-mannopyranosyl-(1→3)-2-*O*-benzyl-4,6-*O*-benzylidene-β-D-mannopyranosyl-(1→4)-2-acetamido-3,6-di-*O*-benzyl-2-deoxy-β-D-glucopyranoside (42)

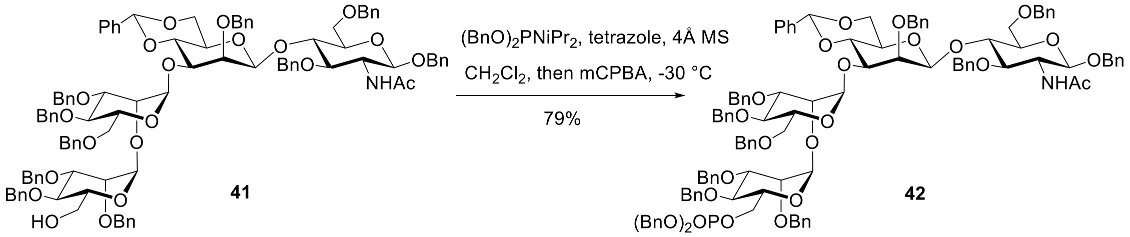

To a solution of compound **41** (73.6 mg, 0.043 mmol) in anhydrous CH_2Cl_2 (2.5 mL) was added activated 4Å molecular sieves (250 mg) and tetrazole (0.45 M in MeCN, 482 µL) and the mixture was stirred at room temperature for 1.5 h before $(BnO)_2PNiPr_2$ (58.6 µL) was added. The resulting mixture was further stirred overnight under argon atmosphere at room temperature until the complete disappearance of the starting material. Then the reaction was cooled to -30 °C, and mCPBA (77 wt %, 51.5 mg) was added, the reaction mixture was stirred at this temperature for 1 h and then filtered through a Celite pad. The filtrate was diluted with CH_2Cl_2, washed with saturated $NaHCO_3$ (aq.), dried over Na_2SO_4 and concentrated to dryness. The residue was purified by flash chromatography (hexanes/EtOAc = 4:1~2:3) to give compound **42** (67.1 mg, 79%) as colorless syrup. R_f = 0.20 (hexanes/EtOAc = 1:1); ^{1}H NMR (400 MHz, $CDCl_3$) δ 7.41-7.17 (64H, m), 7.06 (1H, t, J = 7.5 Hz), 5.91 (1H, d, J = 8.2 Hz), 5.44 (1H, s), 5.27 (1H, m), 5.20 (1H, m), 5.06 (1H, dd, J = 6.9 Hz, J = 11.8 Hz), 5.00-4.79 (10H, m), 4.64-4.43 (14H, m), 4.41-4.36 (2H, m), 4.23 (1H, m), 4.05-3.97 (5H, m), 3.94-3.82 (5H, m), 3.80-3.73 (5H, m), 3.72-3.65 (5H, m), 3.58-3.51 (2H, m), 3.13 (1H, m), 1.72 (3H, s); ^{13}C NMR (100 MHz, $CDCl_3$) δ 170.15, 138.73, 138.55, 138.39, 138.37, 138.18, 138.13, 138.02,

137.86, 137.64, 137.33, 136.16, 136.08, 136.05, 135.97, 128.55, 128.53, 128.49, 128.44, 128.40, 128.37, 128.32, 128.28, 128.21, 128.18, 128.11, 127.94, 127.93, 127.85, 127.83, 127.79, 127.70, 127.66, 127.58, 127.52, 127.48, 126.11, 101.79, 101.34, 99.62, 99.26, 98.68, 79.95, 79.53, 78.93, 78.77, 77.26, 75.94, 75.44, 75.19, 74.96, 74.71, 74.65, 74.53, 73.91, 73.42, 73.25, 73.09, 72.85, 72.46, 72.06, 71.96, 71.30, 71.23, 70.62, 69.49, 69.15, 69.09, 69.02, 68.97, 68.54, 66.78, 53.94, 29.72, 23.20; ^{31}P NMR (146 MHz, $CDCl_3$) δ -1.39; MALDI-TOF: $[M + Na]^+$ calcd for $C_{117}H_{122}NNaO_{24}P^+$, 1980.21; found, 1979.89.

6-*O*-phosphonato-α-D-mannopyranosyl-(1→2)-α-D-mannopyranosyl-(1→3)-β-D-mannopyranosyl-(1→4)-2-acetamido-2-deoxy-αβ-D-glucopyranoside (43)

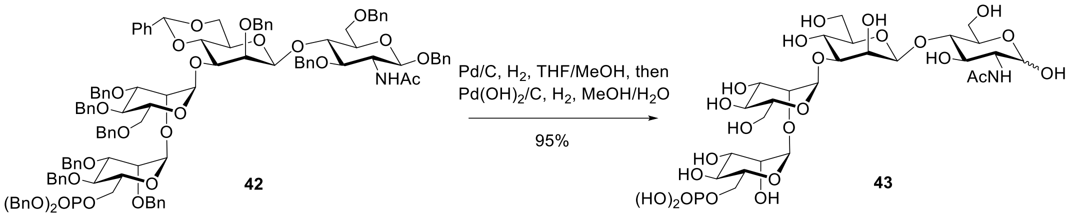

A mixture of compound **42** (67.1 mg, 0.034 mmol) and Pd/C (10 wt.% loading, 40 mg) in MeOH (2.5 mL) and THF (2.5 mL) was stirred under H_2 atmosphere for 21 h. The reaction mixture was filtered through a Celite pad, then concentrated to dryness. The mixture of the residue and $Pd(OH)_2$/C (20 wt.% loading, 50 mg) in MeOH (4.0 mL) and H_2O (4.0 mL) was stirred under H_2 atmosphere for further 21 h. The reaction mixture was filtered through a Celite pad. The filtrate was concentrated to dryness then dissolved in H_2O and lyophilized. The crude product was purified on a Sephadex G-10 column by elution with H_2O. Fractions containing the product were pooled and lyophilized to give compound **43** (25.6 mg, 95%) as white solid. R_f = 0.20 (n-BuOH/EtOH/H_2O/AcOH = 1:1:1:0.05); ^{1}H NMR (400 MHz, D_2O) δ 5.32 (0.96H, s), 5.11 (0.68H, d, J = 2.7 Hz), 4.95 (1.17H, s), 4.63-4.61 (0.80H, m), 4.12-4.11 (1.32H, m), 4.01-3.98 (2.47H, m), 3.93-3.90 (2.46H, m), 3.89-3.73 (8.07H, m), 3.73-3.51 (10.71H, m), 3.51-3.38 (1.67H, m), 1.95 (3.00H, s); ^{13}C NMR (100 MHz, D_2O) δ 174.43, 102.39, 100.66, 99.81, 99.74, 94.92, 90.51, 79.89, 79.22, 78.82, 78.50, 76.10, 74.67, 73.40, 72.54, 72.24, 70.43, 70.39, 70.13, 70.00, 69.94, 69.09, 66.96, 66.47, 66.23, 63.43, 61.05, 60.82, 60.14, 60.00, 59.39, 56.13, 53.66, 22.18, 21.88; ^{31}P NMR (146 MHz, D_2O) δ 4.52 (overlapped signals); HRMS: $[M + H]^+$ calcd for $C26H47NO24P^+$, 788.2220; found, 788.2224.

2-Methyl-[6-*O*-phosphonato-α-D-mannopyranosyl-(1→2)-α-D-mannopyranosyl-(1→3)-β-D-mannopyranosyl-(1→4)-1,2-dideoxy-α-D-glucopyrano]-[2,1-*d*]-2-oxazoline (8)

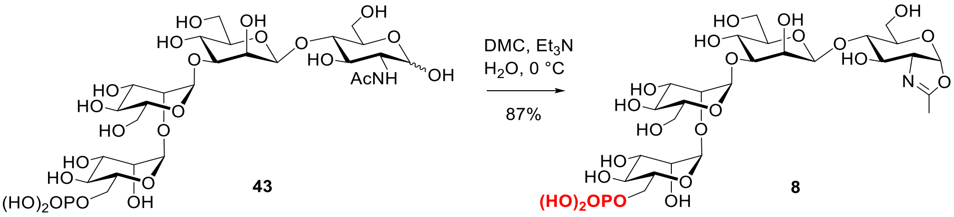

To a solution of compound **43** (5.0 mg, 0.0064 mmol) in H_2O (250 μL) were added Et_3N (35.6 μL) and 2-chloro-1,3-dimethylimidazolinium chloride (DMC, 21.5 mg) at 0 °C. The reaction mixture was monitored by DIONEX HPAEC-PAD. After 2 h, the HPAEC analysis indicated that the free oligosaccharide was converted into a new oligosaccharide that was eluted earlier than the reducing

sugar under the HPAEC condition (see general method). The product was purified by gel filtration on a Sephadex G-10 column that was eluted with 0.1% aq Et_3N to afford compound **8** (4.3 mg, 87%) as white solid after lyophilization with 5 mol.% of NaOH. ^{1}H NMR (400 MHz, D_2O) δ 6.02 (1H, m), 5.35 (1H, m), 4.97 (1H, m), 4.31 (1H, m), 4.05-4.03 (2H, m), 4.01-3.99 (1H, m), 3.96-3.89 (4H, m), 3.83-3.76 (3H, m), 3.75-3.64 (9H, m), 3.62-3.54 (3H, m), 3.46-3.41 (1H, m), 3.37-3.33 (1H, m), 2.00 (3H, s); ^{13}C NMR (100 MHz, D_2O) δ 167.21, 102.43, 100.92, 100.58, 99.87, 79.58, 78.42, 77.33, 76.18, 73.35, 72.77, 72.69, 71.07, 70.37, 70.13, 70.01, 69.13, 66.96, 66.50, 66.45, 62.83, 61.69, 61.02, 60.98, 12.96; ^{31}P NMR (146 MHz, D_2O) δ 4.52; HRMS: $[M + H]^+$ calcd for C26H45NO23P$^+$, 770.2114; found, 770.2124.

Ethyl 3,4-di-*O*-benzyl-2-*O*-benzoyl-6-*O*-triisopropylsilyl-α-D-mannopyranosyl-(1→6)-2,3,4-tri-*O*-benzyl-1-thio-α-D-mannopyranoside (45)

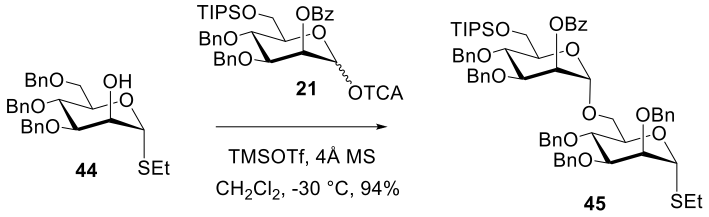

A mixture of trichloroacetimidate donor **21** [4] (502 mg, 0.658 mmol), acceptor **44** [8] (250 mg, 0.506 mmol) and activated 4Å molecular sieves (600 mg) in anhydrous CH_2Cl_2 (6.0 mL) was stirred at room temperature under argon atmosphere for 1.5 h, and then cooled to -30 °C. TMSOTf (9.25 μL, 0.051 mmol) was added. After stirring at -30 °C for 50 min, the mixture was quenched with triethylamine (50 μL) and filtered then concentrated in vacuo. The residue was purified via silica gel chromatography (hexanes/EtOAc = 20:1~10:1) to give product **45** (522 mg, 94%) as white foam. R_f = 0.30 (hexanes/EtOAc = 10:1); ^{1}H NMR (400 MHz, $CDCl_3$) δ 8.16-8.14, 7.62-7.58, 7.48-7.43, 7.38-7.20 (30H, m, Ar-*H*), 5.75 (1H, m), 5.39 (1H, m), 5.02-4.92 (3H, m), 4.80-4.75 (2H, m), 4.72-4.66 (2H, m), 4.64-4.58 (2H, m), 4.56-4.51 (2H, m), 4.17-4.06 (3H, m), 4.00-3.92 (3H, m), 3.92-3.86 (3H, m), 3.72-3.70 (2H, m), 2.65-2.51 (2H, m), 1.25 (3H, t, J = 7.4 Hz), 1.17-1.08 (21H, m); ^{13}C NMR (100 MHz, $CDCl_3$) δ 165.63, 138.98, 138.54, 138.22, 138.14, 138.11, 133.03, 130.17, 130.05, 128.41, 128.31, 128.25, 128.21, 128.19, 128.09, 127.99, 127.87, 127.81, 127.74, 127.69, 127.63, 127.51, 127.40, 98.04, 81.60, 80.52, 78.09, 76.35, 75.10, 74.99, 74.92, 73.96, 72.68, 72.08, 71.98, 71.42, 71.28, 69.01, 66.45, 62.47, 25.29, 18.09, 18.05, 15.04, 12.06; MALDI-TOF: $[M + Na]^+$ calcd for $C_{65}H_{80}NaO_{11}SSi^+$, 1119.51; found, 1119.17.

Ethyl 2,3,4-tri-*O*-benzyl-6-*O*-triisopropylsilyl-α-D-mannopyranosyl-(1→6)-2,3,4-tri-*O*-benzyl-1-thio-α-D-mannopyranoside (46)

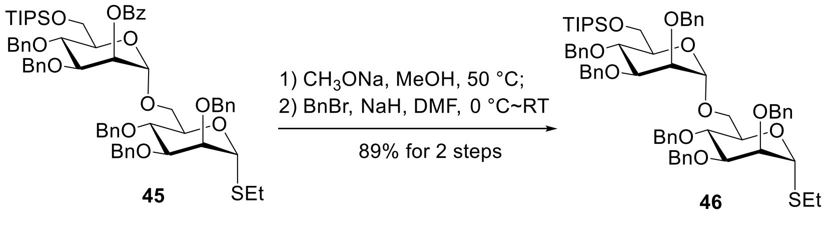

To a solution of compound **45** (300 mg, 0.274 mmol) in MeOH (4.0 mL) was added sodium methoxide until pH = 10, the solution was heated to 50 °C and stirred overnight. After the complete disappearance

of the starting material, the solution was concentrated to dryness and dissolved in dry *N*,*N*-dimethylformamide (3.0 mL) and cooled to 0 °C, sodium hydride (27.4 mg) and benzyl bromide (63.5 µL) were added successively, and the mixture was slowly warmed to room temperature. After the completion of the reaction as monitored by TLC, MeOH was added to quench the excess sodium hydride. The reaction was diluted with CH_2Cl_2, successively washed with H_2O and brine and dried over anhydrous Na_2SO_4. The residue was purified by flash column chromatography (hexanes/EtOAc = 20:1~10:1) to afford compound **46** (265 mg, 89% for 2 steps) as colorless syrup. R_f = 0.60 (hexanes/EtOAc = 10:1); ^{1}H NMR (400 MHz, $CDCl_3$) δ 7.40-7.23 (30H, m, Ar-*H*), 5.35 (1H, m), 5.05 (1H, m), 4.97-4.91 (2H, m), 4.73-4.52 (10H, m), 4.10-4.05 (1H, m), 4.04-3.98 (1H, m), 3.95-3.85 (8H, m), 3.71-3.63 (2H, m), 2.63-2.48 (2H, m), 1.23 (3H, dt, *J* = 7.3 Hz, *J* = 0.85 Hz), 1.10-1.07 (21H, m); ^{13}C NMR (100 MHz, $CDCl_3$) δ 139.06, 138.83, 138.58, 138.54, 138.24, 138.08, 128.40, 128.34, 128.31, 128.23, 128.16, 127.91, 127.87, 127.84, 127.78, 127.75, 127.69, 127.54, 127.47, 127.38, 127.28, 97.76, 81.80, 80.50, 79.73, 76.50, 75.27, 75.06, 74.97, 74.93, 74.70, 73.34, 72.34, 72.20, 72.07, 71.93, 71.66, 65.82, 63.01, 25.27, 18.06, 18.02, 15.05, 12.06; MALDI-TOF: $[M + Na]^+$ calcd for $C_{65}H_{82}NaO_{10}SSi^+$, 1105.53; found, 1105.15.

Benzyl 2,3,4-tri-*O*-benzyl-6-*O*-triisopropylsilyl-α-D-mannopyranosyl-(1→6)-2,3,4-tri-*O*-benzyl-α-D-mannopyranosyl-(1→6)-2,4-di-*O*-benzyl-3-*O*-*p*-methoxybenzyl-β-D-mannopyranosyl-(1→4)-2-azido-3,6-di-*O*-benzyl-2-deoxy-β-D-glucopyranoside (47)

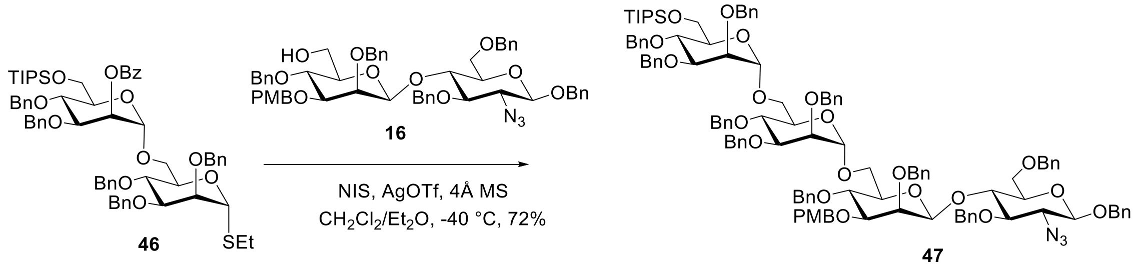

A mixture of compound **46** (120 mg, 0.111 mmol), acceptor **16** (80 mg, 0.085 mmol) and activated 4Å molecular sieves (400 mg) in anhydrous CH_2Cl_2/Et_2O (3 mL/1 mL) was stirred at room temperature under argon atmosphere for 1.5 h, and then cooled to -40 °C. N-iodosuccinimide (38.2 mg, 0.170 mmol) and AgOTf (4.4 mg, 0.017 mmol) were successively added. After stirring at -40 °C for 1 h, the mixture was quenched with triethylamine (10 µL) and filtered then concentrated in vacuo. The residue was purified via silica gel chromatography (hexanes/EtOAc = 10:1~4:1) to give the desired product **47** (120 mg, 72%) as colorless oil along with β isomer (29.5 mg, 17%). R_f = 0.40 (hexanes/EtOAc = 4:1); ^{1}H NMR (400 MHz, $CDCl_3$) δ 7.41-7.17 (57H, m, Ar-*H*), 6.87-6.85 (2H, m, Ar-*H*), 5.04-4.98 (2H, m), 4.94-4.76 (8H, m), 4.70-4.40 (15H, m), 4.37-4.28 (3H, m), 4.07-4.02 (1H, m), 4.00-3.64 (16H, m), 3.57-3.42 (4H, m), 3.41-3.33 (4H, m), 3.29-3.26 (1H, m), 1.14-1.00 (21H, m); ^{13}C NMR (100 MHz, $CDCl_3$) δ 170.64, 158.80, 138.73, 138.59, 138.40, 138.35, 138.16, 138.10, 138.06, 137.96, 137.94, 137.49, 136.44, 129.70, 128.75, 128.71, 128.13, 128.04, 127.97, 127.84, 127.79, 127.77, 127.74, 127.67, 127.58, 127.44, 127.41, 127.36, 127.32, 127.27, 127.13, 127.07, 127.00, 126.96, 126.87, 126.82, 126.77, 126.62, 113.36, 101.05, 100.16, 97.87, 97.71, 82.17, 80.61, 79.44, 78.96, 76.94, 74.91, 74.83, 74.68, 74.60, 74.42, 74.29, 73.98, 73.83, 73.78, 73.70, 73.12, 72.67, 72.28, 71.75, 71.43, 71.02, 70.85, 70.43, 68.21, 66.05, 65.55, 65.16, 62.30, 59.91, 54.79, 20.56, 17.59, 17.55, 13.73, 11.59; MALDI-TOF: $[M + Na]^+$ calcd for $C_{118}H_{135}N_3NaO_{21}Si^+$, 1980.93; found, 1981.54.

Benzyl 2,3,4-tri-*O*-benzyl-6-*O*-triisopropylsilyl-α-D-mannopyranosyl-(1→6)-2,3,4-tri-*O*-benzyl-α-D-mannopyranosyl-(1→6)-2,4-di-*O*-benzyl-3-*O*-*p*-methoxybenzyl-β-D-mannopyranosyl-(1→4)-2-acetamido-3,6-di-*O*-benzyl-2-deoxy-β-D-glucopyranoside (48)

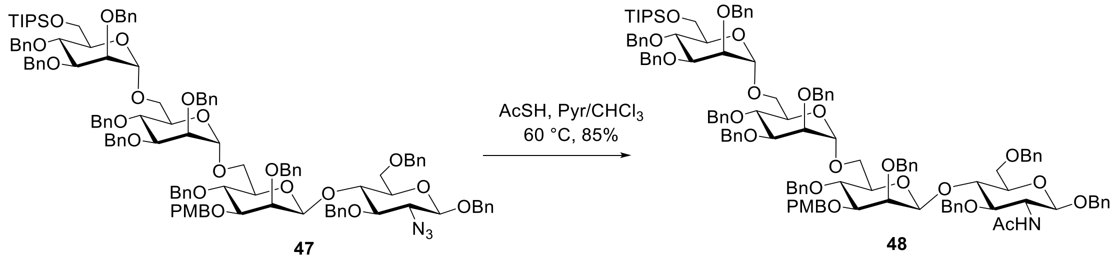

47 48

A solution of compound **47** (72.0 mg, 0.037 mmol) in a mixture of AcSH/pyridine/$CHCl_3$ (0.6 mL/0.4 mL/0.6 mL) was stirred at 60 °C for 20 h. After the completion of the reaction as monitored by TLC, the resulting mixture was concentrated and subjected to flash chromatography on silica gel (hexanes/EtOAc = 6:1~2:1) to afford compound **48** (61.7 mg, 85%) as colorless syrup. R_f = 0.30 (hexanes/EtOAc = 2:1); ^{1}H NMR (400 MHz, $CDCl_3$) δ 7.44-7.16 (57H, m, Ar-*H*), 6.88-6.86 (2H, m, Ar-*H*), 5.42 (1H, d, *J* = 7.6 Hz, N*H*), 5.01-4.84 (10H, m), 4.67-4.38 (18H, m), 4.10-4.02 (3H, m), 3.96-3.94 (1H, m), 3.89-3.77 (13H, m), 3.67-3.64 (2H, m), 3.57-3.53 (2H, m), 3.48-3.42 (2H, m), 3.36-3.33 (1H, m), 3.27-3.25 (1H, m), 1.51 (3H, s), 1.14-1.06 (21H, m); ^{13}C NMR (100 MHz, $CDCl_3$) δ 169.87, 158.77, 148.48, 138.66, 138.58, 138.42, 138.14, 138.09, 138.05, 138.02, 137.93, 137.60, 137.36, 136.24, 129.70, 128.74, 128.10, 127.98, 127.84, 127.78, 127.70, 127.66, 127.56, 127.53, 127.40, 127.37, 127.33, 127.29, 127.25, 127.18, 127.15, 127.07, 127.01, 126.96, 126.86, 126.81, 126.61, 123.54, 113.34, 100.52, 98.75, 97.61, 97.57, 82.11, 79.75, 78.96, 77.16, 74.92, 74.65, 74.47, 74.39, 74.35, 74.22, 74.13, 74.04, 73.95, 73.37, 73.07, 72.64, 72.29, 71.70, 71.41, 71.32, 70.96, 70.84, 70.39, 68.82, 66.11, 65.10, 62.31, 54.79, 22.63, 17.59, 17.55, 11.59; MALDI-TOF: $[M + Na]^+$ calcd for $C_{120}H_{139}NNaO_{22}Si^+$, 1996.95; found, 1997.36.

Benzyl 2,3,4-tri-*O*-benzyl-α-D-mannopyranosyl-(1→6)-2,3,4-tri-*O*-benzyl-α-D-mannopyranosyl-(1→6)-2,4-di-*O*-benzyl-3-*O*-*p*-methoxybenzyl-β-D-mannopyranosyl-(1→4)-2-acetamido-3,6-di-*O*-benzyl-2-deoxy-β-D-glucopyranoside (49)

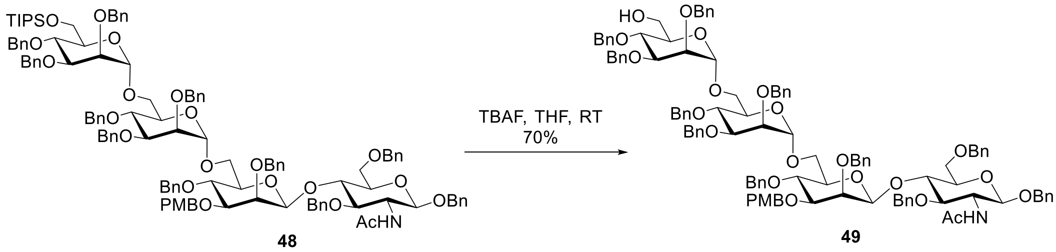

48 49

To a solution of compound **48** (61.7 mg, 0.031 mmol) in THF (1.2 mL) was added TBAF (1 M in THF, 174 µL), and the mixture was stirred at room temperature for 20 h. After the completion of the reaction as monitored by TLC, the resulting mixture was concentrated and subjected to flash chromatography on silica gel (hexanes/EtOAc = 6:1~1:1) to afford compound **49** (40.0 mg, 70%) as colorless syrup. R_f = 0.30 (hexanes/EtOAc = 1:1); ^{1}H NMR (400 MHz, $CDCl_3$) δ 7.43-7.41, 7.35-7.20 (57H, m, Ar-*H*), 6.89-6.87 (2H, m, Ar-*H*), 5.46 (1H, d, *J* = 7.5 Hz, N*H*), 5.10-5.06 (2H, m), 4.99-4.94 (4H, m), 4.92-4.85 (4H, m), 4.69-4.61 (5H, m), 4.59-4.53 (6H, m), 4.50-4.38 (7H, m), 4.17 (1H, dd, *J* = 8.4 Hz, *J* = 8.4 Hz), 4.05 (1H, dd, *J* = 8.2 Hz, *J* = 8.2 Hz), 4.00-3.94 (2H, m), 3.92-3.76 (12H, m), 3.75-3.69 (3H,

m), 3.67-3.62 (3H, m), 3.59-3.55 (1H, m), 3.48-3.45 (1H, m), 3.41-3.32 (2H, m), 3.25-3.22 (1H, m), 1.53 (3H, s); ^{13}C NMR (100 MHz, $CDCl_3$) δ 170.29, 159.26, 139.25, 138.81, 138.67, 138.65, 138.59, 138.53, 138.45, 138.43, 138.28, 138.12, 137.89, 130.20, 129.23, 128.51, 128.45, 128.39, 128.34, 128.32, 128.30, 128.26, 128.20, 128.15, 128.05, 128.02, 127.96, 127.90, 127.84, 127.75, 127.74, 127.72, 127.69, 127.64, 127.58, 127.55, 127.51, 127.49, 127.46, 127.43, 127.37, 113.84, 100.81, 99.22, 97.90, 82.57, 80.31, 79.22, 77.53, 75.34, 75.30, 75.16, 75.12, 75.05, 74.99, 74.93, 74.79, 74.76, 74.57, 74.15, 73.60, 72.80, 72.45, 72.15, 71.92, 71.76, 71.41, 71.36, 71.05, 69.31, 66.75, 65.31, 62.31, 56.54, 55.31, 29.74, 23.25; MALDI-TOF: $[M + Na]^+$ calcd for $C_{111}H_{119}NNaO_{22}^+$, 1840.81; found, 1841.28.

Benzyl 2,3,4-tri-*O*-benzyl-6-*O*-dibenzylphosphonato-α-D-mannopyranosyl-(1→6)-2,3,4-tri-*O*-benzyl-α-D-mannopyranosyl-(1→6)-2,4-di-*O*-benzyl-3-*O*-*p*-methoxybenzyl-β-D-mannopyranosyl-(1→4)-2-acetamido-3,6-di-*O*-benzyl-2-deoxy-β-D-glucopyranoside (50)

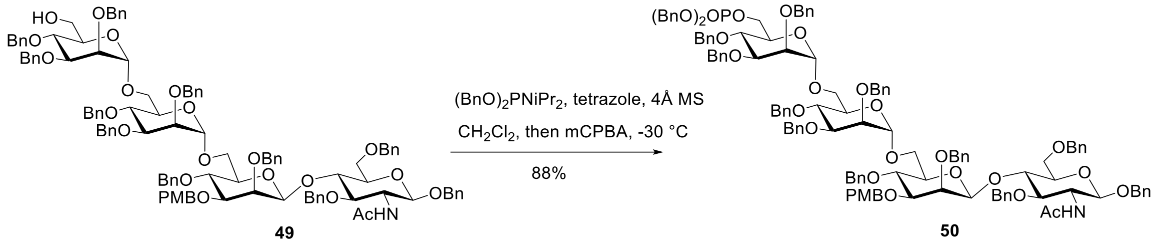

To a solution of compound **49** (40.0 mg, 0.022 mmol) in anhydrous CH_2Cl_2 (1.5 mL) was added activated 4Å molecular sieves (150 mg) and tetrazole (0.45 M in MeCN, 244 µL) and the mixture was stirred at room temperature for 1.5 h before $(BnO)_2PNiPr_2$ (37.3 µL) was added. The resulting mixture was further stirred overnight under argon atmosphere at room temperature until the complete disappearance of the starting material. Then the reaction was cooled to -30 °C, and mCPBA (77 wt %, 26.1 mg) was added, the reaction mixture was stirred at this temperature for 1 h and then filtered through a Celite pad. The filtrate was diluted with CH_2Cl_2, washed with saturated $NaHCO_3$ (aq.), dried over Na_2SO_4 and concentrated to dryness. The residue was purified by flash chromatography (hexanes/EtOAc = 6:1~3:2) to give compound **50** (40.0 mg, 88%) as colorless syrup. R_f = 0.15 (hexanes/EtOAc = 3:2); ^{1}H NMR (400 MHz, $CDCl_3$) δ 7.43-7.18 (67H, m, Ar-*H*), 6.89-6.87 (2H, m, Ar-*H*), 5.45 (1H, d, *J* = 7.6 Hz, N*H*), 5.13-5.01 (6H, m), 4.99-4.83 (8H, m), 4.67-4.34 (18H, m), 4.26-4.24 (2H, m), 4.18-4.11 (1H, m), 4.06-4.01 (2H, m), 3.97-3.92 (1H, m), 3.89-3.77 (12H, m), 3.69-3.63 (3H, m), 3.60-3.55 (2H, m), 3.49-3.46 (1H, m), 3.40-3.34 (2H, m), 3.26-3.23 (1H, m), 1.53 (3H, s); ^{13}C NMR (100 MHz, $CDCl_3$) δ 170.35, 159.27, 139.25, 138.84, 138.64, 138.61, 138.57, 138.49, 138.45, 138.21, 138.12, 137.91, 130.20, 129.25, 128.49, 128.47, 128.38, 128.34, 128.29, 128.21, 128.17, 128.06, 128.00, 127.91, 127.86, 127.83, 127.80, 127.75, 127.67, 127.51, 127.44, 127.37, 113.84, 100.95, 99.27, 98.00, 97.93, 82.60, 80.22, 79.10, 77.79, 75.31, 75.23, 75.09, 75.00, 74.92, 74.79, 74.55, 74.07, 73.83, 73.58, 72.81, 72.38, 71.89, 71.43, 71.17, 70.96, 69.34, 69.29, 69.19, 69.13, 66.52, 65.56, 56.36, 55.31, 29.74, 23.24; ^{31}P NMR (146 MHz, $CDCl_3$) δ -1.13; MALDI-TOF: $[M + Na]^+$ calcd for $C_{125}H_{132}NNaO_{25}P^+$, 2100.87; found, 2101.36.

6-*O*-phosphonato-α-D-mannopyranosyl-(1→6)-α-D-mannopyranosyl-(1→6)-β-D-mannopyranosyl-(1→4)-2-acetamido-2-deoxy-αβ-D-glucopyranoside (51)

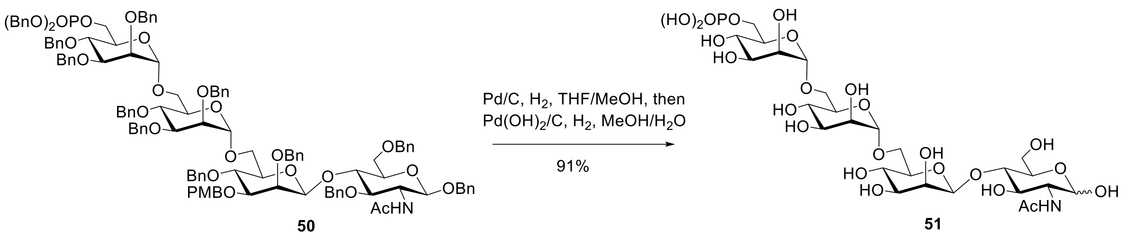

A mixture of compound **50** (40.0 mg, 0.019 mmol) and Pd/C (10 wt.% loading, 20 mg) in MeOH (1.5 mL) and THF (1.5 mL) was stirred under H_2 atmosphere for 21 h. The reaction mixture was filtered through a Celite pad, then concentrated to dryness. The mixture of the residue and $Pd(OH)_2/C$ (20 wt.% loading, 30 mg) in MeOH (2.0 mL) and H_2O (2.0 mL) was stirred under H_2 atmosphere for further 22 h. The reaction mixture was filtered through a Celite pad. The filtrate was concentrated to dryness then dissolved in H_2O and lyophilized. The crude product was purified on a Sephadex G-10 column by elution with H_2O. Fractions containing the product were pooled and lyophilized to give compound **51** (13.7 mg, 91%) as white solid. R_f = 0.20 (n-BuOH/EtOH/H_2O/AcOH = 1:1:1:0.05); ^{1}H NMR (400 MHz, D_2O) δ 5.10 (0.67H, m), 4.80 (3.45H, m), 4.62 (0.54H, m), 4.04-3.96 (3.49H, m), 3.91-3.81 (4.95H, m), 3.81-3.73 (4.83H, m), 3.73-3.64 (6.54H, m), 3.64-3.58 (3.02H, m), 3.58-3.45 (3.36H, m), 1.95 (3.00H, s); ^{13}C NMR (100 MHz, D_2O) δ 174.45, 174.13, 100.17, 100.07, 99.38, 99.34, 99.02, 98.93, 94.41, 90.03, 79.85, 79.61, 73.95, 73.77, 72.30, 71.89, 71.23, 71.18, 70.32, 70.22, 70.17, 70.12, 70.02, 69.97, 69.48, 69.41, 68.66, 66.30, 66.06, 65.80, 65.76, 65.15, 65.08, 63.55, 59.82, 59.70, 55.54, 53.13, 46.23, 21.83, 21.51; ^{31}P NMR (146 MHz, D_2O) δ 0.73 (overlapped signals); HRMS: $[M + H]^+$ calcd for $C26H47NO24P^+$, 788.2220; found, 788.2228.

2-Methyl-[6-*O*-phosphonato-α-D-mannopyranosyl-(1→6)-α-D-mannopyranosyl-(1→6)-β-D-mannopyranosyl-(1→4)-1,2-dideoxy-α-D-glucopyrano]-[2,1-*d*]-2-oxazoline (9)

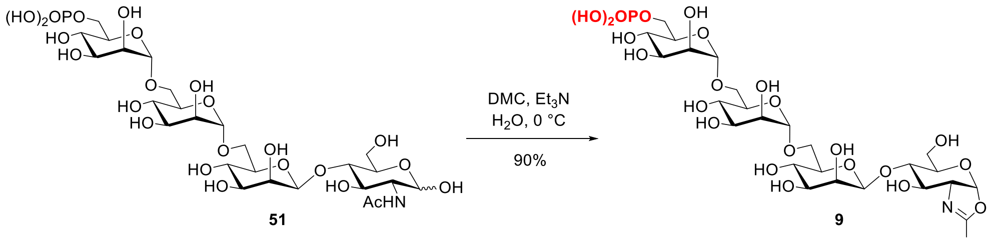

To a solution of compound **51** (7.0 mg, 0.009 mmol) in H_2O (250 μL) were added Et_3N (60 μL) and 2-chloro-1,3-dimethylimidazolinium chloride (DMC, 30 mg) at 0 °C. The reaction mixture was monitored by DIONEX HPAEC-PAD. After 2 h, the HPAEC analysis indicated that the free oligosaccharide was converted into a new oligosaccharide that was eluted earlier than the reducing sugar under the HPAEC condition (see general method). The product was purified by gel filtration on a Sephadex G-10 column that was eluted with 0.1% aq Et_3N to afford compound **9** (6.1 mg, 90%) as white solid after lyophilization with 5 mol.% of NaOH. ^{1}H NMR (400 MHz, D_2O) δ 6.02 (1H, d, J = 7.3 Hz), 4.88-4.83 (3H, m), 4.65 (1H, m), 4.31-4.29 (1H, m), 4.14-4.11 (1H, m), 4.01-3.87 (8H, m), 3.86-3.84 (1H, m), 3.80-3.76 (4H, m), 3.75-3.65 (9H, m), 3.60-3.53 (4H, m), 3.49-3.47 (1H, m), 3.37-3.34 (1H, m), 1.99 (3H, d, J = 1.6 Hz); ^{13}C NMR (100 MHz, D_2O) δ 168.25, 101.49, 99.91, 99.71, 99.67, 77.69, 74.43, 72.90, 72.23, 72.16, 70.89, 70.79, 70.48, 70.26, 70.05, 69.86, 69.18, 69.10, 66.73, 66.58, 66.42, 66.25, 66.10, 65.86, 65.62, 65.13, 62.76, 61.72, 12.97; ^{31}P NMR (146 MHz, D_2O) δ 3.99; HRMS: $[M + H]^+$ calcd for $C26H45NO23P^+$, 770.2114; found, 770.2133.

Benzyl 2,3,4-tri-*O*-benzyl-6-*O*-triisopropylsilyl-α-D-mannopyranosyl-(1→2)-3,4,6-tri-*O*-benzyl-α-D-mannopyranosyl-(1→3)-2,4-di-*O*-benzyl-β-D-mannopyranosyl-(1→4)-2-azido-3,6-di-*O*-benzyl-2-deoxy-β-D-glucopyranoside (52)

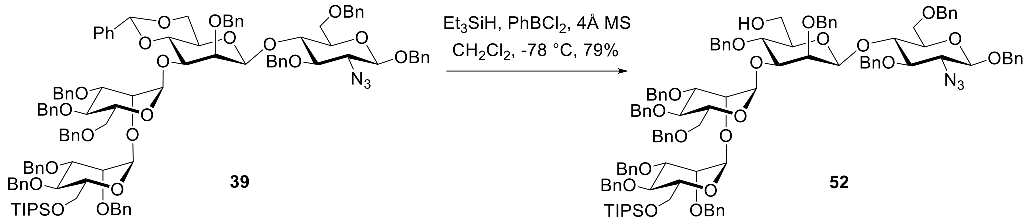

A mixture of compound **39** (130 mg, 0.071 mmol) and activated 4Å molecular sieves (250 mg) in anhydrous CH_2Cl_2 (2.5 mL) was stirred for 1.5 h at room temperature then cooled to −78 °C. Et_3SiH (89.6 μL, 0.565 mmol) and $PhBCl_2$ (45.6 μL, 0.353 mmol) were added. The resulting mixture was stirred for 2.5 h under argon at −78 °C, then Et_3N (135 μL) was added to quench the reaction. The residue was filtered through a Celite pad, diluted with CH_2Cl_2, washed with saturated $NaHCO_3$ (aq) and brine, dried over Na_2SO_4, and concentrated to dryness. Flash chromatography on silica gel (hexane/EtOAc = 10:1~ 3:1) gave compound **52** as colorless syrup (103 mg, 79%). R_f = 0.30 (hexanes/EtOAc = 4:1); Spectroscopic data were in agreement with literature values. [7] MALDI-TOF: $[M + Na]^+$ calcd for $C_{110}H_{127}N_3NaO_{20}Si^+$, 1862.30; found, 1862.04.

Benzyl 2,3,4-tri-*O*-benzyl-6-*O*-triisopropylsilyl-α-D-mannopyranosyl-(1→2)-3,4,6-tri-*O*-benzyl-α-D-mannopyranosyl-(1→3)-[2-*O*-acetyl-3,4,6-tri-*O*-benzyl-α-D-mannopyranosyl-(1→6)]-2,4-di-*O*-benzyl-β-D-mannopyranosyl-(1→4)-2-azido-3,6-di-*O*-benzyl-2-deoxy-β-D-glucopyranoside (53)

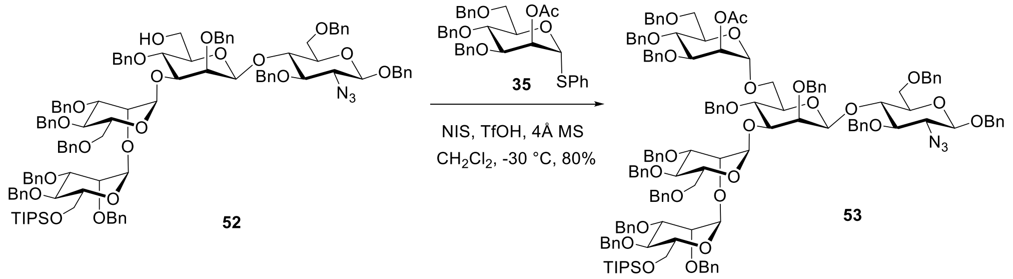

A mixture of compound **35** [6] (20 mg, 0.034 mmol), acceptor **52** (34 mg, 0.018 mmol) and activated 4Å molecular sieves (100 mg) in anhydrous CH_2Cl_2 (1.0 mL) was stirred at room temperature under argon atmosphere for 1.5 h, and then cooled to -30 °C. N-iodosuccinimide (14.7 mg, 0.065 mmol) and TfOH (0.38 μL, 0.004 mmol) were successively added. After stirring at -30 °C for 40 min, the mixture was quenched with triethylamine (5 μL) and filtered then concentrated in vacuo. The residue was purified via silica gel chromatography (hexanes/EtOAc = 10:1~4:1) to give the pentasaccharide **53** (34 mg, 80%) as colorless syrup. R_f = 0.40 (hexanes/EtOAc = 4:1); ^{1}H NMR (400 MHz, $CDCl_3$) δ 7.40-7.17 (70H, m), 5.38 (1H, m), 5.23 (1H, m), 5.13 (1H, m), 5.07 (1H, d, J = 11.3 Hz), 5.00 (1H, d, J = 11.3 Hz), 4.96-4.92 (2H, m), 4.88 (1H, d, J = 5.9 Hz), 4.84 (1H, m), 4.80 (1H, d, J = 7.2 Hz), 4.78-4.75 (2H, m), 4.72 (1H, m), 4.70-4.62 (4H, m), 4.60 (1H, m), 4.57-4.52 (6H, m), 4.51-4.40 (7H, m), 4.32-4.30 (1H, m), 4.29-4.21 (3H, m), 4.05-3.91 (7H, m), 3.90-3.84 (3H, m), 3.81-3.75 (2H, m), 3.70-3.54 (11H, m), 3.51-3.45 (2H, m), 3.32 (1H, dd, J = 9.1 Hz, J = 9.1 Hz), 3.25-3.17 (3H, m), 2.08 (3H, s), 1.06 (21H, m); ^{13}C NMR (100 MHz, $CDCl_3$) δ 170.12, 139.07, 138.99, 138.85, 138.74, 138.65, 138.58, 138.54, 138.47, 138.40, 138.06, 138.05, 137.98, 137.95, 137.01, 128.53, 128.43, 128.37,

128.32, 128.29, 128.27, 128.24, 128.21, 128.20, 128.15, 128.07, 128.05, 128.01, 127.91, 127.85, 127.75, 127.71, 127.65, 127.56, 127.51, 127.48, 127.26, 101.51, 101.09, 100.48, 98.56, 97.81, 82.28, 81.14, 80.22, 79.50, 78.71, 78.16, 77.26, 75.16, 75.02, 74.99, 74.87, 74.80, 74.62, 74.52, 74.43, 74.20, 74.05, 73.83, 73.42, 73.35, 73.19, 72.98, 72.44, 72.14, 71.62, 71.41, 70.71, 69.96, 68.56, 68.40, 66.68, 65.70, 62.46, 29.74, 21.07, 18.09, 18.02, 12.06; MALDI-TOF: $[M + Na]^+$ calcd for $C_{139}H_{158}N_3O_{26}Si^+$, 2336.85; found, 2336.24.

Benzyl 2,3,4-tri-*O*-benzyl-6-*O*-triisopropylsilyl-α-D-mannopyranosyl-(1→2)-3,4,6-tri-*O*-benzyl-α-D-mannopyranosyl-(1→3)-[2,3,4,6-tetra-*O*-benzyl-α-D-mannopyranosyl-(1→6)]-2,4-di-*O*-benzyl-β-D-mannopyranosyl-(1→4)-2-azido-3,6-di-*O*-benzyl-2-deoxy-β-D-glucopyranoside (54)

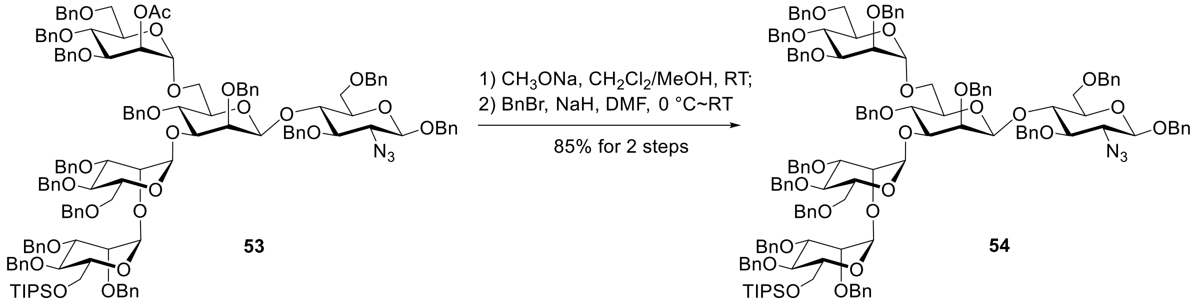

To a solution of compound **53** (34.0 mg, 0.0147 mmol) in MeOH (1.0 mL) and CH_2Cl_2 (1.0 mL) was added sodium methoxide until pH = 10, the solution was stirred at room temperature overnight. After the complete disappearance of the starting material, the solution was concentrated to dryness and dissolved in dry *N*,*N*-dimethylformamide (2.0 mL) and cooled to 0 °C, sodium hydride (3.6 mg, 0.088 mmol) and benzyl bromide (8.5 μL, 0.074 mmol) were added successively, and the mixture was slowly warmed to room temperature. After the completion of the reaction as monitored by TLC, MeOH was added to quench the excess sodium hydride. The reaction was diluted with CH_2Cl_2, successively washed with H_2O and brine and dried over anhydrous Na_2SO_4. The residue was purified by flash column chromatography (hexanes/EtOAc = 15:1~3:1) to afford the compound **54** (29.6 mg, 85% for 2 steps) as colorless syrup. R_f = 0.70 (hexanes/EtOAc = 3:1); ^{1}H NMR (400 MHz, $CDCl_3$) δ 7.39-7.18 (75H, m), 5.24 (1H, m), 5.14 (1H, m), 5.03-4.83 (7H, m), 4.79-4.64 (6H, m), 4.60-4.35 (19H, m), 4.24-4.20 (2H, m), 4.06-3.92 (7H, m), 3.90-3.82 (3H, m), 3.75 (2H, m), 3.69-3.50 (12H, m), 3.47-3.38 (2H, m), 3.29 (1H, dd, J = 9.4 Hz, J = 9.4 Hz), 3.21-3.15 (2H, m), 1.08-1.03 (21H, m); ^{13}C NMR (100 MHz, $CDCl_3$) δ 139.07, 139.03, 138.84, 138.67, 138.61, 138.55, 138.53, 138.38, 138.20, 137.94, 137.91, 136.96, 128.50, 128.44, 128.37, 128.32, 128.27, 128.24, 128.21, 128.18, 128.15, 128.05, 127.99, 127.91, 127.75, 127.71, 127.67, 127.65, 127.60, 127.57, 127.51 ,127.46, 127.40, 127.29, 127.26, 127.15, 127.01, 101.49, 101.34, 100.46, 98.52, 98.07, 82.18, 80.94, 80.27, 79.88, 79.49, 78.94, 77.26, 75.16, 75.05, 74.91, 74.84, 74.77, 74.66, 74.62, 74.50, 74.30, 74.20, 73.83, 73.42, 73.30, 73.18, 72.98, 72.40, 72.30, 72.13, 72.01, 71.59, 71.48, 70.74, 69.95, 68.99, 68.41, 66.32, 65.81, 62.45, 29.73, 18.09, 18.03, 12.06; MALDI-TOF: $[M + Na]^+$ calcd for $C_{144}H_{161}N_3NaO_{25}Si^+$, 2384.94; found, 2384.08.

Benzyl 2,3,4-tri-*O*-benzyl-6-*O*-triisopropylsilyl-α-D-mannopyranosyl-(1→2)-3,4,6-tri-*O*-benzyl-α-D-mannopyranosyl-(1→3)-[2,3,4,6-tetra-*O*-benzyl-α-D-mannopyranosyl-(1→6)]-2,4-di-*O*-benzyl-β-D-mannopyranosyl-(1→4)-2-acetamido-3,6-di-*O*-benzyl-2-deoxy-β-D-glucopyranoside (55)

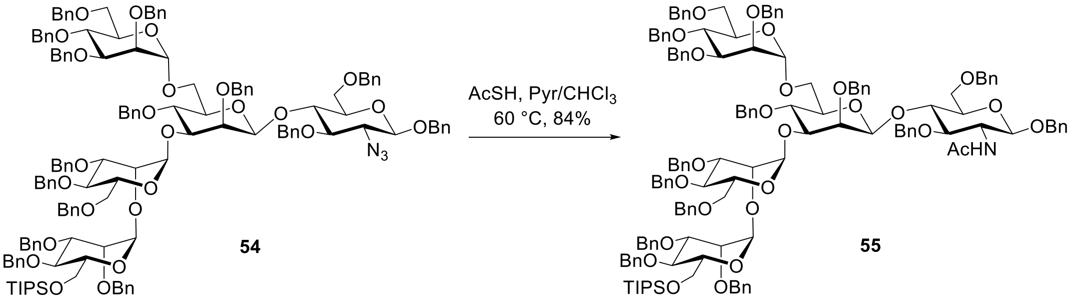

A solution of compound **54** (29.6 mg, 0.013 mmol) in a mixture of AcSH/pyridine/$CHCl_3$ (0.3 mL/0.2 mL/0.3 mL) was stirred at 60 °C for 23 h. After the completion of the reaction as monitored by TLC, the resulting mixture was concentrated and subjected to flash chromatography on silica gel (hexanes/EtOAc = 6:1~2:1) to afford compound **55** (25.0 mg, 84%) as colorless syrup. R_f = 0.30 (hexanes/EtOAc = 2:1); ^{1}H NMR (400 MHz, $CDCl_3$) δ 7.36-7.13 (75H, m), 5.23 (1H, m), 5.17-5.12 (2H, m), 5.03 (1H, d, J = 11.9 Hz), 4.96-4.91 (3H, m), 4.90-4.80 (4H, m), 4.76-4.74 (2H, m), 4.69 (1H, d, J = 10.7 Hz), 4.65 (1H, m), 4.61-4.42 (19H, m), 4.42-4.37 (2H, m), 4.31 (1H, d, J = 12.0 Hz), 4.21 (1H, dd, J = 9.6 Hz, J = 9.6 Hz), 4.08-3.99 (5H, m), 3.94-3.89 (3H, m), 3.88-3.84 (2H, m), 3.79 (1H, dd, J = 9.7 Hz, J = 9.7 Hz), 3.74 (2H, m), 3.70-3.62 (9H, m), 3.59-3.56 (1H, m), 3.55-3.53 (1H, m), 3.51-3.49 (2H, m), 3.34 (1H, q, J = 7.4 Hz), 3.27 (1H, m), 3.21 (1H, d, J = 10.8 Hz), 1.49 (3H, s), 1.08-1.01 (21H, m); ^{13}C NMR (100 MHz, $CDCl_3$) δ 170.07, 139.09, 138.96, 138.84, 138.81, 138.70, 138.67, 138.57, 138.54, 138.35, 138.14, 138.09, 137.97, 137.91, 128.42, 128.38, 128.30, 128.27, 128.23, 128.19, 128.14, 128.05, 128.02, 127.97, 127.81, 127.75, 127.71, 127.70, 127.65, 127.61, 127.56, 127.49, 127.43, 127.28, 101.45, 100.92, 99.16, 98.56, 97.99, 82.06, 80.29, 79.52, 78.87, 77.77, 77.26, 75.33, 75.12, 75.03, 74.90, 74.72, 74.22, 73.91, 73.81, 73.42, 73.19, 72.95, 72.40, 72.29, 72.14, 71.87, 71.82, 71.62, 70.83, 69.71, 68.99, 68.85, 66.56, 62.50, 56.12, 31.96, 29.73, 23.29, 22.72, 18.09, 18.03, 12.06; MALDI-TOF: $[M + Na]^+$ calcd for $C_{146}H_{165}NNaO_{26}Si^+$, 2399.13; found, 2398.92.

Benzyl 2,3,4-tri-*O*-benzyl-α-D-mannopyranosyl-(1→2)-3,4,6-tri-*O*-benzyl-α-D-mannopyranosyl-(1→3)-[2,3,4,6-tetra-*O*-benzyl-α-D-mannopyranosyl-(1→6)]-2,4-di-*O*-benzyl-β-D-mannopyranosyl-(1→4)-2-acetamido-3,6-di-*O*-benzyl-2-deoxy-β-D-glucopyranoside (56)

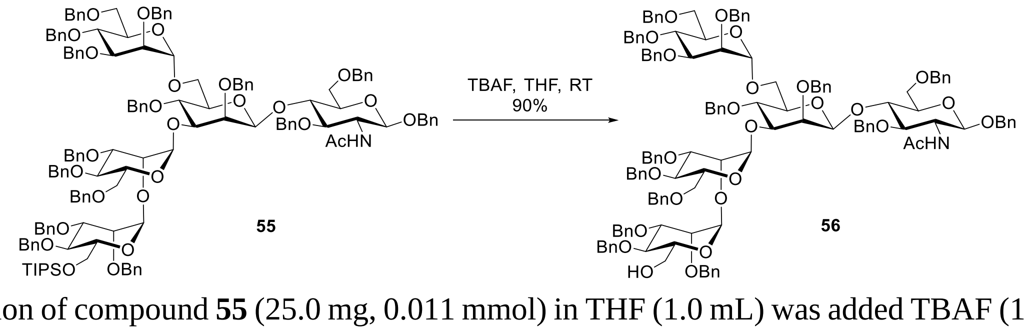

To a solution of compound **55** (25.0 mg, 0.011 mmol) in THF (1.0 mL) was added TBAF (1 M in THF, 84.2 μL), and the mixture was stirred at room temperature for 18 h. After the completion of the reaction as monitored by TLC, the resulting mixture was concentrated and subjected to flash chromatography on silica gel (hexanes/EtOAc = 4:1~1:1) to afford compound **56** (21.0 mg, 90%) as colorless syrup. R_f = 0.10 (hexanes/EtOAc = 2:1); ^{1}H NMR (400 MHz, $CDCl_3$) δ 7.37-7.15 (75H, m), 5.20 (1H, m), 5.15 (1H, d, J = 7.5 Hz), 5.03-5.01 (2H, m), 4.96-4.82 (7H, m), 4.76 (1H, d, J = 11.8 Hz), 4.71 (1H, d, J = 11.7 Hz), 4.63-4.40 (21H, m), 4.33 (1H, d, J = 12.1 Hz), 4.08-3.96 (4H, m), 3.95-3.86 (5H, m), 3.85-3.73 (6H, m), 3.72-3.63 (6H, m), 3.58-3.50 (5H, m), 3.40-3.33 (1H, m), 3.32-3.25 (2H, m), 1.95 (1H,

m), 1.51 (3H, s); ^{13}C NMR (100 MHz, $CDCl_3$) δ 170.04, 139.33, 138.92, 138.82, 138.69, 138.65, 138.58, 138.56, 138.49, 138.39, 138.37, 138.19, 138.09, 137.98, 137.89, 128.53, 128.46, 128.36, 128.31, 128.27, 128.22, 128.21, 128.19, 128.15, 127.99, 127.81, 127.79, 127.70, 127.60, 127.51, 127.47, 127.43, 127.33, 101.26, 100.91, 100.09, 99.17, 98.04, 82.19, 80.28, 79.62, 79.53, 78.85, 77.77, 77.26, 75.77, 75.26, 75.20, 75.01, 74.92, 74.85, 74.75, 73.90, 73.47, 73.41, 73.22, 72.99, 72.80, 72.61, 72.48, 72.34, 72.27, 71.96, 71.86, 70.81, 69.54, 68.96, 68.89, 66.55, 62.21, 56.12, 29.73, 23.29; MALDI-TOF: [M + Na]$^+$ calcd for $C_{137}H_{145}NNaO_{26}^+$, 2242.99; found, 2243.15.

Benzyl 2,3,4-tri-*O*-benzyl-6-*O*-dibenzylphosphonato-α-D-mannopyranosyl-(1→2)-3,4,6-tri-*O*-benzyl-α-D-mannopyranosyl-(1→3)-[2,3,4,6-tetra-*O*-benzyl-α-D-mannopyranosyl-(1→6)]-2,4-di-*O*-benzyl-β-D-mannopyranosyl-(1→4)-2-acetamido-3,6-di-*O*-benzyl-2-deoxy-β-D-glucopyranoside (57)

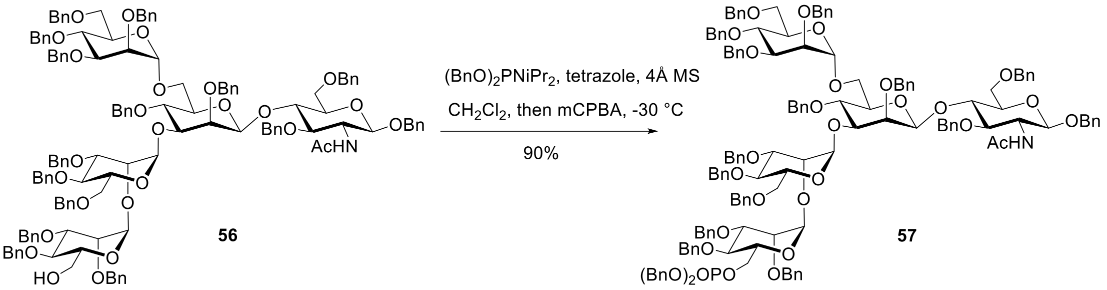

To a solution of compound **56** (21.0 mg, 0.0095 mmol) in anhydrous CH_2Cl_2 (1.0 mL) was added activated 4Å molecular sieves (100 mg) and tetrazole (0.45 M in MeCN, 105 µL) and the mixture was stirred at room temperature for 1.5 h before $(BnO)_2PNiPr_2$ (12.8 µL) was added. The resulting mixture was further stirred overnight under argon atmosphere at room temperature until the complete disappearance of the starting material. Then the reaction was cooled to -30 °C, and mCPBA (77 wt %, 11.2 mg) was added, the reaction mixture was stirred at this temperature for 1 h and then filtered through a Celite pad. The filtrate was diluted with CH_2Cl_2, washed with saturated $NaHCO_3$ (aq.), dried over Na_2SO_4 and concentrated to dryness. The residue was purified by flash chromatography (hexanes/EtOAc = 4:1~1:1) to give compound **57** (21.0 mg, 90%) as colorless syrup. R_f = 0.10 (hexanes/EtOAc = 2:1); ^{1}H NMR (400 MHz, $CDCl_3$) δ 7.34-7.13 (85H, m), 5.28 (1H, d, J = 7.7 Hz), 5.12-5.07 (2H, m), 5.05-5.02 (2H, m), 4.98-4.93 (5H, m), 4.90-4.79 (6H, m), 4.74-4.71 (3H, m), 4.60-4.58 (2H, m), 4.56-4.53 (5H, m), 4.51-4.43 (12H, m), 4.40-4.31 (3H, m), 4.15-4.13 (2H, m), 4.07-3.97 (7H, m), 3.93-3.85 (6H, m), 3.84-3.78 (3H, m), 3.75-3.68 (6H, m), 3.60-3.55 (4H, m), 3.52-3.51 (1H, m), 3.44 (1H, q, J = 7.5 Hz), 3.39-3.34 (1H, m), 3.26 (1H, m), 1.50 (3H, s); ^{13}C NMR (100 MHz, $CDCl_3$) δ 169.63, 138.74, 138.44, 138.36, 138.21, 138.14, 138.04, 137.97, 137.91, 137.84, 137.74, 137.61, 137.51, 137.42, 135.51, 135.46, 135.41, 135.36, 132.59, 129.17, 128.22, 128.06, 127.99, 127.97, 127.88, 127.87, 127.83, 127.82, 127.79, 127.74, 127.70, 127.67, 127.57, 127.55, 127.50, 127.42, 127.35, 127.30, 127.25, 127.24, 127.17, 127.09, 127.03, 126.99, 126.93, 126.89, 126.78, 126.70, 100.67, 100.33, 98.96, 98.65, 97.52, 81.75, 79.78, 79.23, 79.05, 78.53, 77.00, 74.91, 74.77, 74.71, 74.53, 74.49, 74.36, 74.27, 74.24, 74.18, 74.12, 73.57, 73.12, 72.86, 72.84, 72.69, 72.26, 71.90, 71.82, 71.72, 71.58, 71.44, 71.33, 70.21, 69.00, 68.82, 68.69, 68.65, 68.61, 68.57, 68.40, 66.87, 66.83, 66.24, 66.04, 54.88, 29.21, 22.70; ^{31}P NMR (146 MHz, $CDCl_3$) δ -1.33; MALDI-TOF: [M + Na]$^+$ calcd for $C_{151}H_{158}NNaO_{29}P^+$, 2503.05; found, 2502.94.

6-*O*-phosphonato-α-D-mannopyranosyl-(1→2)-α-D-mannopyranosyl-(1→3)-[α-D-

mannopyranosyl-(1→6)]-β-D-mannopyranosyl-(1→4)-2-acetamido-2-deoxy-αβ-D-glucopyranoside (58)

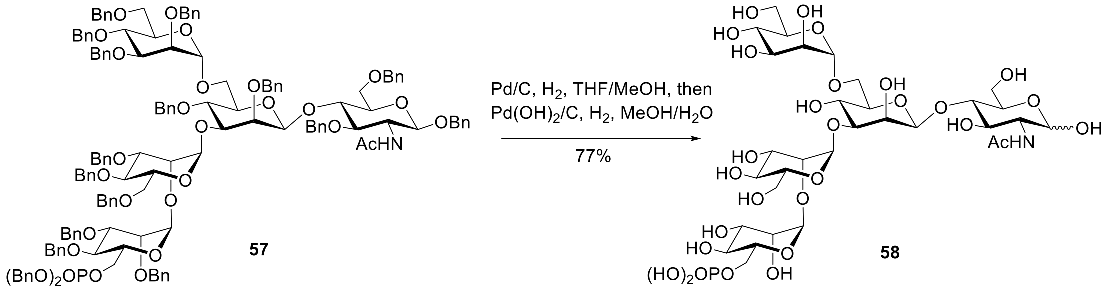

A mixture of compound **57** (44.6 mg, 0.018 mmol) and Pd/C (10 wt.% loading, 20 mg) in MeOH (1.5 mL) and THF (1.5 mL) was stirred under H_2 atmosphere for 21 h. The reaction mixture was filtered through a Celite pad, then concentrated to dryness. The mixture of the residue and Pd(OH)$_2$/C (20 wt.% loading, 30 mg) in MeOH (2.0 mL) and H_2O (2.0 mL) was stirred under H_2 atmosphere for further 22 h. The reaction mixture was filtered through a Celite pad. The filtrate was concentrated to dryness then dissolved in H_2O and lyophilized. The crude product was purified on a Sephadex G-10 column by elution with H_2O. Fractions containing the product were pooled and lyophilized to give compound **58** (13.1 mg, 77%) as white solid. R_f = 0.30 (n-BuOH/EtOH/H_2O/AcOH = 1:1:1:0.05); ^{1}H NMR (400 MHz, D_2O) δ 5.34 (1.00H, m), 5.15 (1H, d, J = 3.2 Hz), 4.97 (1.14H, m), 4.87 (1.16H, m), 4.76-4.75 (1.80H, m), 4.66-4.65 (1.00H, m), 4.17-4.15 (1.19H, m), 4.03-4.01 (2.51H, m), 3.99-3.96 (1.55H, m), 3.95-3.89 (3.98H, m), 3.88-3.76 (11.96H, m), 3.74-3.64 (11.78H, m), 3.62-3.55 (3.85H, m), 1.99 (3.00H, m); ^{13}C NMR (100 MHz, D_2O) δ 174.42, 174.13, 102.00, 100.33, 99.73, 99.26, 94.50, 90.07, 79.69, 79.63, 79.29, 78.28, 74.11, 73.75, 73.01, 72.27, 72.12, 72.07, 71.82, 69.99, 69.92, 69.77, 69.56, 69.51, 68.65, 66.60, 66.45, 66.09, 65.85, 65.62, 65.53, 63.10, 60.68, 60.56, 59.76, 59.63, 55.67, 53.20, 21.85, 21.54; ^{31}P NMR (146 MHz, D_2O) δ 2.94 (overlapped signals); HRMS: $[M + H]^+$ calcd for $C32H57NO29P^+$, 950.2748; found, 950.2755.

2-Methyl-[6-*O*-phosphonato-α-D-mannopyranosyl-(1→2)-α-D-mannopyranosyl-(1→3)-[α-D-mannopyranosyl-(1→6)]-β-D-mannopyranosyl-(1→4)-1,2-dideoxy-α-D-glucopyrano]-[2,1-*d*]-2-oxazoline (10)

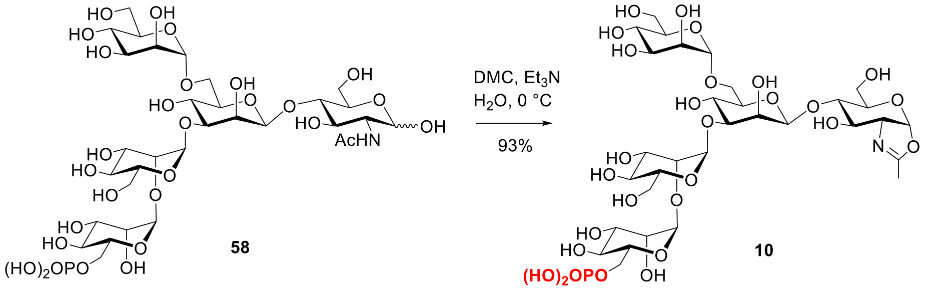

To a solution of compound **58** (8.0 mg, 0.0084 mmol) in H_2O (250 μL) were added Et_3N (47.2 μL) and 2-chloro-1,3-dimethylimidazolinium chloride (DMC, 28.6 mg) at 0 °C. The reaction mixture was monitored by DIONEX HPAEC-PAD. After 2 h, the HPAEC analysis indicated that the free oligosaccharide was converted into a new oligosaccharide that was eluted earlier than the reducing sugar under the HPAEC condition (see general method). The product was purified by gel filtration on a Sephadex G-10 column that was eluted with 0.1% aq Et_3N to afford compound **10** (7.3 mg, 93%) as white solid after lyophilization with 5 mol.% of NaOH. ^{1}H NMR (400 MHz, D_2O) δ 6.01 (1H, d, J = 7.3 Hz), 5.32 (1H, m), 4.94 (1H, m), 4.88 (1H, m), 4.32-4.31 (1H, m), 4.12-4.11 (1H, m), 4.04-3.98

(3H, m), 3.95-3.85 (6H, m), 3.84-3.80 (2H, m), 3.79-3.73 (5H, m), 3.71-3.62 (8H, m), 3.61-3.55 (4H, m), 3.35-3.32 (1H, m), 1.99 (3H, d, J = 1.7 Hz); ^{13}C NMR (100 MHz, D_2O) δ 167.65, 102.48, 101.25, 100.69, 99.92, 99.59, 80.03, 78.74, 77.78, 74.31, 73.33, 72.68, 72.59, 70.96, 70.50, 70.37, 70.13, 69.98, 69.95, 69.89, 69.05, 66.95, 66.84, 66.43, 66.20, 65.73, 65.03, 62.99, 61.67, 61.00, 60.94, 12.95; ^{31}P NMR (146 MHz, D_2O) δ 4.02; HRMS: $[M + H]^+$ calcd for $C32H55NO28P^+$, 932.2643; found, 932.2669.

Synthesis of the GlcNAc-peptide derived from rhGAA containing N470 glycosite.

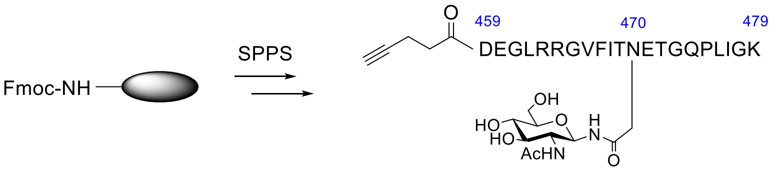

The GlcNAc-Peptide was obtained from SPPS. Synthesis was based on Fmoc chemistry using Rink Amide AM resin (0.66 mmol/g) on a 0.1 mmol scale. Couplings were performed using 5 equiv. of Fmoc-protected amino acids, 5 equiv. of HOBT and 5 equiv. of DIC in DMF. The GlcNAc-Asn building block (3 equiv.) was coupled to the growing peptide at 90 °C with a 50 Hz MW power for 10 min, Fmoc-Arg(Pbf)-OH was double coupled (RT without MW for 25 min, followed by 90 °C with 50 Hz MW power for 2 min), and all other amino acids were coupled at 90 °C with 50 Hz MW power for 2 min. Fmoc deprotection was carried out with 20% piperidine in DMF containing 0.1 M HOBt. Upon completion of the sequence, 4-pentynoic acid was coupled at the N-terminus to install the alkyne group. The resin was washed with DMF (3x) and DCM (3x) then cleavage was carried out using cocktail R (TFA/Thioanisole/Ethanedithiol/Anisole = 90/5/3/2) treatment for 2 h. The resin was then filtered and the solution was added to cold diethyl ether for precipitation. The crude peptide was purified on preparative RP-HPLC to afford the peptide (99.1 mg, 38% yield over all steps).
ESI-MS: Calcd., M = 2583.89; found (m/z): 646.90 $[M + 4H]^{4+}$, 861.43 $[M + 3H]^{3+}$, 1292.36 $[M + 2H]^{2+}$. Deconvolution of the ESI-MS: M = 2583.4; RP-HPLC retention time, t_R = 18.7 min (gradient, 5−40% aq MeCN containing 0.1% FA for 30 min; flow rate, 1.0 mL/min).

Synthesis of glycopeptide 59

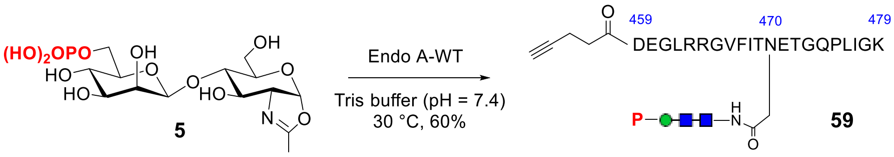

GlcNAc-peptide (3.0 mg, 1.16 µmol) was incubated at 30 °C together with oxazoline **5** (2.1 mg, 4 eq) and Endo A-WT (120 µg) in Tris buffer (100 mM, pH 7.4, 100 µL). The reaction was monitored by analytical RP-HPLC. Upon completion of the transglycosylation, the reaction was quenched using 0.1% aq. TFA and purified by RP-HPLC (gradient, 10−40% aq MeCN containing 0.1% FA for 30 min; flow rate, 4.0 mL/min) to give glycopeptides **59** (2.1 mg, 60%) as white solid. ESI-MS: Calcd., M = 3029.20; found (m/z): 758.13 $[M + 4H]^{4+}$, 1010.14 $[M + 3H]^{3+}$, 1515.15 $[M + 2H]^{2+}$. Deconvolution of the ESI-MS: M = 3028.7; RP-HPLC retention time, t_R = 19.5 min (gradient, 5−40% aq MeCN containing 0.1% FA for 30 min; flow rate, 1.0 mL/min).

Synthesis of glycopeptide 60

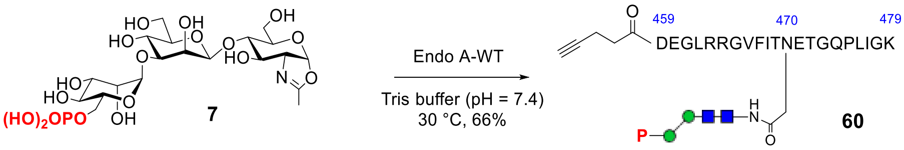

GlcNAc-peptide (3.0 mg, 1.16 µmol) was incubated at 30 °C together with oxazoline **7** (3.5 mg, 5 eq) and Endo A-WT (120 µg) in Tris buffer (100 mM, pH 7.4, 100 µL). The reaction was monitored by analytical RP-HPLC. Upon completion of the transglycosylation, the reaction was quenched using 0.1%

aq. TFA and purified by RP-HPLC (gradient, 10−40% aq MeCN containing 0.1% FA for 30 min; flow rate, 4.0 mL/min) to give glycopeptides **60** (2.4 mg, 66%) as white solid. ESI-MS: Calcd., M = 3191.34; found (m/z): 798.74 $[M + 4H]^{4+}$, 1064.61 $[M + 3H]^{3+}$, 1596.46 $[M + 2H]^{2+}$. Deconvolution of the ESI-MS: M = 3190.9; RP-HPLC retention time, t_R = 19.4 min (gradient, 5−40% aq MeCN containing 0.1% FA for 30 min; flow rate, 1.0 mL/min).

Synthesis of glycopeptide 61

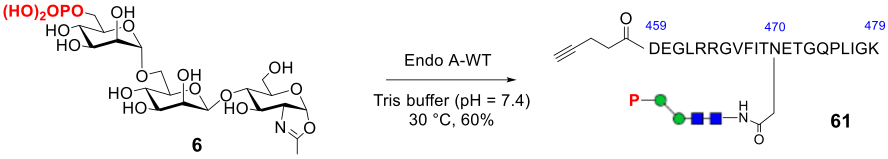

GlcNAc-peptide (3.0 mg, 1.16 μmol) was incubated at 30 °C together with oxazoline **6** (2.8 mg, 4 eq) and Endo A-WT (60 μg) in Tris buffer (100 mM, pH 7.4, 100 μL). The reaction was monitored by analytical RP-HPLC. Upon completion of the transglycosylation, the reaction was quenched using 0.1% aq. TFA and purified by RP-HPLC (gradient, 10−40% aq MeCN containing 0.1% FA for 30 min; flow rate, 4.0 mL/min) to give glycopeptides **61** (2.2 mg, 60%) as white solid. ESI-MS: Calcd., M = 3191.34; found (m/z): 798.74 $[M + 4H]^{4+}$, 1064.86 $[M + 3H]^{3+}$, 1596.58 $[M + 2H]^{2+}$. Deconvolution of the ESI-MS: M = 3190.4; RP-HPLC retention time, t_R = 19.2 min (gradient, 5−40% aq MeCN containing 0.1% FA for 30 min; flow rate, 1.0 mL/min).

Synthesis of glycopeptide 62

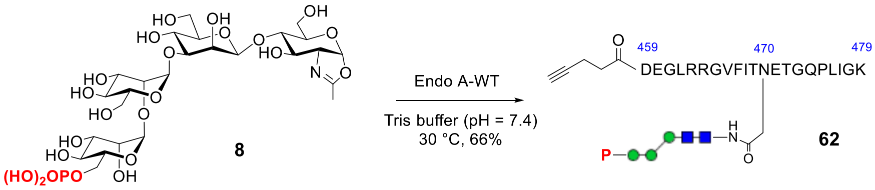

GlcNAc-peptide (3.0 mg, 1.16 μmol) was incubated at 30 °C together with oxazoline **8** (4.2 mg, 5 eq) and Endo A-WT (60 μg) in Tris buffer (100 mM, pH 7.4, 100 μL). The reaction was monitored by analytical RP-HPLC. Upon completion of the transglycosylation, the reaction was quenched using 0.1% aq. TFA and purified by RP-HPLC (gradient, 10−40% aq MeCN containing 0.1% FA for 30 min; flow rate, 4.0 mL/min) to give glycopeptides **62** (2.6 mg, 66%) as white solid. ESI-MS: Calcd., M = 3353.48; found (m/z): 839.27 $[M + 4H]^{4+}$, 1118.50 $[M + 3H]^{3+}$, 1677.12 $[M + 2H]^{2+}$. Deconvolution of the ESI-MS: M = 3352.7; RP-HPLC retention time, t_R = 19.2 min (gradient, 5−40% aq MeCN containing 0.1% FA for 30 min; flow rate, 1.0 mL/min).

Synthesis of glycopeptide 63

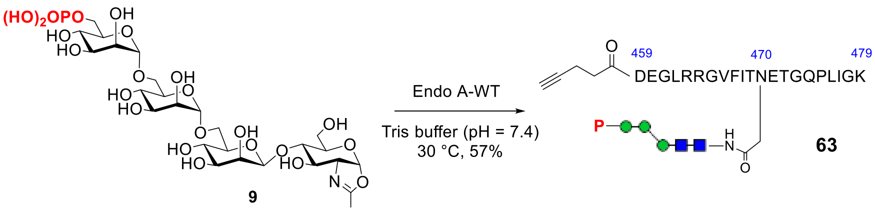

GlcNAc-peptide (3.0 mg, 1.16 μmol) was incubated at 30 °C together with oxazoline **9** (4.8 mg, 6 eq) and Endo A-WT (120 μg) in Tris buffer (100 mM, pH 7.4, 100 μL). The reaction was monitored by

analytical RP-HPLC. Upon completion of the transglycosylation, the reaction was quenched using 0.1% aq. TFA and purified by RP-HPLC (gradient, 10−40% aq MeCN containing 0.1% FA for 30 min; flow rate, 4.0 mL/min) to give glycopeptides **63** (2.2 mg, 57%) as white solid. ESI-MS: Calcd., M = 3353.48; found (m/z): 839.25 $[M + 4H]^{4+}$, 1118.85 $[M + 3H]^{3+}$, 1677.48 $[M + 2H]^{2+}$. Deconvolution of the ESI-MS: M = 3353.1; RP-HPLC retention time, t_R = 19.2 min (gradient, 5−40% aq MeCN containing 0.1% FA for 30 min; flow rate, 1.0 mL/min).

Synthesis of glycopeptide 64

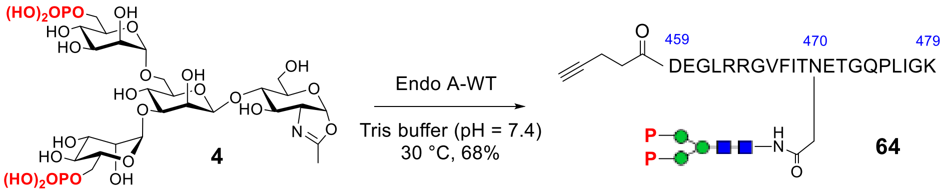

GlcNAc-peptide (3.0 mg, 1.16 μmol) was incubated at 30 °C together with oxazoline **4**[7] (4.9 mg, 5 eq) and Endo A-WT (100 μg) in Tris buffer (100 mM, pH 7.4, 100 μL). The reaction was monitored by analytical RP-HPLC. Upon completion of the transglycosylation, the reaction was quenched using 0.1% aq. TFA and purified by RP-HPLC (gradient, 10−40% aq MeCN containing 0.1% FA for 30 min; flow rate, 4.0 mL/min) to give glycopeptides **64** (2.7 mg, 68%) as white solid. ESI-MS: Calcd., M = 3433.46; found (m/z): 859.25 $[M + 4H]^{4+}$, 1145.02 $[M + 3H]^{3+}$, 1717.00 $[M + 2H]^{2+}$. Deconvolution of the ESI-MS: M = 3432.9; RP-HPLC retention time, t_R = 20.2 min (gradient, 5−40% aq MeCN containing 0.1% FA for 30 min; flow rate, 1.0 mL/min).

Synthesis of glycopeptide 65

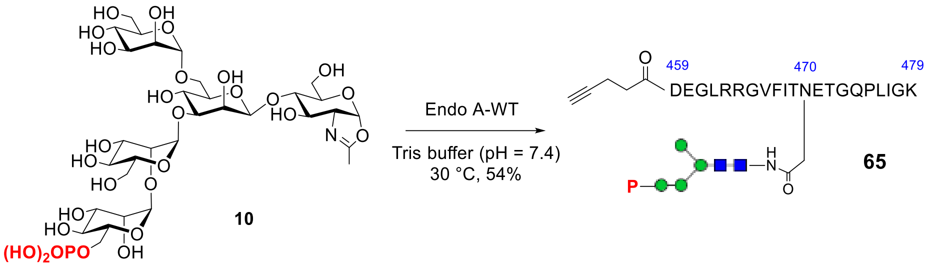

GlcNAc-peptide (3.0 mg, 1.16 μmol) was incubated at 30 °C together with oxazoline **10** (5.4 mg, 5 eq) and Endo A-WT (120 μg) in Tris buffer (100 mM, pH 7.4, 100 μL). The reaction was monitored by analytical RP-HPLC. Upon completion of the transglycosylation, the reaction was quenched using 0.1% aq. TFA and purified by RP-HPLC (gradient, 10−40% aq MeCN containing 0.1% FA for 30 min; flow rate, 4.0 mL/min) to give glycopeptides **65** (2.2 mg, 54%) as white solid. ESI-MS: Calcd., M = 3515.62; found (m/z): 879.89 $[M + 4H]^{4+}$, 1172.78 $[M + 3H]^{3+}$, 1758.44 $[M + 2H]^{2+}$. Deconvolution of the ESI-MS: M = 3515.6; RP-HPLC retention time, t_R = 19.1 min (gradient, 5−40% aq MeCN containing 0.1% FA for 30 min; flow rate, 1.0 mL/min).

Synthesis of glycopeptide 66

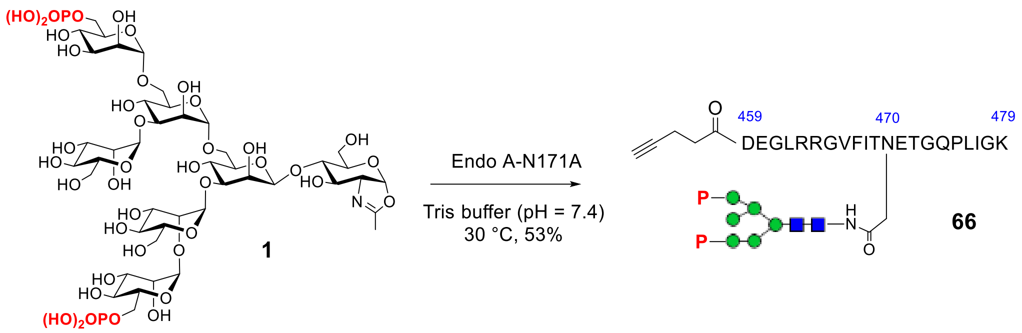

GlcNAc-peptide (2.0 mg, 0.77 µmol) was incubated at 30 °C together with phosphorylated oxazoline **1**[7] (5.2 mg, 5 eq) and Endo A-N171A (180 µg) in Tris buffer (100 mM, pH 7.4, 100 µl). The reaction was monitored by analytical RP-HPLC. Upon completion of the transglycosylation, the reaction was quenched using 0.1% aq. TFA and purified by RP-HPLC to give glycopeptides **66** (1.54 mg, 53%). ESI-MS: Calcd., M = 3919.88; found (m/z): 980.88 $[M + 4H]^{4+}$, 1307.60 $[M + 3H]^{3+}$, 1960.92 $[M + 2H]^{2+}$. Deconvolution of the ESI-MS: M = 3919.5; RP-HPLC retention time, t_R = 19.9 min (gradient, 5−40% aq MeCN containing 0.1% FA for 30 min; flow rate, 1.0 mL/min).

Synthesis of glycoprotein 68

a) Stepwise strategy. RNase B (5.8 mg) was treated with wild-type Endo A (66 µg) in PBS buffer (pH = 7.2, 580 µL) at 37 °C for 1 h. Upon the completion of the reaction as monitored by analytical RP-HPLC, the reaction was purified by preparative HPLC to give the homogeneous GlcNAc-RNase B **67** (4.6 mg, 86%). ESI-MS: Calcd., M = 13886; found (m/z): 1157.93 $[M + 12H]^{12+}$, 1263.28 $[M + 11H]^{11+}$, 1389.51 $[M + 10H]^{10+}$, 1543.78 $[M + 9H]^{9+}$, 1736.63 $[M + 8H]^{8+}$. Deconvolution of the ESI-MS: M = 13886.

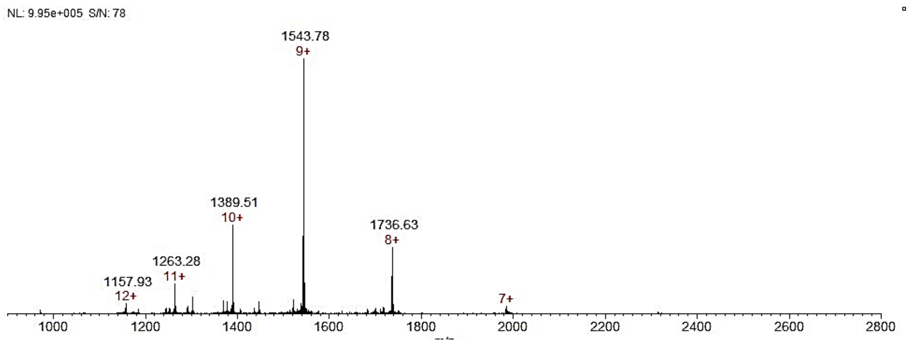

Then to a solution of oxazoline **8** (500 µg, 0.65 µmol, 9 eq) and GlcNAc-RNase B **67** (1.0 mg, 0.072 µmol) in PBS buffer (150 mM, pH 7.2, 20 µL) was added Endo A-WT (200 µg) at 30 °C. The reaction was monitored with analytical RP-HPLC. After 3 h, another potion of oxazoline (280 µg, 5 eq) was added and this procedure was repeated 2 to 3 times until the GlcNAc-RNase B was consumed. Upon completion of the transglycosylation, the reaction was purified by RP-HPLC to give glycoprotein **68** as white solid (0.74 mg, 71%). ESI-MS: Calcd., M = 14655; found (m/z): 1047.87 $[M + 14H]^{14+}$, 1128.39 $[M + 13H]^{13+}$, 1222.13 $[M + 12H]^{12+}$, 1333.33 $[M + 11H]^{11+}$, 1466.57 $[M + 10H]^{10+}$, 1629.37 $[M + 9H]^{9+}$, 1832.80 $[M + 8H]^{8+}$. Deconvolution of the ESI-MS: M = 14656. RP-HPLC retention time, t_R = 17.4 min (gradient, 5−40% aq MeCN containing 0.1% FA for 30 min; flow rate, 1.0 mL/min). Note: in this step, 0.93 mg of solid was obtained after HPLC purification, and ca. ~80% was the desired glycoprotein **68** according to the MS spectrum, which was not separable from the starting material due to the small size of the tetrasaccharide, so the yield was calculated as follows: 930 µg * 80% = 744 µg

(0.051 μmol), 0.051/0.072 = 70.5%. The same method was used in the "one-pot" strategy.

b) "one-pot" strategy. RNase B (1.0 mg) was incubated with Endo A-WT (200 μg) in PBS buffer (pH = 7.2, 20 μL) at 30 °C for 30 min before the addition of oxazoline **8** (308 μg, 6 eq). The reaction was monitored with analytical RP-HPLC, and additional oxazoline was added to push the reaction to completion as described in the stepwise strategy. Preparative RP-HPLC afforded the desired glycoprotein **68** as white solid (0.62 mg, 64%).

"one-pot" glycan remodeling of rhGAA with wild-type Endo A. The commercial Lumizyme (Genzyme, Sanofi) was purified by buffer exchange with PBS (150 mM, pH = 7.2) to remove extra additions before use. The resulting rhGAA (0.95 mg) was incubated with Endo A-WT (100 μg) in PBS buffer (pH = 7.2, 25 μL) at 30 °C for 2 h before the addition of oxazoline **8** (125 μg, 15 eq). After 30 min, another batch of oxazoline (125 μg, 15 eq) was added and this procedure was repeated 6 to 7 times to consume most of the starting material. Upon the completion of the reaction, the resulting mixture was treated with Glutathione Agarose (Thermo Fisher, resin suspended in 200 μL solution) to remove the GST-tagged Endo A-WT, and the cleaved glycans and extra salts were removed by buffer exchange (PBS x 5) to get the remodeled rhGAA (820 μg, 86%).

Transglycosylation of α1,6FucGlcNAc-CD52 with wild-type Endo F3.

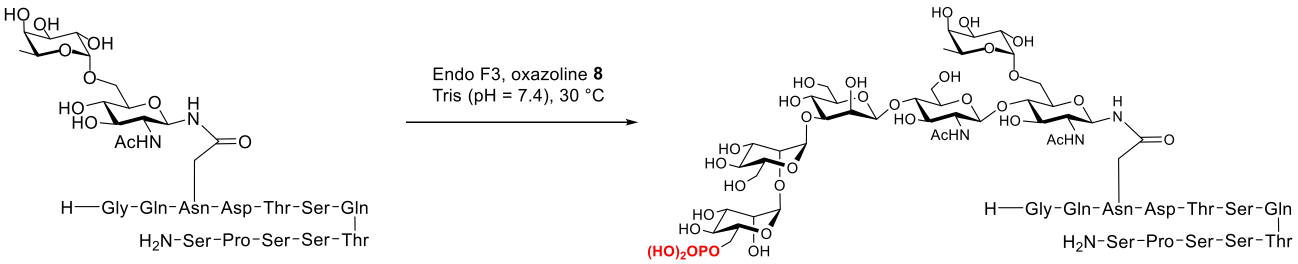

To a solution of oxazoline **8** (200 μg, 0.26 μmol, 4 eq) and Fucα1,6GlcNAc-CD52 (100 μg, 0.072 μmol) in Tris buffer (100 mM, pH 7.4, 5 μL) was added Endo F3-WT (3.0 μg) at 30 °C. The reaction was complete within 30 min. MALDI-TOF: $[M + H]^+$ calcd for $C_{85}H_{142}N_{18}O_{55}P^+$, 2327.12; found, 2327.65; $[M + Na]^+$ calcd for $C_{85}H_{141}N_{18}NaO_{55}P^+$, 2349.10; found, 2349.51; $[M - H + 2Na]^+$ calcd for $C_{85}H_{140}N_{18}Na_2O_{55}P^+$, 2371.08; found, 2371.50; $[M - 2H + 3Na]^+$ calcd for $C_{85}H_{139}N_{18}Na_3O_{55}P^+$, 2393.06; found, 2393.49; $[M - H]^-$ calcd for $C_{85}H_{140}N_{18}O_{55}P^-$, 2325.10; found, 2325.84; $[M - 2H + Na]^-$ calcd for $C_{85}H_{139}N_{18}NaO_{55}P^-$, 2347.08; found, 2348.04.

"One-pot" glycan remodeling of rhGAA with wild-type Endo F3. The commercial Lumizyme (Genzyme, Sanofi) was directly used without pretreatment (the additions such as mannitol were necessary to keep the protein soluble because Endo F3 would cleave most of the complex-type N-glycans). To the commercial mixture (2.4 mg powder, containing ~400 μg rhGAA) in PBS (pH = 7.2, 10 μL) was added Endo F3-WT (40 μg) and oxazoline **8** (100 μg, 30 eq). After 2 h, another batch of oxazoline (100 μg, 30 eq) was added and this procedure was repeated twice to consume most of the starting material. Upon the completion of the reaction, the resulting mixture was treated with Histrap

column (GE Healthcare, 1 mL) to remove the His-tagged Endo F3-WT, and the cleaved glycans and extra salts were removed by buffer exchange (PBS x 5) to get the remodeled rhGAA (282 μg, 71%).

Surface Plasmon Resonance (SPR) Measurements. SPR measurements were performed on a Biacore T200 instrument (GE Healthcare). Recombinant human IGF-II R (CI-MPR) was purchased from R&D Systems. Approximately 7000 resonance units (RU) of CI-MPR was immobilized on a CM5 sensor chip in a sodium acetate buffer (25 μg/mL, pH 4.0) at 25 °C, using the amine coupling kit provided by the manufacturer. Mannose 6-phosphate containing glycopeptides or glycoproteins were determined at 25 °C under a flow rate of 10 μL/min. HBS-P+ buffer (10 mM HEPES, 150 mM NaCl, 0.05% surfactant P20, pH 7.4) was used as sample buffer and running buffer. Association was measured for 3 min and dissociation for 10 min at same flow rate (10 μL/min). The surface regeneration was performed by 2 M $MgCl_2$ at a flow rate of 10 μL/min for 60 s. Synthetic glycopeptide and glycoprotein analytes flowed over an immobilized chip with 2-fold serial dilution of the highest concentration of 4 μM (for glycopeptides) or 1 μM (for RNase B) or 250 nM (for rhGAA). Kinetic analyses were performed by global fitting of the binding data to a 1:1 Langmuir binding model using BIAcore T200 evaluation software.

rhGAA enzyme activity assay. The enzyme activity was assayed by using the substrate 4-methylumbelliferyl-α-D-glucopyranoside (4-MUG) (Sigma-Aldrich) which generates fluorescence on digestion.[9, 10] To a solution of 4-MUG (3.0 mM) in acetate buffer (200 μL, containing 0.2 M sodium acetate, 0.4 M potassium chloride, pH 4.3) was added 1.0 μg of rhGAA or remodelled rhGAA (~ 50 nM), and the reaction mixture was incubated at 37 °C. The reaction was monitored at 0 min, 1 min, 2 min, 5 min, 10 min and 15 min by taking 20 μL of aliquot and adding 50 μL of stop buffer (100 mM glycine/NaOH, pH 11). Fluorescence was measured by a spectrophotometer with 355 nm excitation and 460 nm emission, and the error bar was based on three independent assays.

Muscle cell culture, treatment, processing, and analysis

The biological effect of the Endo-A (**69**) and Endo-F3 (**70**) remodeled rhGAA was investigated in an *in vitro* model of Pompe disease.[11, 12] The myoblasts are grown on Matrigel (Corning; 354234)-coated 6-well plates at 33°C in an atmosphere of 5% CO_2 in proliferation medium [20% fetal bovine serum, 10% horse serum, 1% chick embryo extract, recombinant IFN-γ (100 U/mL; Life Technologies), 1× penicillin/streptomycin/L-glutamine in high-glucose (4.5 g/L) DMEM]. When the cells reach 70-80% confluency (3-4 days), the medium is switched to differentiation medium [DMEM containing 2% horse serum, 0.5% chick embryo extract, recombinant human insulin (10 μg/mL, Life Technologies, 12585–014), 1× penicillin/streptomycin/L-glutamine], and the cells are moved to 37 °C in an atmosphere of 5% CO_2. Myotubes begin to form within 3–4 days; they can survive for ~ 8-10 days in culture until they start twitching and detach from the surface.

The commercial rhGAA, Endo-A- or Endo-F3 remodeled rhGAA were added to the myotubes (on day 8 in differentiation medium) at a concentration of 5μM for 24 hours; n=5 independent experiments for each condition. Wild type (WT) immortalized myotubes and untreated KO myotubes were used for comparison. The cells were homogenized on ice in deionized H_2O, sonicated, and centrifuged at 18,000 × *g* at 4°C for 15 min. The supernatants were used for measuring GAA activity and glycogen content.

Measurement of cell-associated GAA activity. The GAA activity in the cells was measured by using 4-methylumbelliferyl-α-D-glucopyranoside (4-MU- α-glucopyranoside; Sigma-Aldrich #M9766) as the fluorogenic GAA substrate as described.[13] Briefly, myotubes grown on Matrigel-coated 6-well plates were rinsed 3 times with PBS, homogenized in distilled water (using a syringe-based homogenization), sonicated, and centrifuged at 18,000 × *g* at 4°C for 15 min. The supernatants were incubated with the substrate in 0.2 M sodium acetate buffer (pH 4.3) in 96-well plates for 1 h at 37°C; the reaction was stopped by adding 0.5 M carbonate buffer (pH 10.5). 4-Methylumbelliferone (4-MU; Sigma-Aldrich #M1381) was used as a standard. Fluorescence was measured on a multi-label plate reader (TECAN, SPARK 10M) at 360 nm excitation/465 nm emission.

Measurement of the glycogen content. The glycogen content was measured as the amount of glucose released after glycogen digestion with Aspergillus Niger amyloglucosidase (Sigma-Aldrich). Samples were denatured at 100°C for 3 min to inactivate endogenous enzymes, centrifuged at 9,000 RPM at room temperature for 3 min, and the supernatants were incubated with/or without 0.175 U/mL amyloglucosidase for 90 min at 37°C in 0.1 M potassium acetate buffer (pH 5.5) and boiled again to stop the reaction. The released glucose was measured using Glucose (Hexokinase) Liquid Reagents (Fisher) as recommended by the manufacturer; the absorbance at 340 nm was read on the Agilent Technologies Cary 60 UV-VIS Spectrophotometer. Protein concentration (BCA assay) was measured and used to normalize the data.

Western blotting. The myotubes were extensively washed, homogenized in RIPA buffer (PBS containing 1% NP-40, 0.5% sodium deoxycholate, 0.1% SDS, and a protease/phosphatase inhibitor cocktail), and centrifuged for 10 min at 18,000 × *g* at 4°C. Protein concentrations of the supernatants were measured using the Bio-Rad Protein Assay (Bio-Rad Laboratories, Inc.), and equal amounts of protein were run on SDS–PAGE gels (Invitrogen, Carlsbad, CA). Separated proteins were electro-transferred onto nitrocellulose membranes (Invitrogen, Carlsbad, CA, USA). Membranes were then treated with blocking buffer (5% nonfat milk), incubated with primary antibodies [rat monoclonal anti-mouse LAMP-1 (Lysosomal-Associated Membrane Protein 1; CD107a #553792) and mouse monoclonal anti-Rab5 (#610724) from BD Pharmingen; rabbit monoclonal anti-human GAA(EPR4716(2) from Abcam] overnight at 4°C, washed, incubated with the appropriate Alexa Fluor-conjugated secondary antibodies and washed again. Horseradish peroxidase (HRP)-chemiluminescence was developed using Azure Radiance plus kit and scanned on imager (Azure Biosystems). Mouse monoclonal anti-GAPDH antibody (Abcam, ab9484) served as loading controls. For immunofluorescence, cultured myotubes were fixed with 2% paraformaldehyde (PFA) for 30 min at room temperature, followed by several washes with PBS and incubation with blocking reagent (MOM kit; Vector Laboratories, Burlingame, CA) for 1 h at room temperature. Myotubes were then incubated with primary antibodies overnight at 4°C, washed with PBS, incubated with secondary antibody for 2 h, and washed again before examination by confocal microscopy (Zeiss LSM 880).

Statistical significance was determined by one way ANOVA test using Prism software. Error bars represent SD. $^{*}P < 0.05$ was considered statistically significant. ** indicate P-values < 0.01; *** indicate P-values < 0.001.

References

[1] Z. Tu, H.-W. Hsieh, C.-M. Tsai, C.-W. Hsu, S.-G. Wang, K.-J. Wu, K.-I. Lin, C.-H. Lin, *Chem. Asian J.* **2013**, *8,* 1536-1550.

[2] D. Crich, W. Li, H. Li, *J. Am. Chem. Soc*., **2004**, *126*, 15081-15086.

[3] H. Ochiai, W. Huang, L.-X. Wang, *J. Am. Chem. Soc*., **2008**, *130*, 13790-13803.

[4] X. Liu, B. L. Stocker, P. H. Seeberger, *J. Am. Chem. Soc.* **2006**, *128*, 3638-3648.

[5] C. F. W. Becker, X. Liu, D. Olschewski, R. Castelli, R. Seidel, P. H. Seeberger, *Angew. Chem. Int. Ed.* **2008**, 47, 8215-8219.

[6] M. M. Mukherjee, N. Basu, R. Ghosh, *RSC Adv*, **2016**, *6*, 45112-45119.

[7] T. Yamaguchi, M. N. Amin, C. Toonstra, L.-X. Wang, *J. Am. Chem. Soc.*, **2016**, *138*, 12472-12485.

[8] L. Zou, R. B. Zheng, T. L. Lowary, *Beilstein J. Org. Chem.* **2012**, *8*, 1219–1226.

[9] J.-Y. Kang, K. K. Shin, H. H. Kim, J.-K. Min, E. S. Ji, J. Y. Kim, Oh. Kwon, D.-B. Oh, *Sci Rep*, **2018**, *8*, 8730.

[10] K. E. Cheikh, I. Basile, A. D. Silva, C. Bernon, P. Cérutti, F. Salgues, M. Perez, M. Maynadier, M. Gary-Bobo, C. Caillaud, M. Cérutti, M. Garcia, A. Morère, *Angew. Chem. Int. Ed*. **2016**, *55*, 14774 –14777.

[11] C. Spampanato, E. Feeney, L. Li, M. Cardone, J. -A. Lim, F. Annunziata, H. Zare, R. Polishchuk, R. Puertollano, G. Parenti, A. Ballabio, N. Raben, *EMBO Mol. Med.* **2013**, *5*, 691-706.

[12] J. -A. Lim, L. Li, O. S. Shirihai, K. M. Trudeau, R. Puertollano, N. Raben, *EMBO Mol. Med.* **2013**, *9*, 353-370.

[13] A. Amalfitano, A. J. McVie-Wylie, H. Hu, T. L. Dawson, N. Raben, P. Plotz, Y. T. Chen, *Proc. Natl. Acad. Sci. USA.* **1999**, *96*, 8861–8866.

NMR Spectra

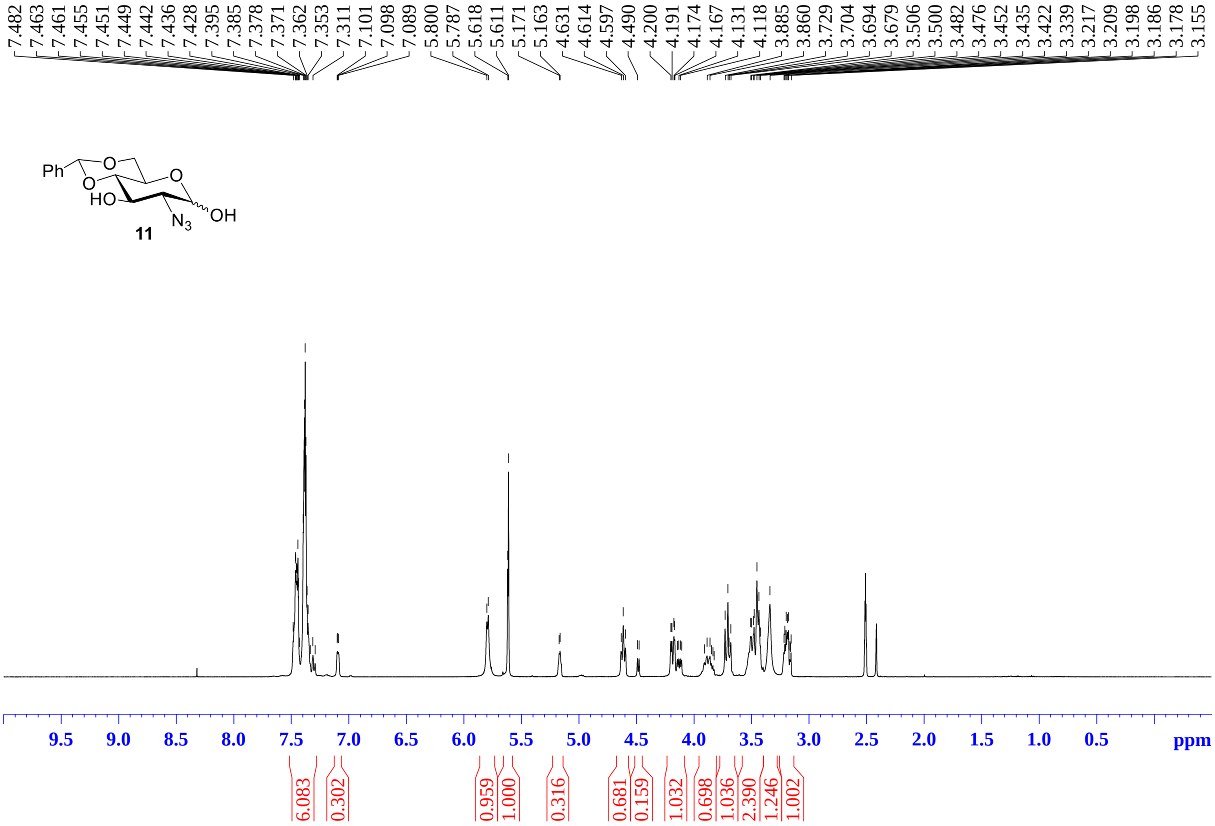

Compound 11: ^{1}H NMR (DMSO-*d*$_6$, 400 MHz)

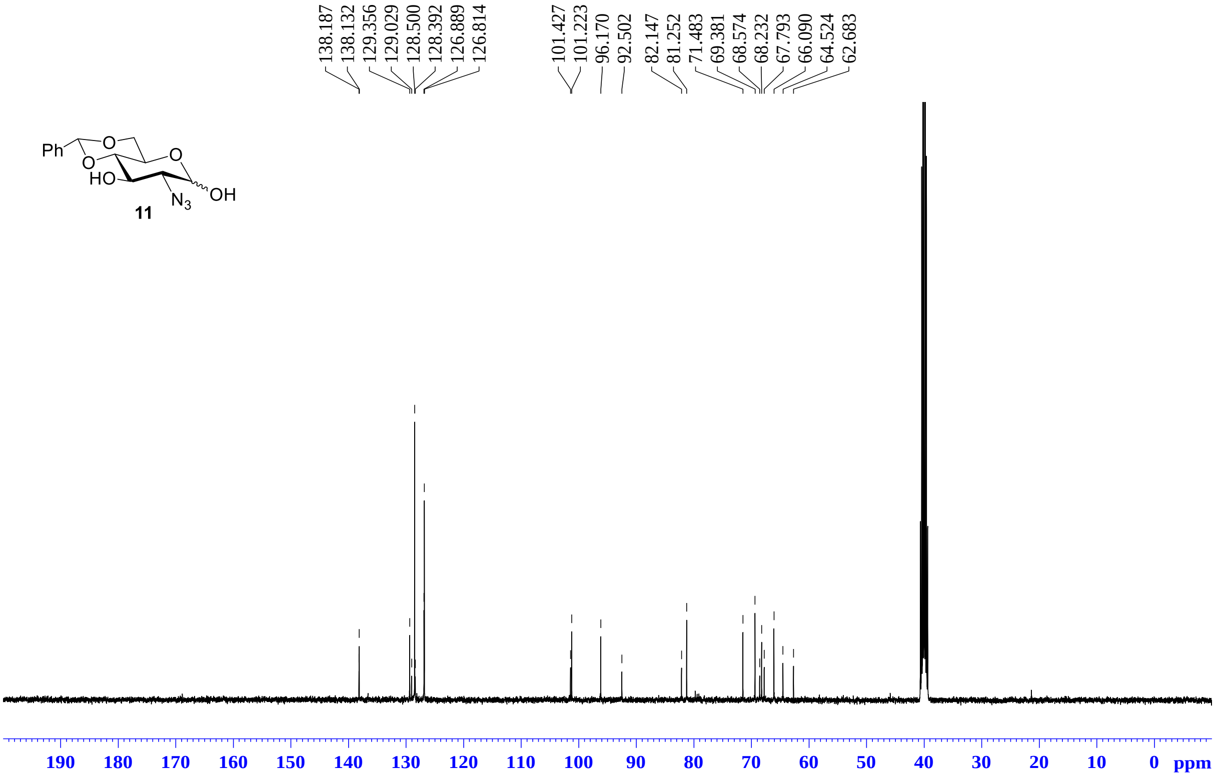

Compound 11: ^{13}C NMR (DMSO-*d*$_6$, 100 MHz)

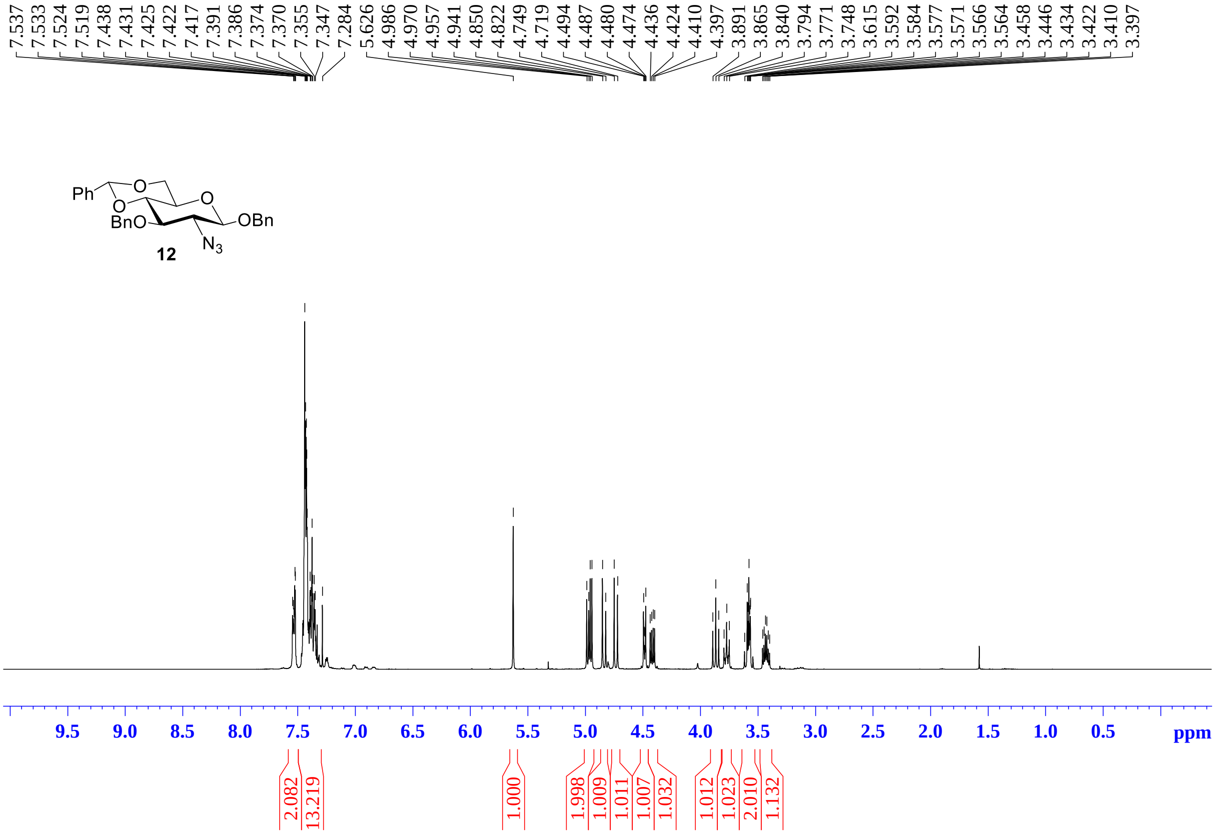

Compound 12: ^{1}H NMR ($CDCl_3$, 400 MHz)

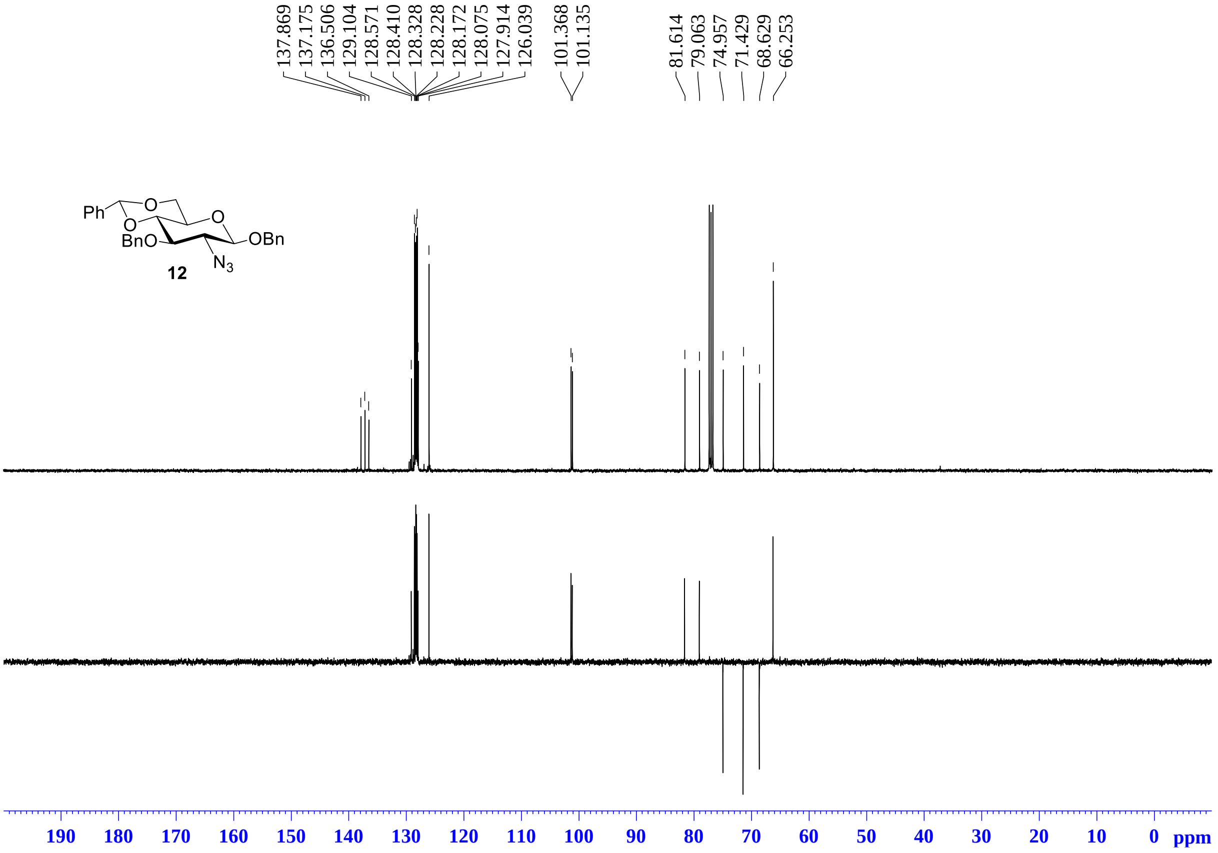

Compound 12: ^{13}C and Dept-135 NMR ($CDCl_3$, 100 MHz)

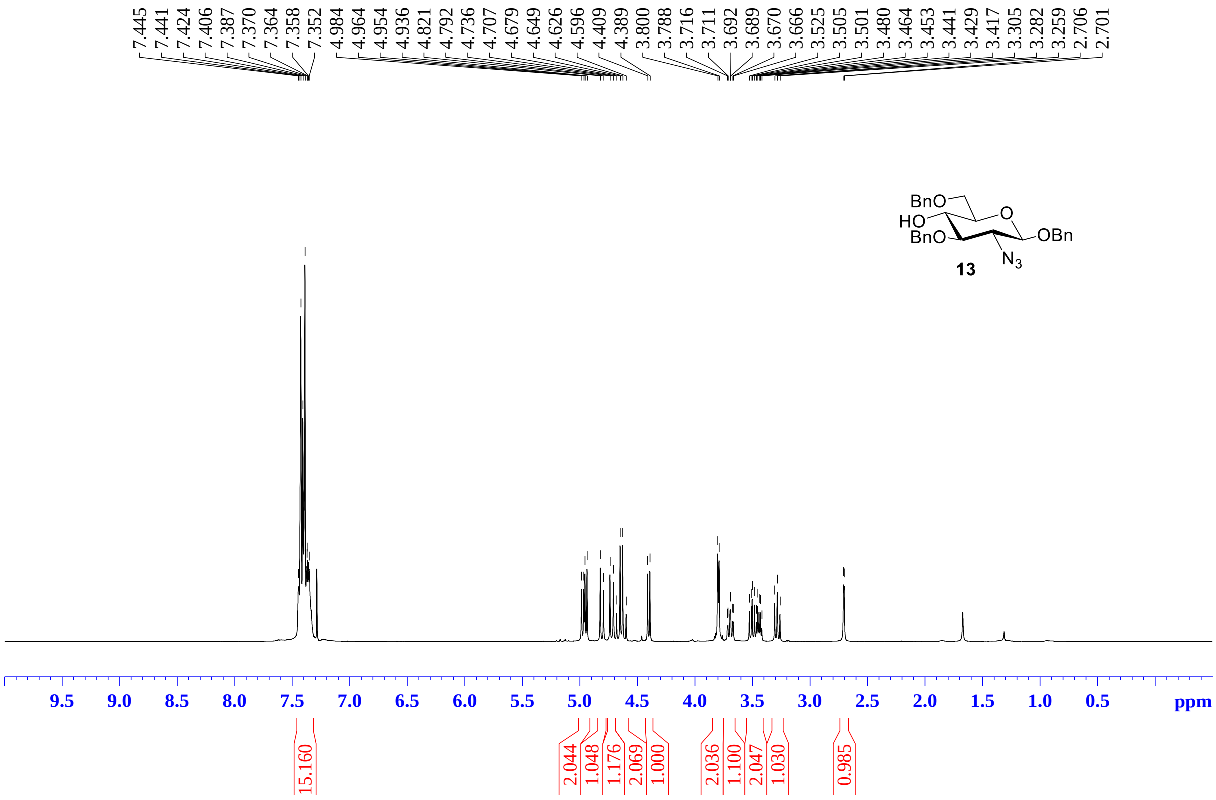

Compound 13: ^{1}H NMR ($CDCl_3$, 400 MHz)

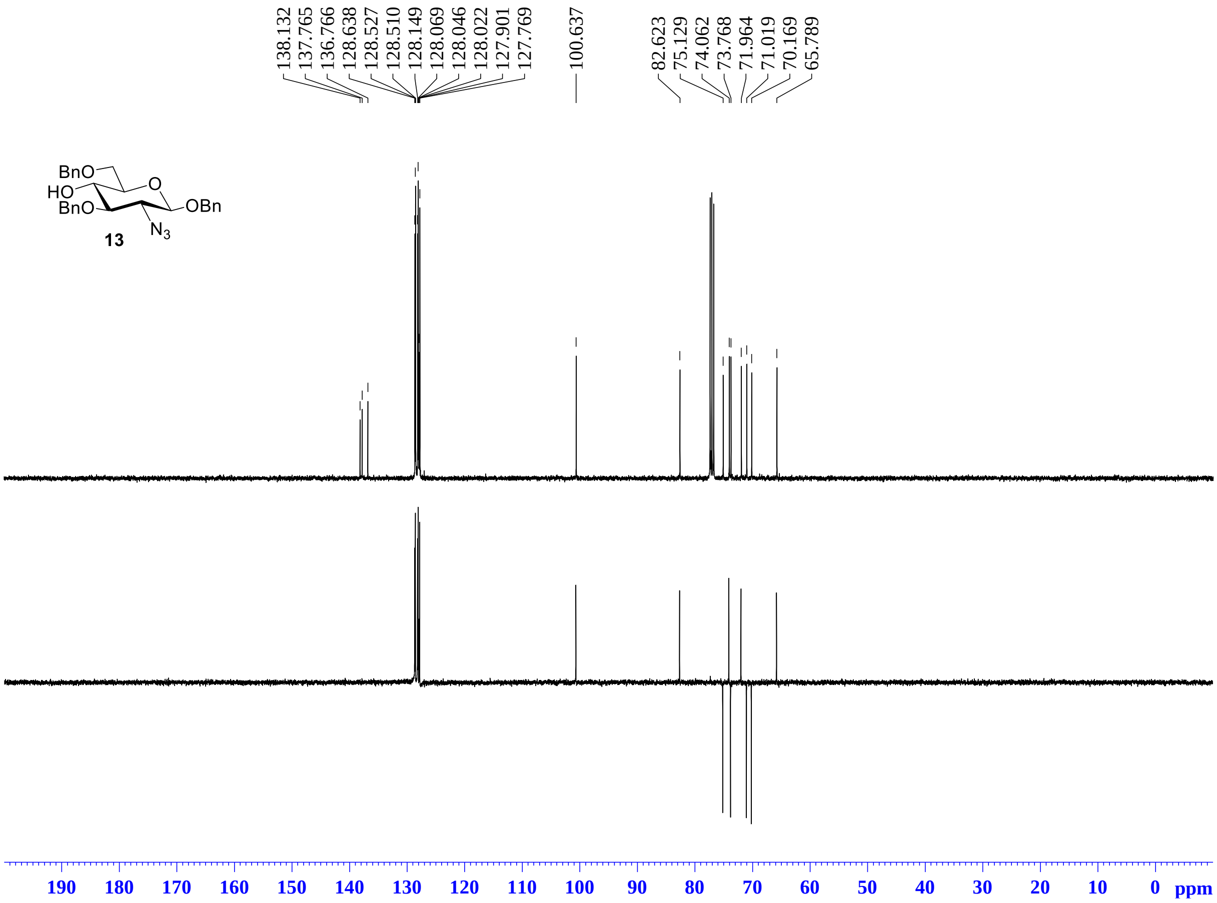

Compound 13: ^{13}C and Dept-135 NMR ($CDCl_3$, 100 MHz)

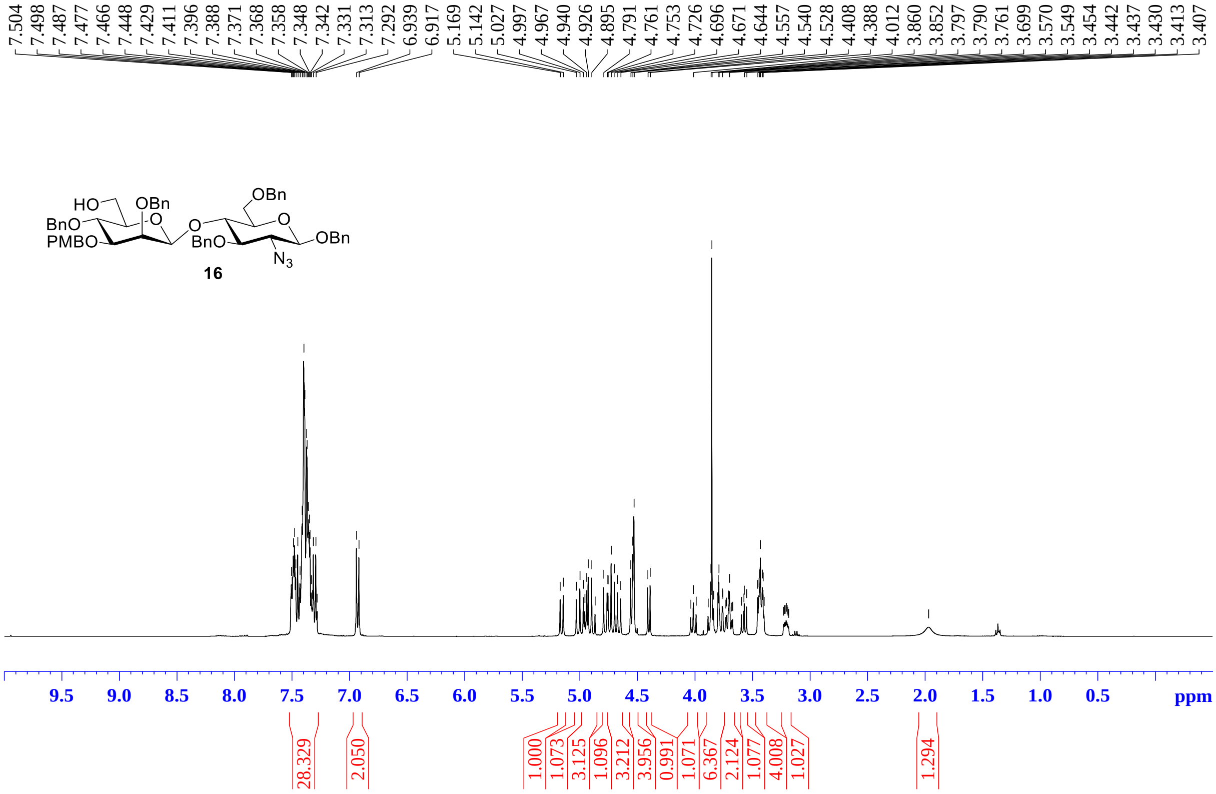

Compound 16: ^{1}H NMR ($CDCl_3$, 400 MHz)

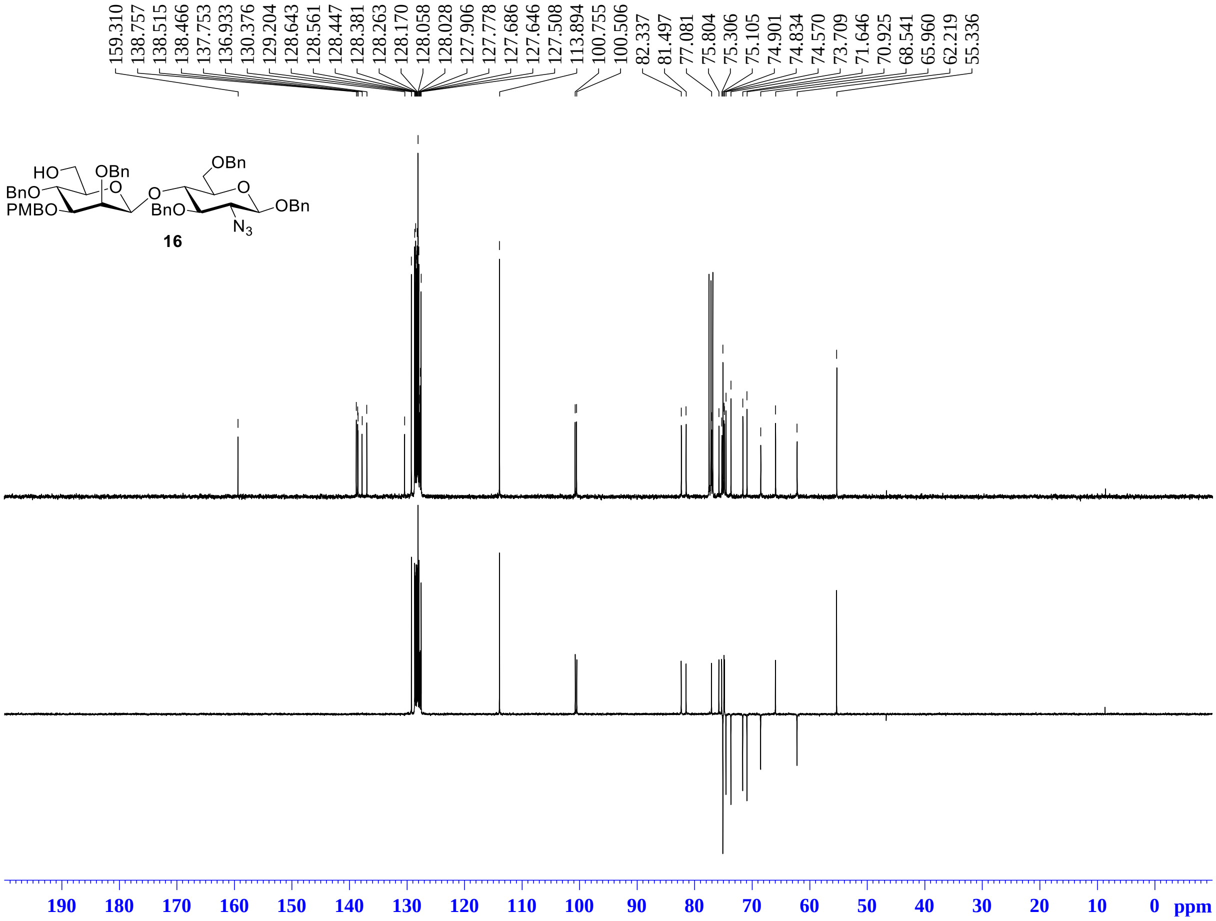

Compound 16: ^{13}C and Dept-135 NMR ($CDCl_3$, 100 MHz)

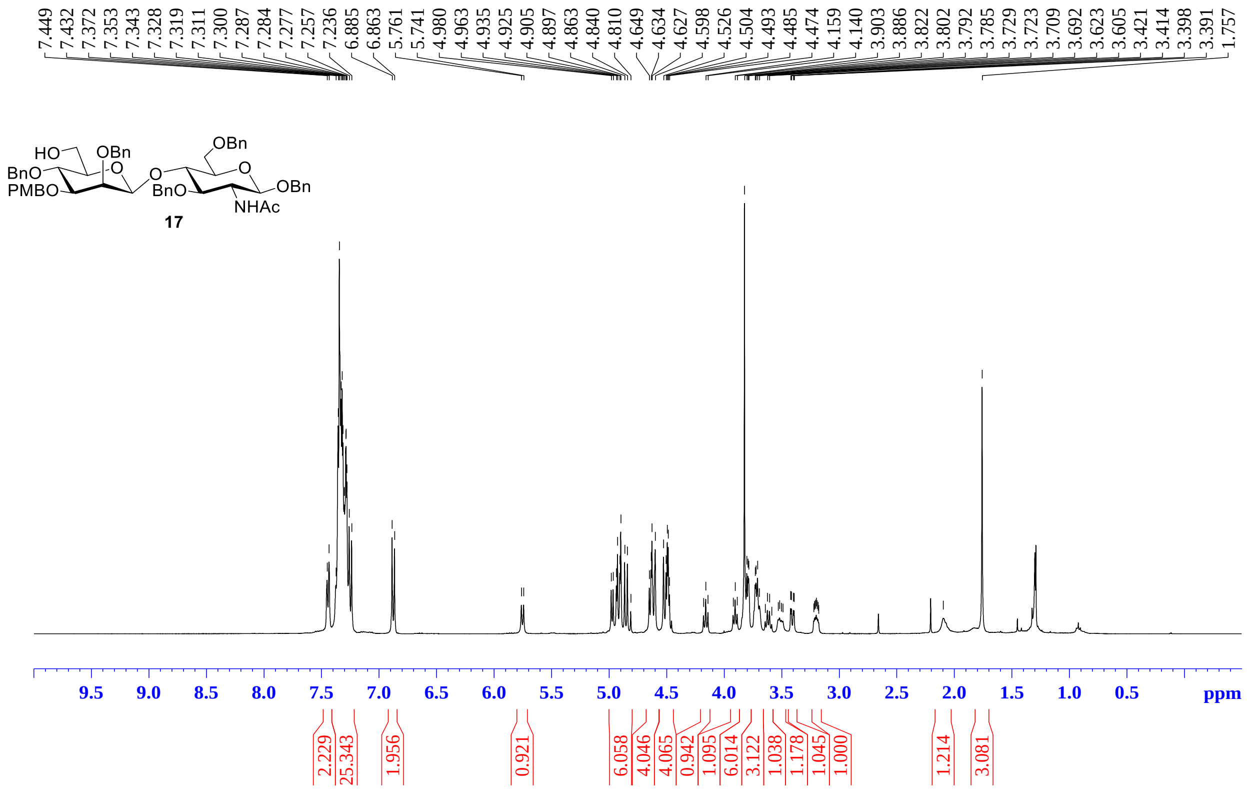

Compound 17: ^{1}H NMR ($CDCl_3$, 400 MHz)

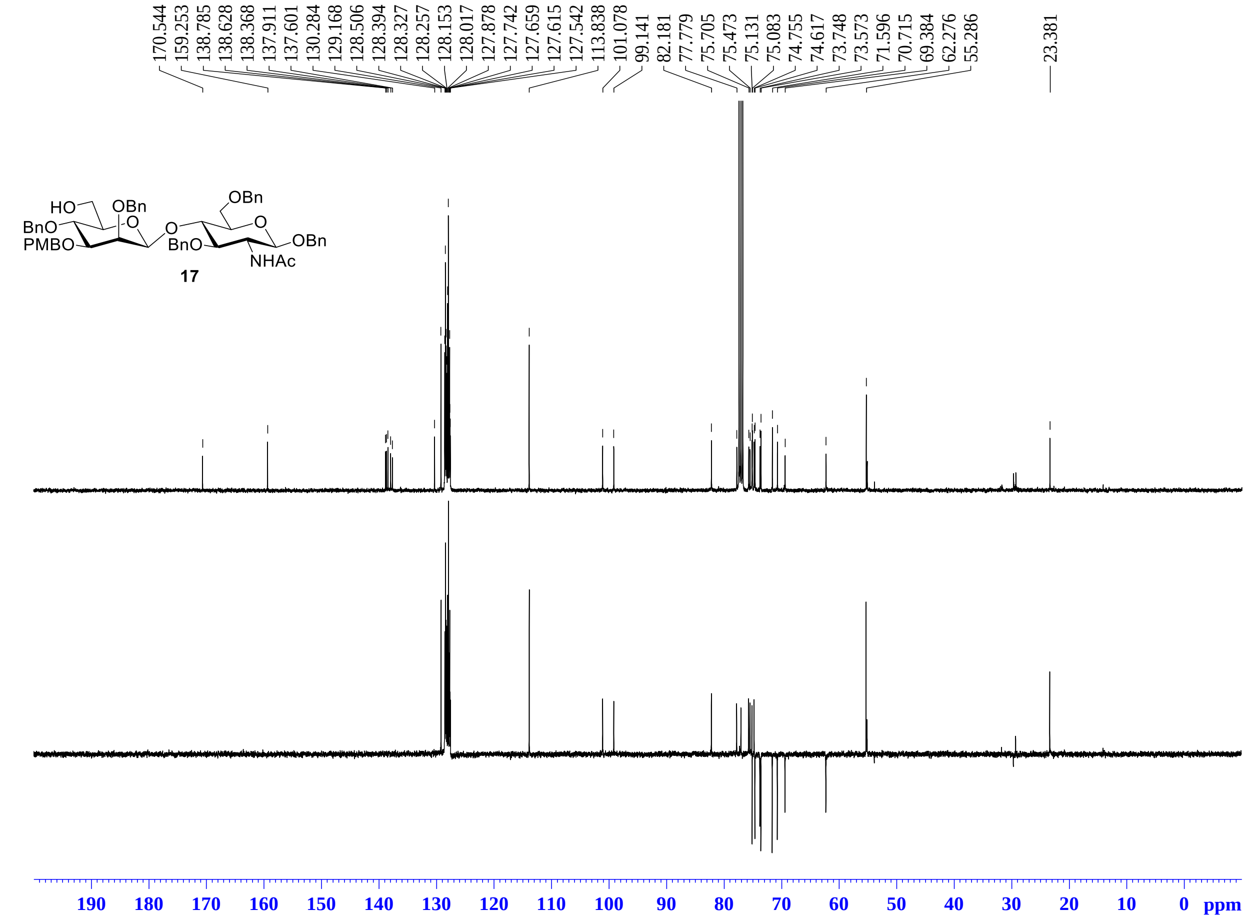

Compound 17: ^{13}C and Dept-135 NMR ($CDCl_3$, 100 MHz)

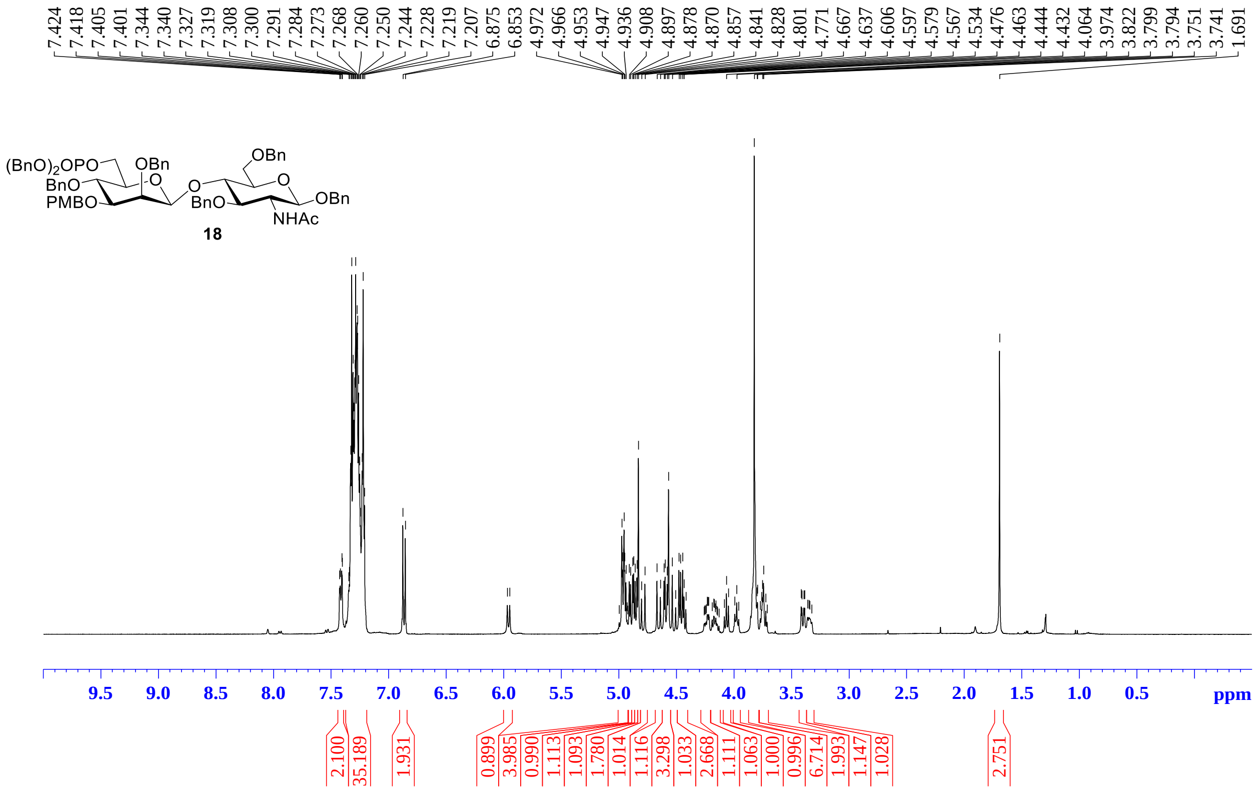

Compound 18: ^{1}H NMR ($CDCl_3$, 400 MHz)

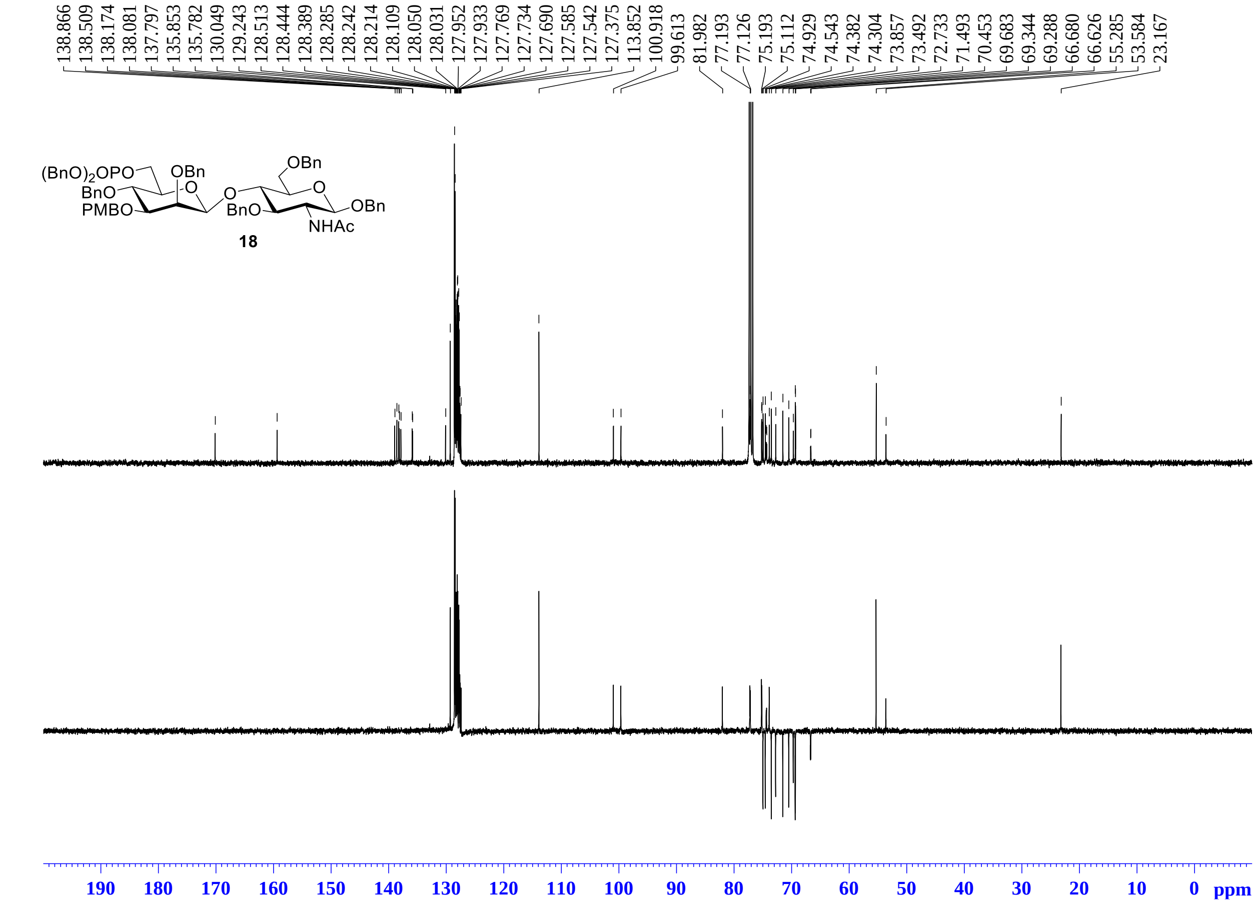

Compound 18: ^{13}C and Dept-135 NMR ($CDCl_3$, 100 MHz)

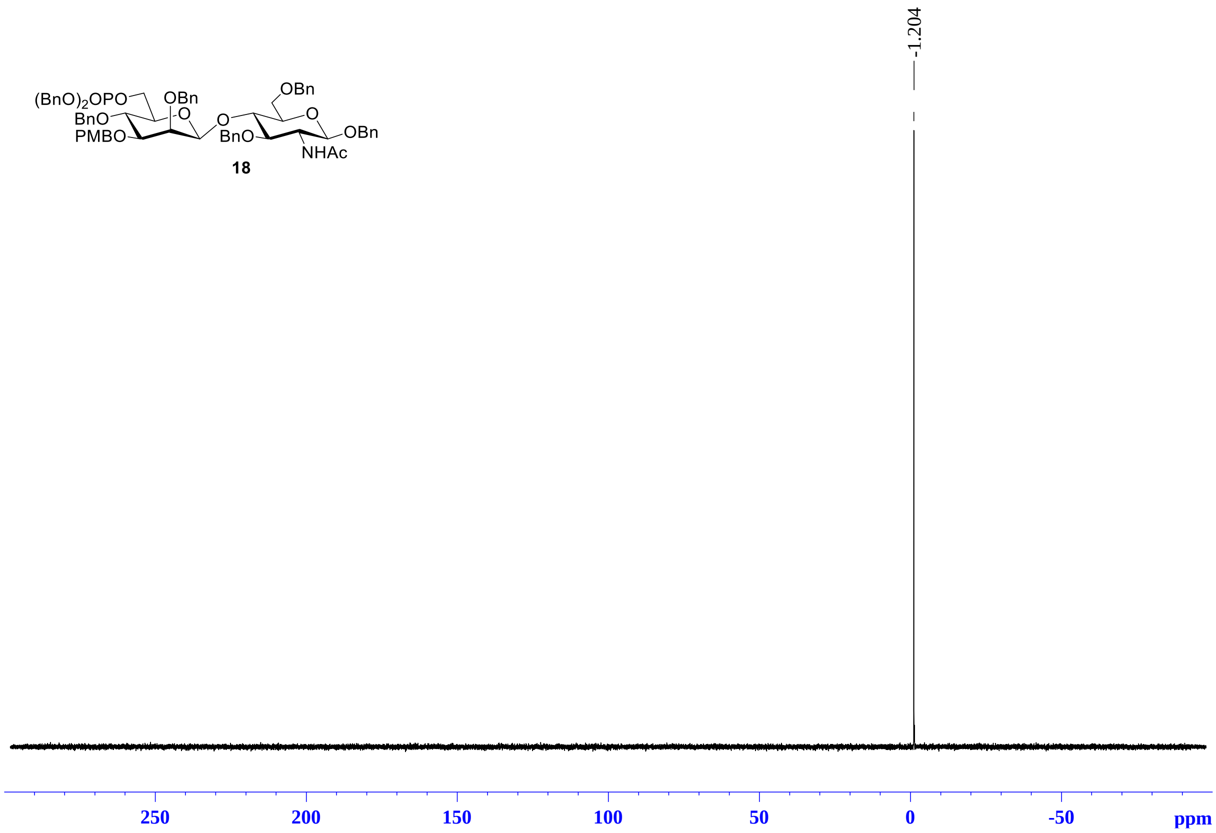

Compound 18: ^{31}P NMR ($CDCl_3$, 146 MHz)

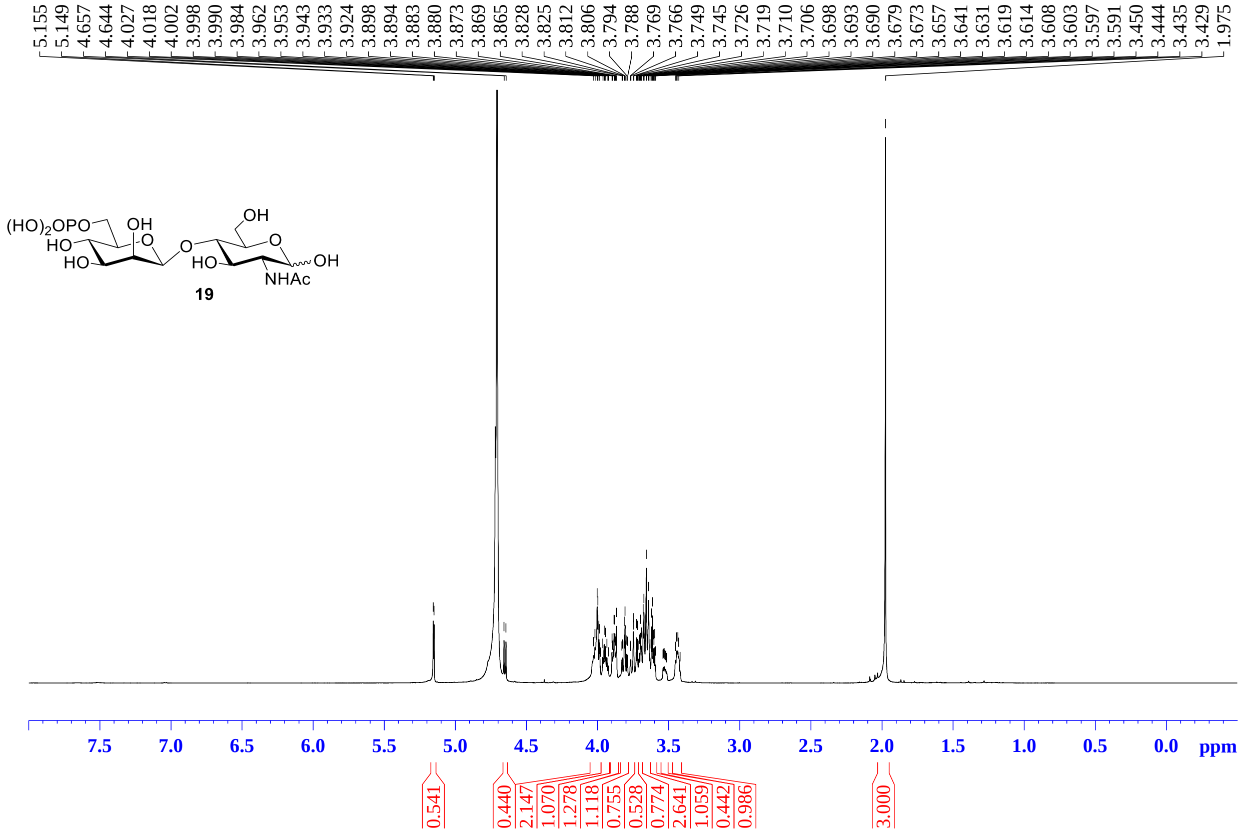

Compound 19: ^{1}H NMR (D_2O, 400 MHz)

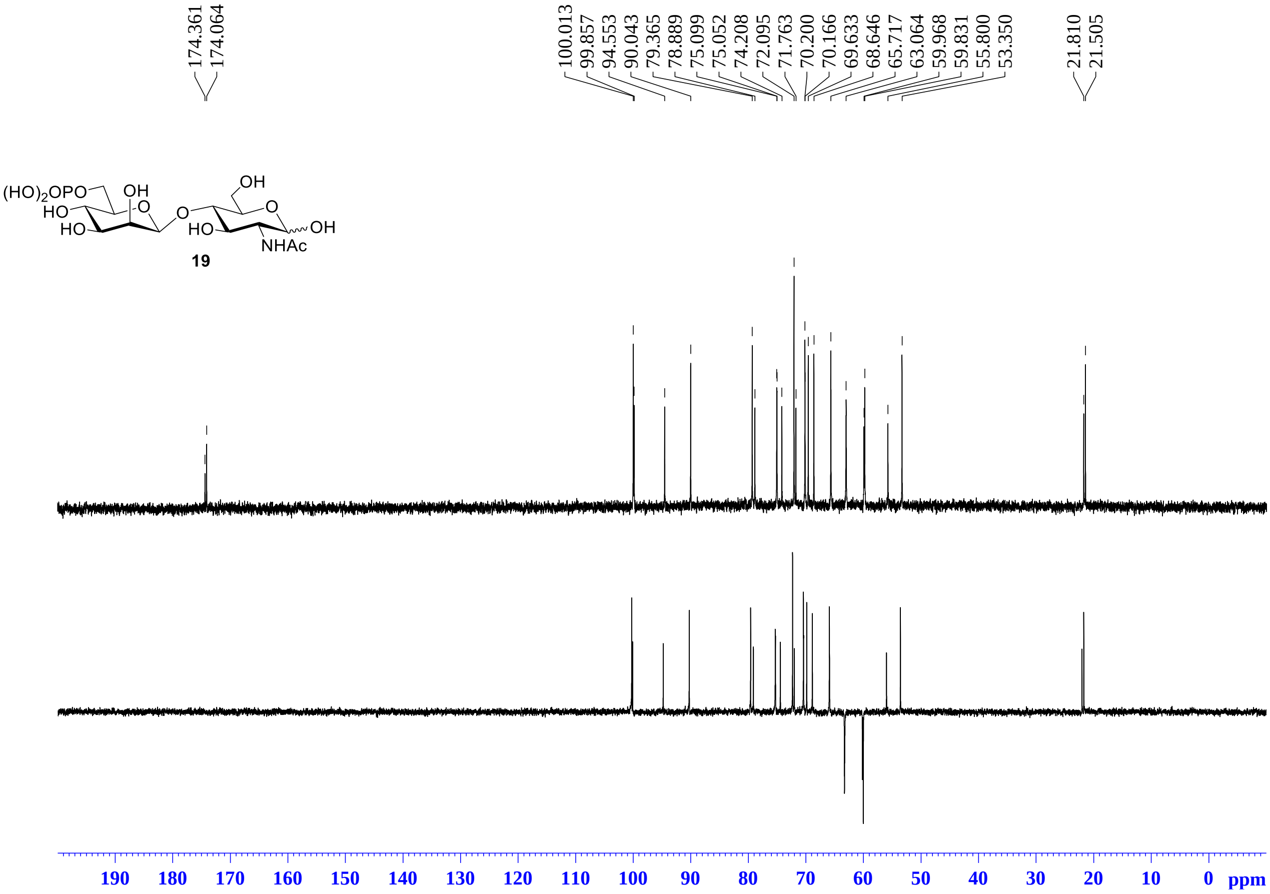

Compound 19: ^{13}C and Dept-135 NMR (D_2O, 100 MHz)

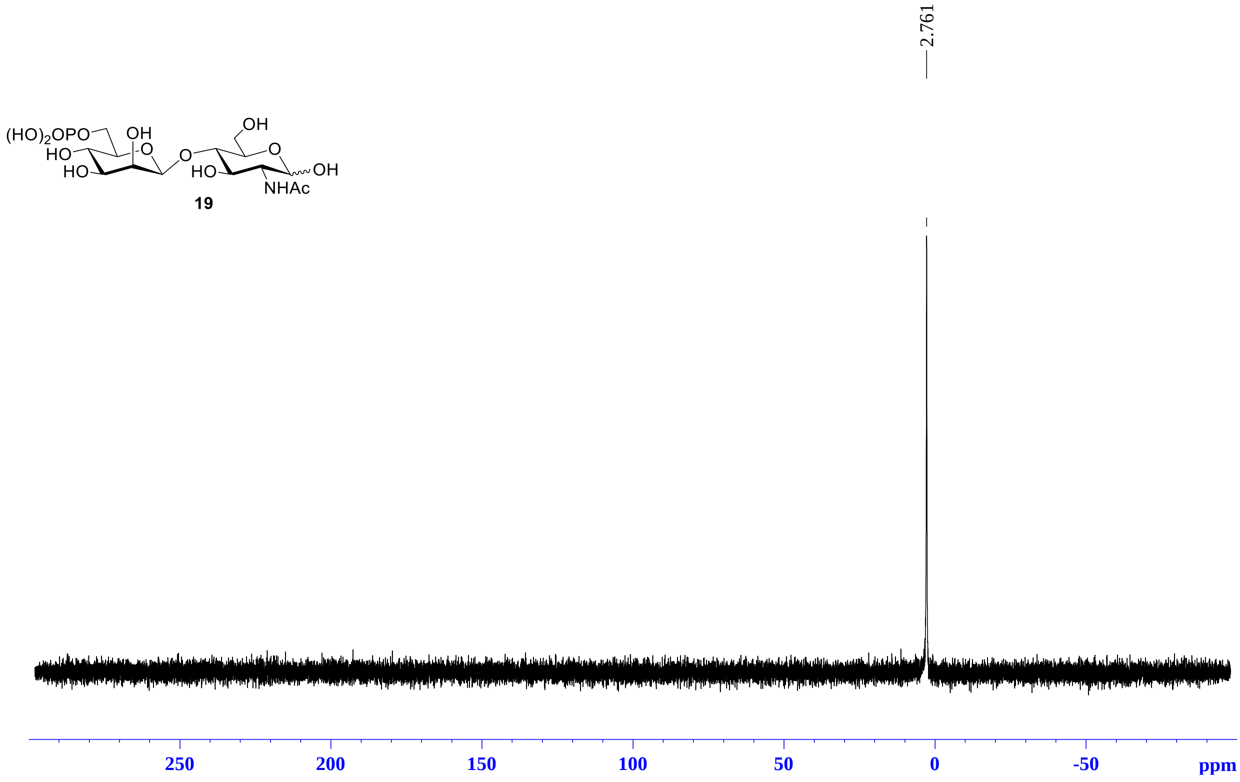

Compound 19: ^{31}P NMR (D_2O, 146 MHz)

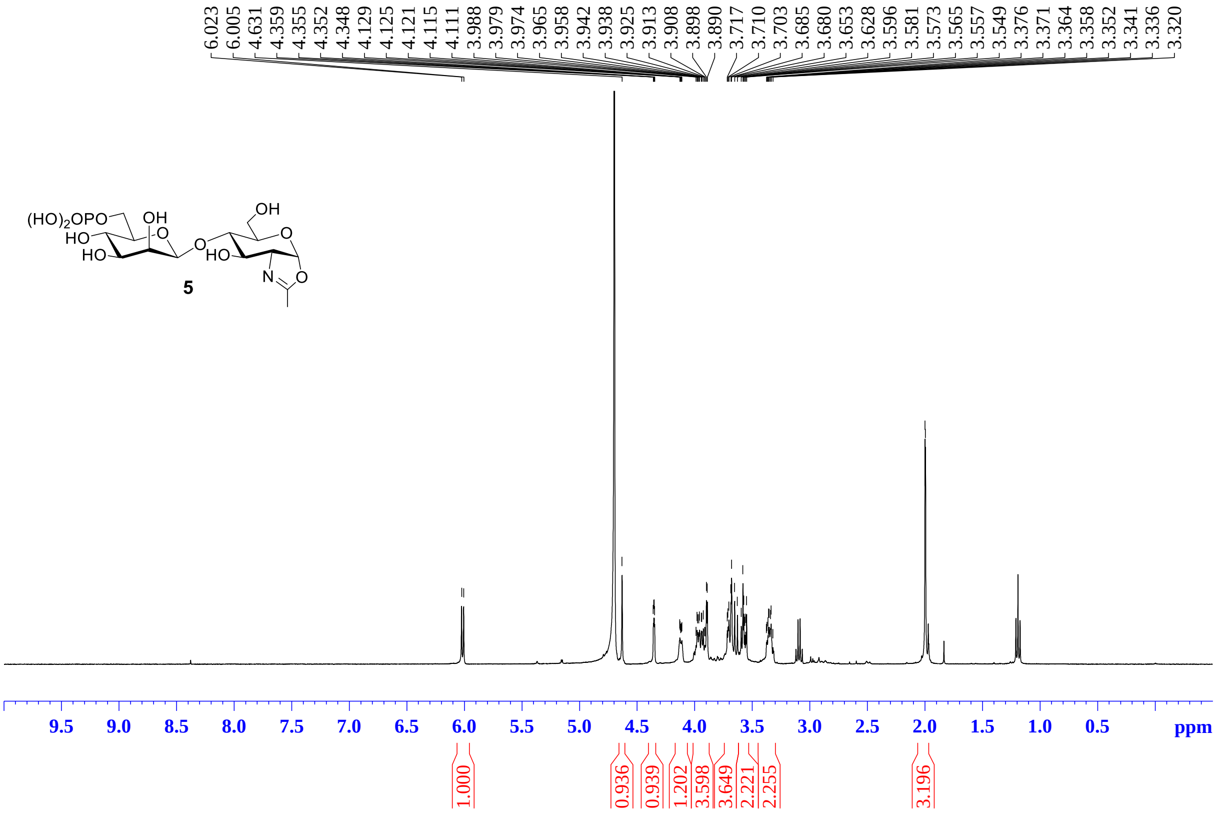

Compound 5: ¹H NMR (D₂O, 400 MHz)

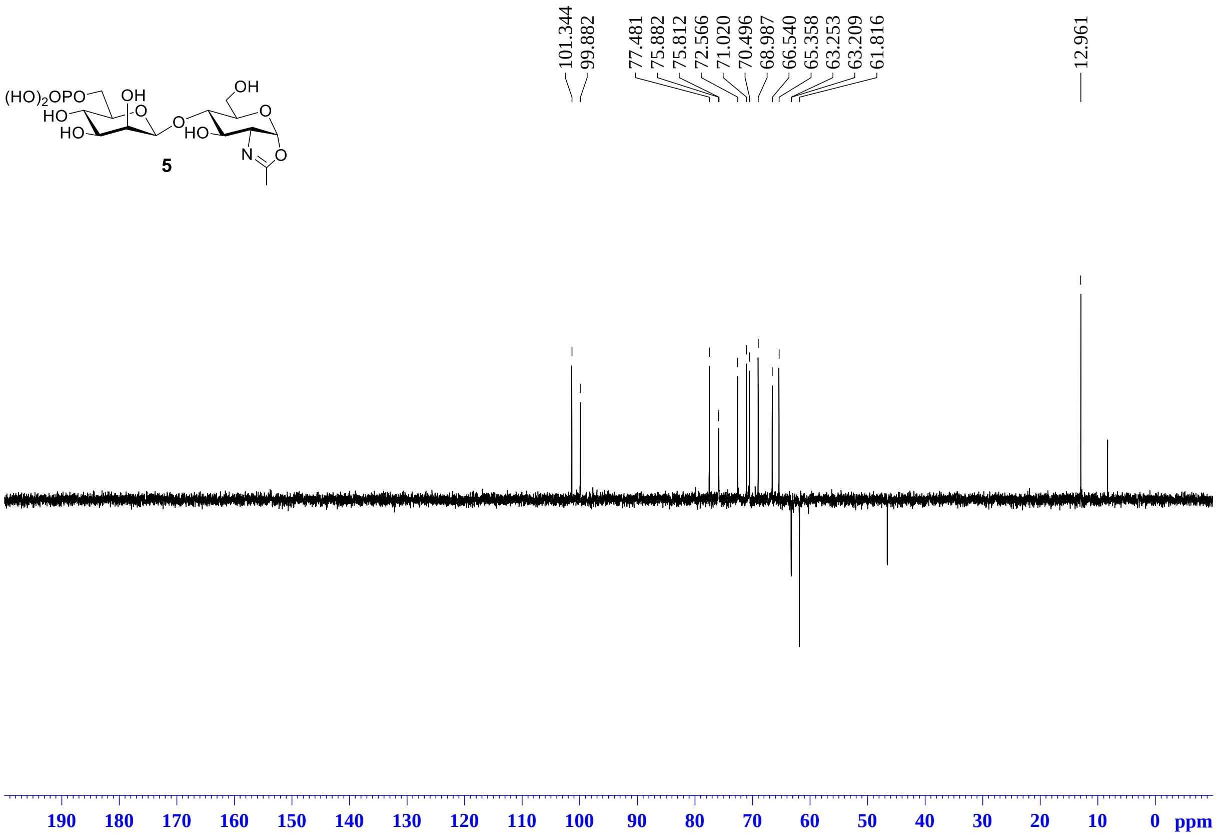

Compound 5: Dept-135 NMR (D₂O, 100 MHz)

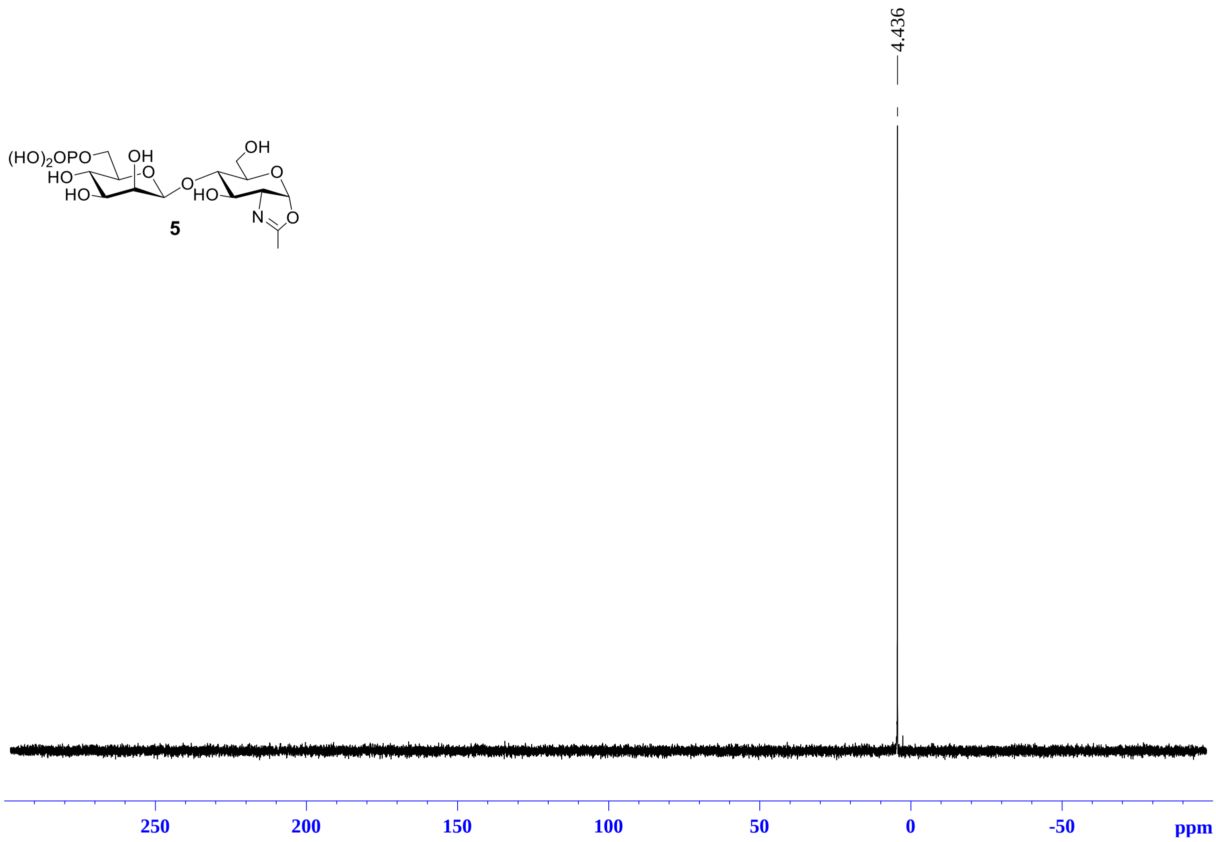

Compound 5: ^{31}P NMR (D_2O, 146 MHz)

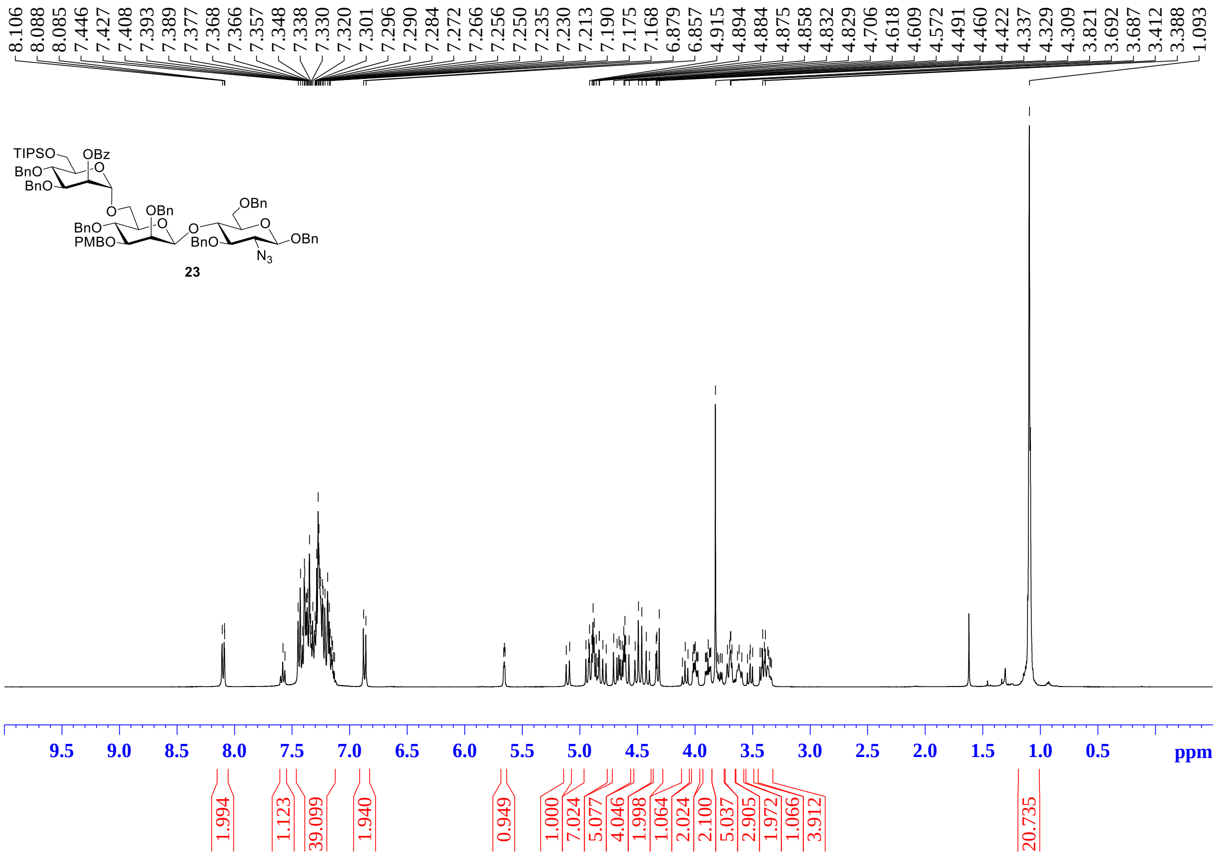

Compound 23: ^{1}H NMR ($CDCl_3$, 400 MHz)

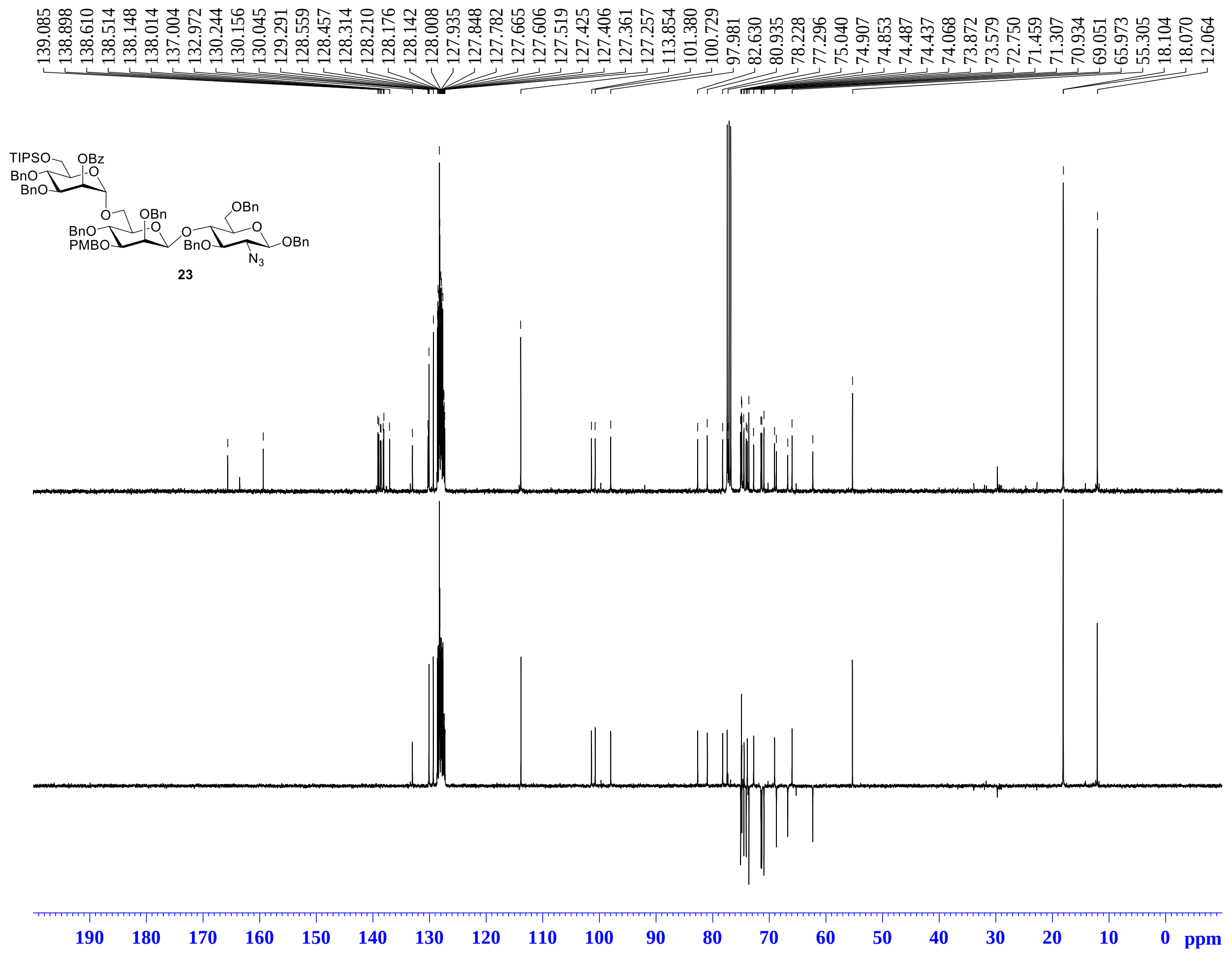

Compound 23: ^{13}C and Dept-135 NMR (CDCl$_3$, 100 MHz)

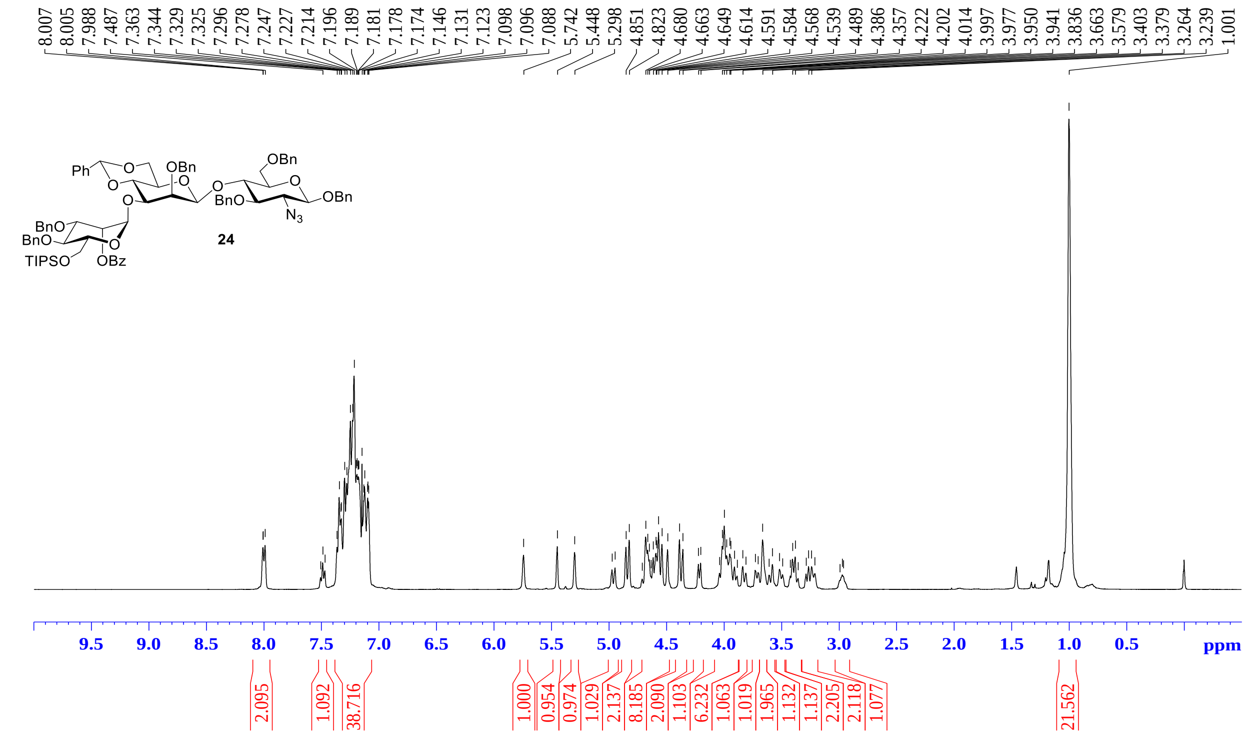

Compound 24: ^{1}H NMR (CDCl$_3$, 400 MHz)

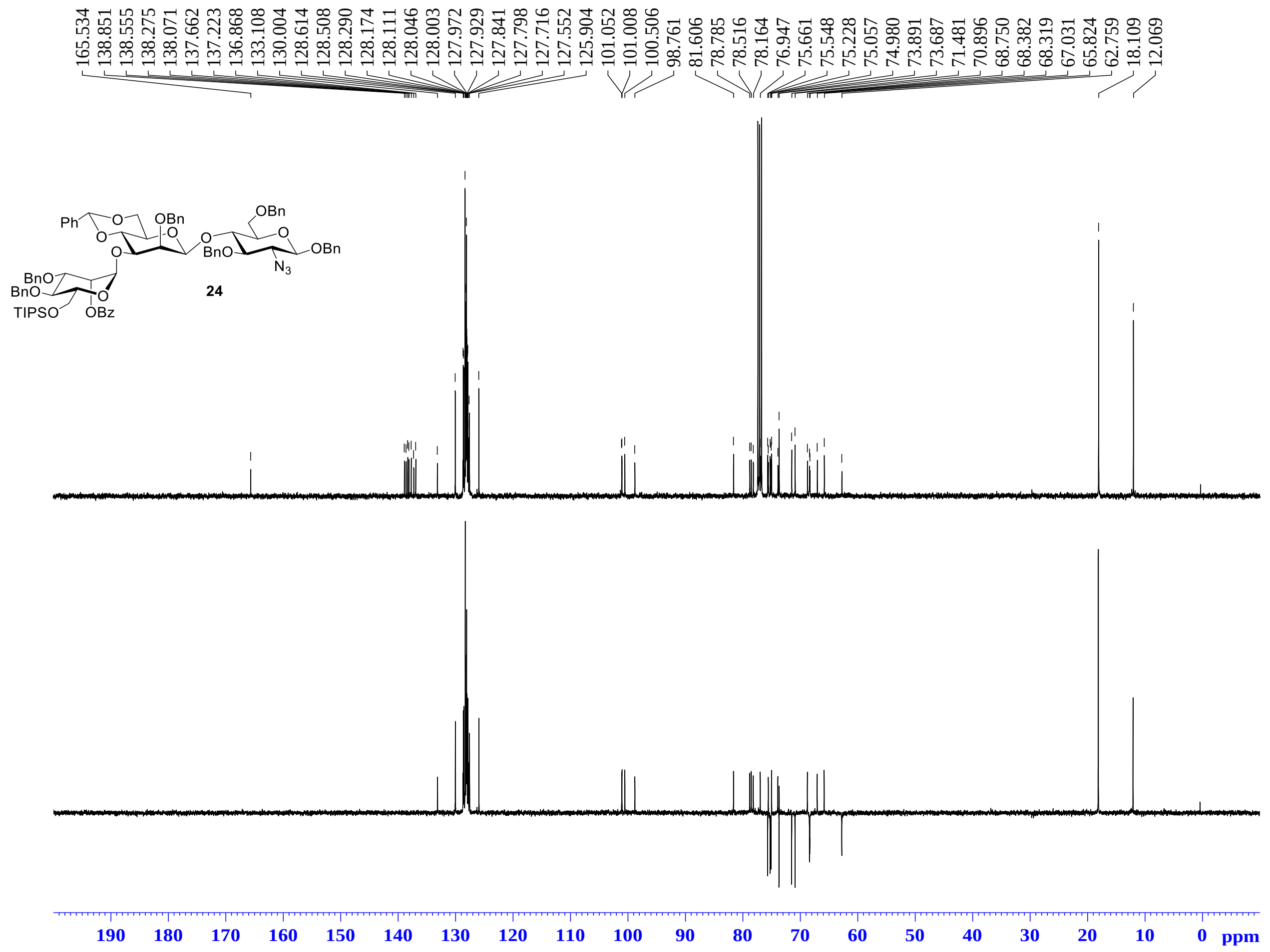

Compound 24: ^{13}C and Dept-135 NMR ($CDCl_3$, 100 MHz)

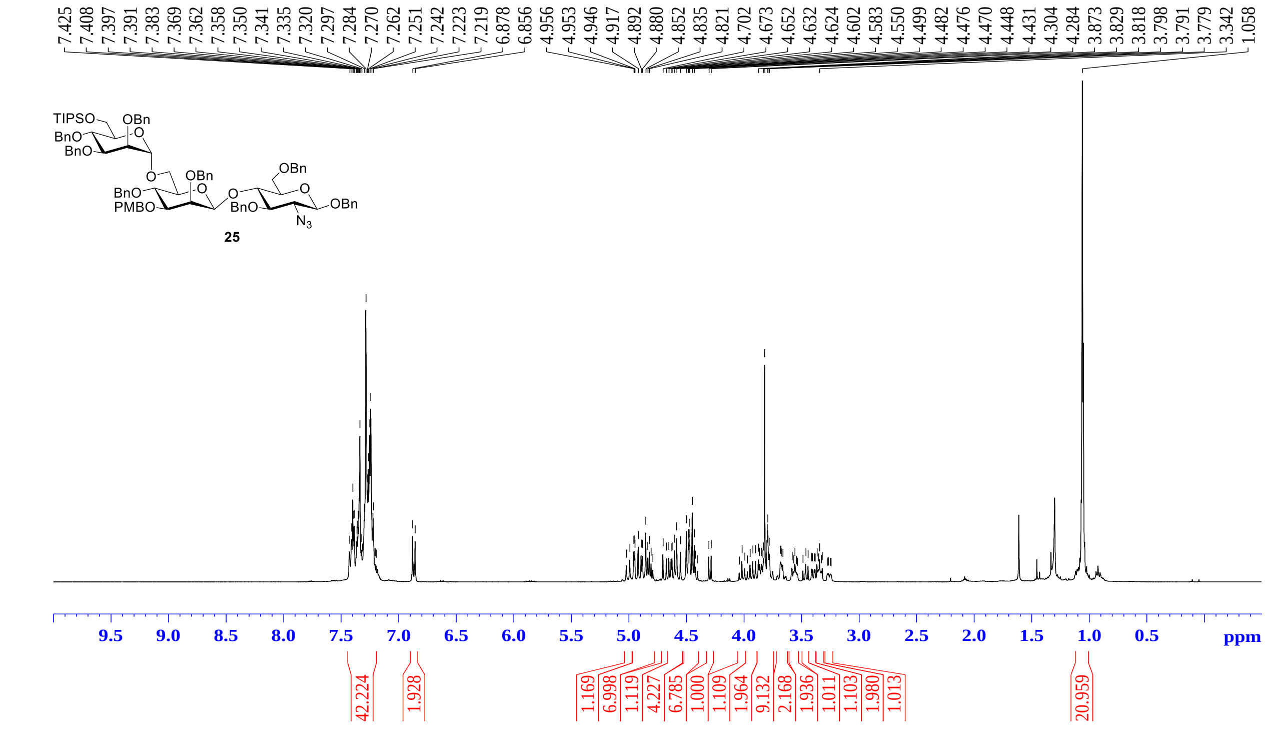

Compound 25: ^{1}H NMR ($CDCl_3$, 400 MHz)

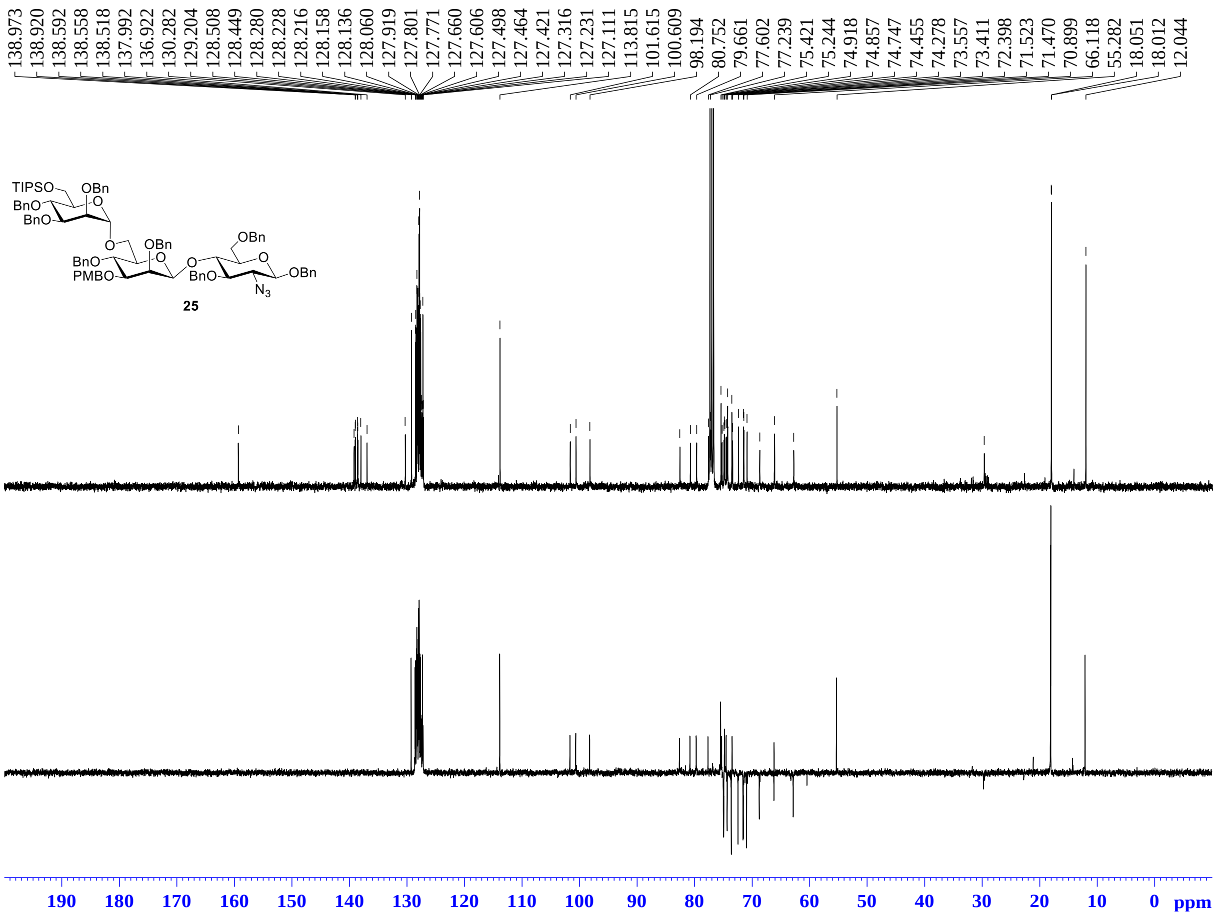

Compound 25: ^{13}C and Dept-135 NMR (CDCl₃, 100 MHz)

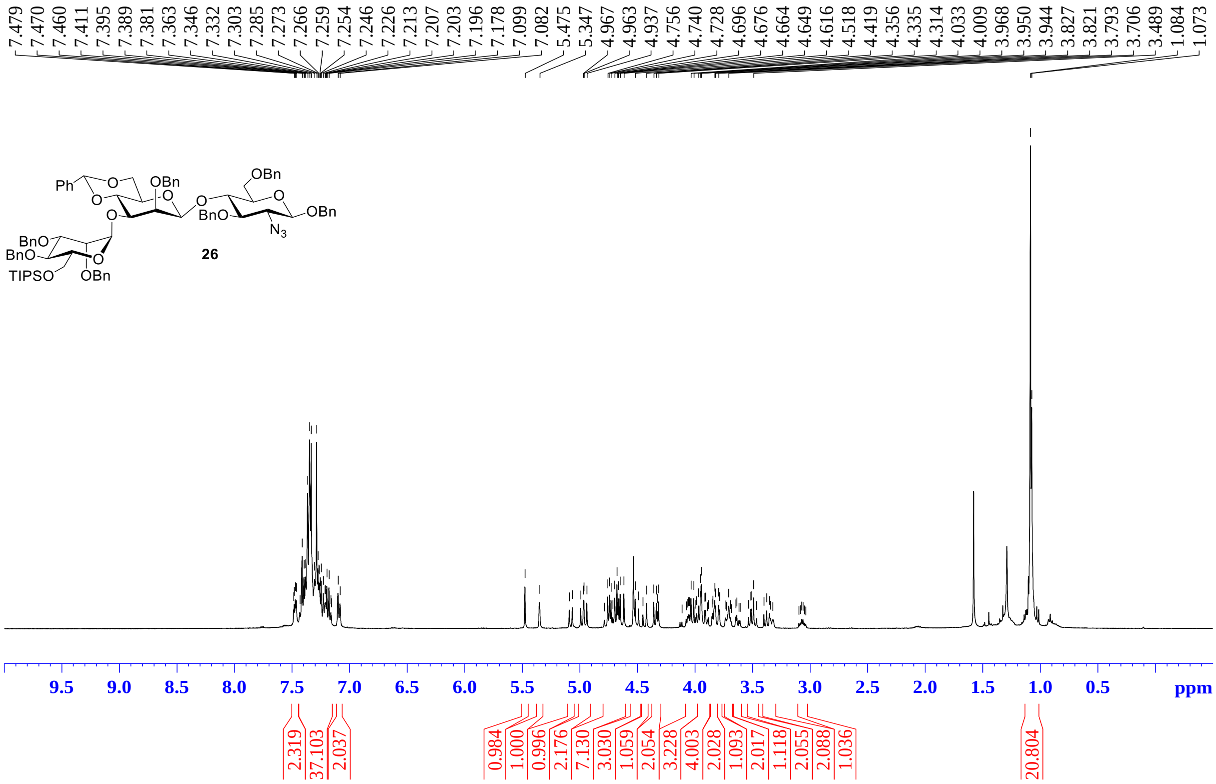

Compound 26: ^{1}H NMR (CDCl₃, 400 MHz)

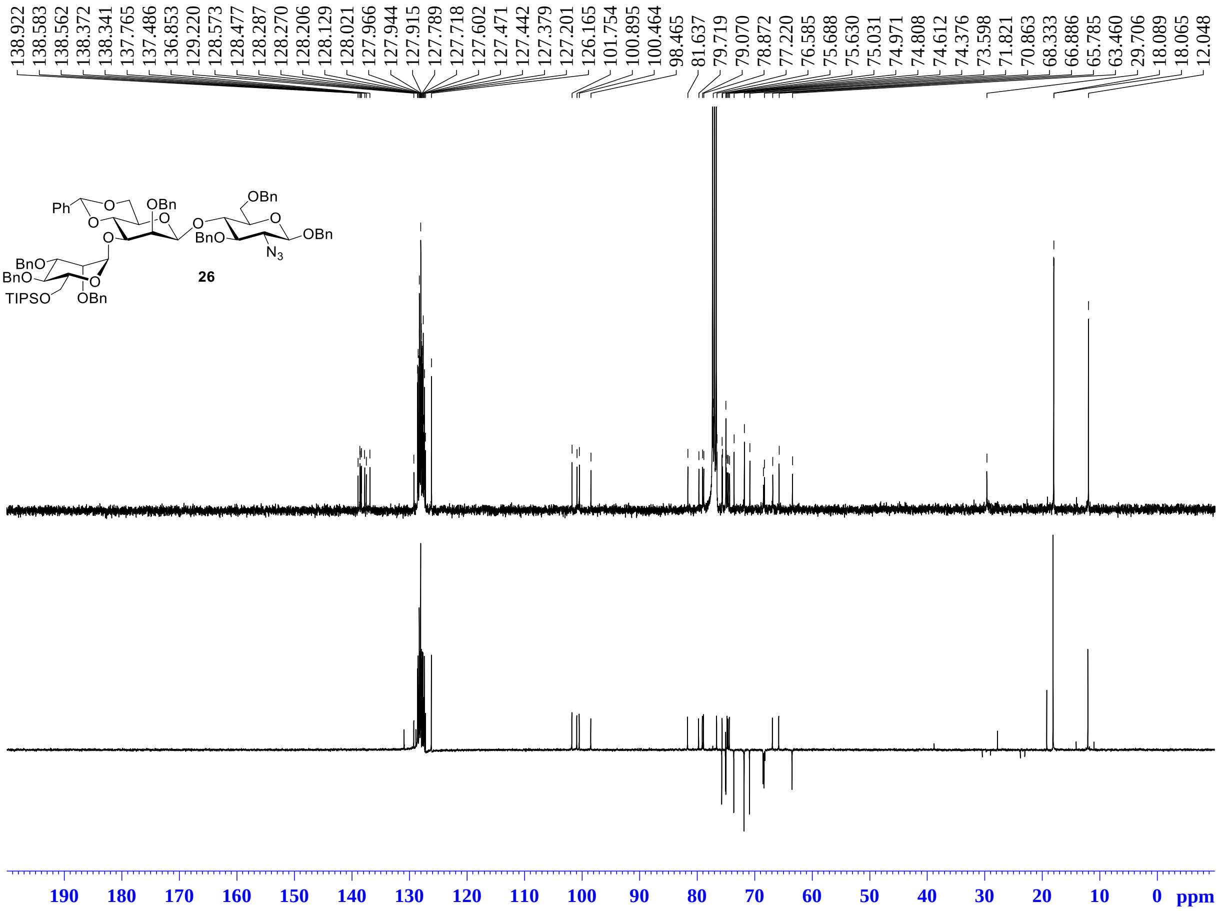

Compound 26: ^{13}C and Dept-135 NMR ($CDCl_3$, 100 MHz)

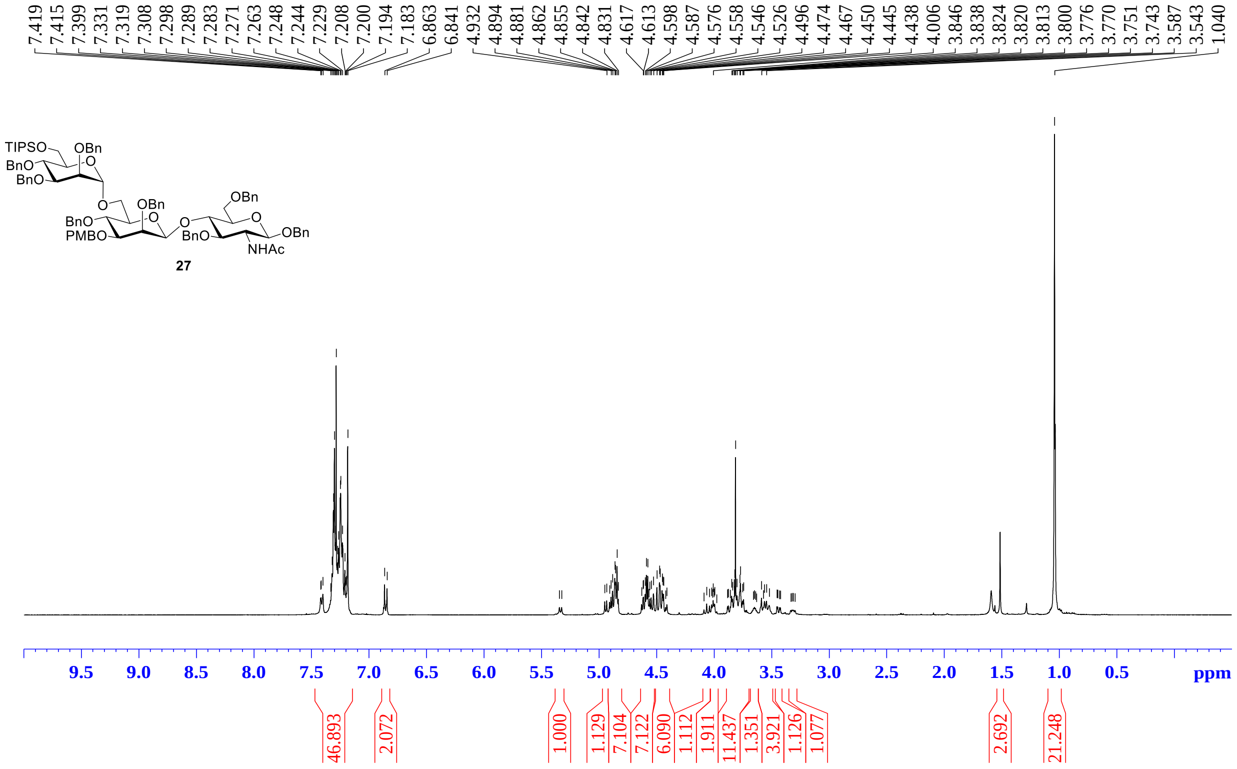

Compound 27: ^{1}H NMR ($CDCl_3$, 400 MHz)

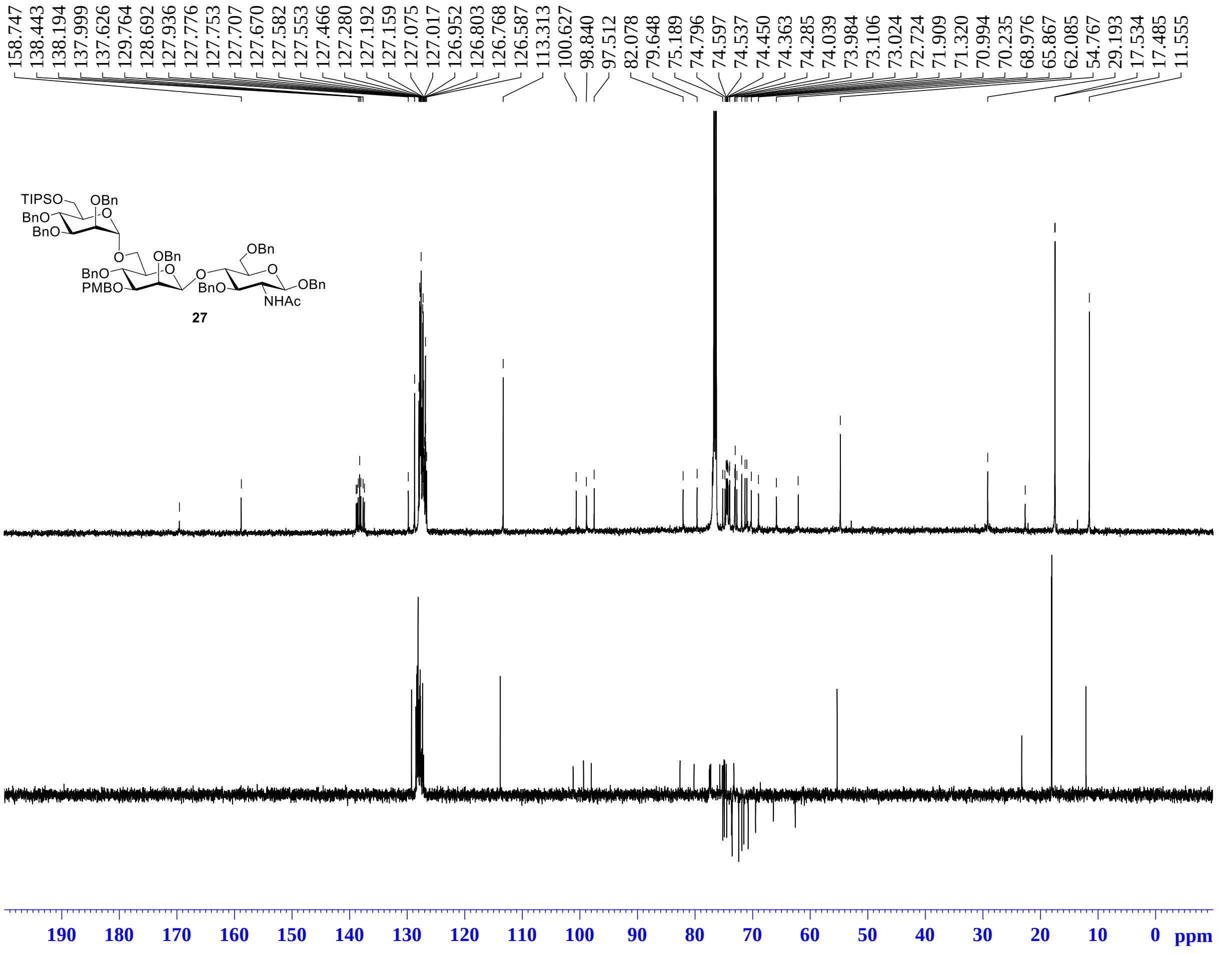

Compound 27: ^{13}C and Dept-135 NMR ($CDCl_3$, 100 MHz)

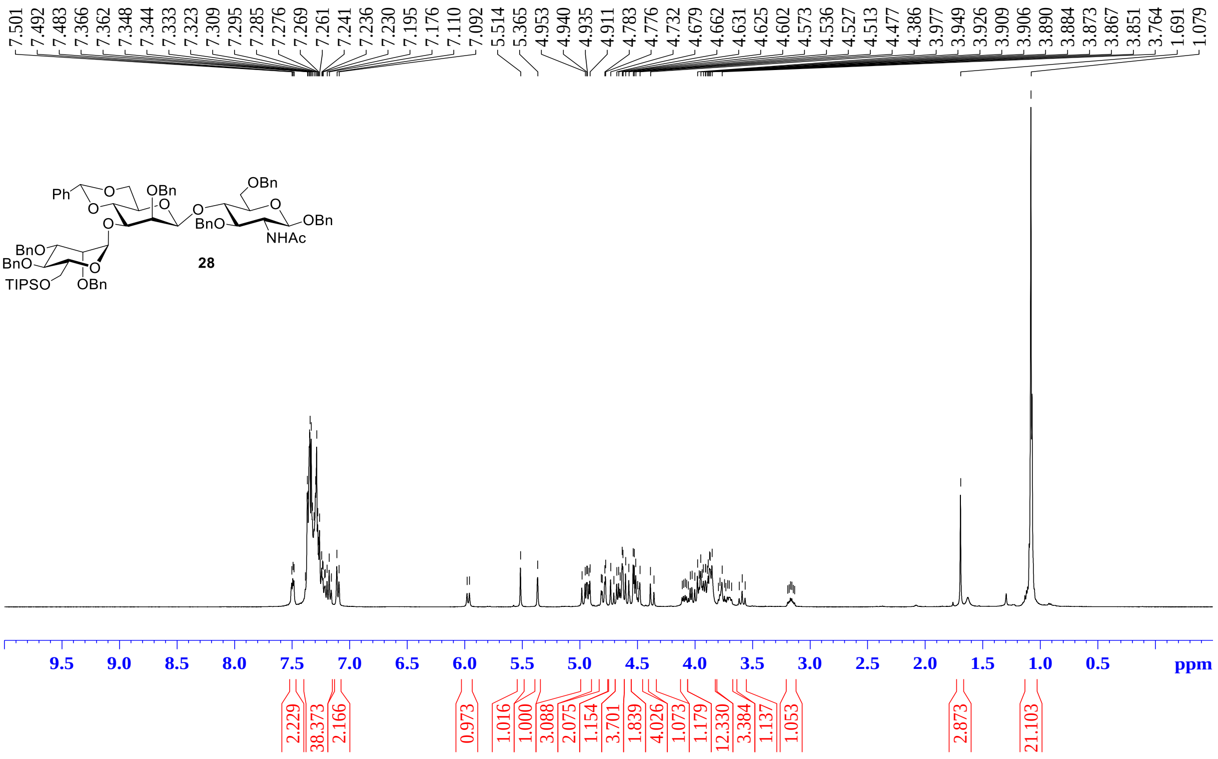

Compound 28: ^{1}H NMR ($CDCl_3$, 400 MHz)

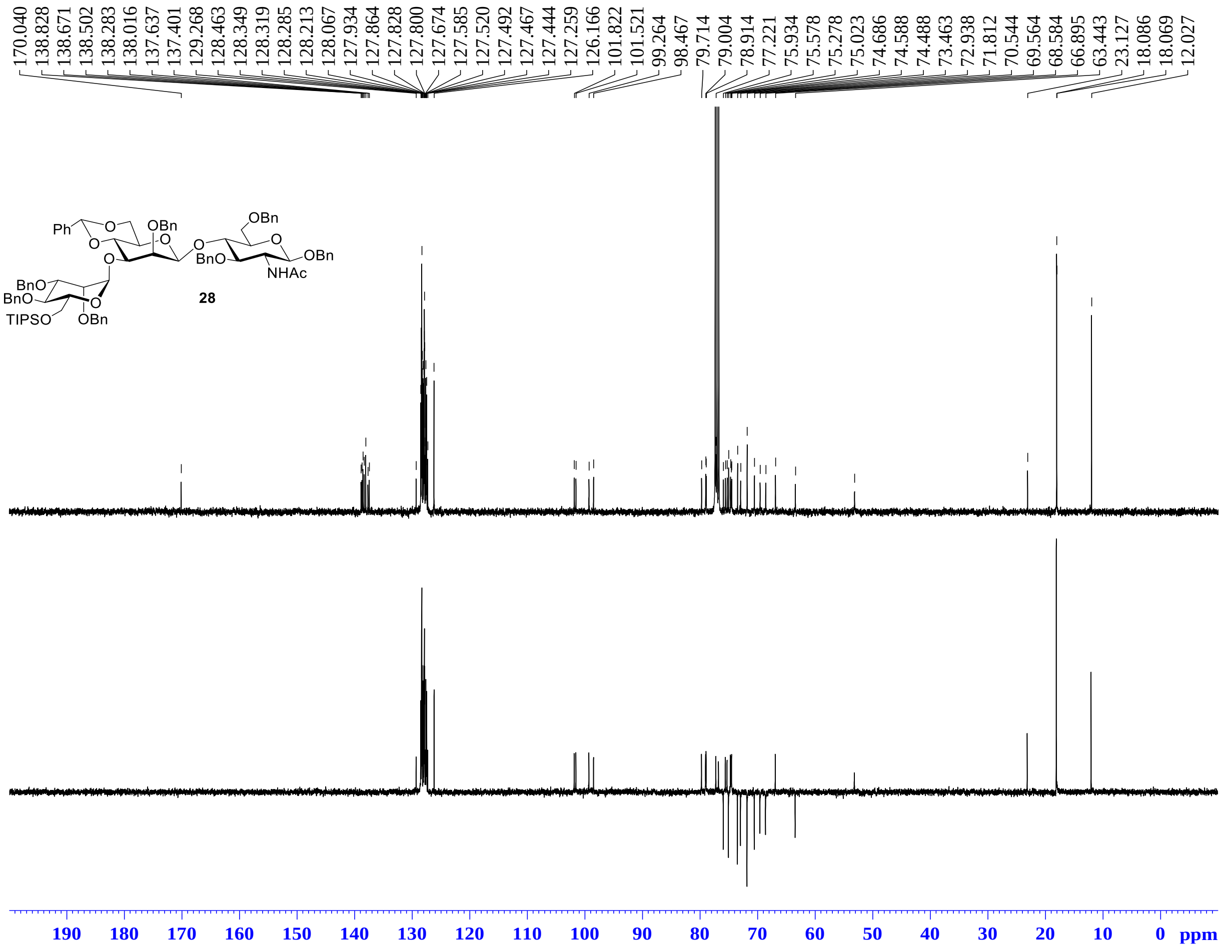

Compound 28: ^{13}C and Dept-135 NMR ($CDCl_3$, 100 MHz)

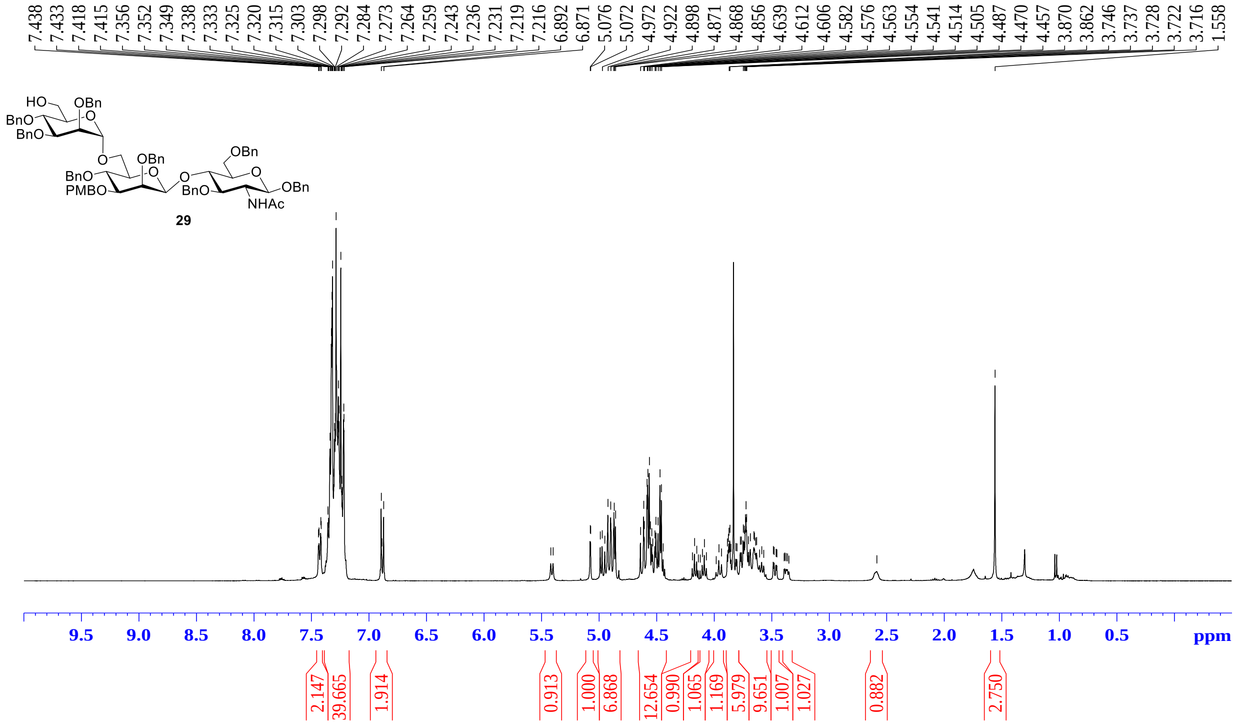

Compound 29: ^{1}H NMR ($CDCl_3$, 400 MHz)

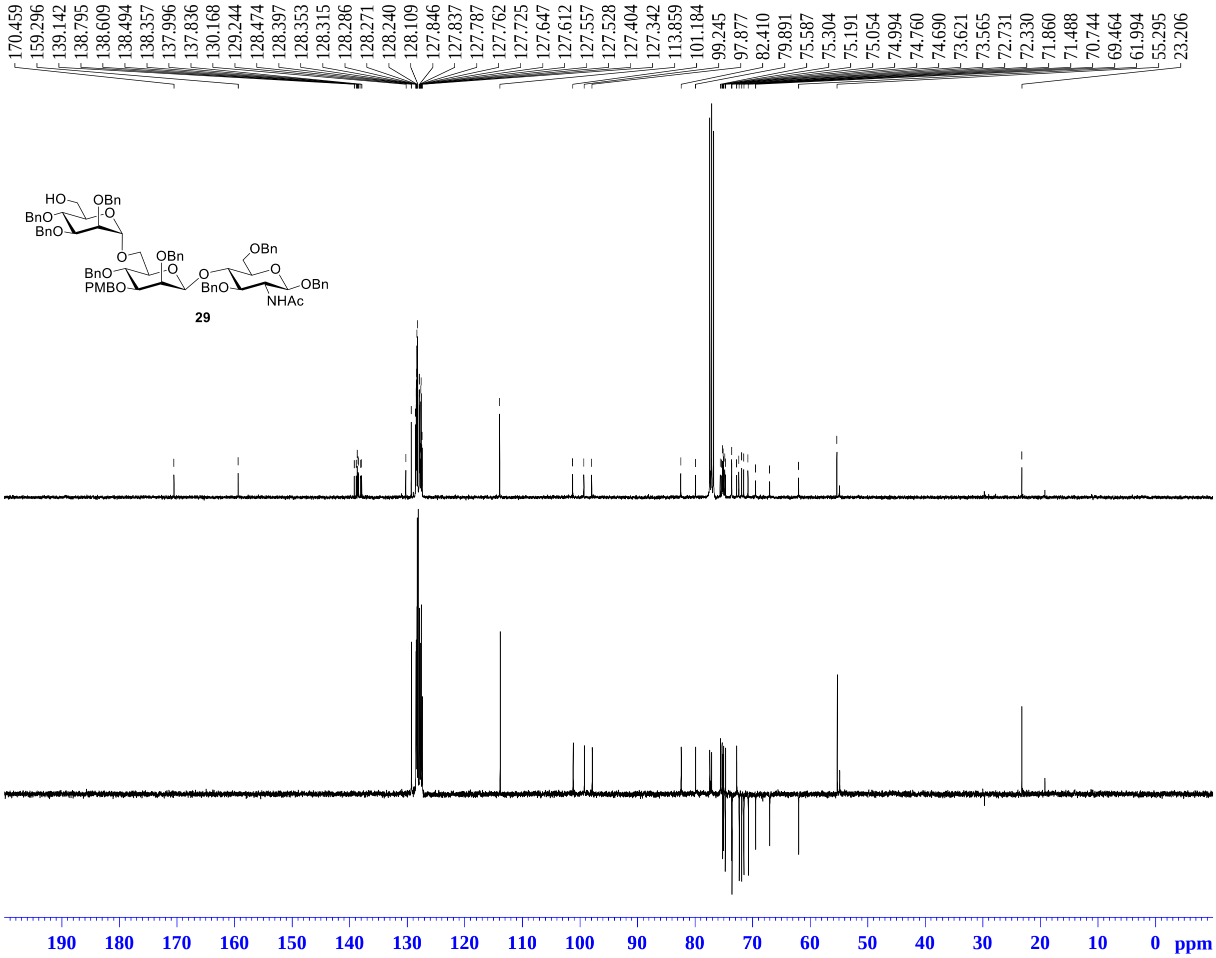

Compound 29: ^{13}C and Dept-135 NMR ($CDCl_3$, 100 MHz)

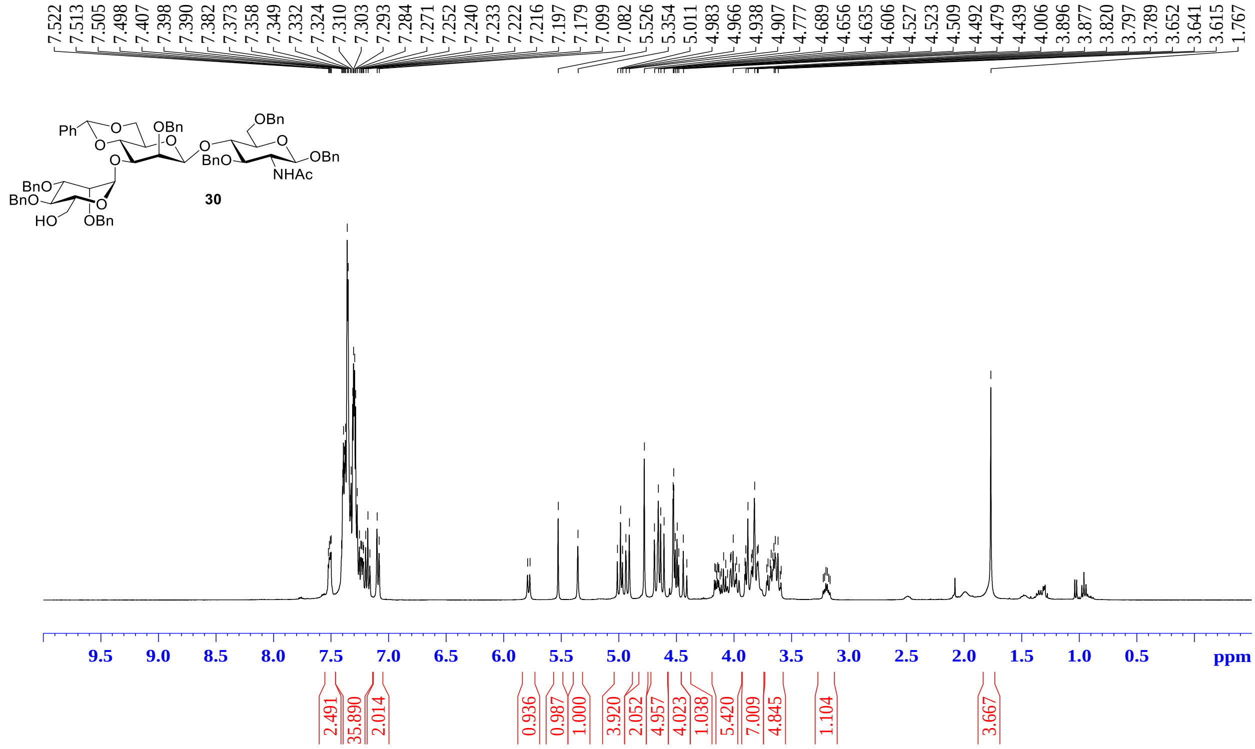

Compound 30: ^{1}H NMR ($CDCl_3$, 400 MHz)

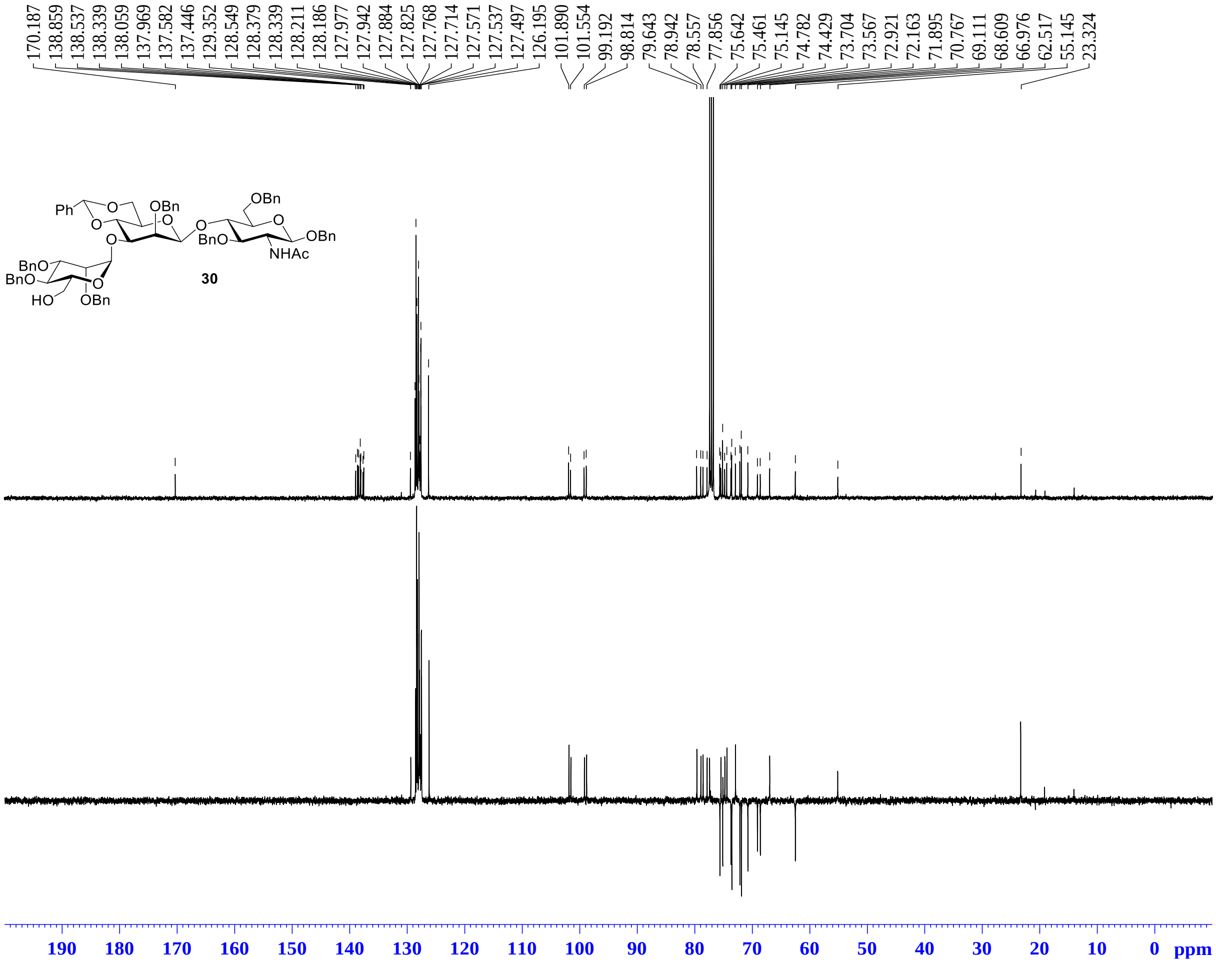

Compound 30: ^{13}C and Dept-135 NMR ($CDCl_3$, 100 MHz)

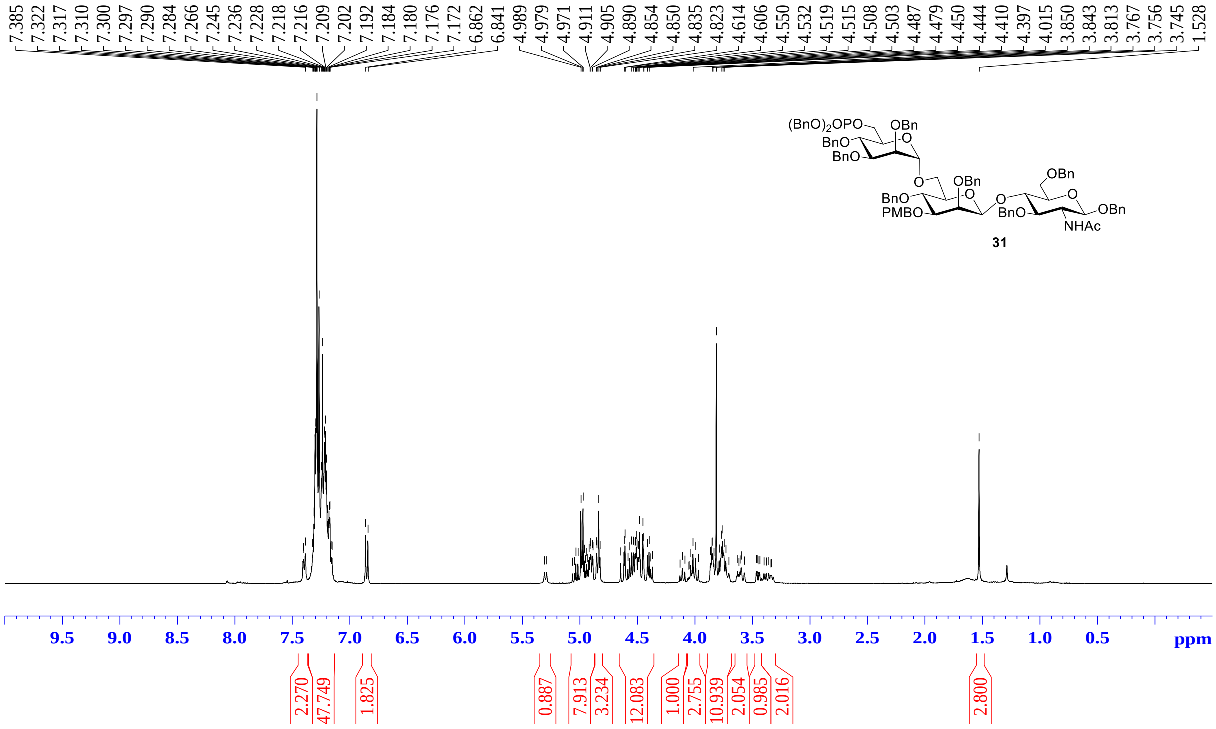

Compound 31: ^{1}H NMR ($CDCl_3$, 400 MHz)

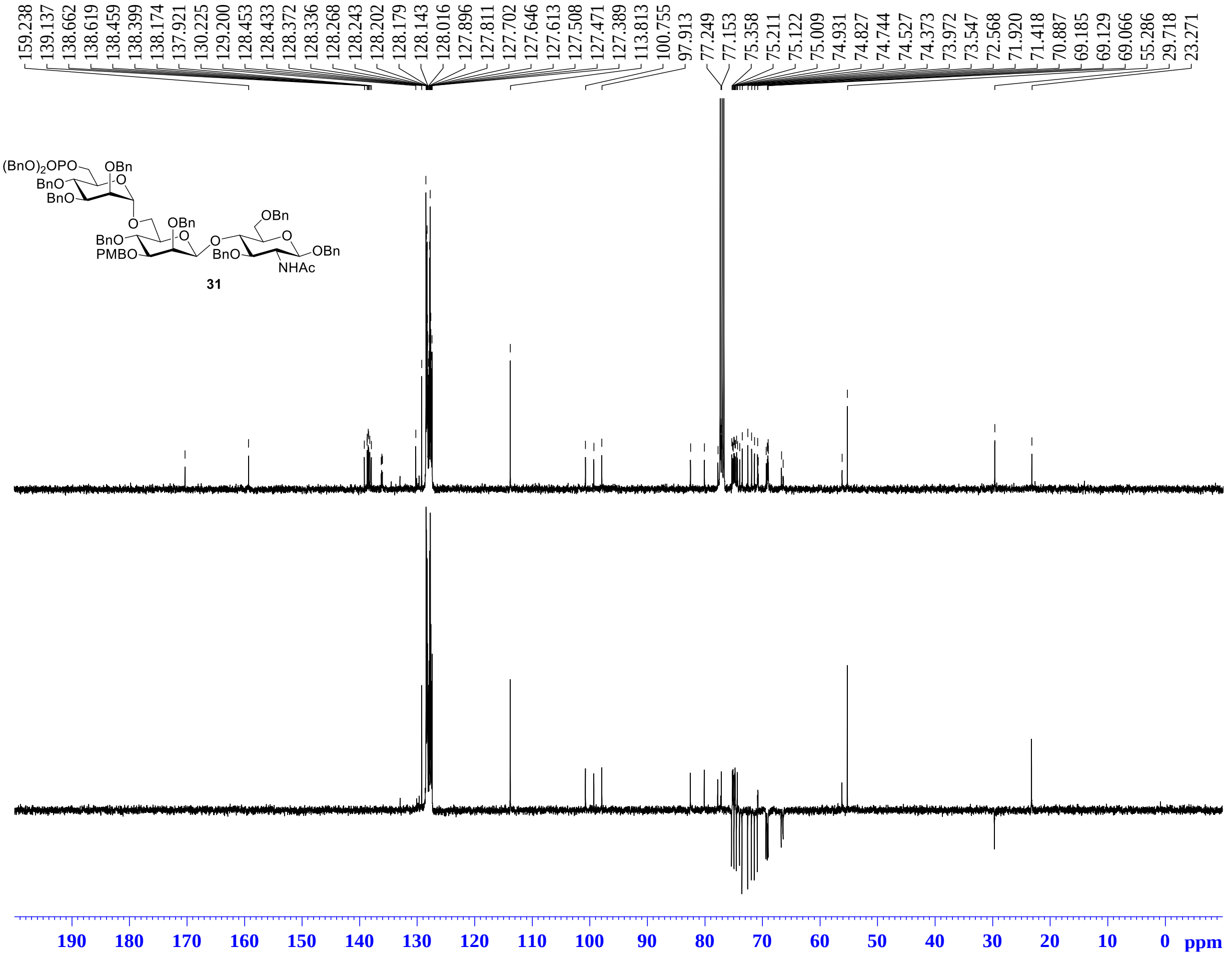

Compound 31: ^{13}C and Dept-135 NMR (CDCl$_3$, 100 MHz)

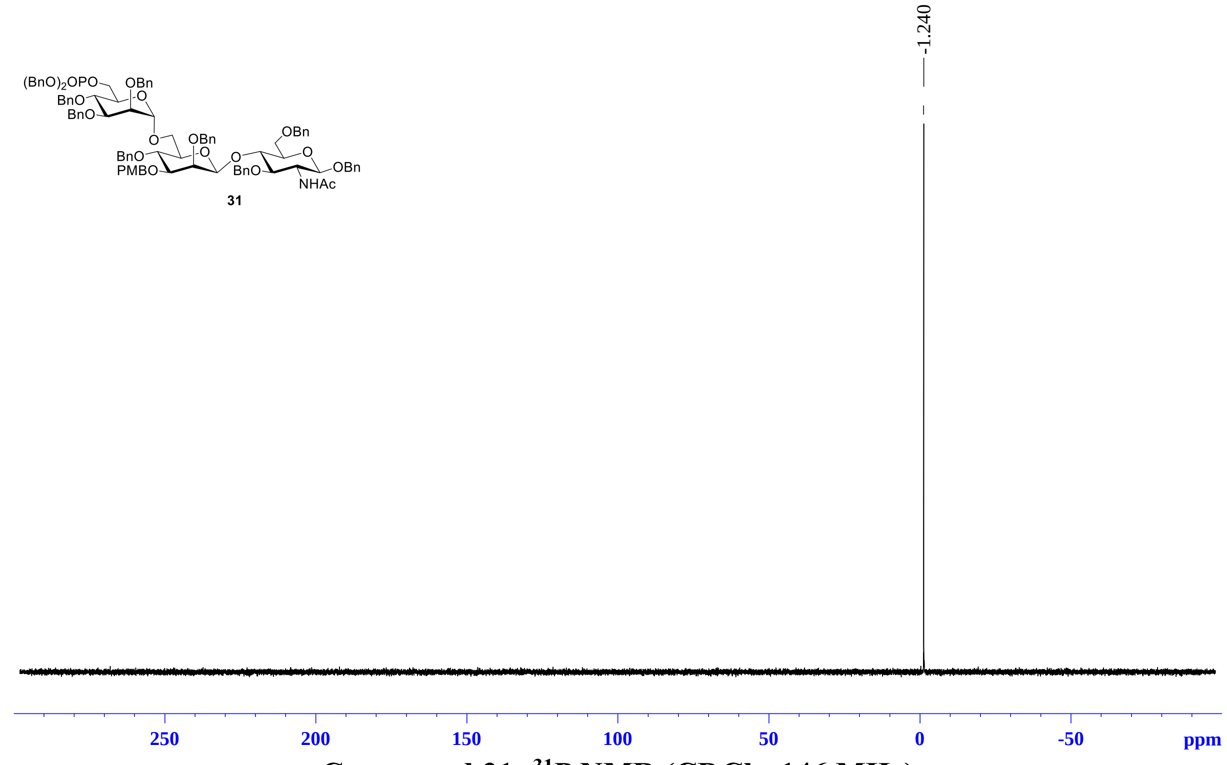

Compound 31: ^{31}P NMR (CDCl$_3$, 146 MHz)

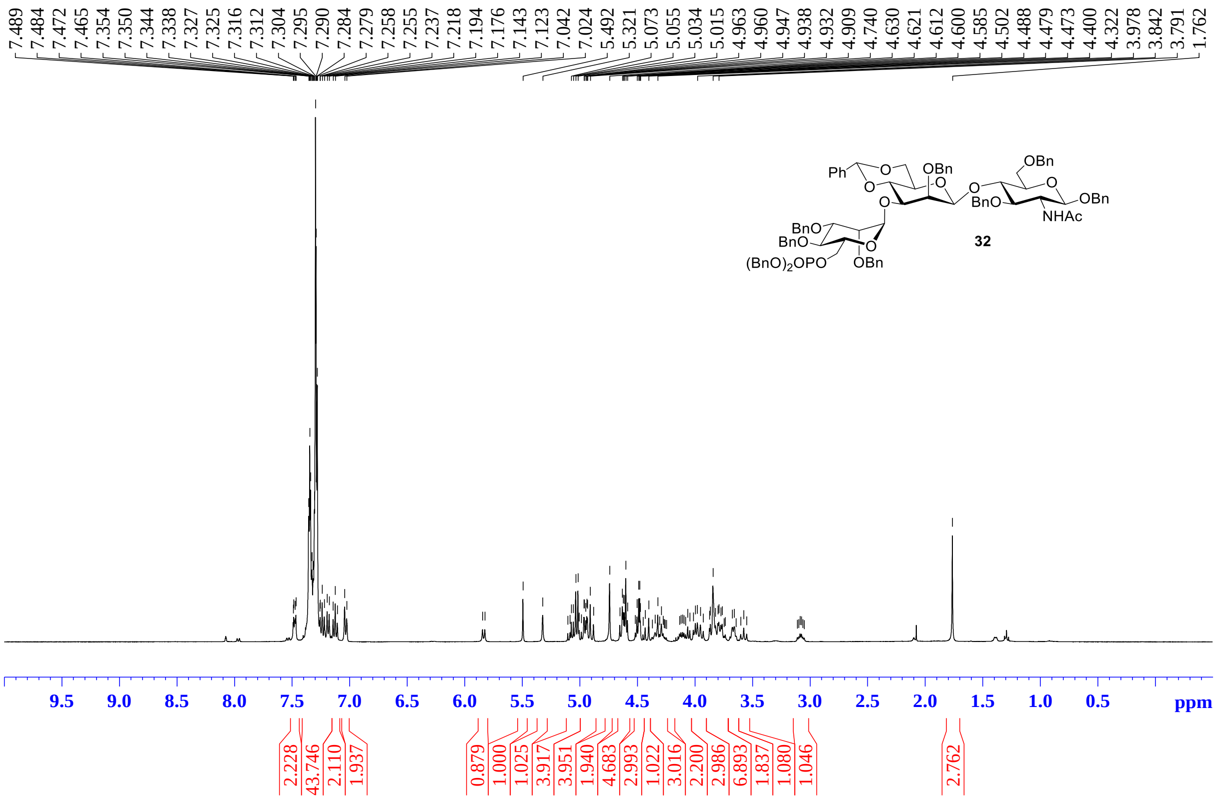

Compound 32: ^{1}H NMR (CDCl$_3$, 400 MHz)

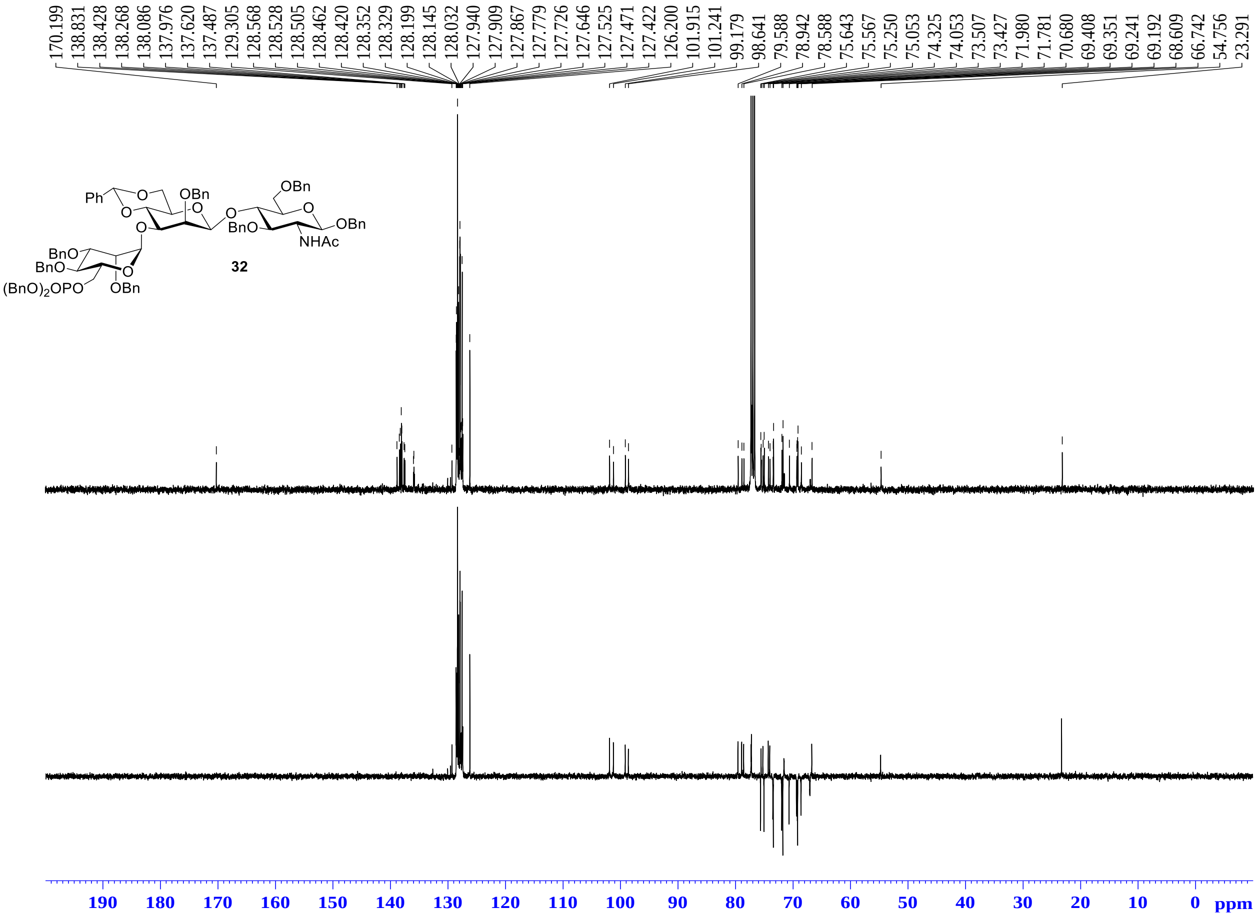

Compound 32: ^{13}C and Dept-135 NMR (CDCl$_3$, 100 MHz)

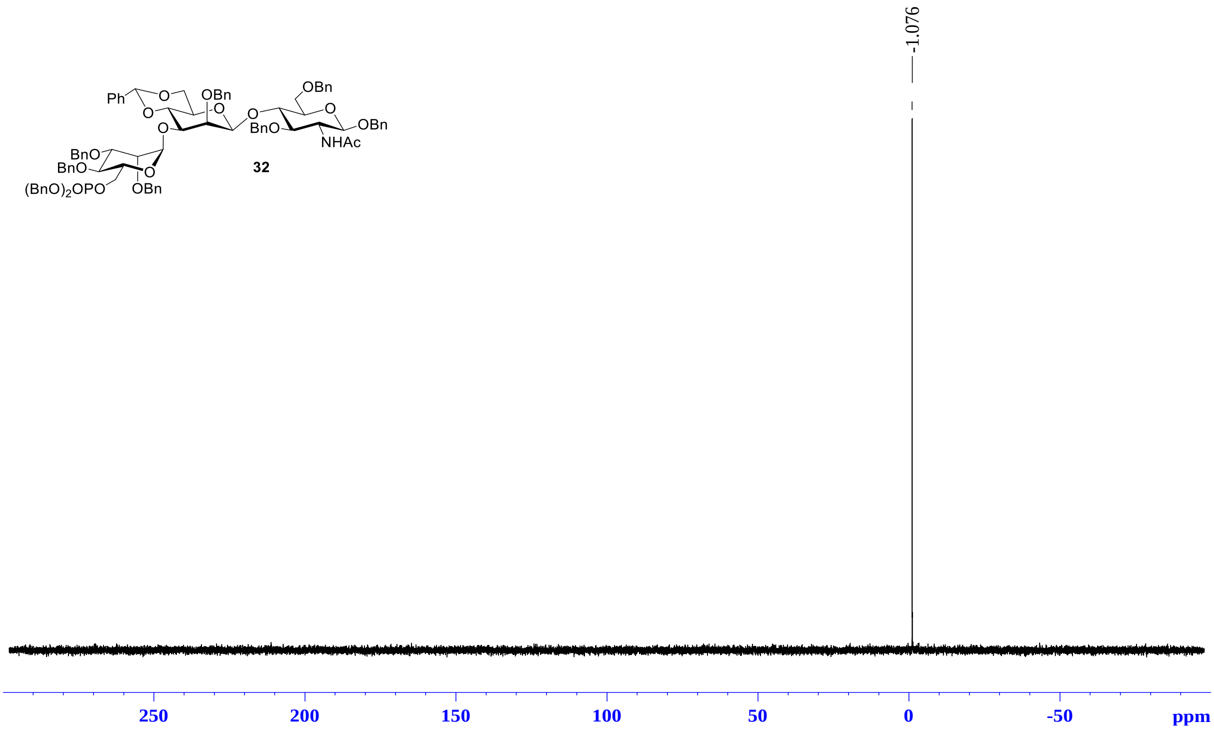

Compound 32: ^{31}P NMR ($CDCl_3$, 146 MHz)

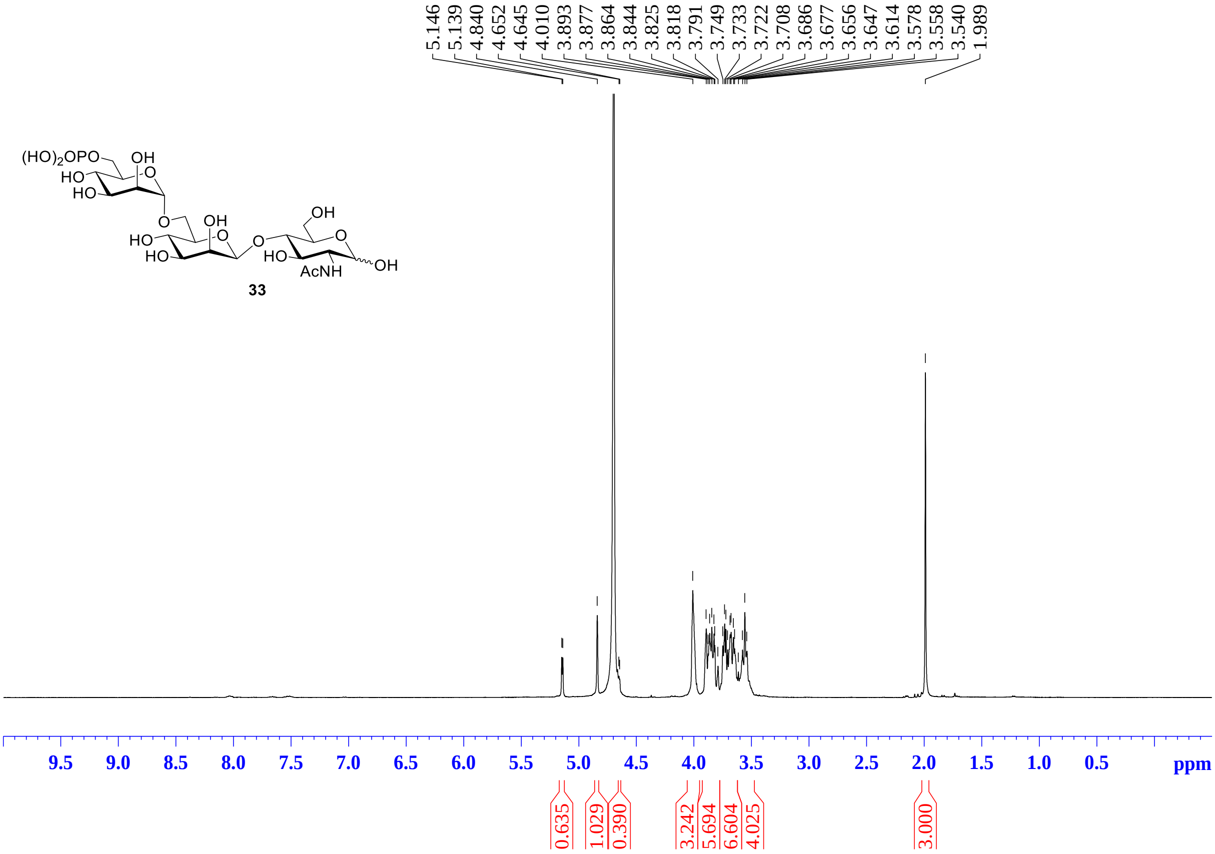

Compound 33: ^{1}H NMR (D_2O, 400 MHz)

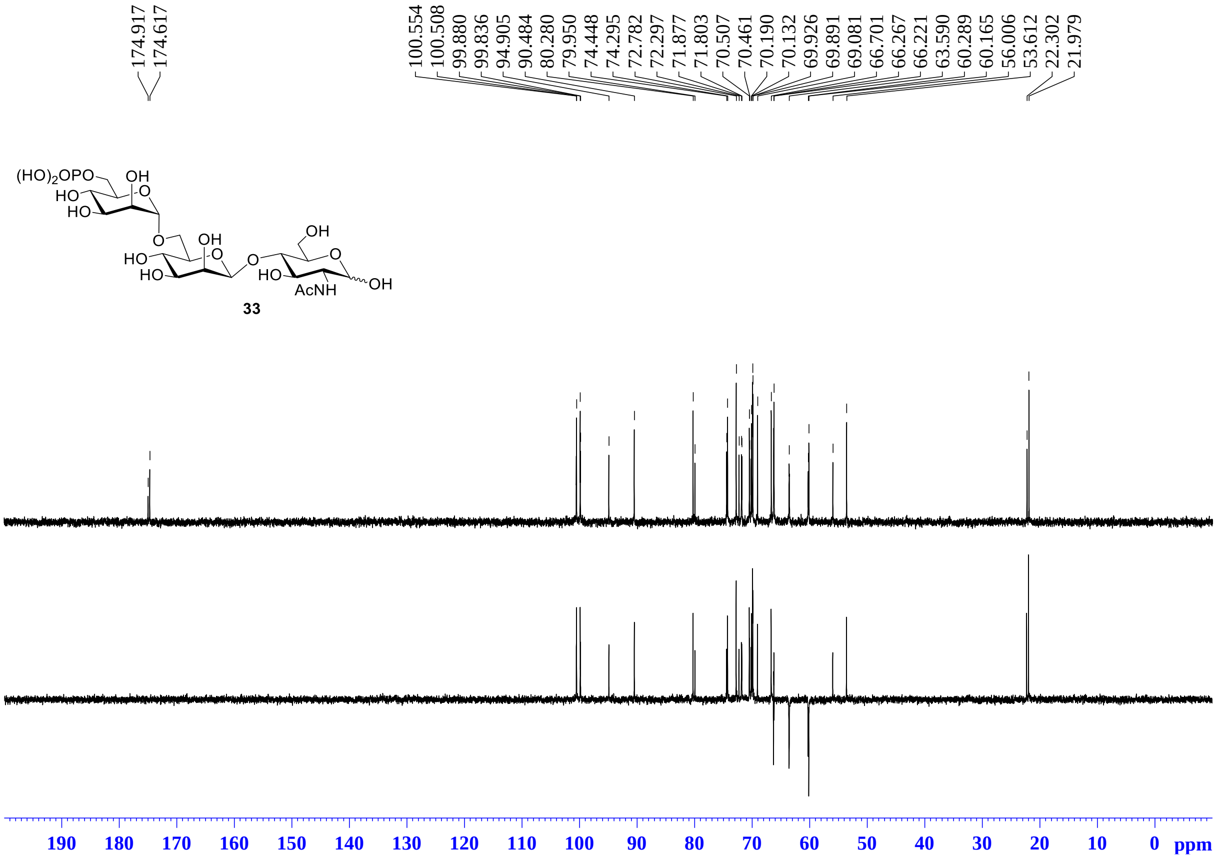

Compound 33: ^{13}C and Dept-135 NMR (D_2O, 100 MHz)

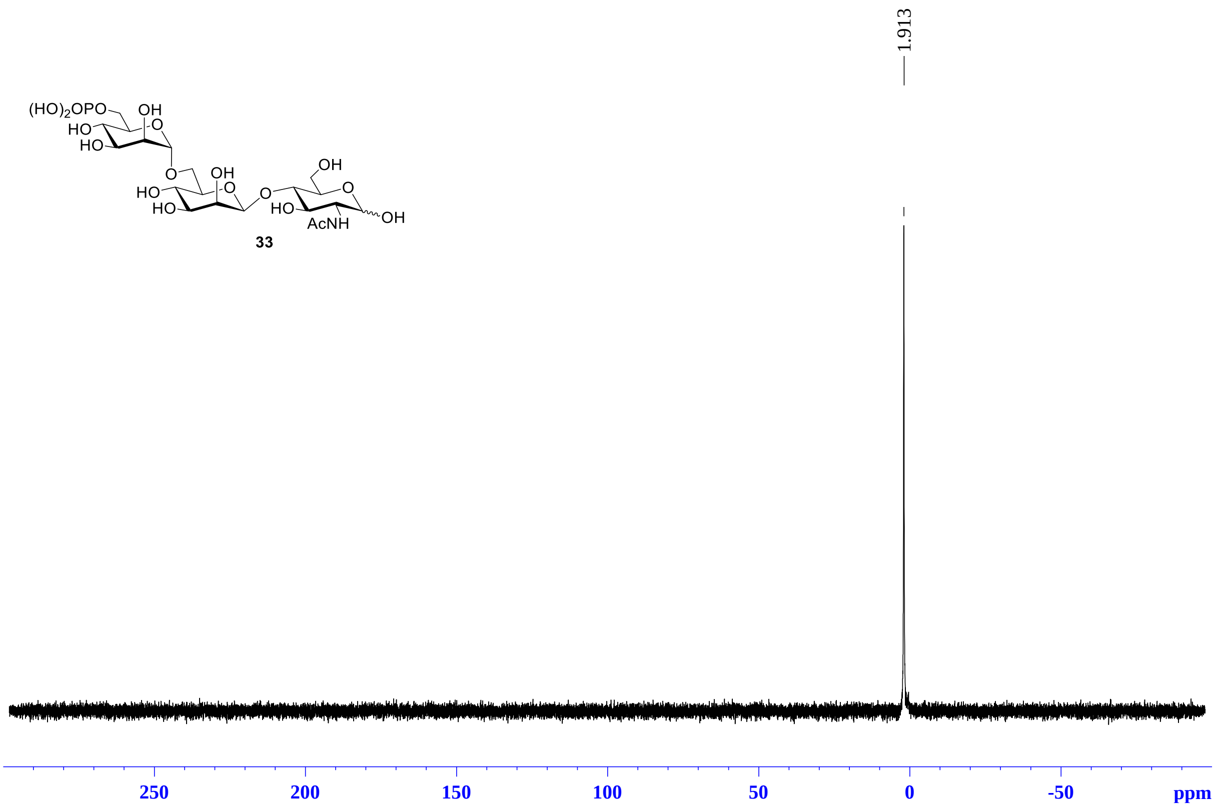

Compound 33: ^{31}P NMR (D_2O, 146 MHz)

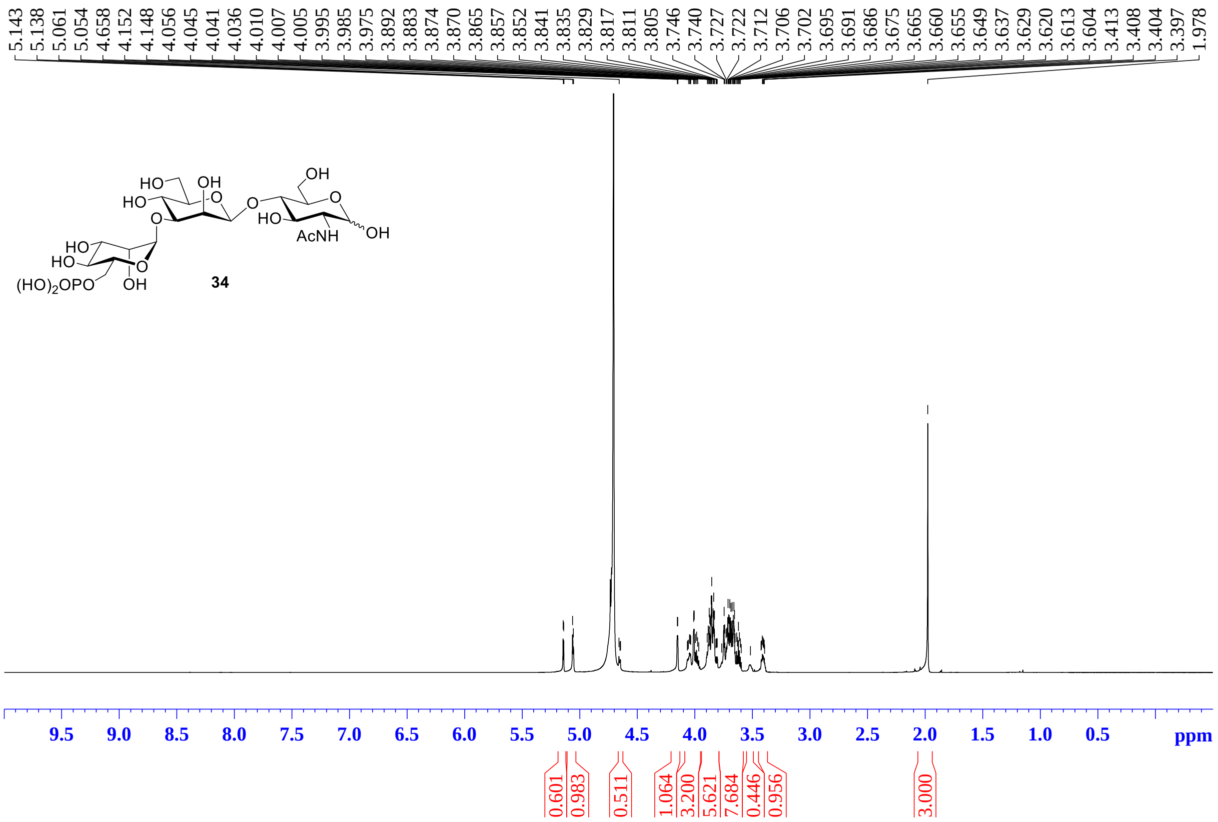

Compound 34: ^{1}H NMR (D$_2$O, 400 MHz)

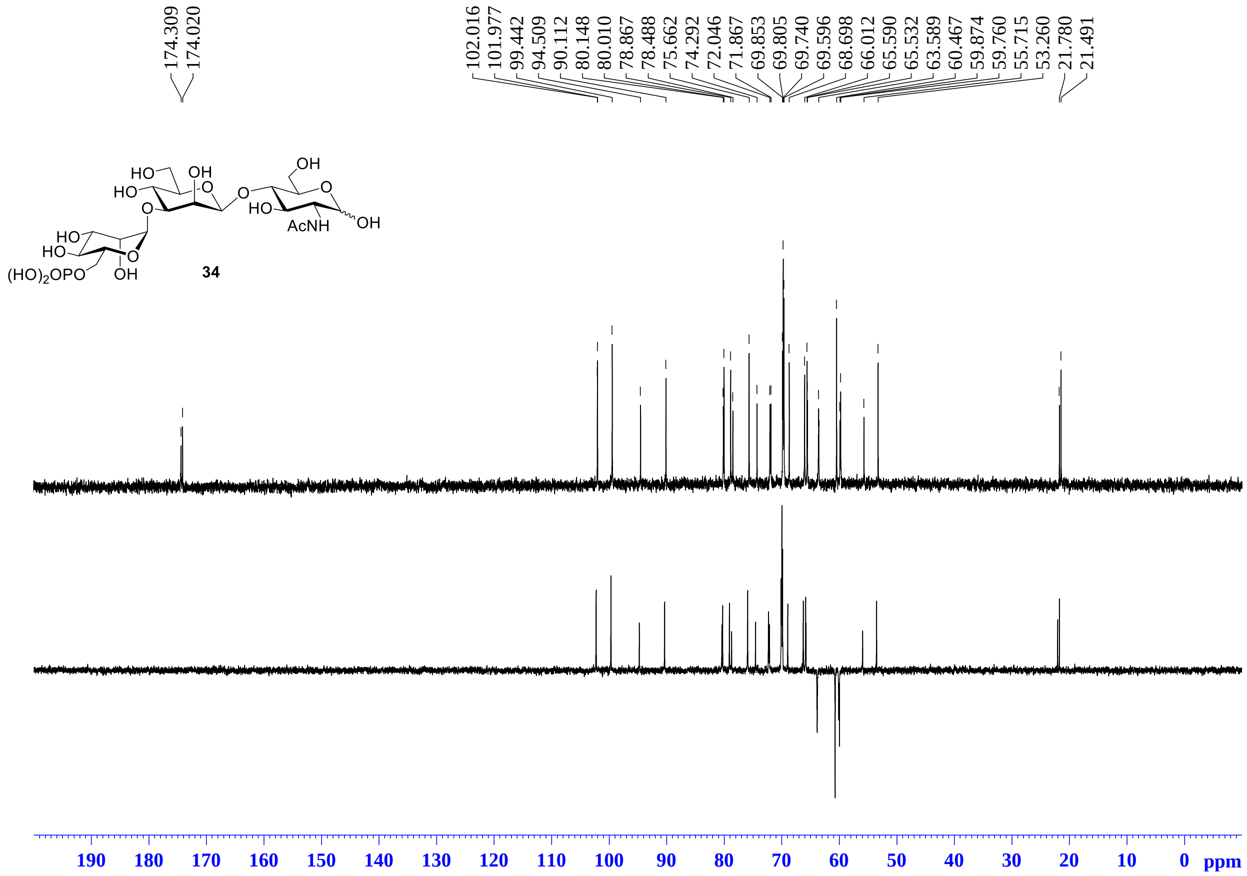

Compound 34: ^{13}C and Dept-135 NMR (D$_2$O, 100 MHz)

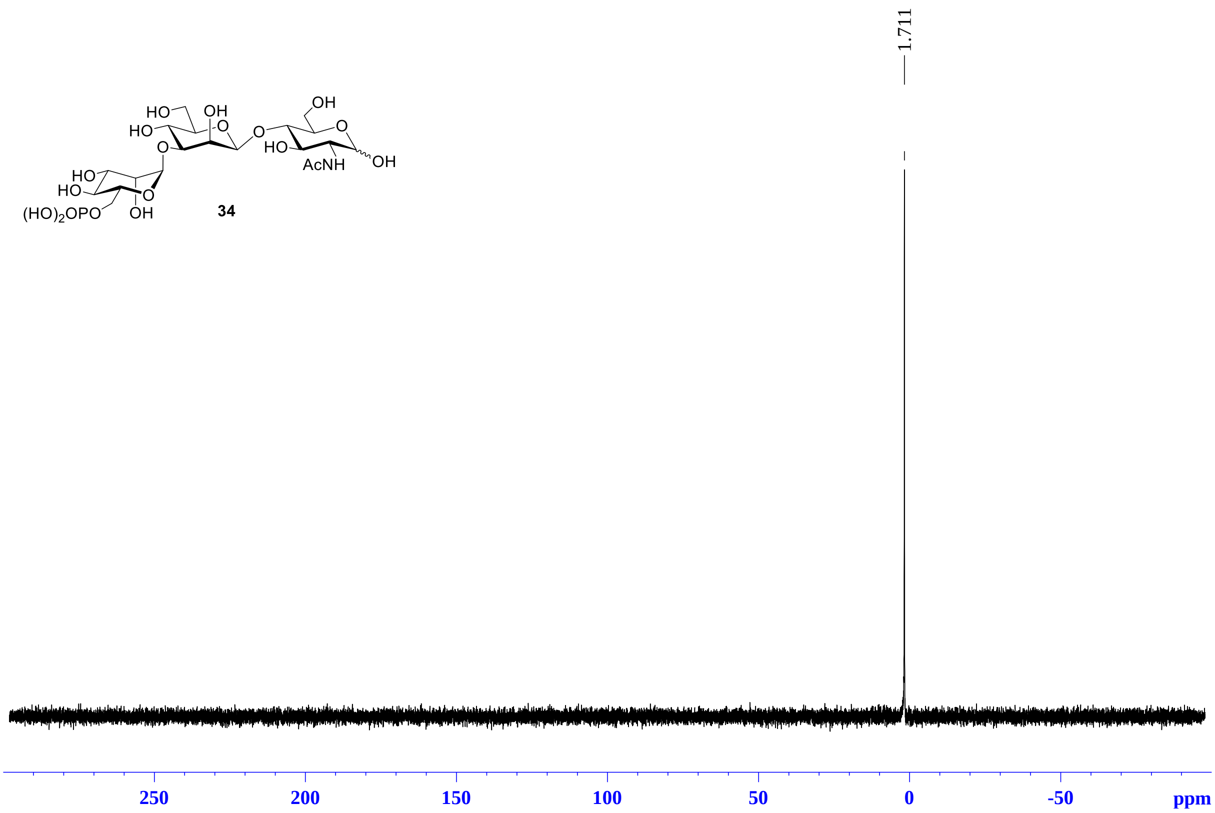

Compound 34: ^{31}P NMR (D_2O, 146 MHz)

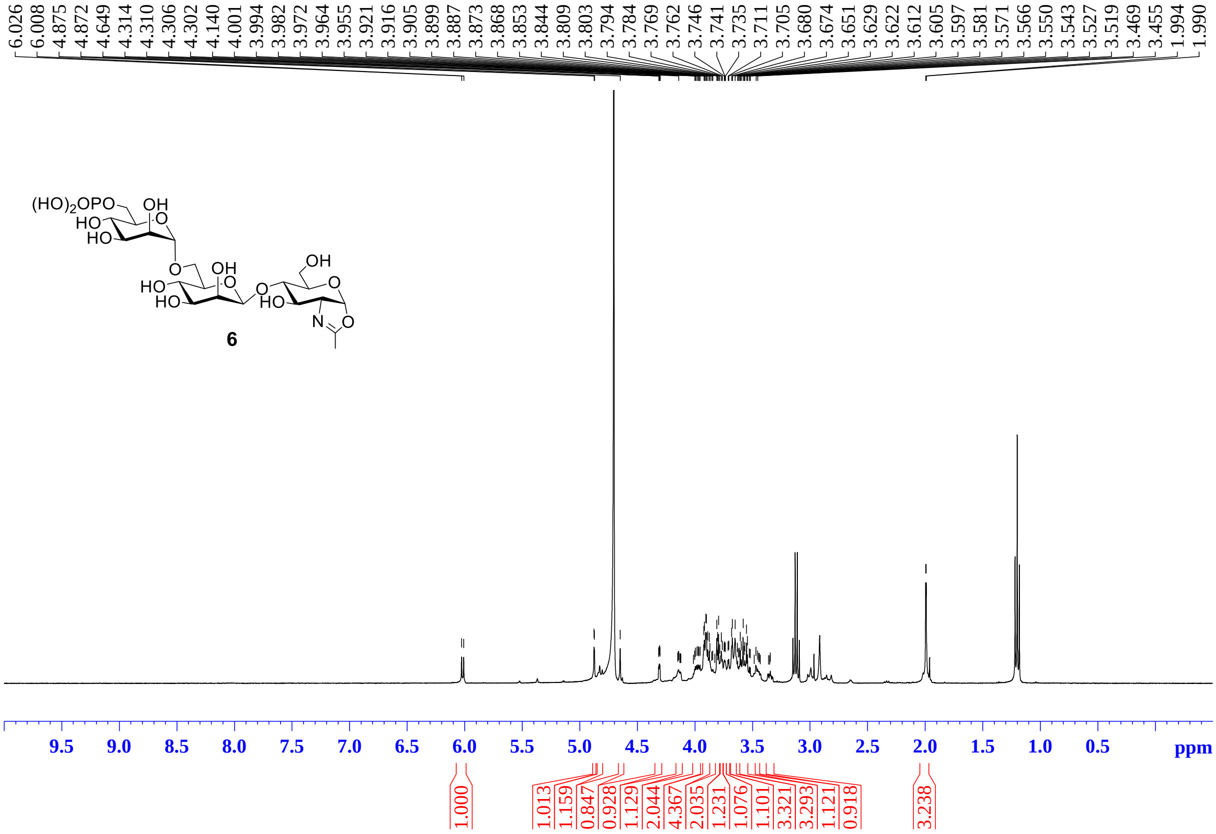

Compound 6: ^{1}H NMR (D_2O, 400 MHz)

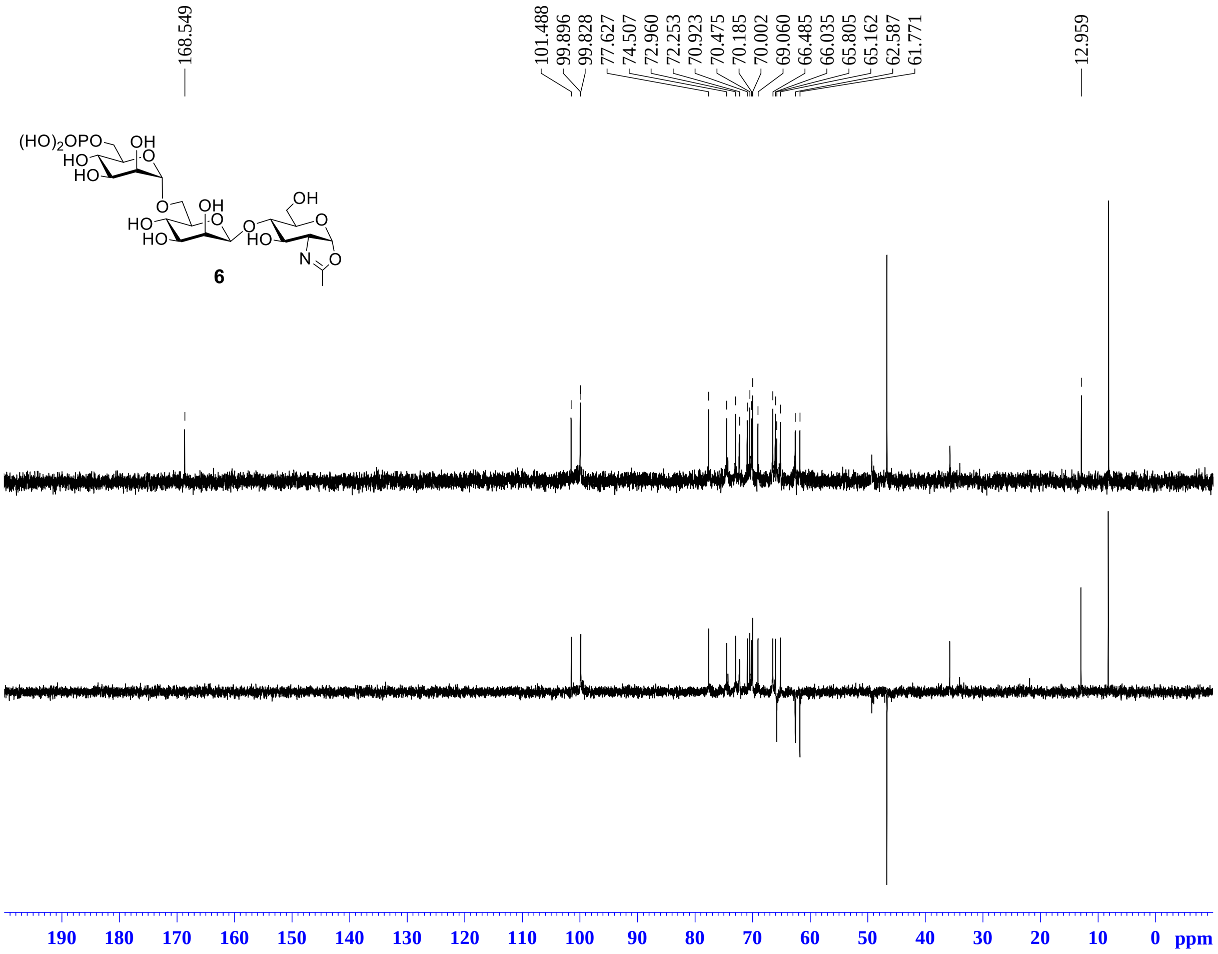

Compound 6: ^{13}C and Dept-135 NMR (D_2O, 100 MHz)

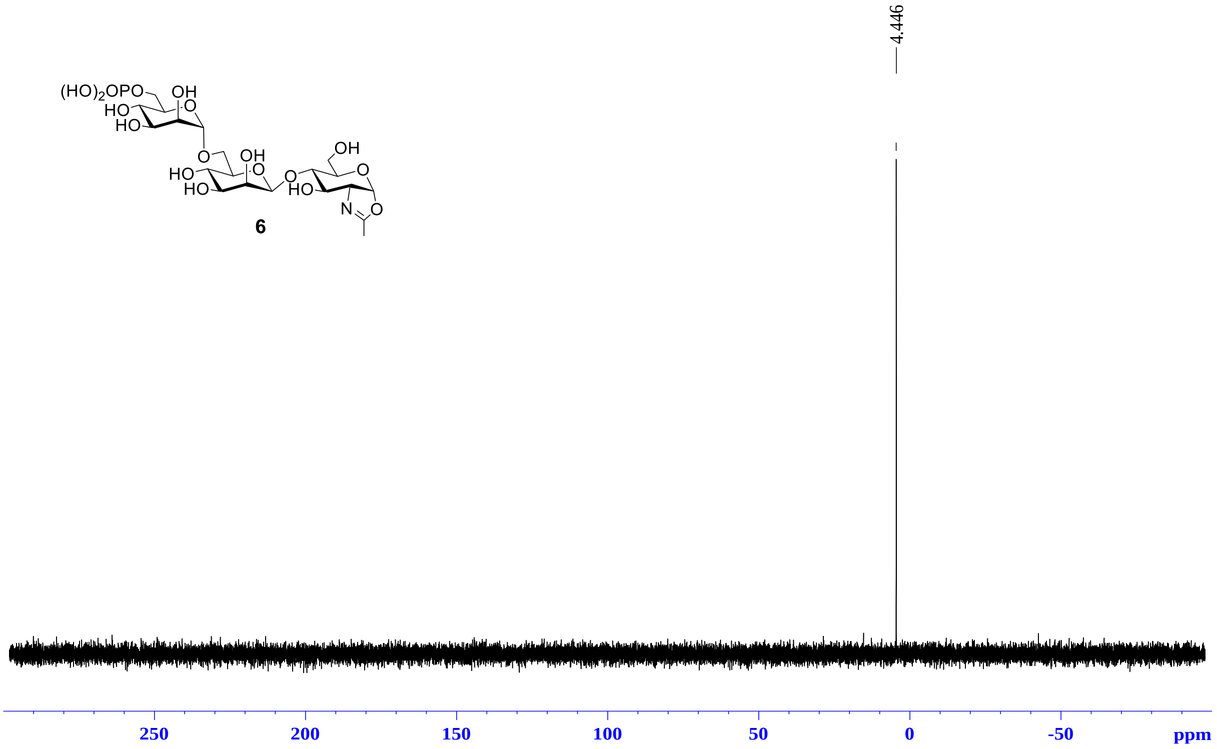

Compound 6: ^{31}P NMR (D_2O, 146 MHz)

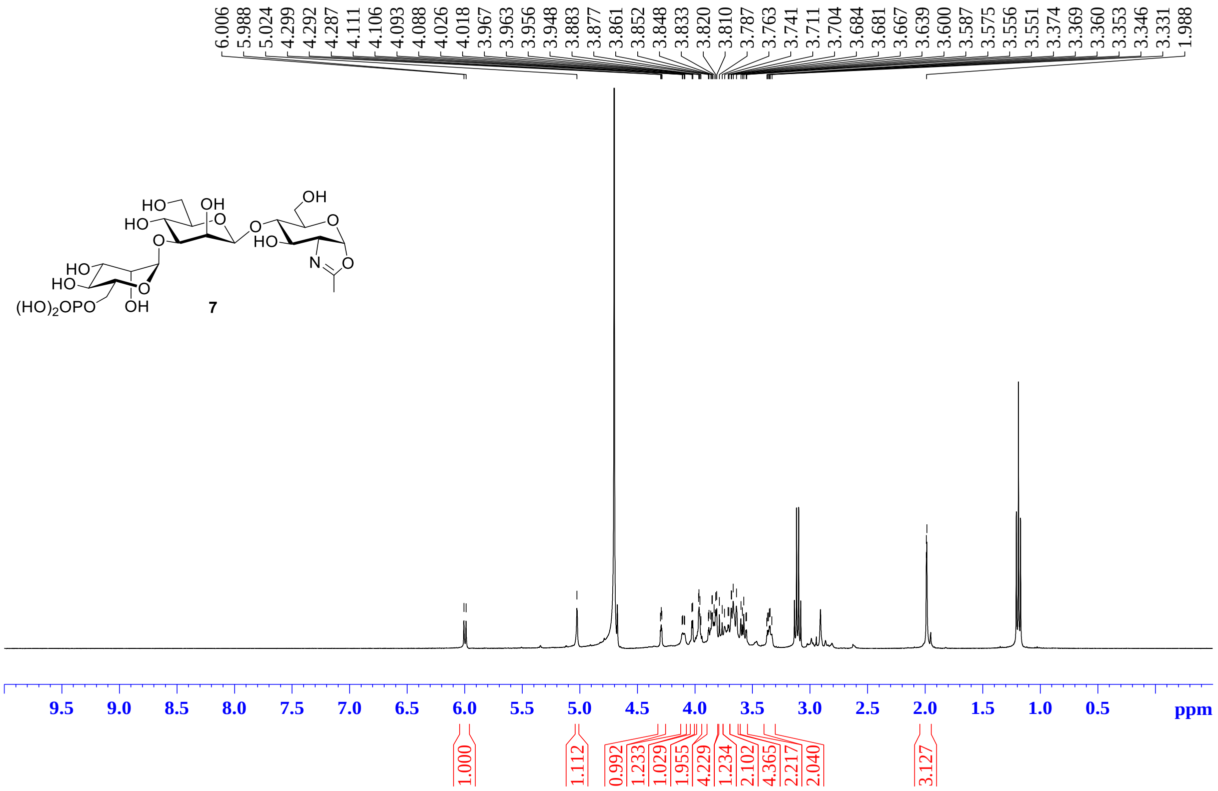

Compound 7: ^{1}H NMR (D_2O, 400 MHz)

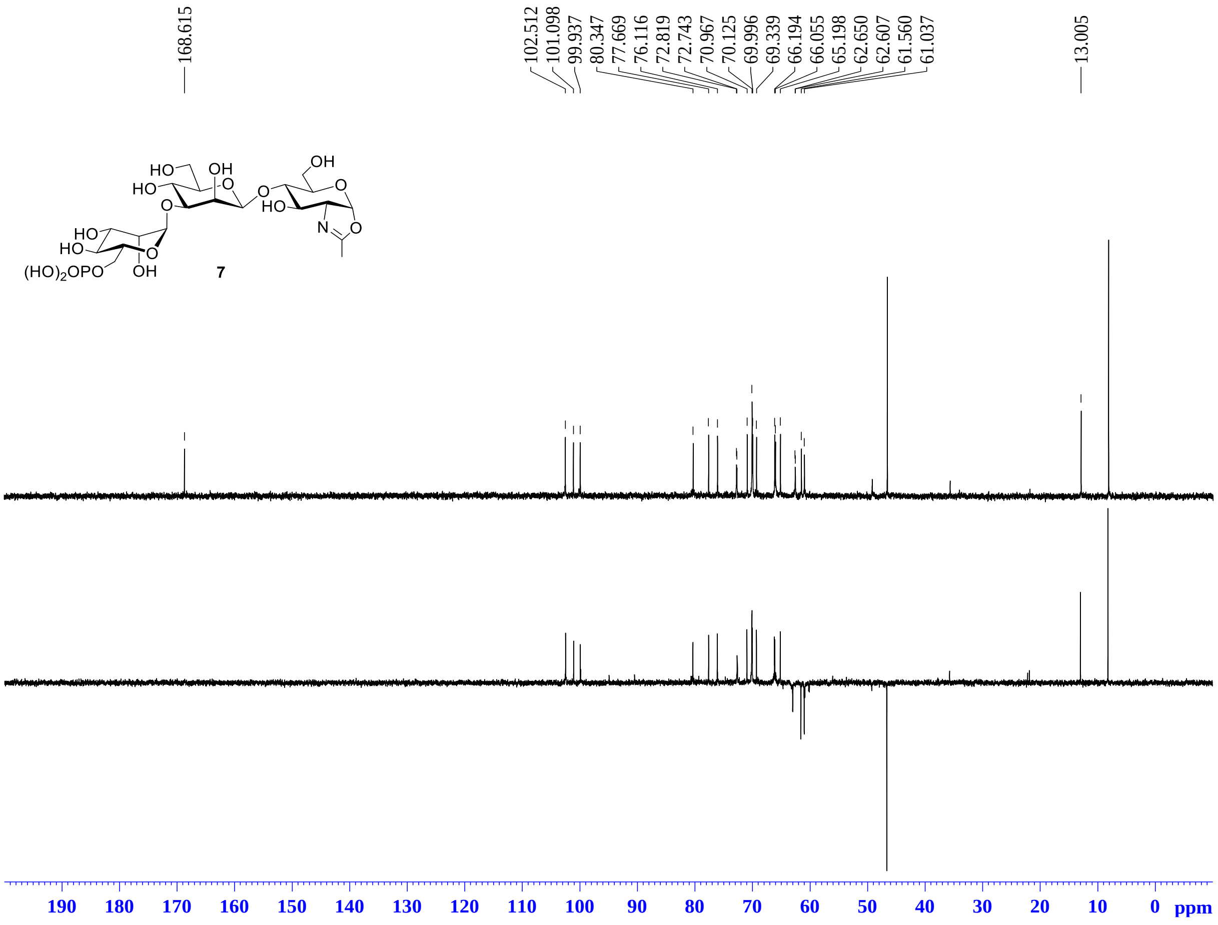

Compound 7: ^{13}C and Dept-135 NMR (D_2O, 100 MHz)

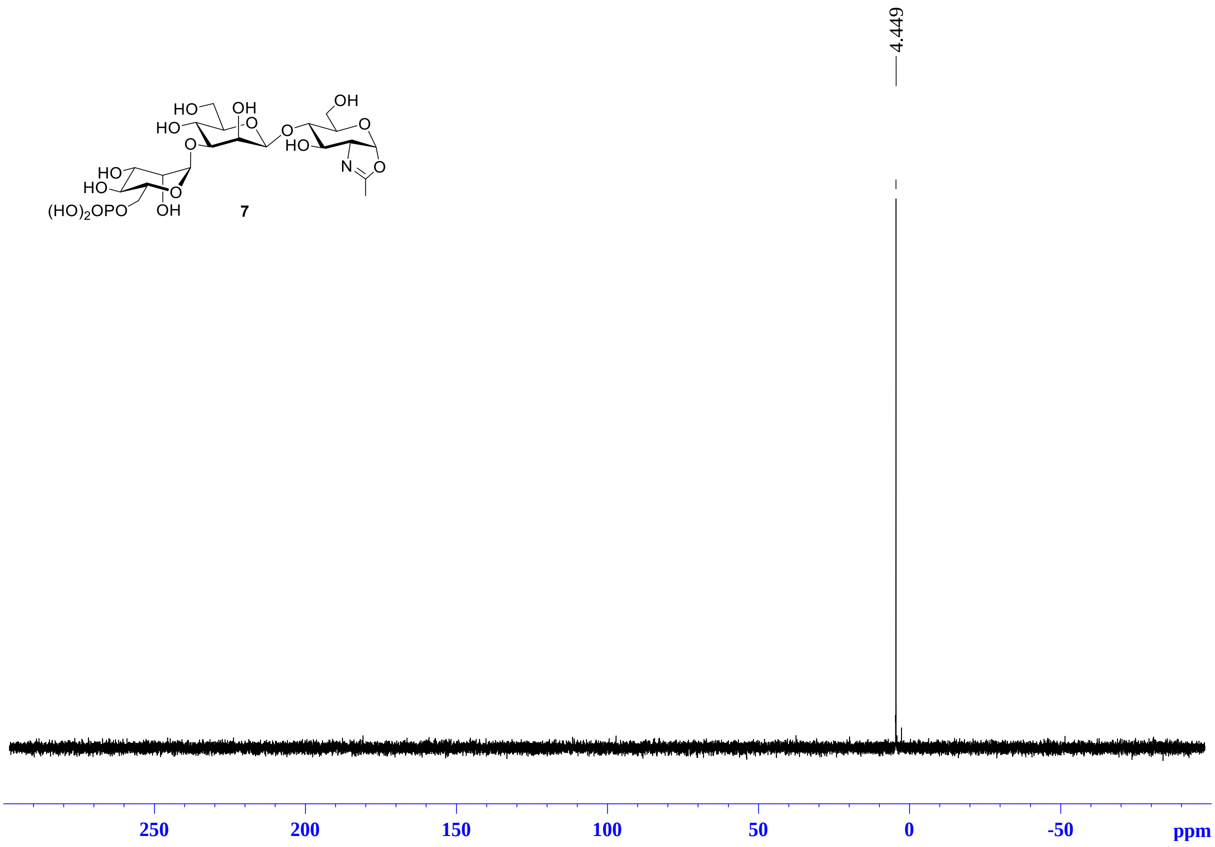

Compound 7: ^{31}P NMR (D_2O, 146 MHz)

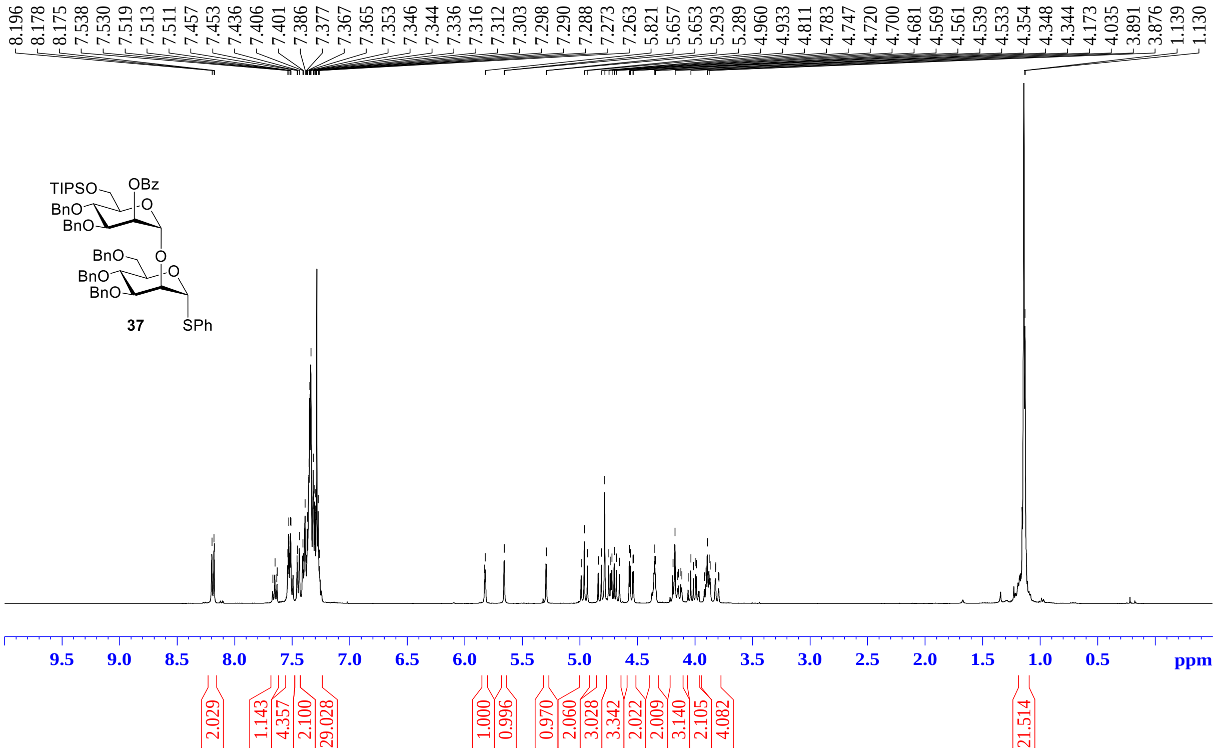

Compound 37: ^{1}H NMR ($CDCl_3$, 400 MHz)

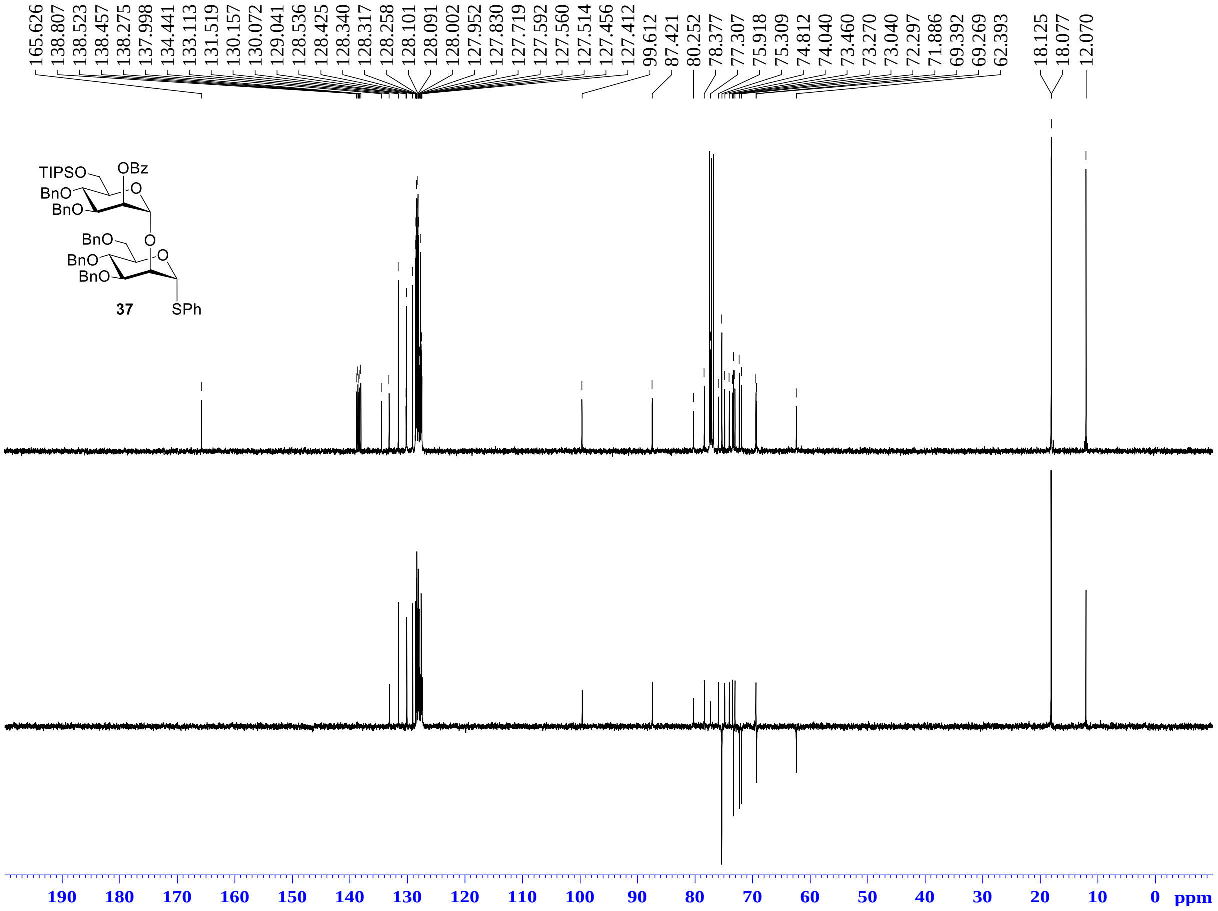

Compound 37: ^{13}C and Dept-135 NMR ($CDCl_3$, 100 MHz)

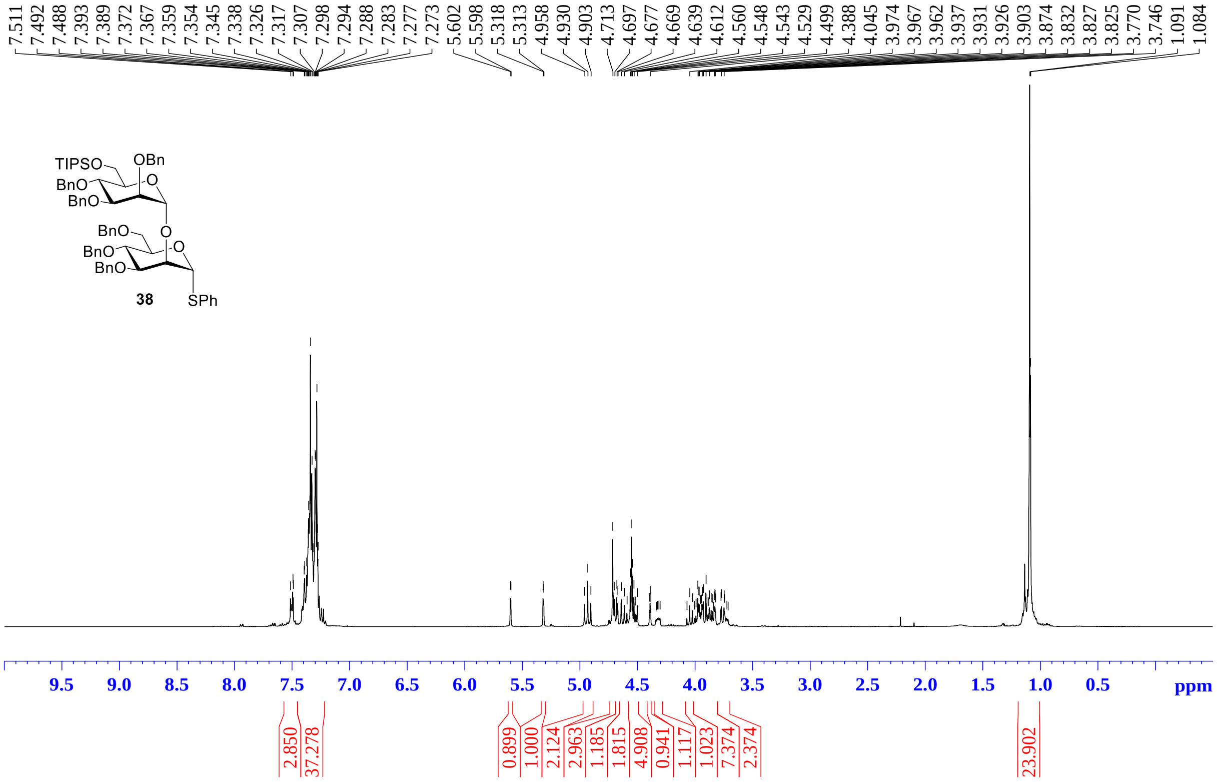

Compound 38: ^{1}H NMR ($CDCl_3$, 400 MHz)

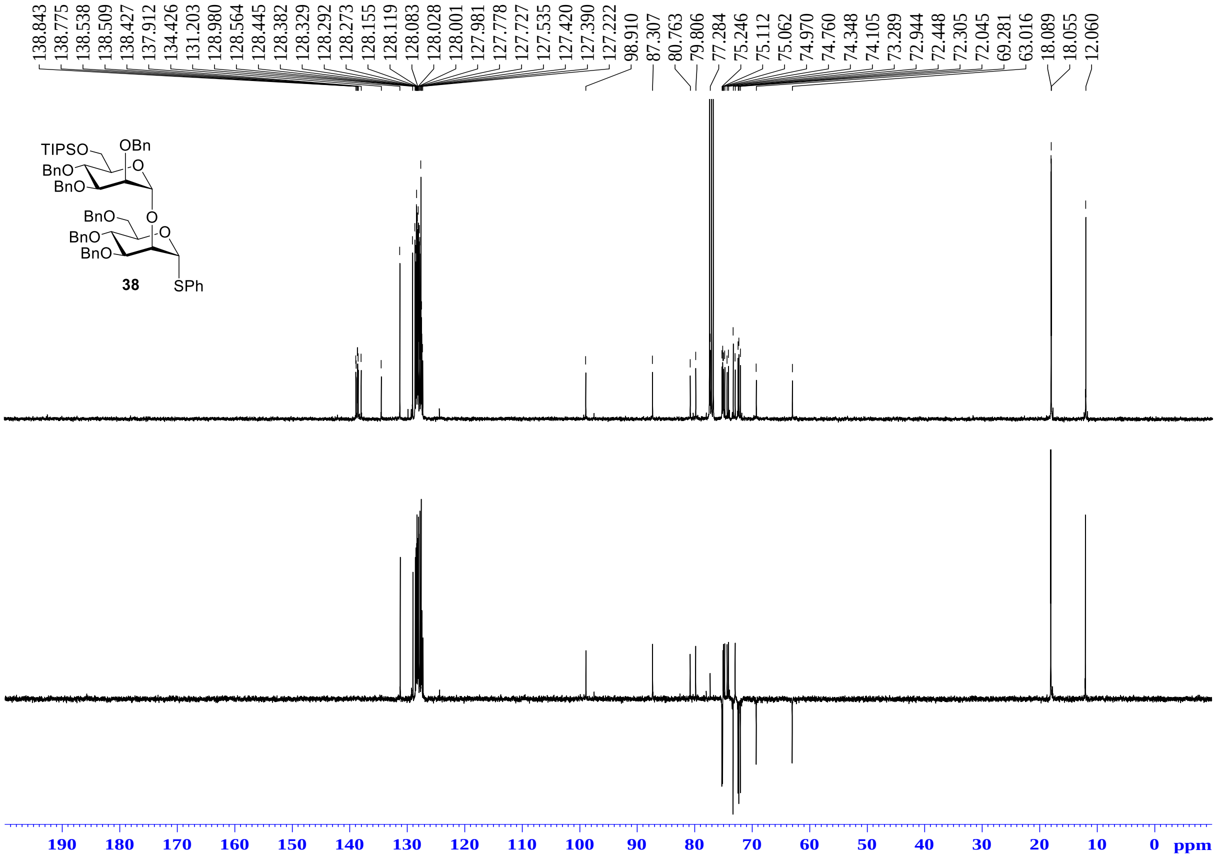

Compound 38: ^{13}C and Dept-135 NMR (CDCl$_3$, 100 MHz)

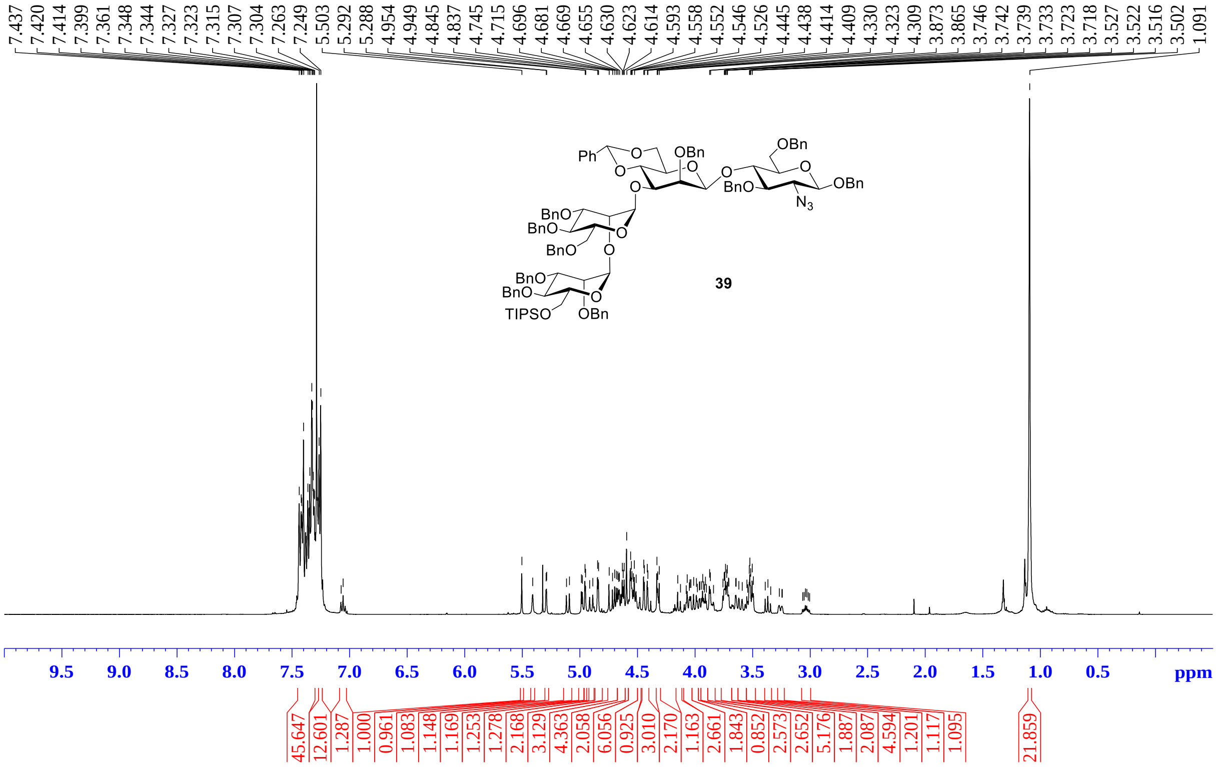

Compound 39: ^{1}H NMR (CDCl$_3$, 400 MHz)

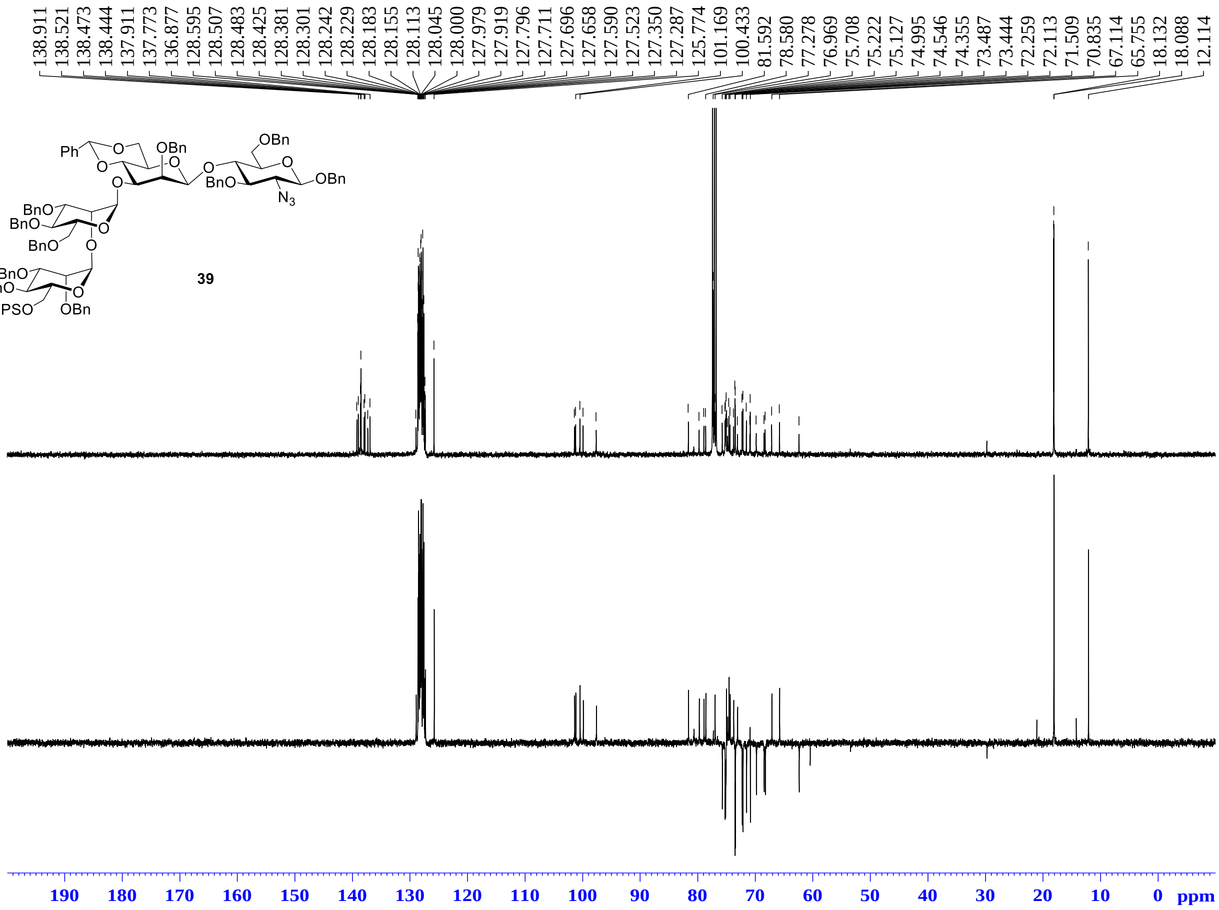

Compound 39: ^{13}C and Dept-135 NMR ($CDCl_3$, 100 MHz)

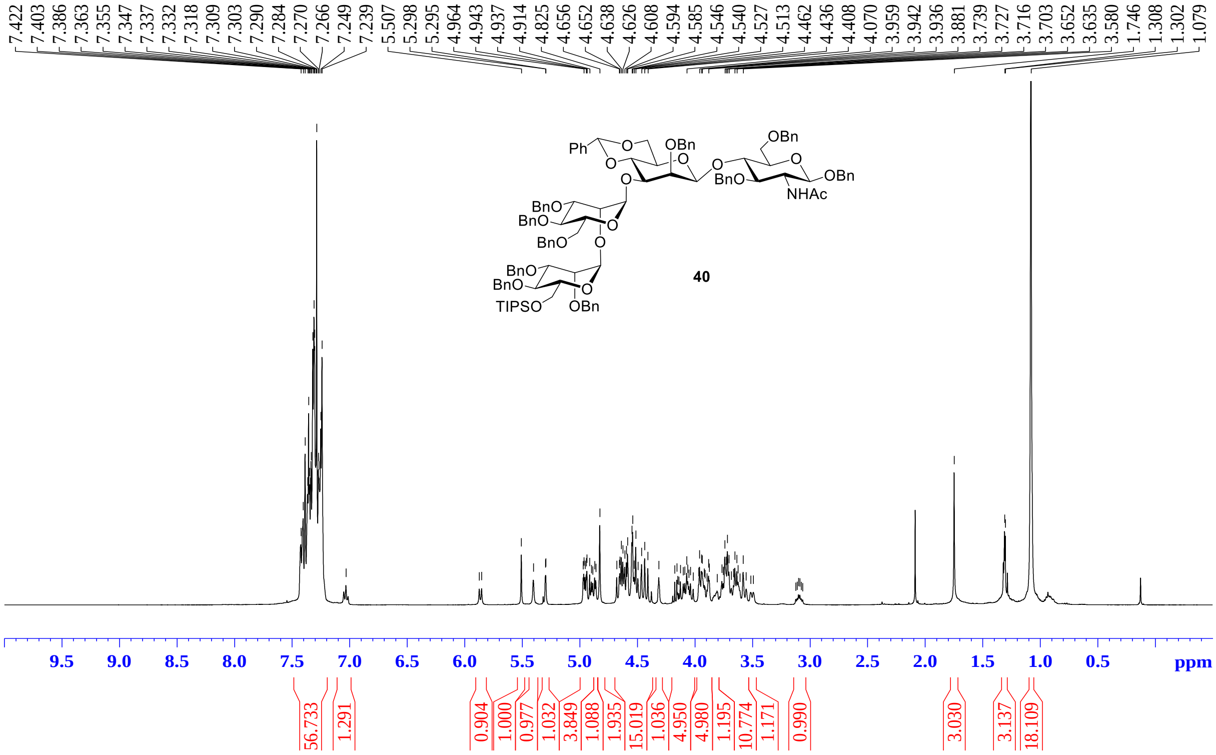

Compound 40: ^{1}H NMR ($CDCl_3$, 400 MHz)

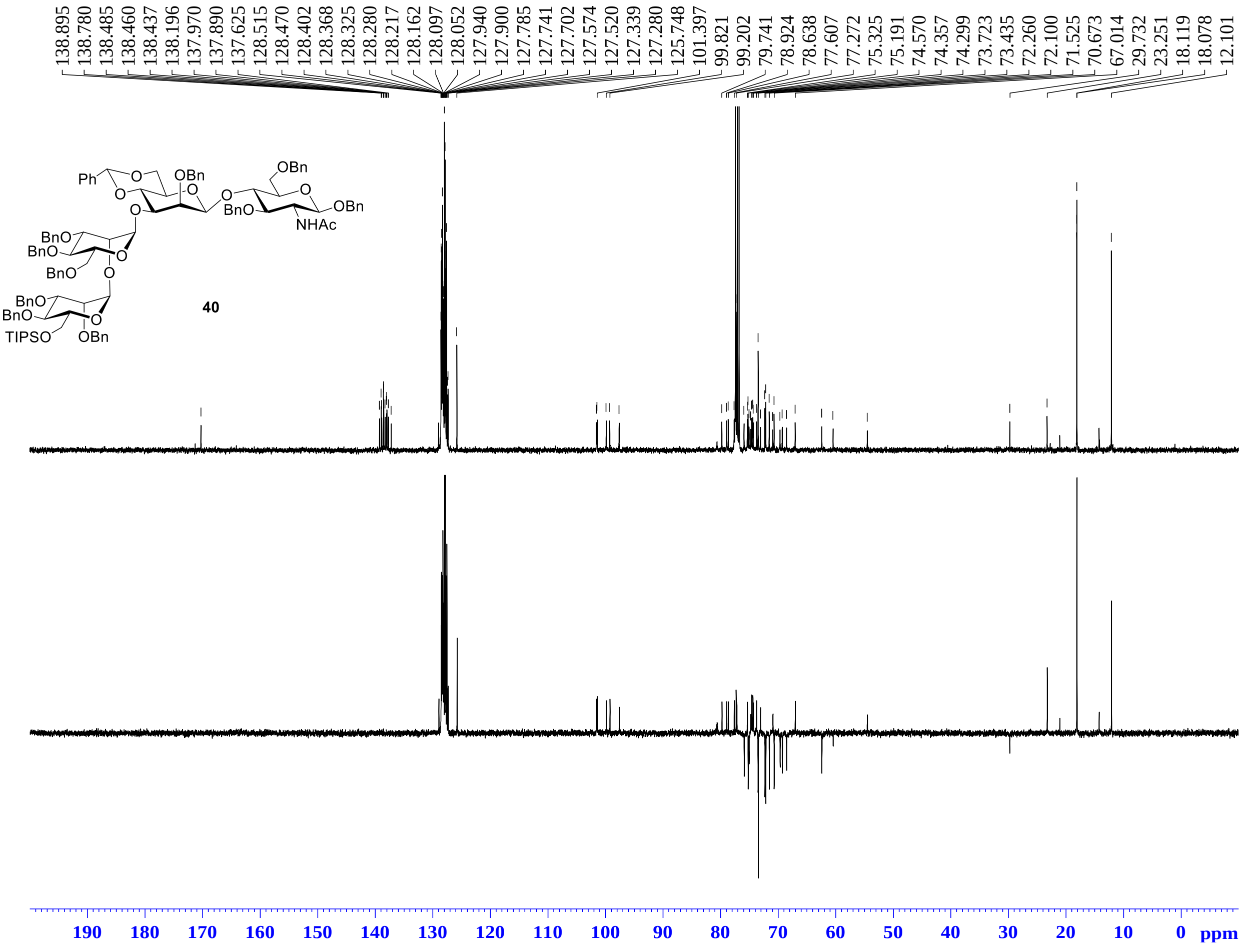

Compound 40: ^{13}C and Dept-135 NMR (CDCl$_3$, 100 MHz)

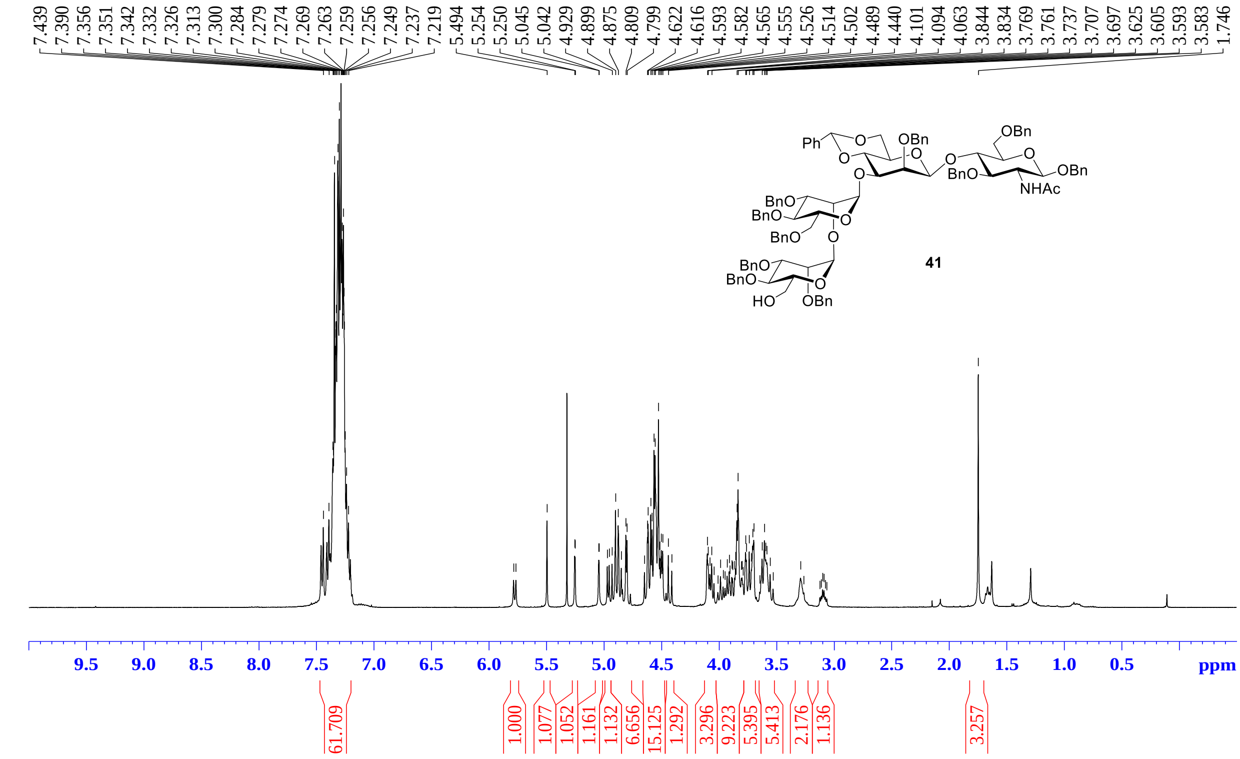

Compound 41: ^{1}H NMR (CDCl$_3$, 400 MHz)

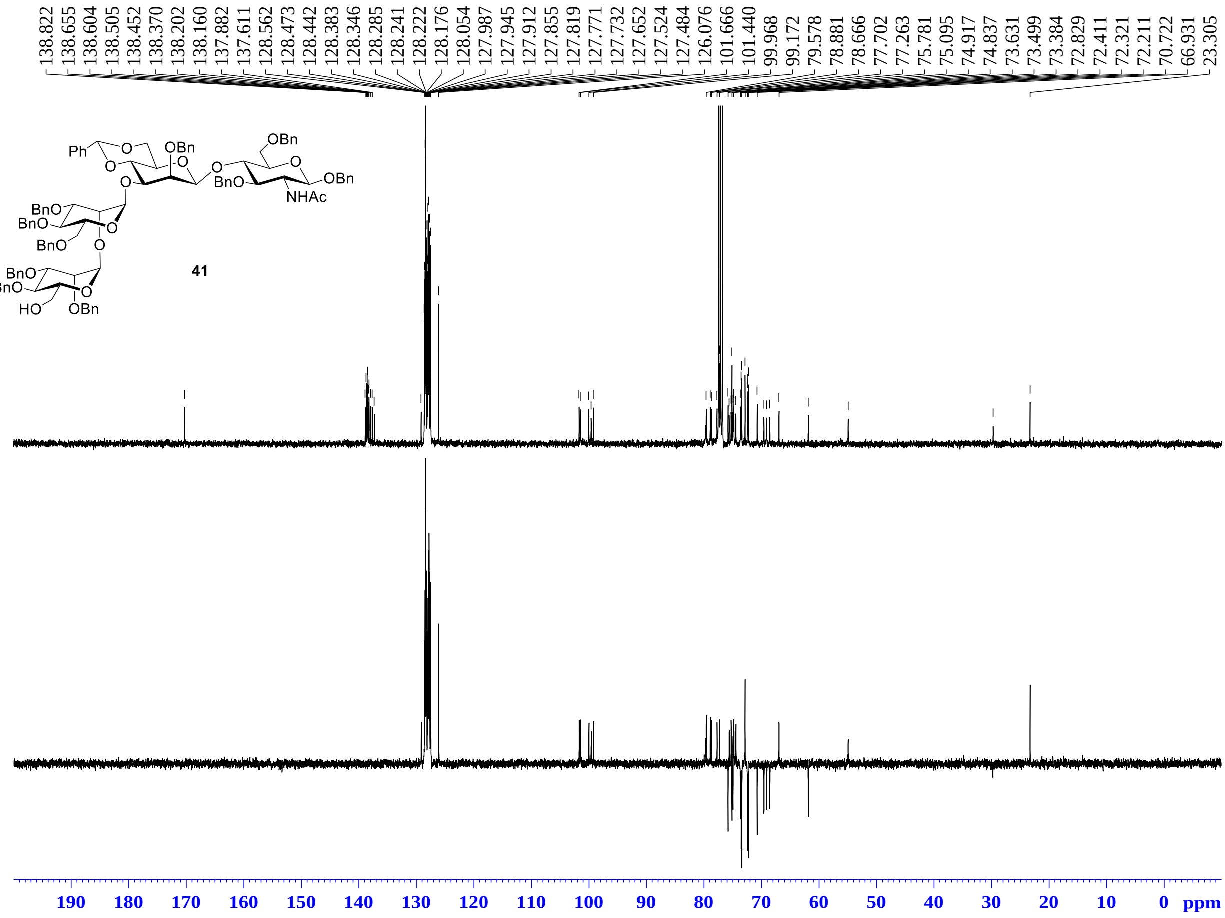

Compound 41: ^{13}C and Dept-135 NMR ($CDCl_3$, 100 MHz)

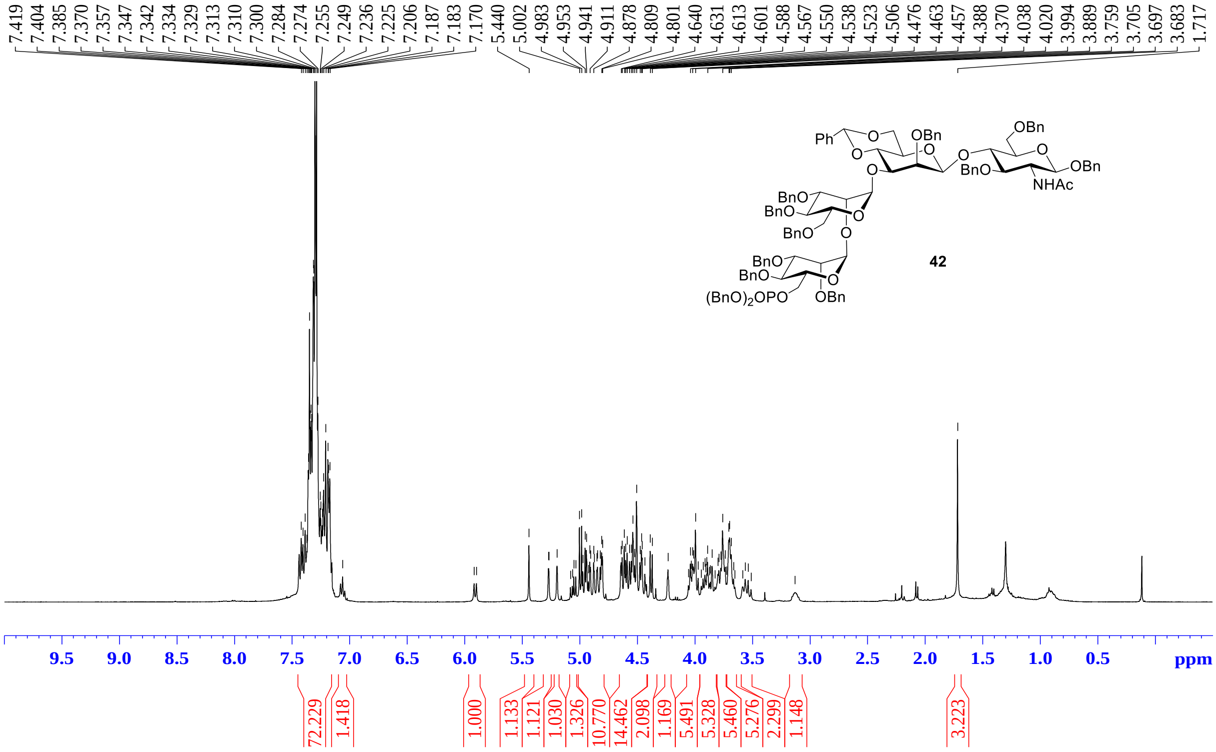

Compound 42: ^{1}H NMR ($CDCl_3$, 400 MHz)

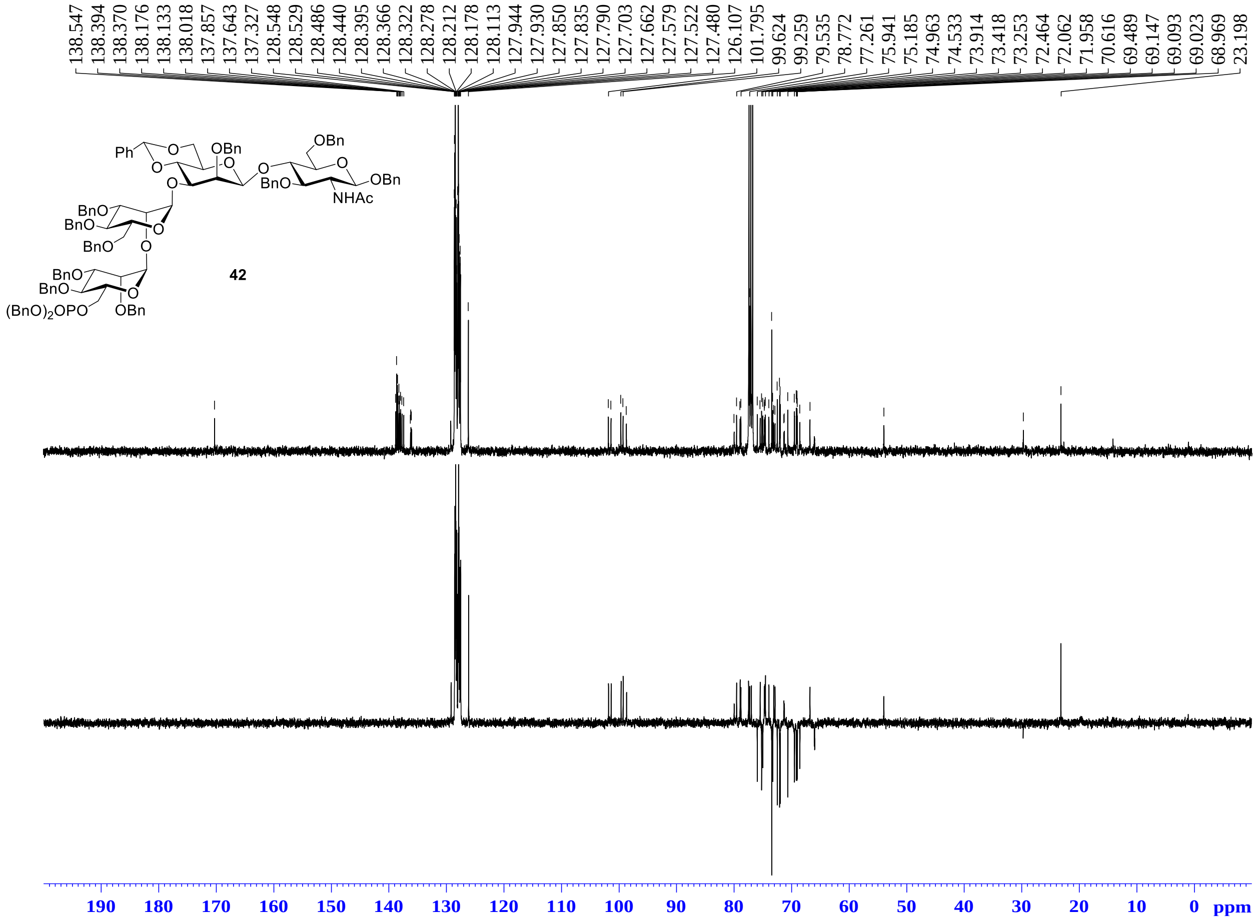

Compound 42: ^{13}C and Dept-135 NMR ($CDCl_3$, 100 MHz)

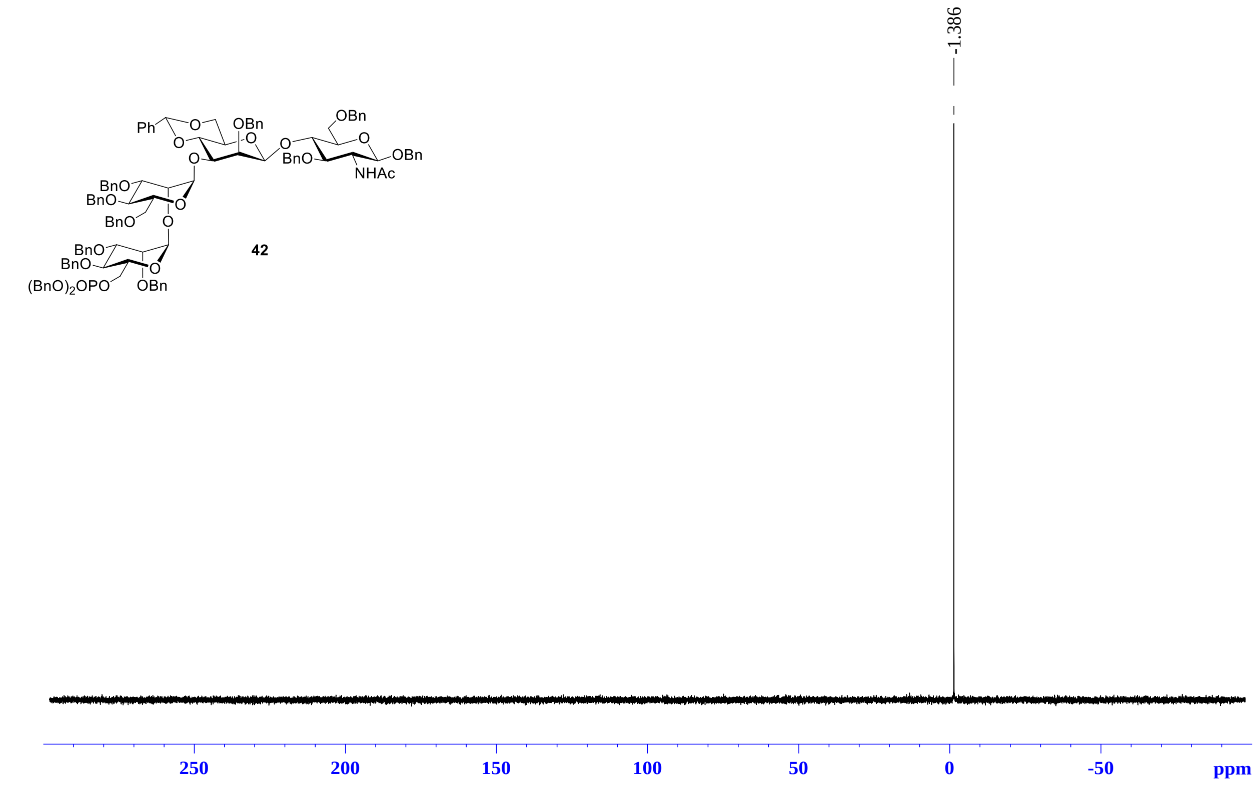

Compound 42: ^{31}P NMR ($CDCl_3$, 146 MHz)

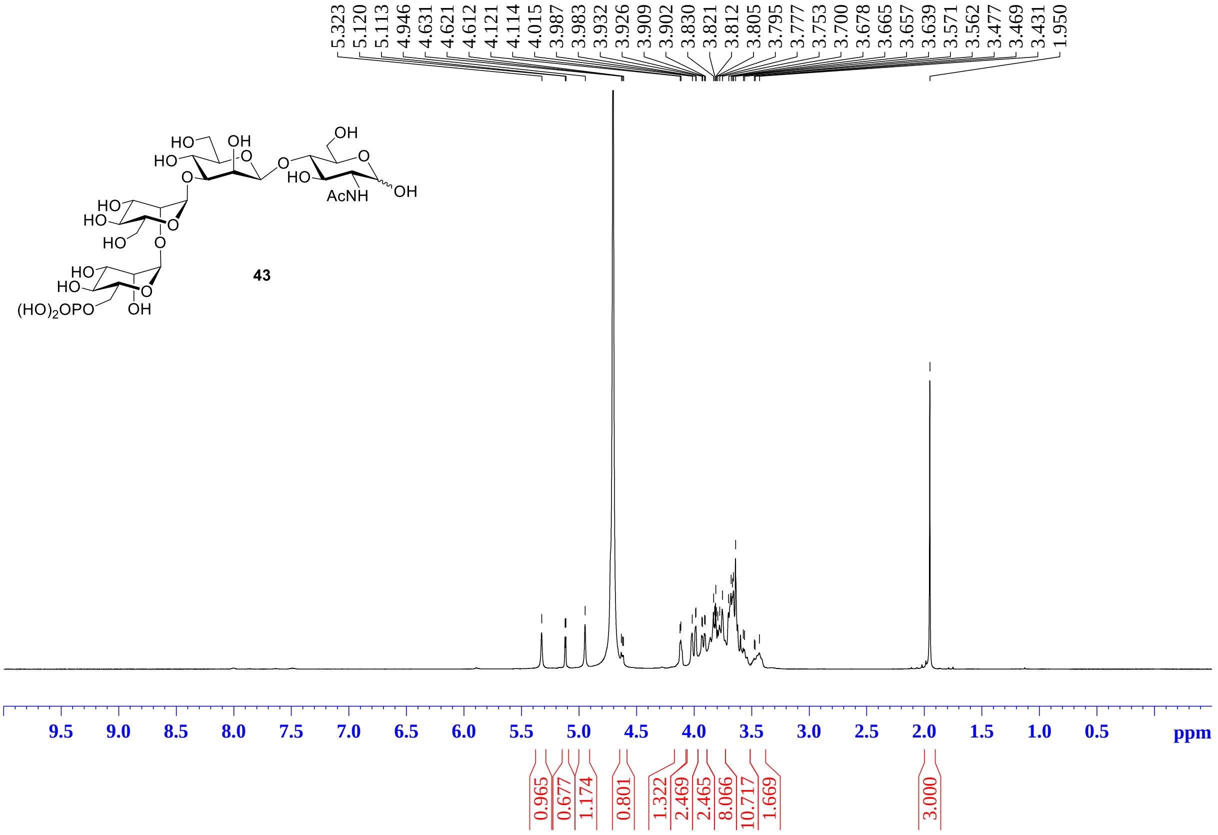

Compound 43: ^{1}H NMR (D_2O, 400 MHz)

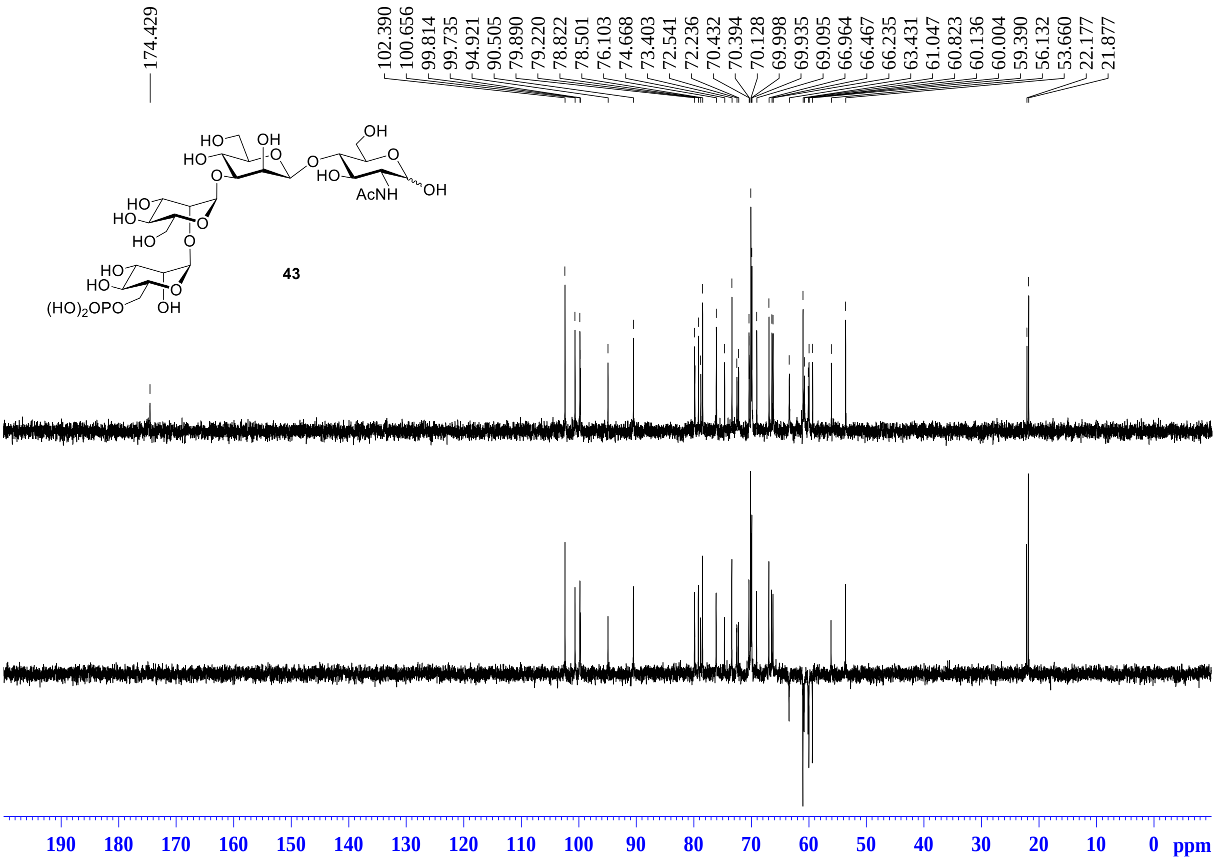

Compound 43: ^{13}C and Dept-135 NMR (D_2O, 100 MHz)

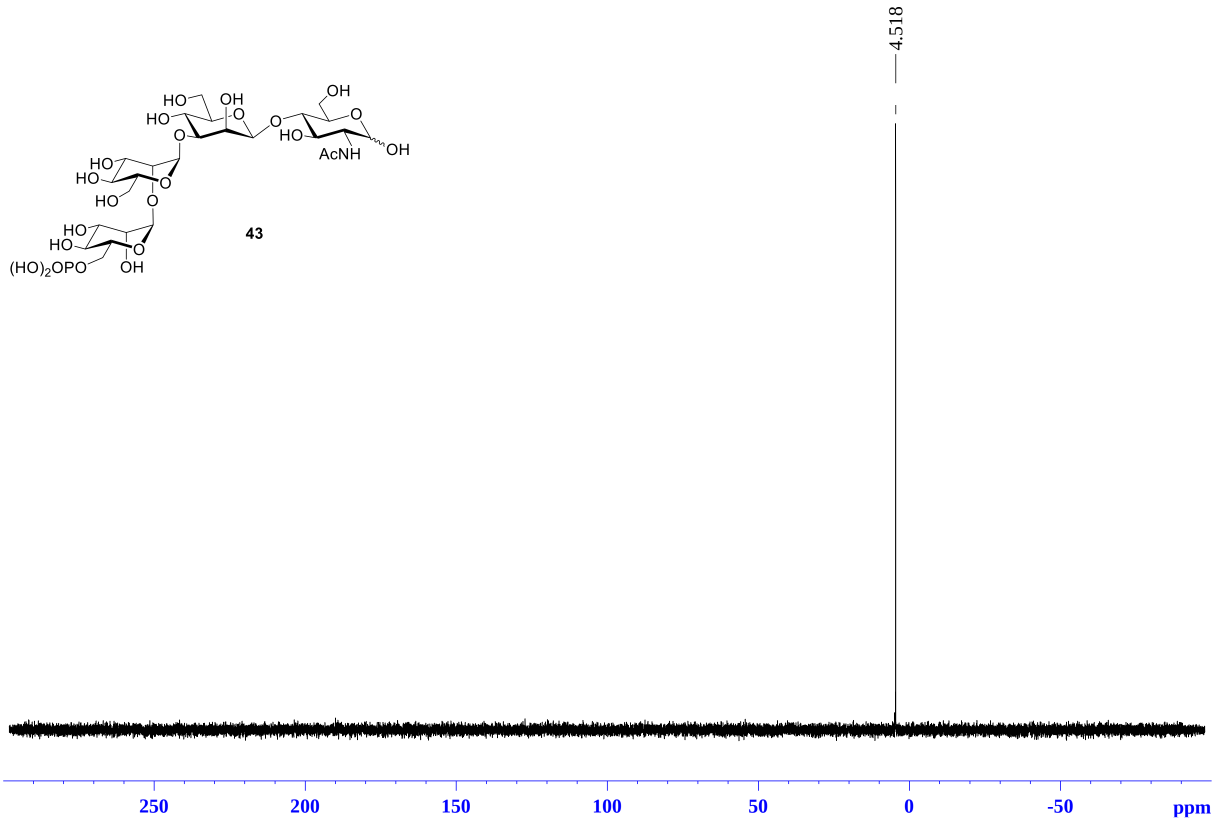

Compound 43: ^{31}P NMR (D_2O, 146 MHz)

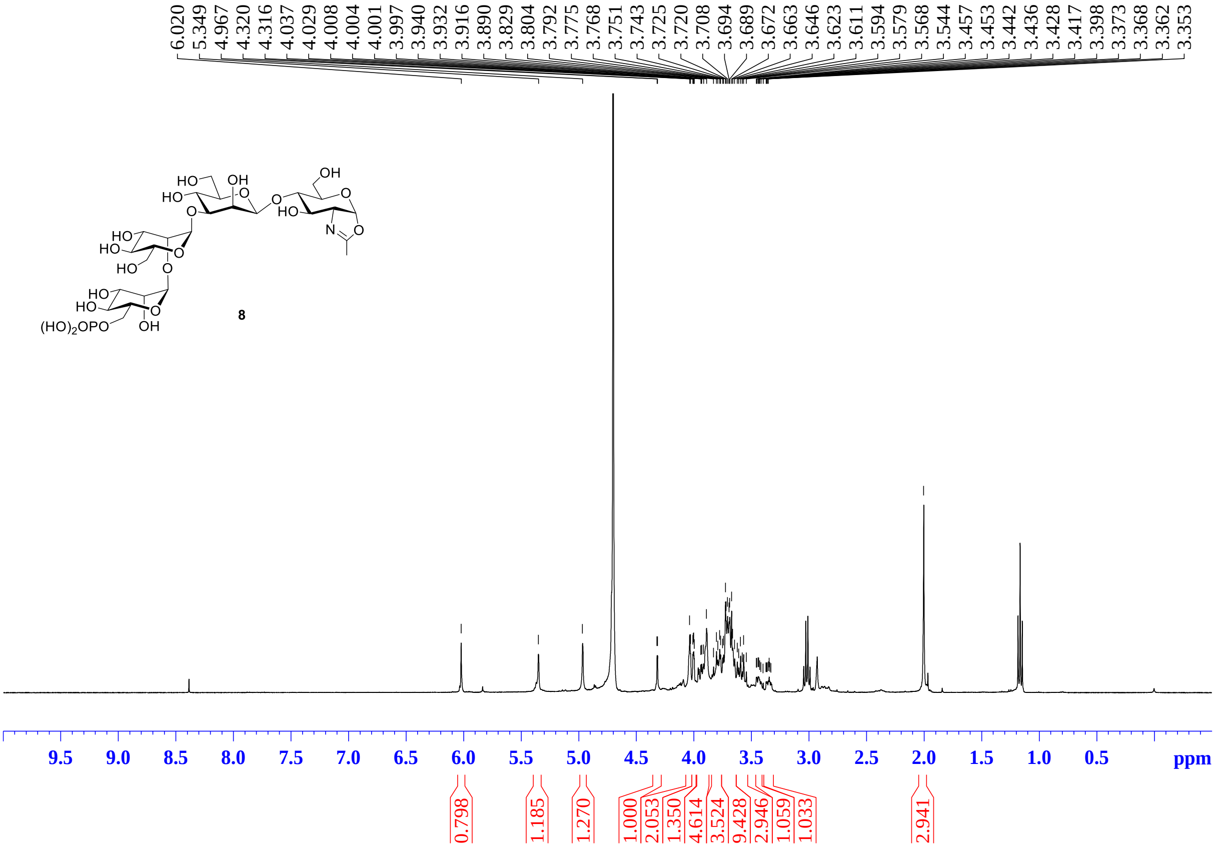

Compound 8: ^{1}H NMR (D_2O, 400 MHz)

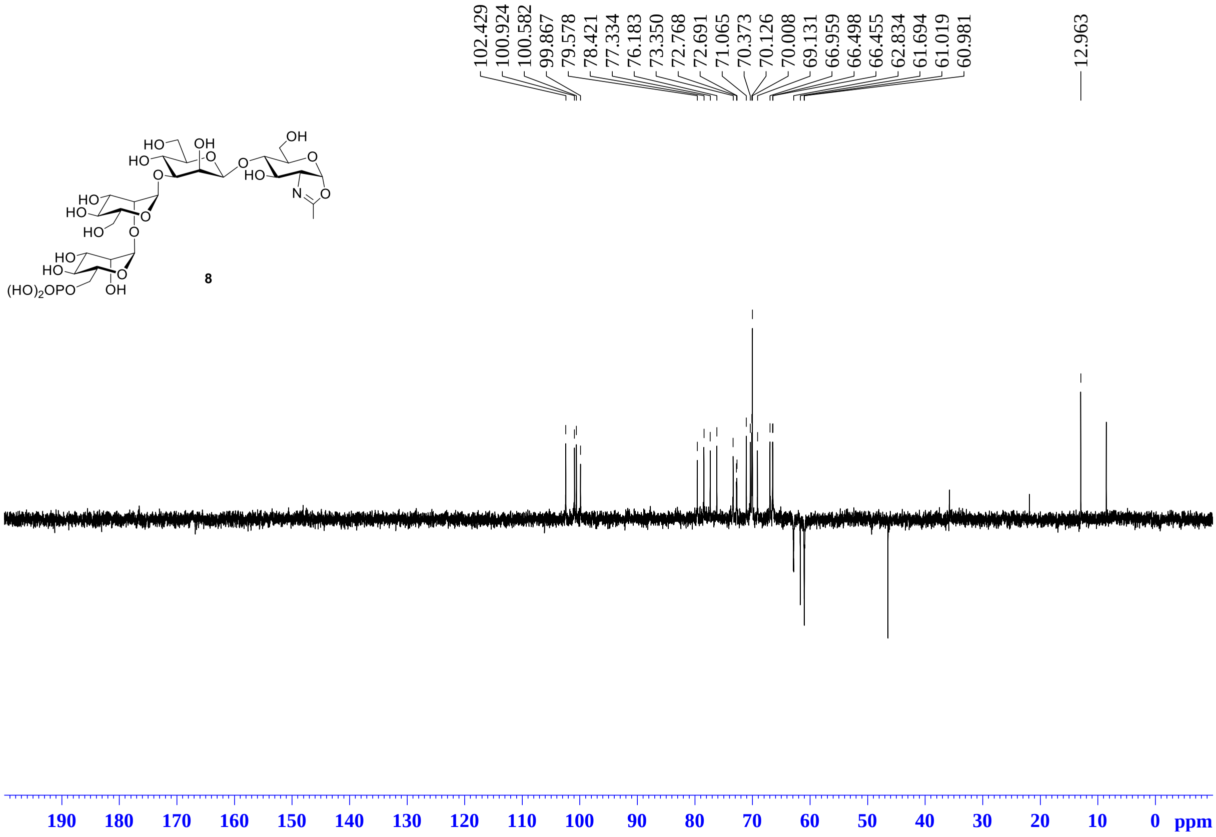

Compound 8: Dept-135 NMR (D_2O, 100 MHz)

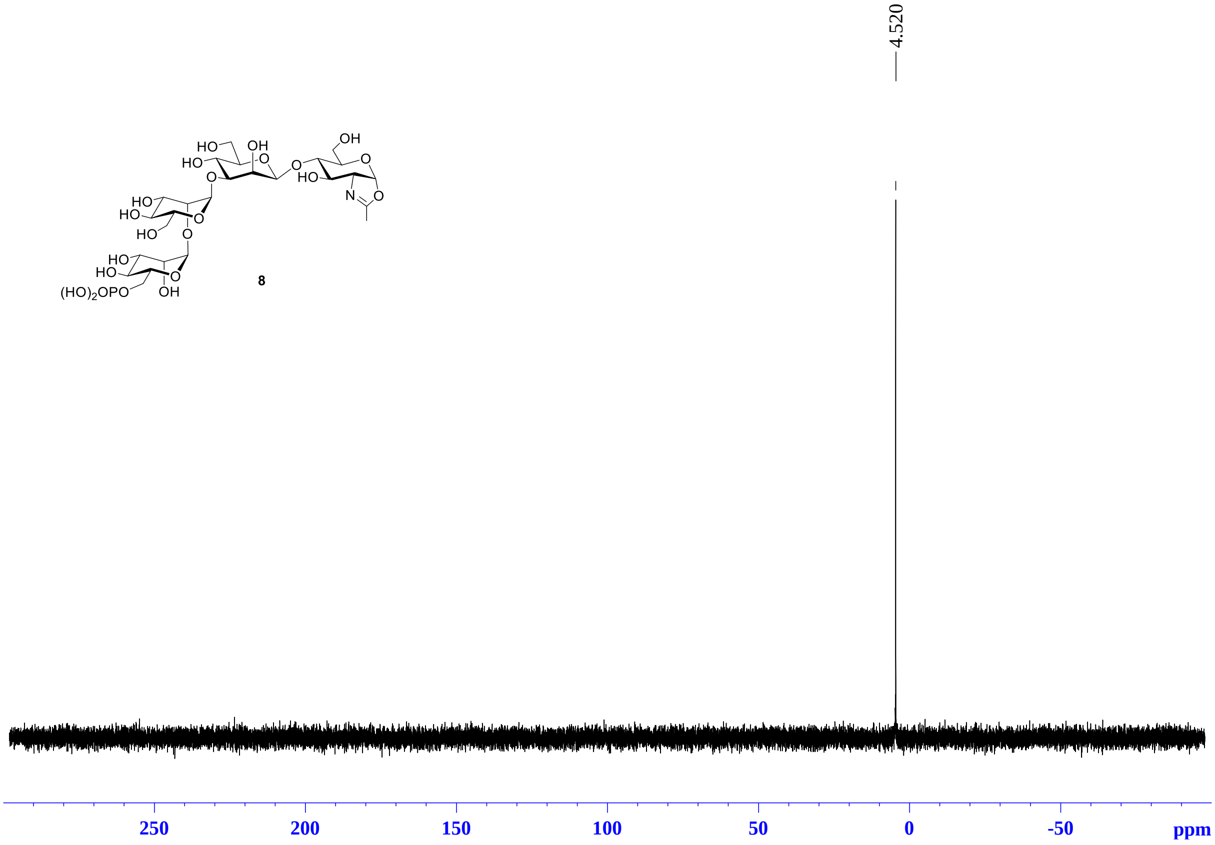

Compound 8: ^{31}P NMR (D_2O, 146 MHz)

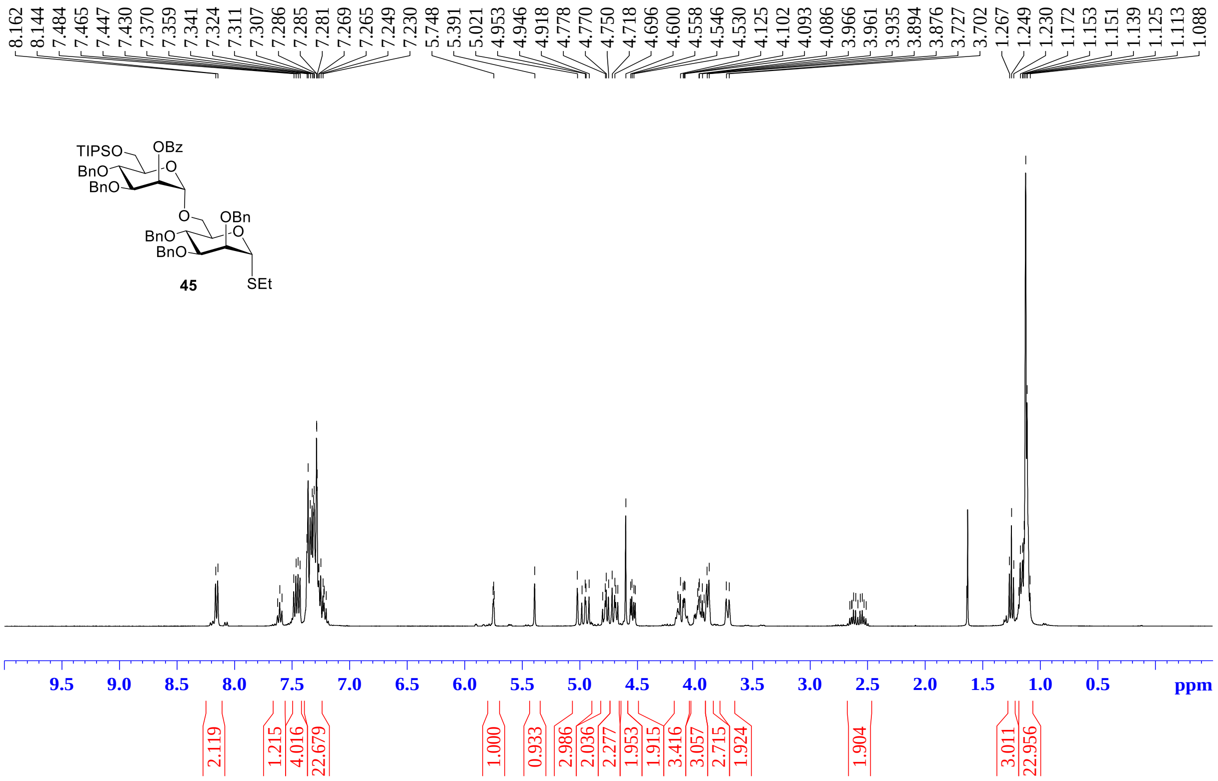

Compound 45: ^{1}H NMR ($CDCl_3$, 400 MHz)

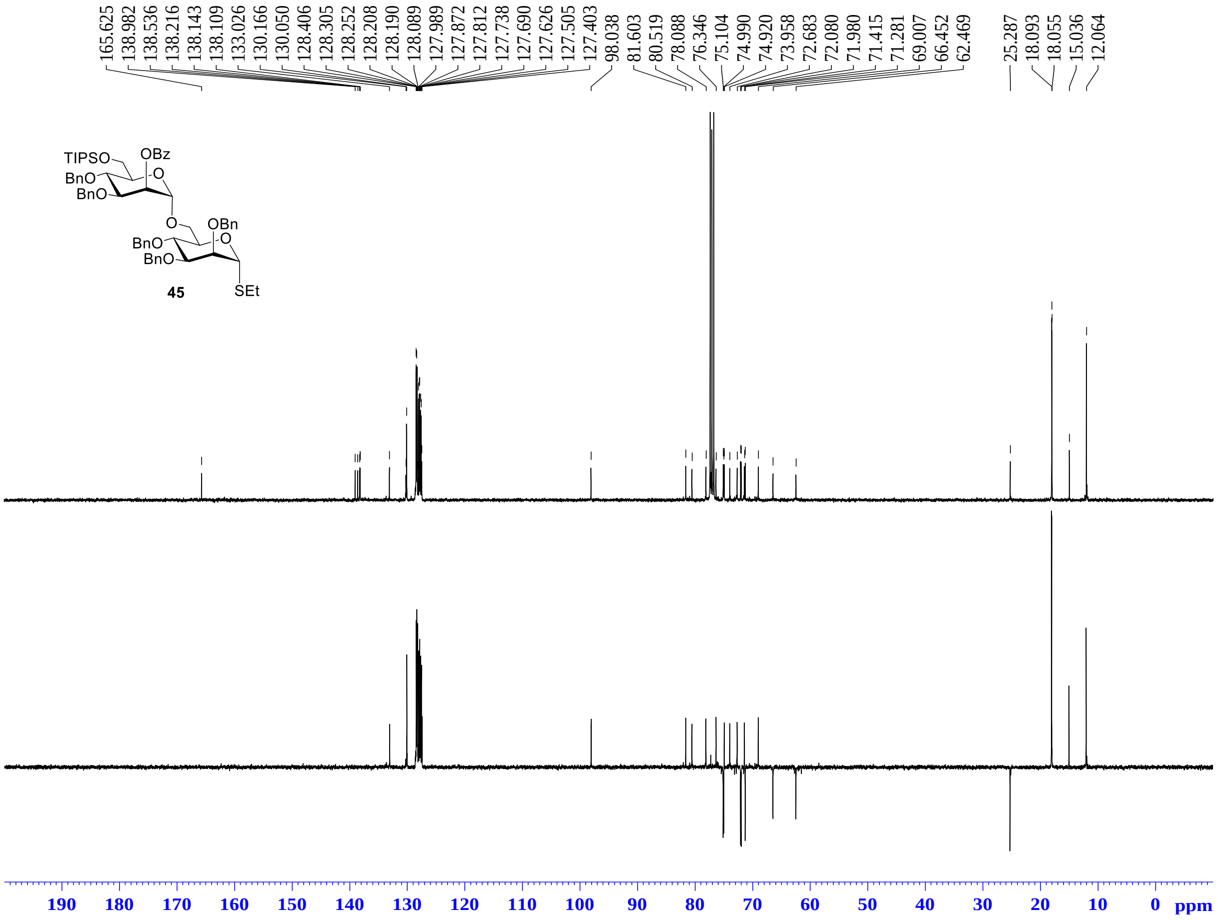

Compound 45: ^{13}C and Dept-135 NMR ($CDCl_3$, 100 MHz)

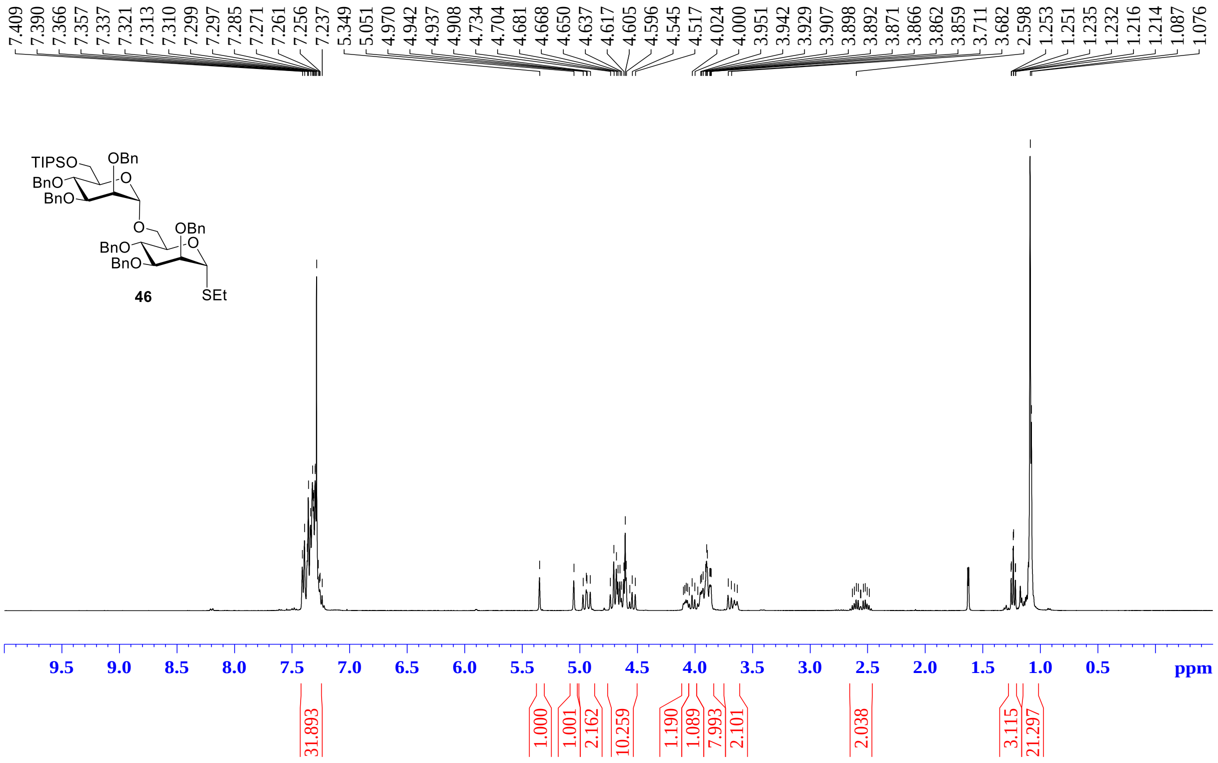

Compound 46: ^{1}H NMR (CDCl₃, 400 MHz)

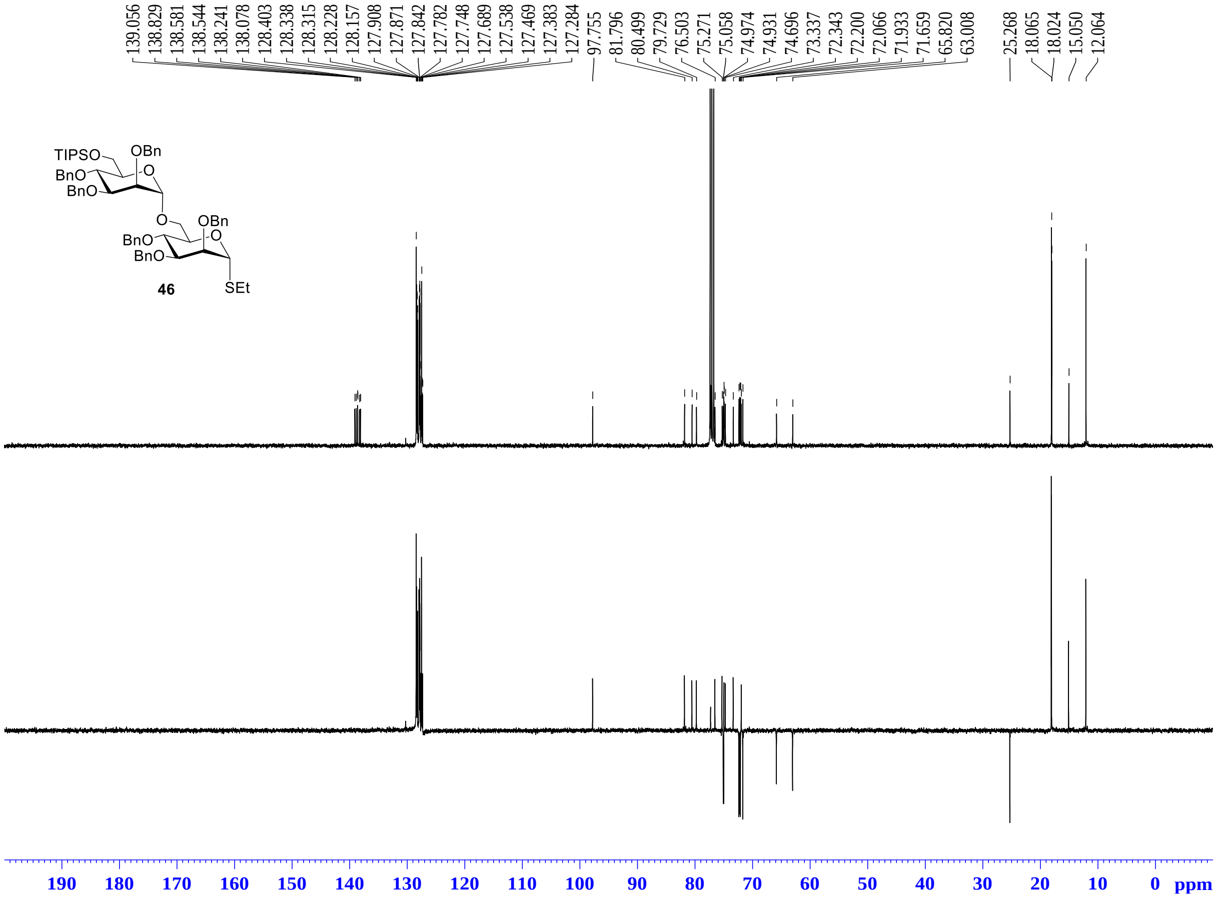

Compound 46: ^{13}C and Dept-135 NMR (CDCl₃, 100 MHz)

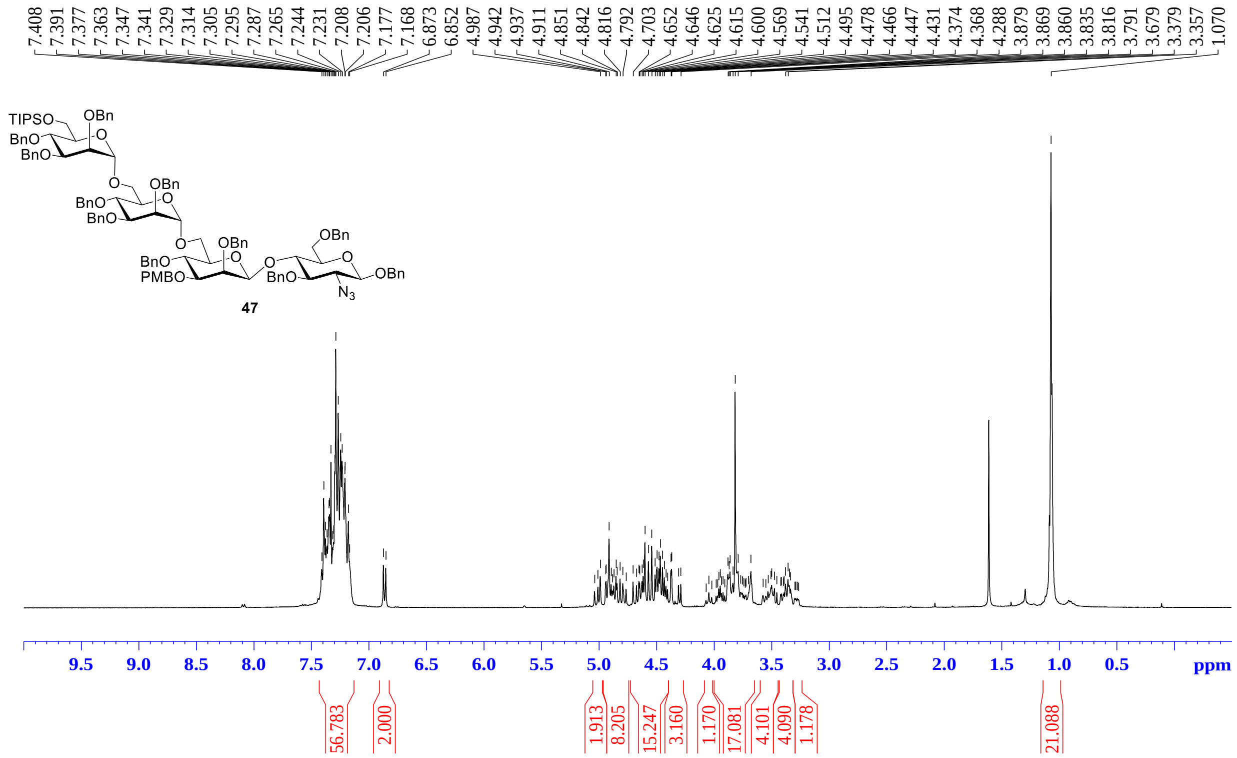

Compound 47: ^{1}H NMR ($CDCl_3$, 400 MHz)

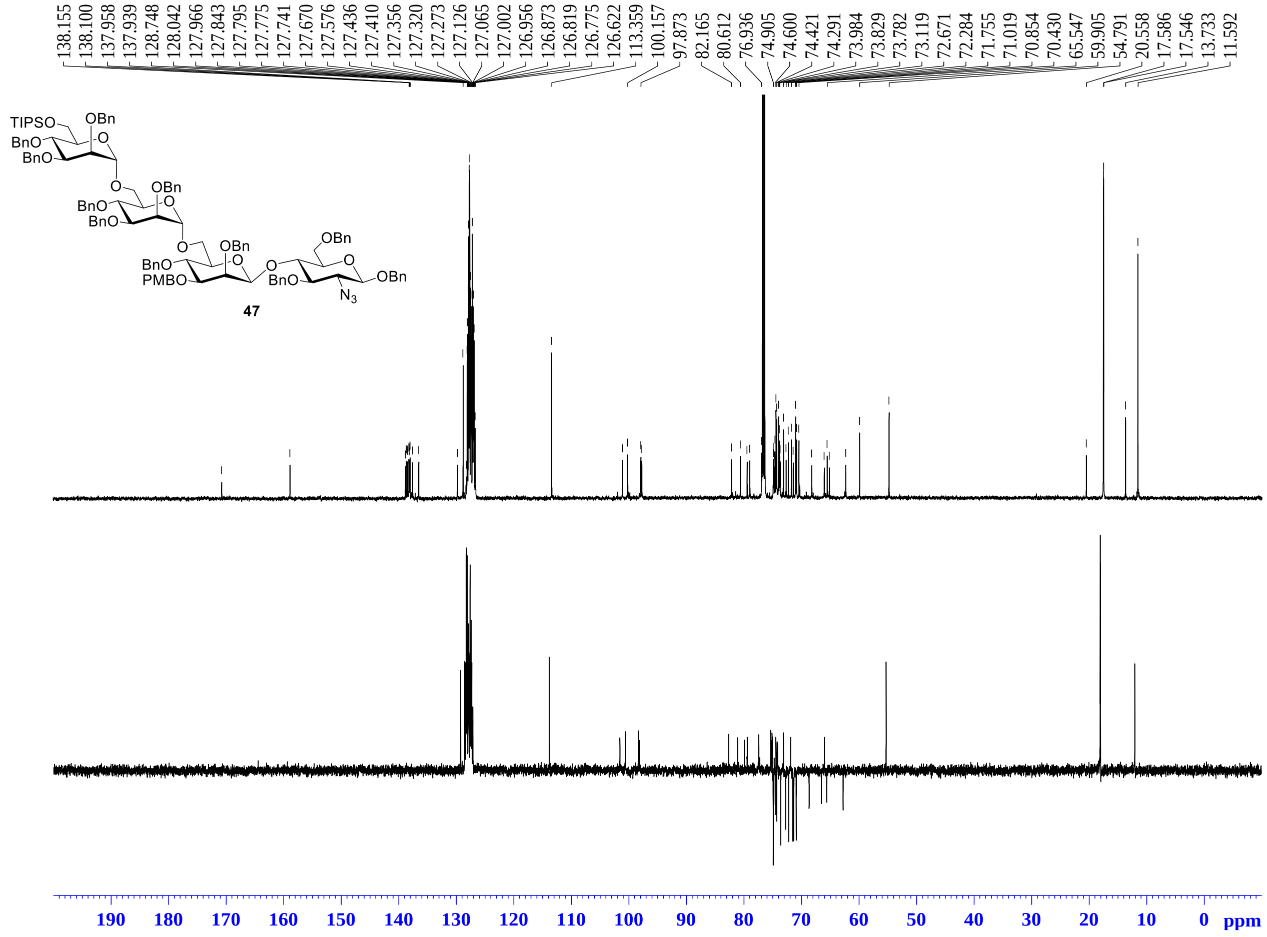

Compound 47: ^{13}C and Dept-135 NMR ($CDCl_3$, 100 MHz)

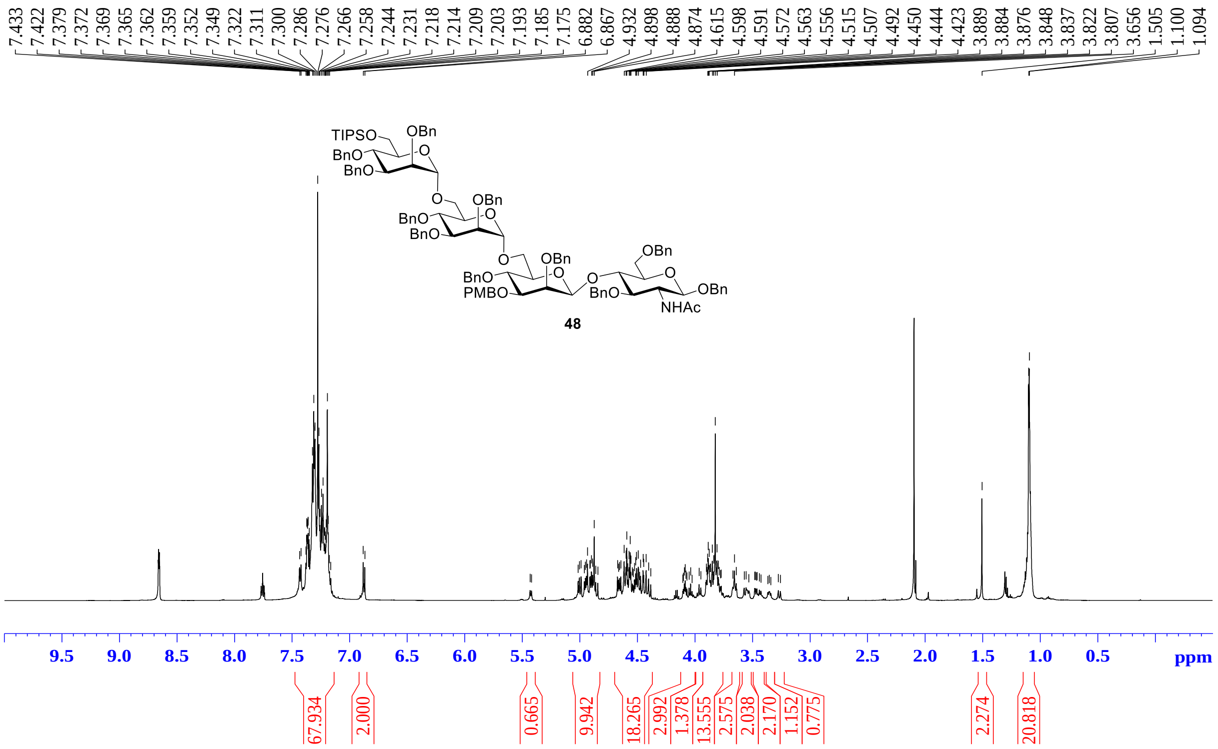

Compound 48: ^{1}H NMR ($CDCl_3$, 400 MHz)

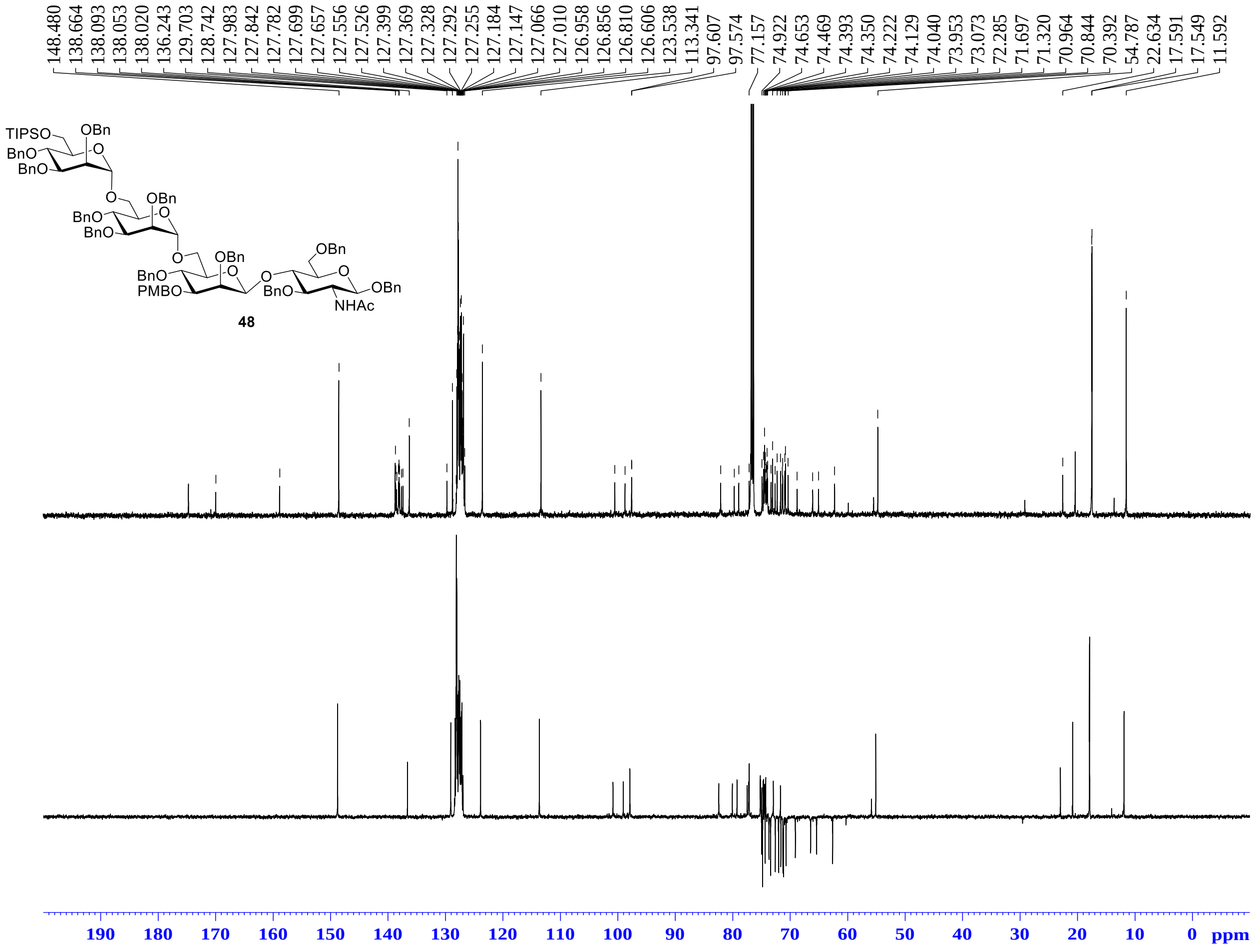

Compound 48: ^{13}C and Dept-135 NMR ($CDCl_3$, 100 MHz)

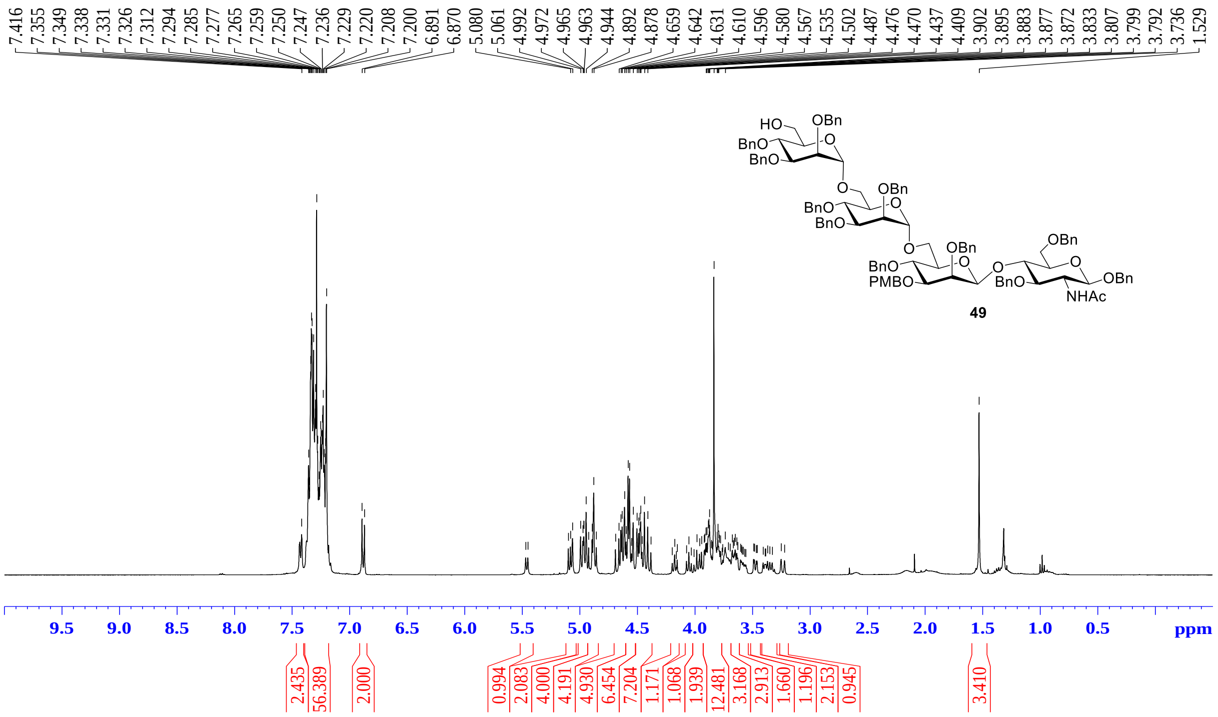

Compound 49: ^{1}H NMR ($CDCl_3$, 400 MHz)

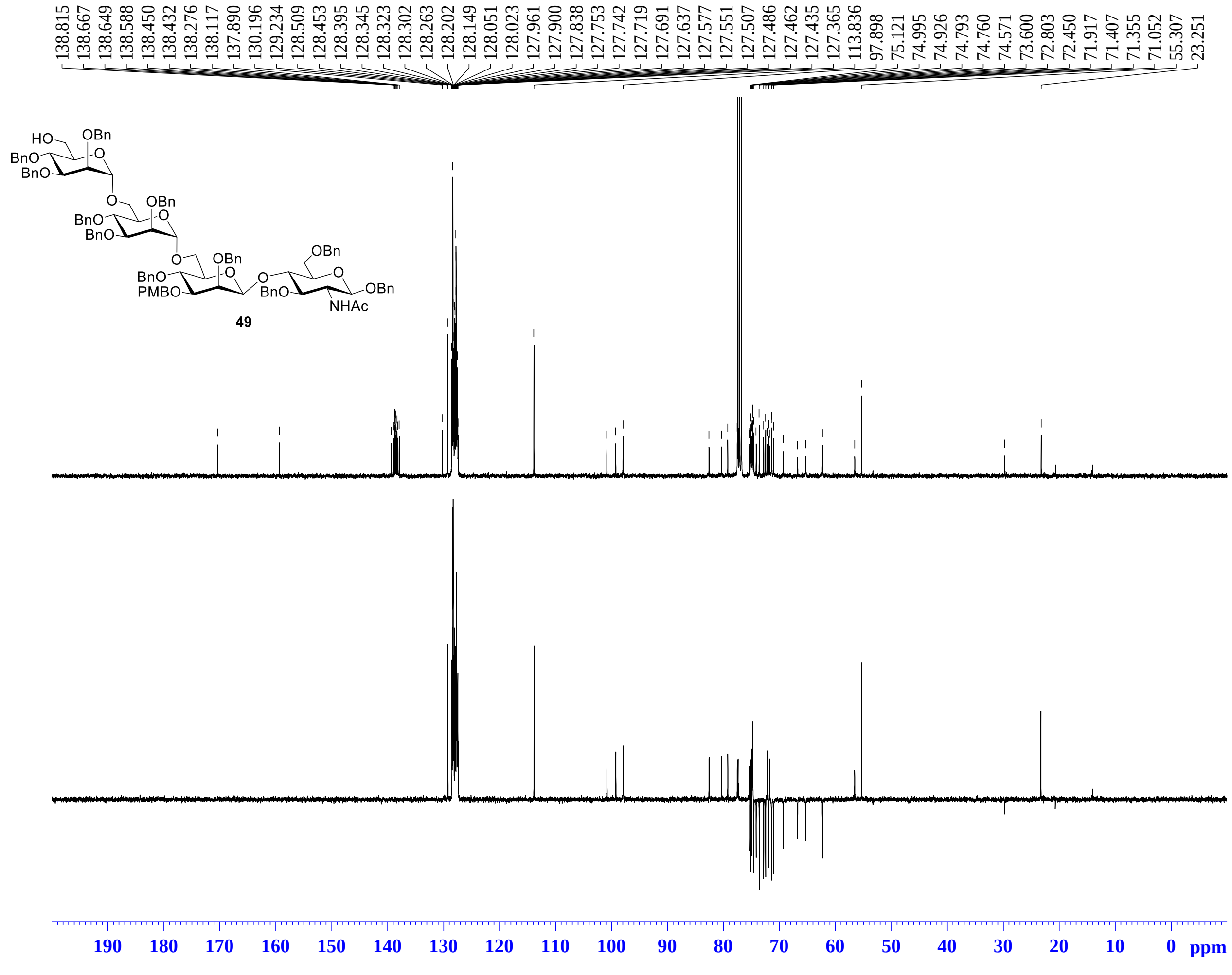

Compound 49: ^{13}C and Dept-135 NMR ($CDCl_3$, 100 MHz)

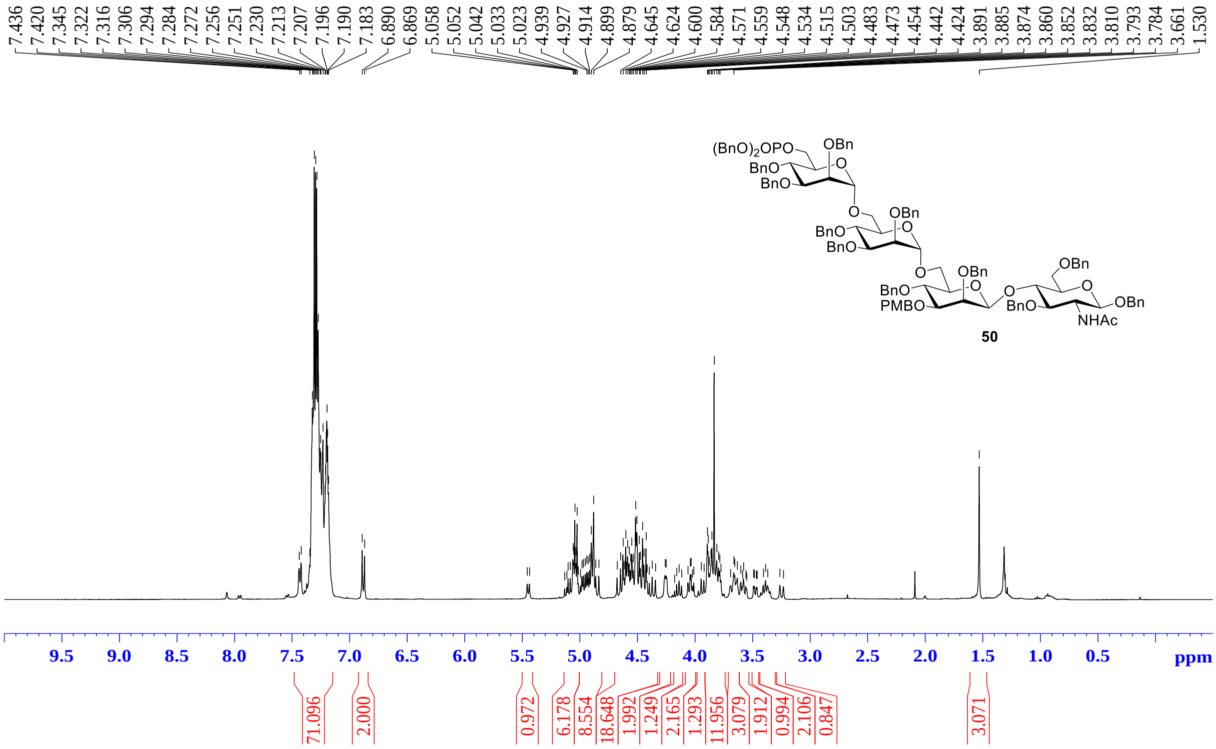

Compound 50: ^{1}H NMR ($CDCl_3$, 400 MHz)

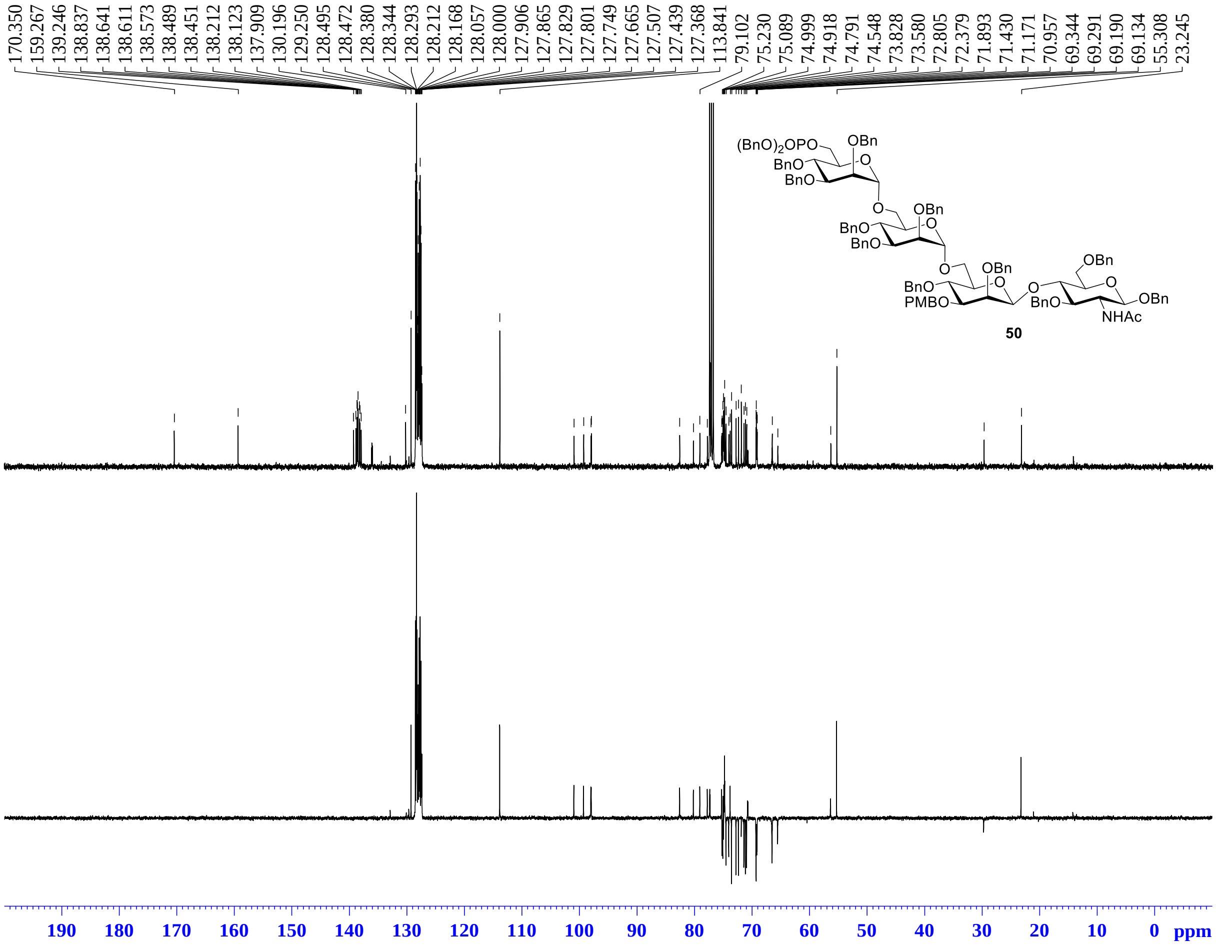

Compound 50: ^{13}C and Dept-135 NMR ($CDCl_3$, 100 MHz)

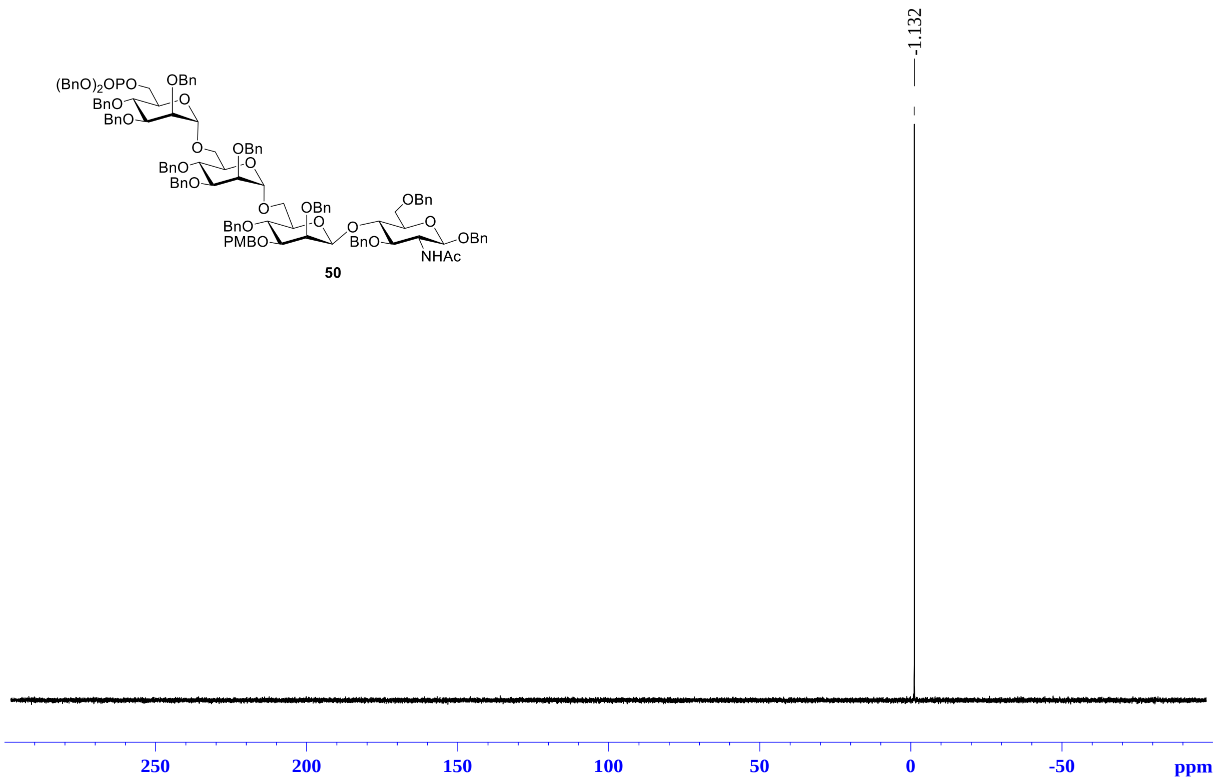

Compound 50: ^{31}P NMR (CDCl$_3$, 146 MHz)

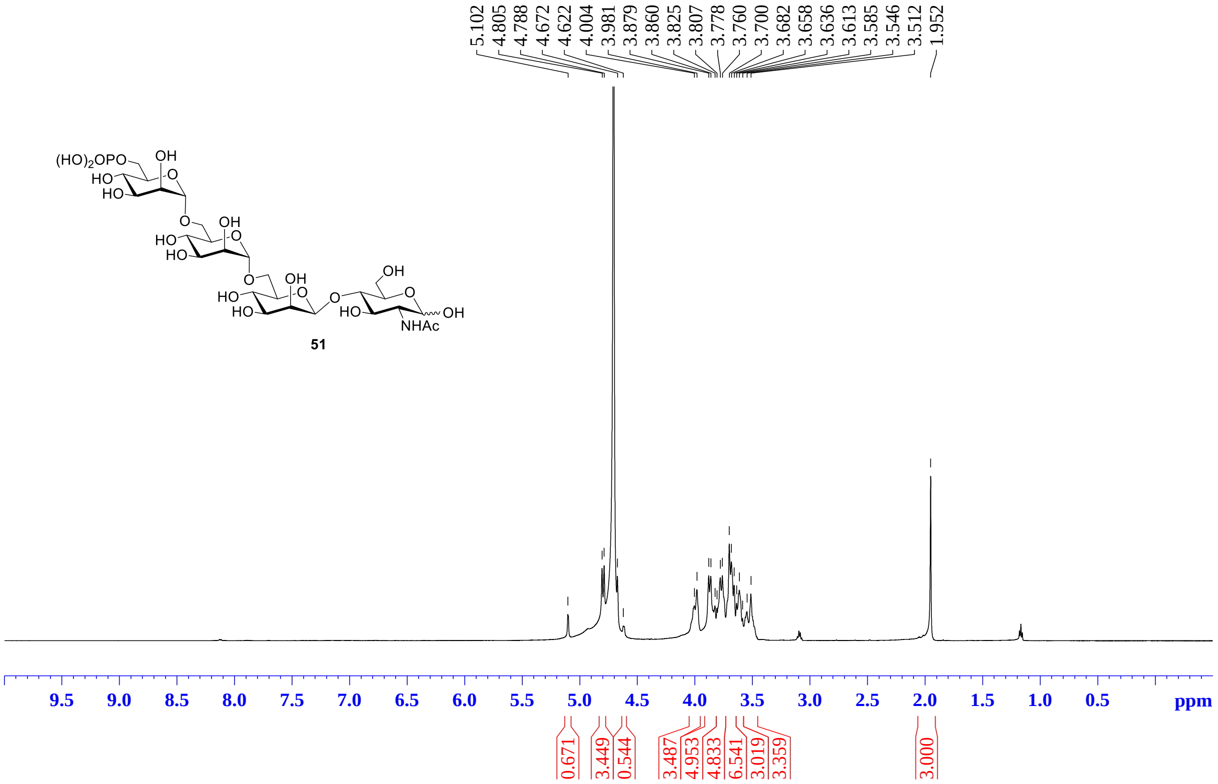

Compound 51: ^{1}H NMR (D$_2$O, 400 MHz)

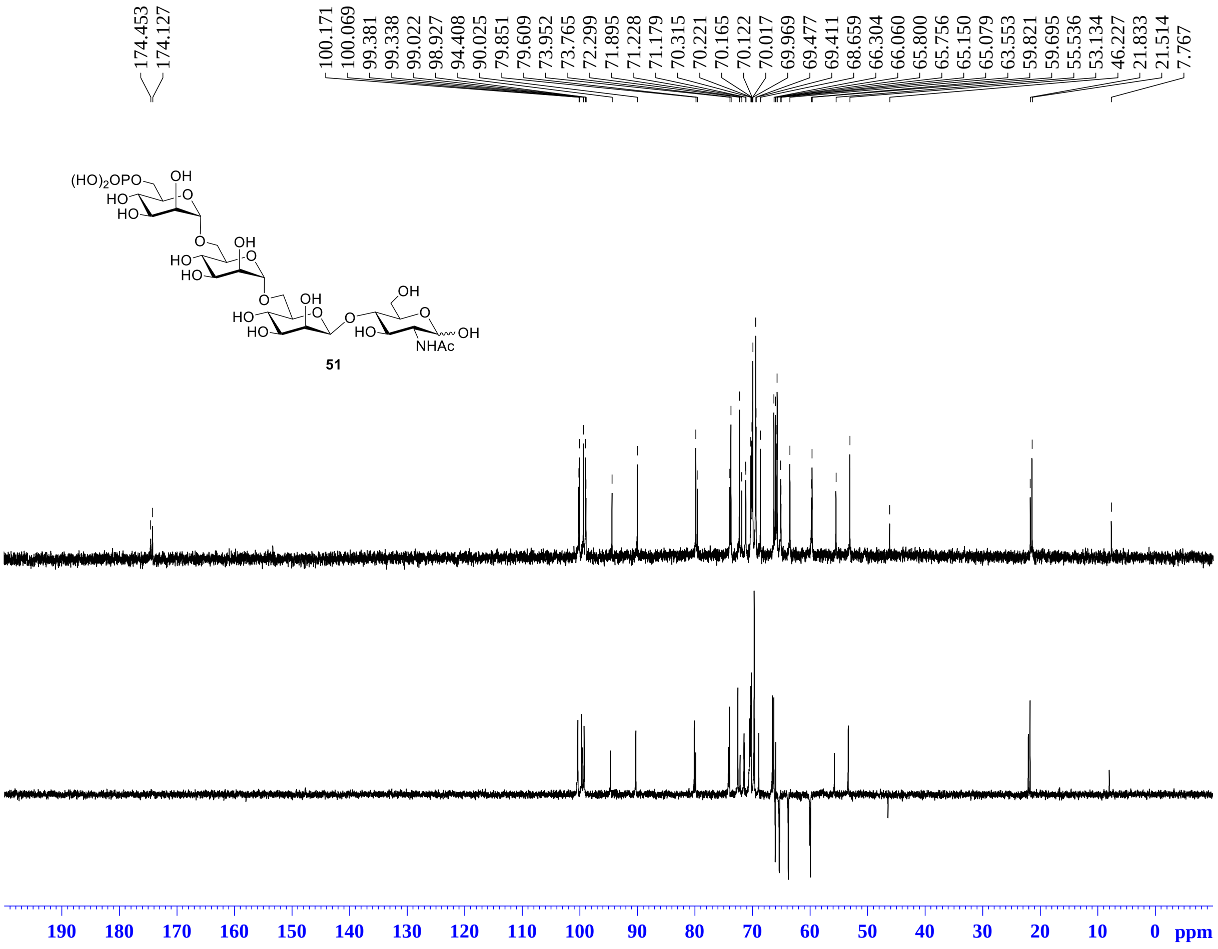

Compound 51: ^{13}C and Dept-135 NMR (D_2O, 100 MHz)

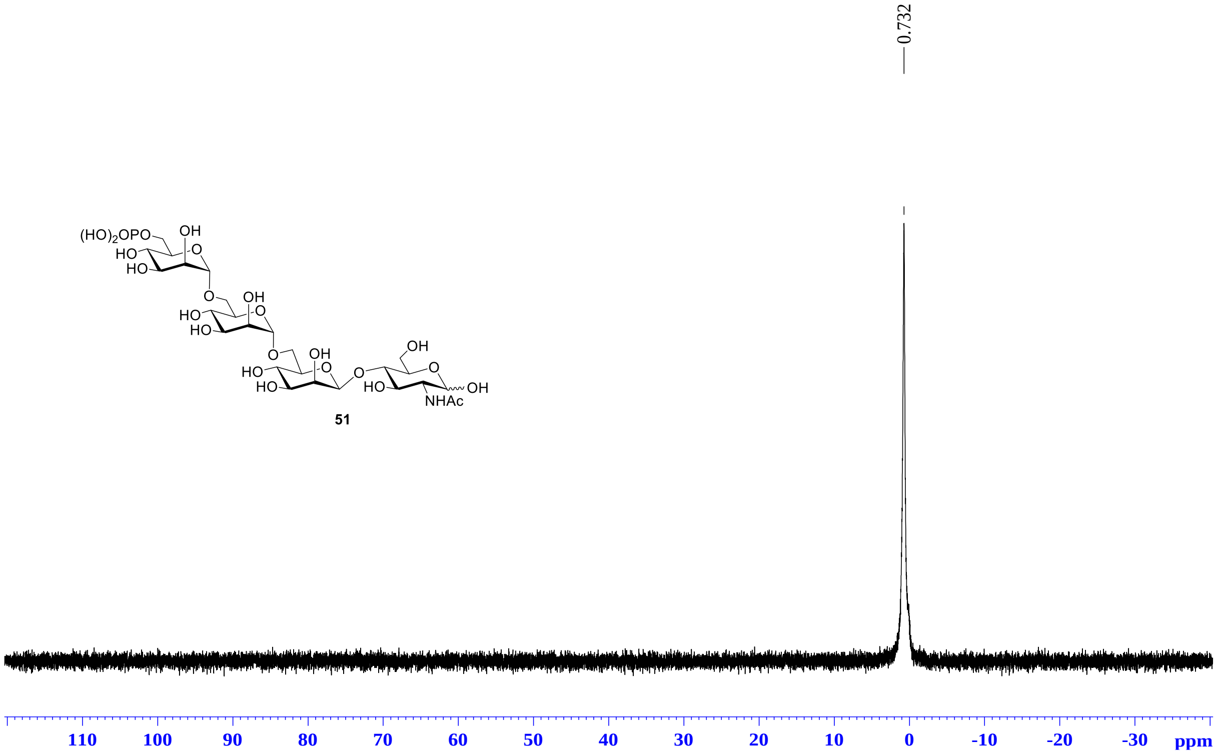

Compound 51: ^{31}P NMR (D_2O, 146 MHz)

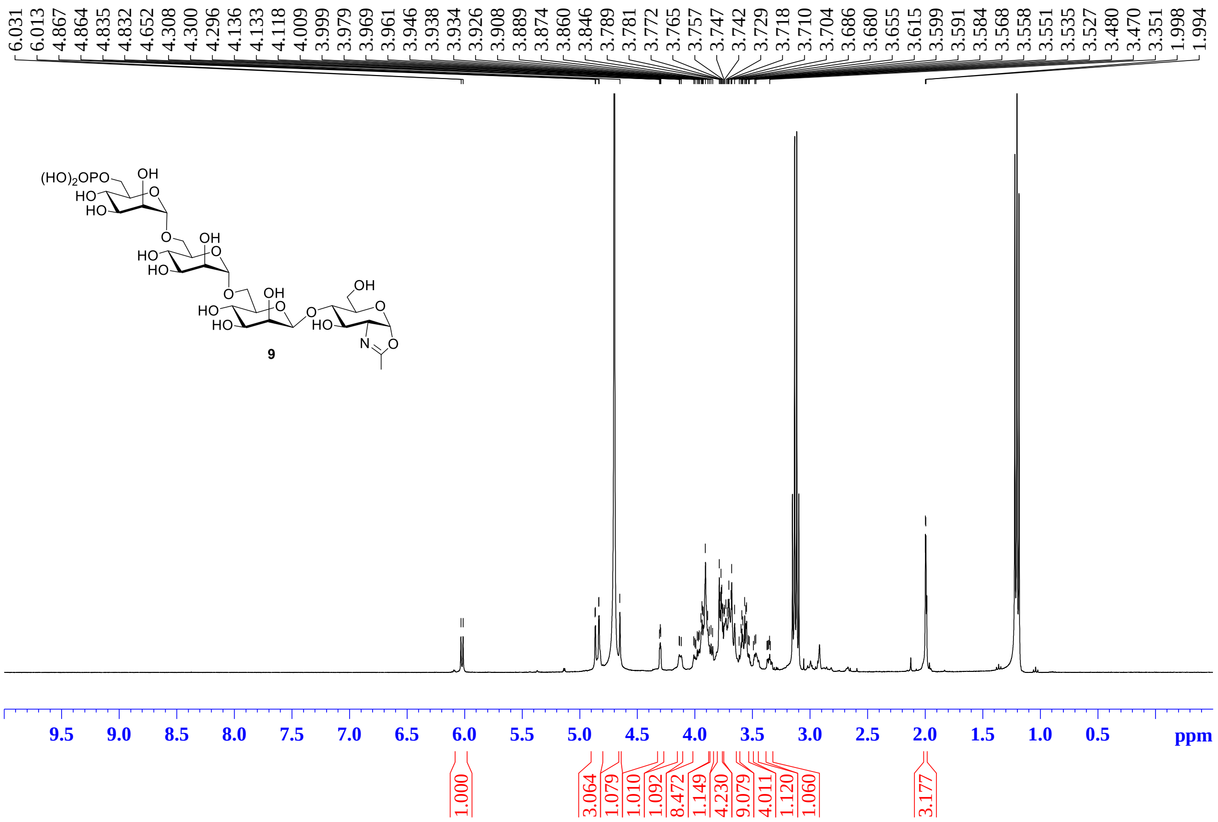

Compound 9: ^{1}H NMR (D_2O, 400 MHz)

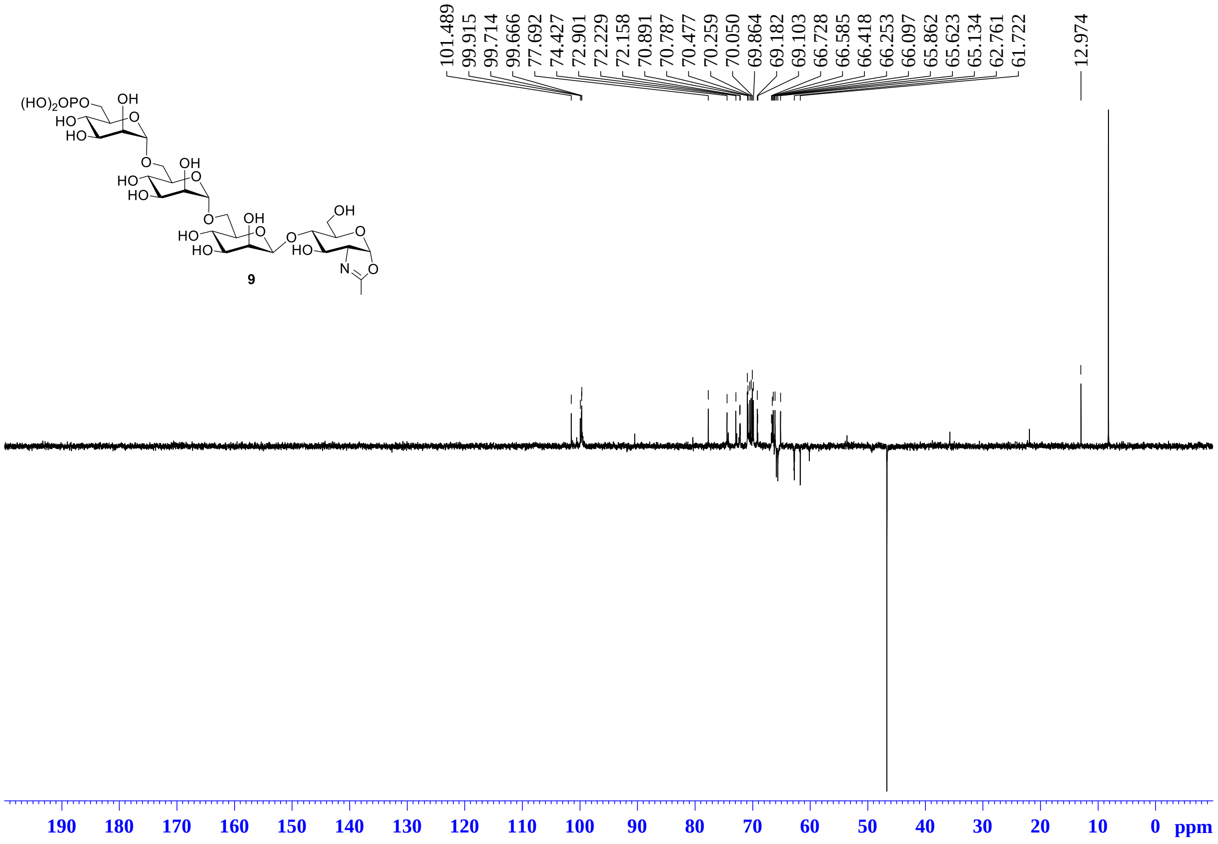

Compound 9: Dept-135 NMR (D_2O, 100 MHz)

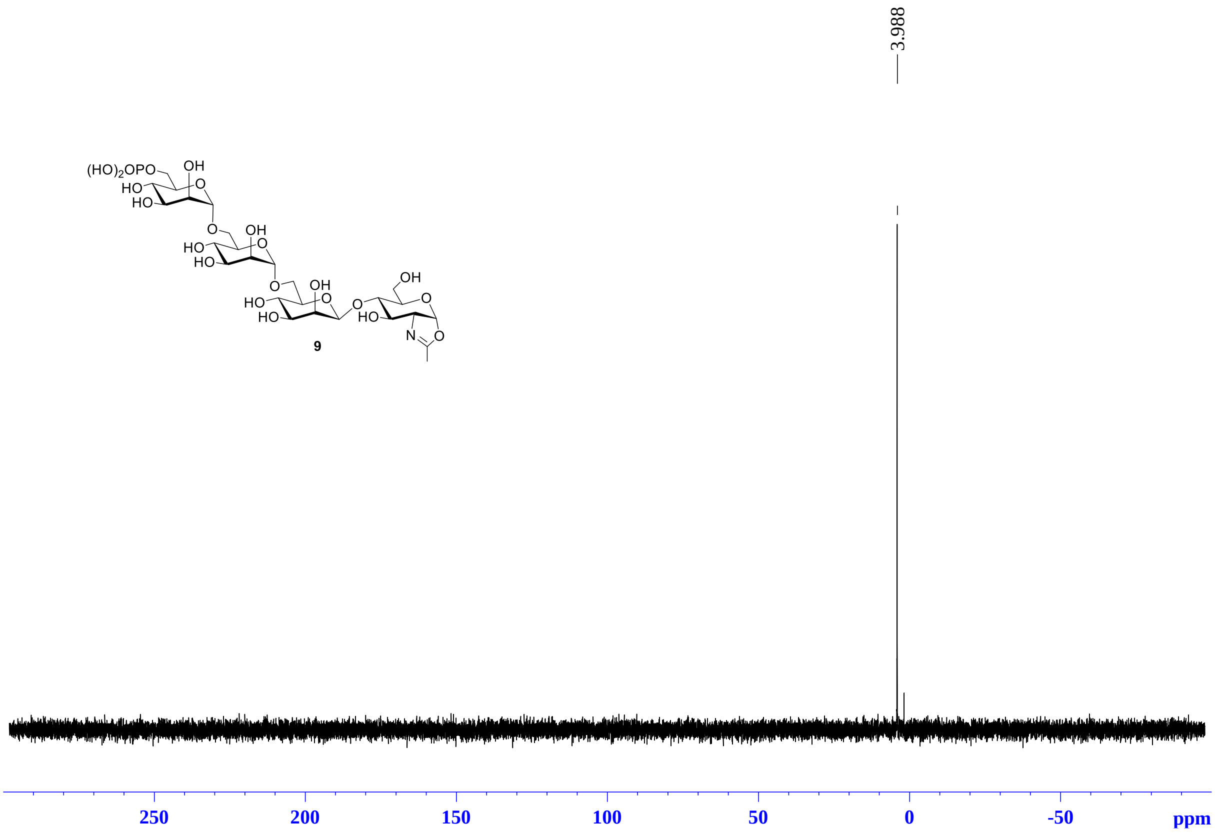

Compound 9: ^{31}P NMR (D_2O, 146 MHz)

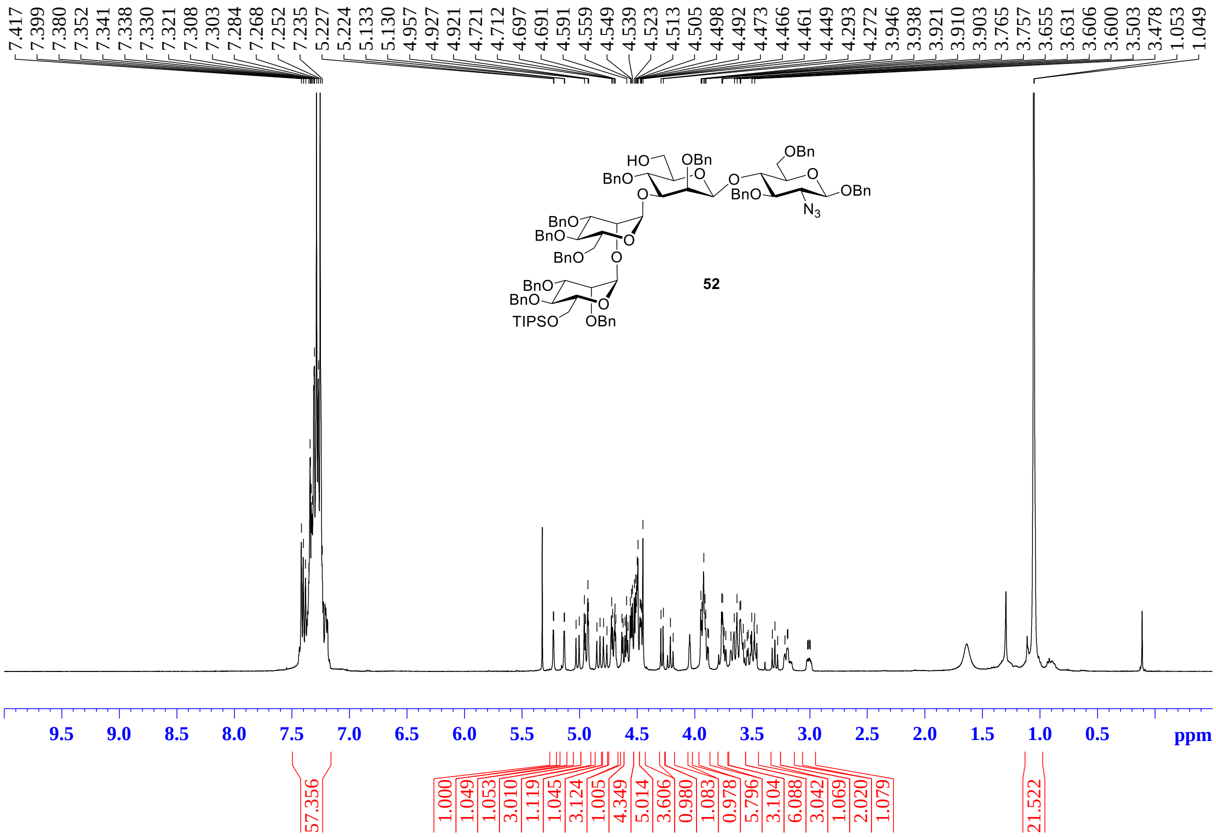

Compound 52: ^{1}H NMR ($CDCl_3$, 400 MHz)

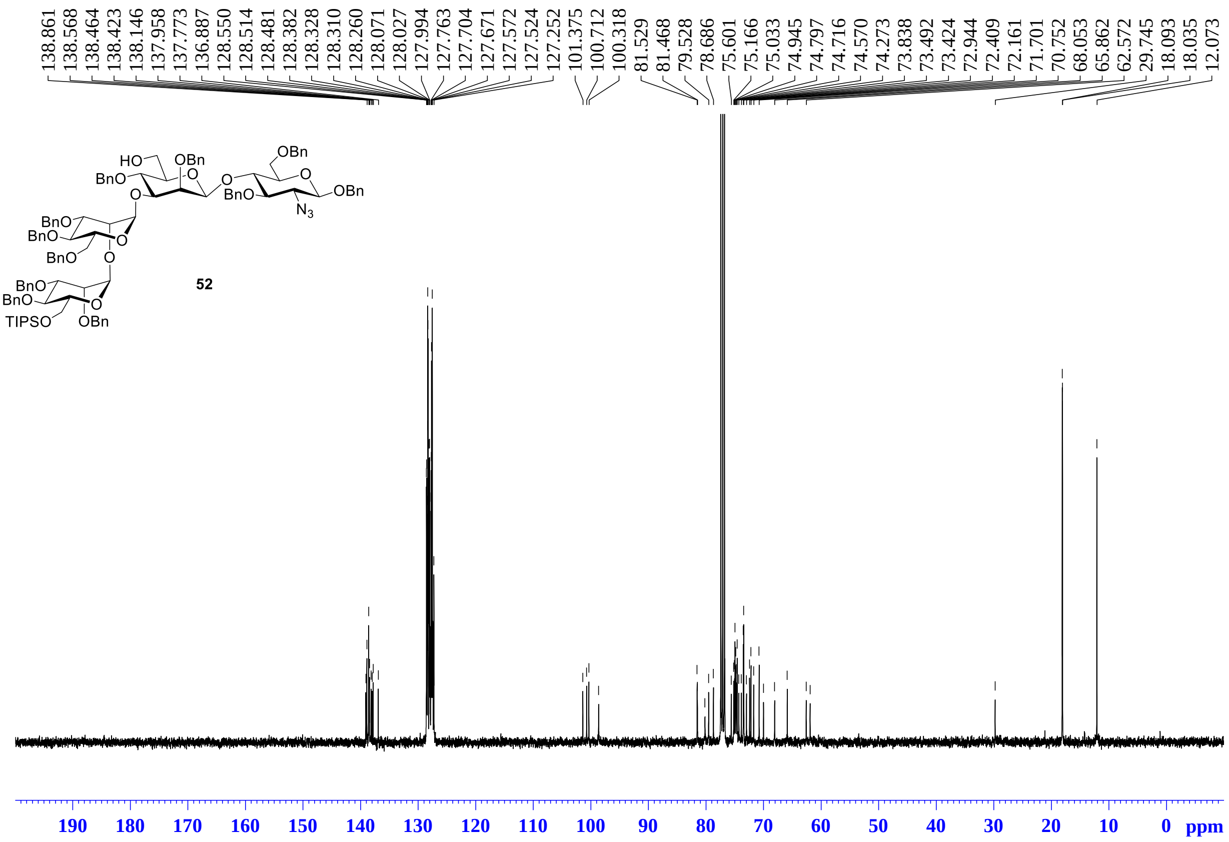

Compound 52: ^{13}C NMR ($CDCl_3$, 100 MHz)

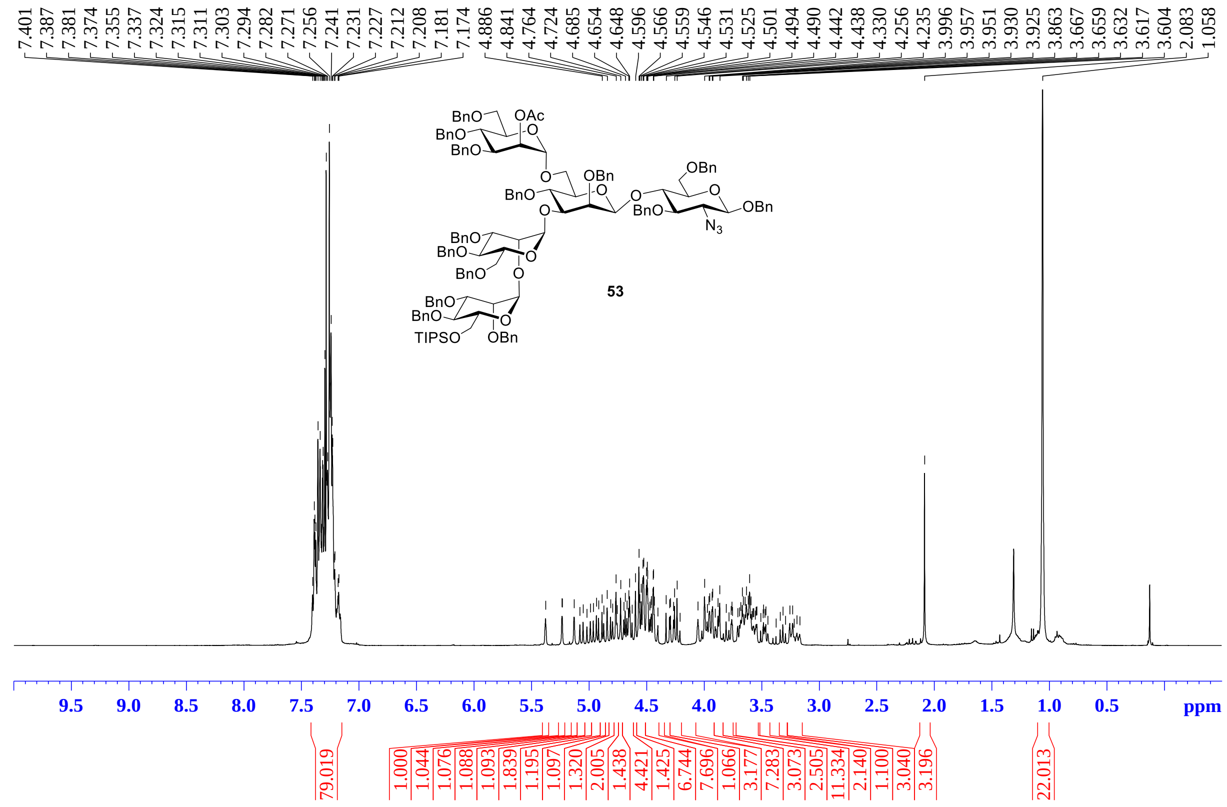

Compound 53: ^{1}H NMR ($CDCl_3$, 400 MHz)

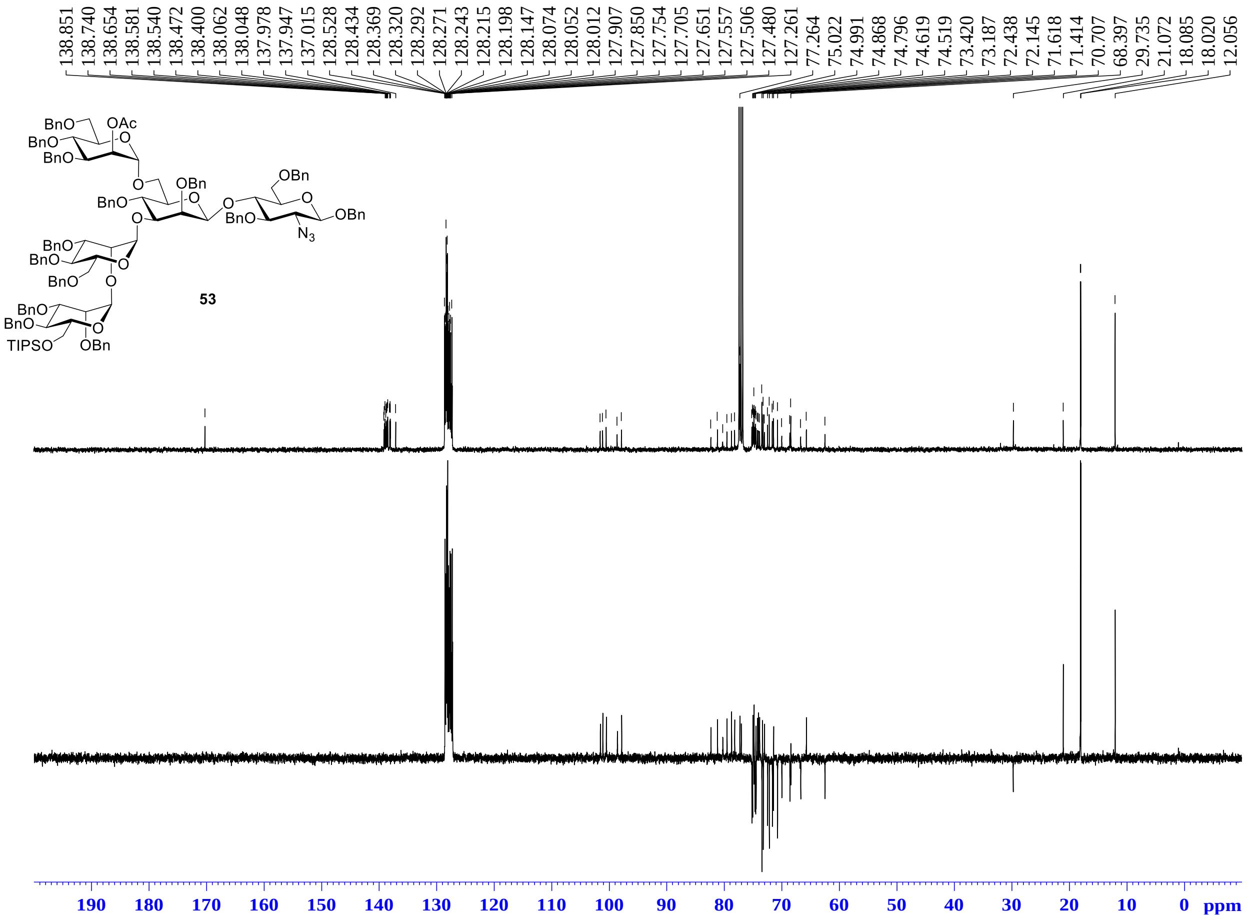

Compound 53: ^{13}C and Dept-135 NMR (CDCl₃, 100 MHz)

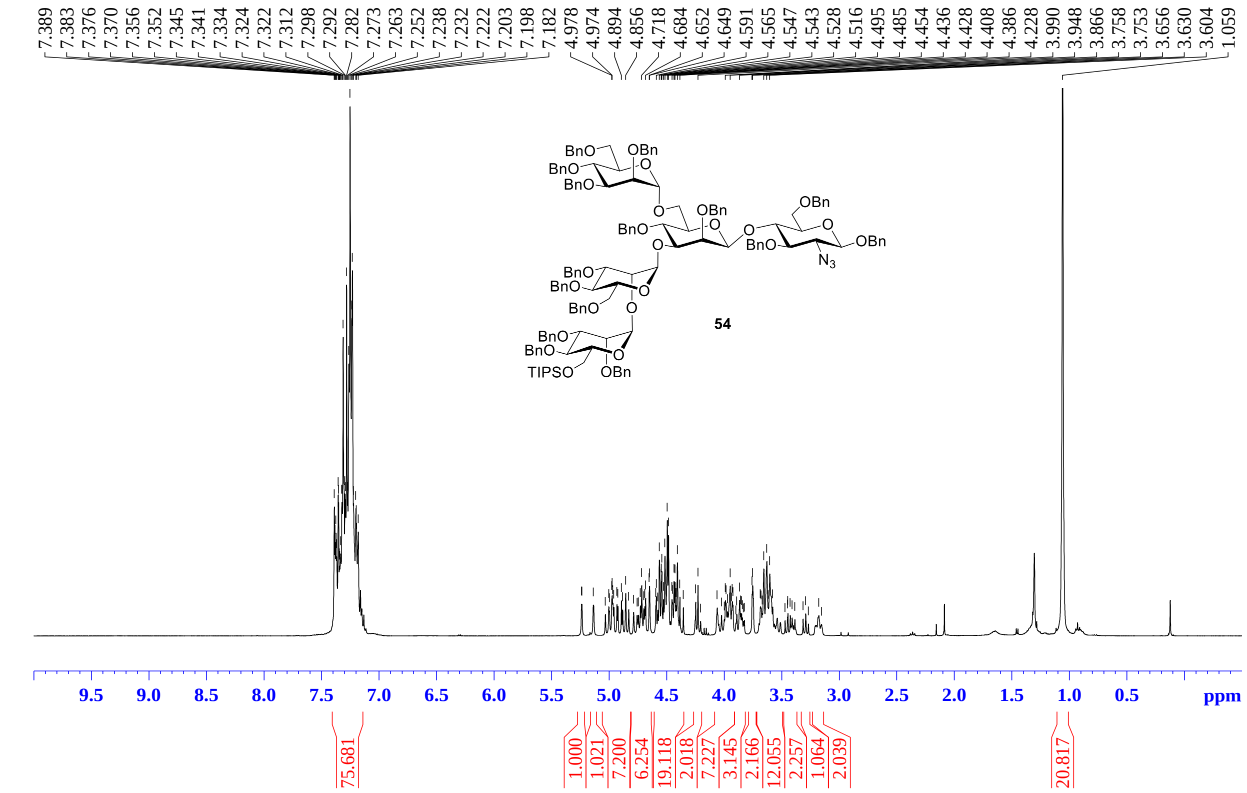

Compound 54: ^{1}H NMR (CDCl₃, 400 MHz)

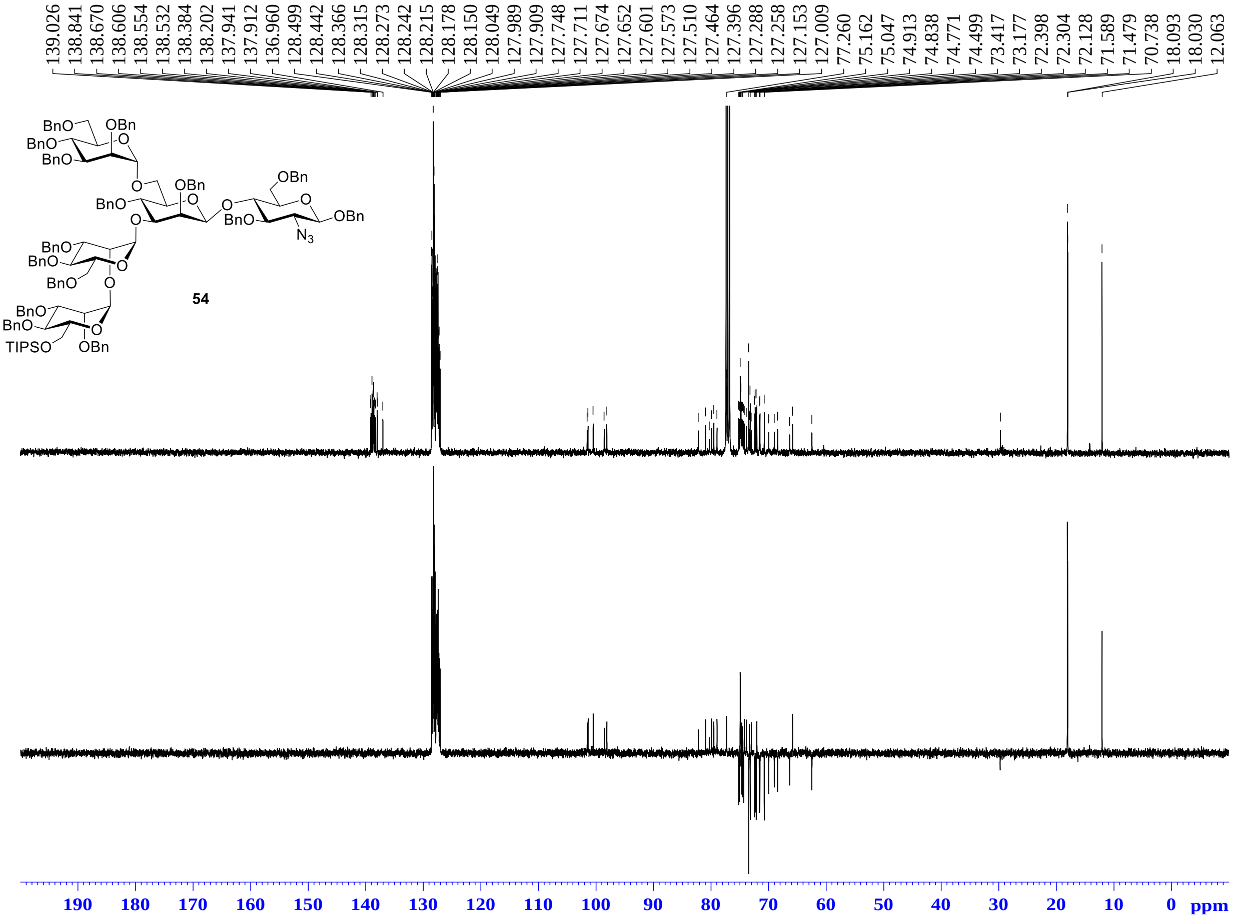

Compound 54: ^{13}C and Dept-135 NMR (CDCl₃, 100 MHz)

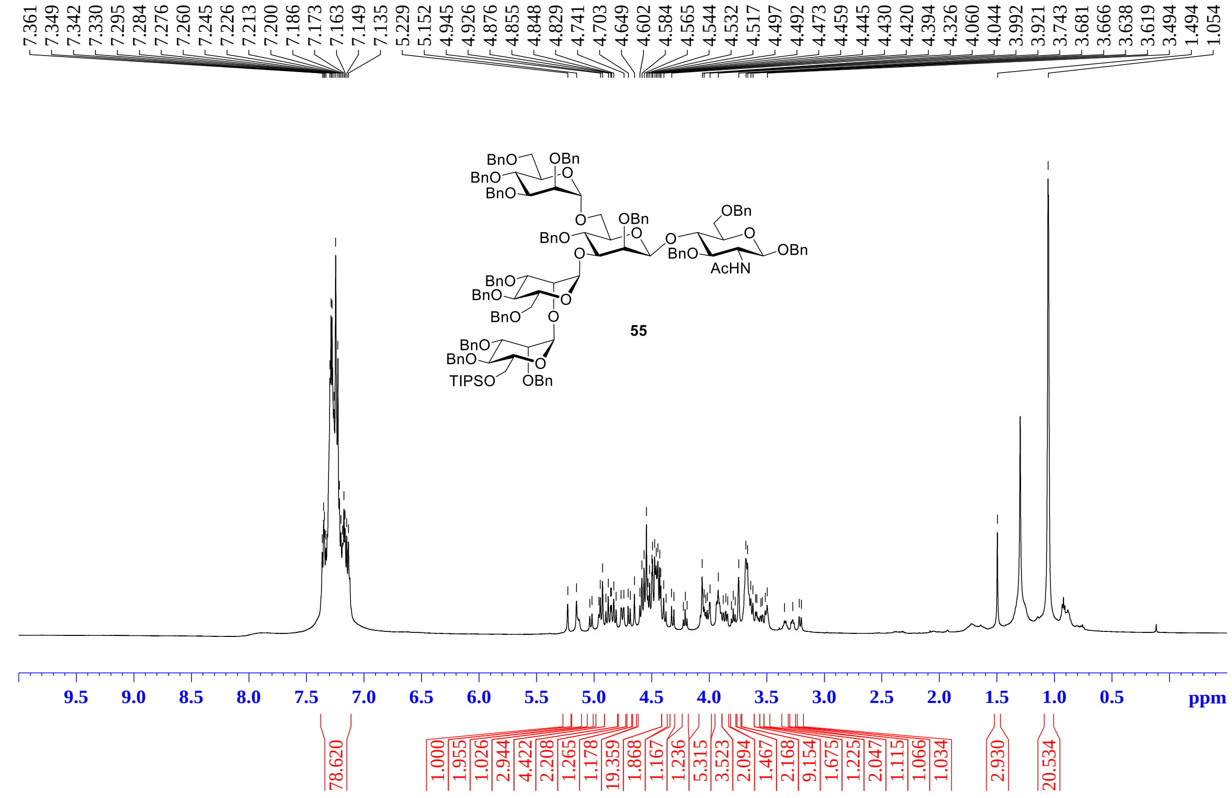

Compound 55: ^{1}H NMR (CDCl₃, 400 MHz)

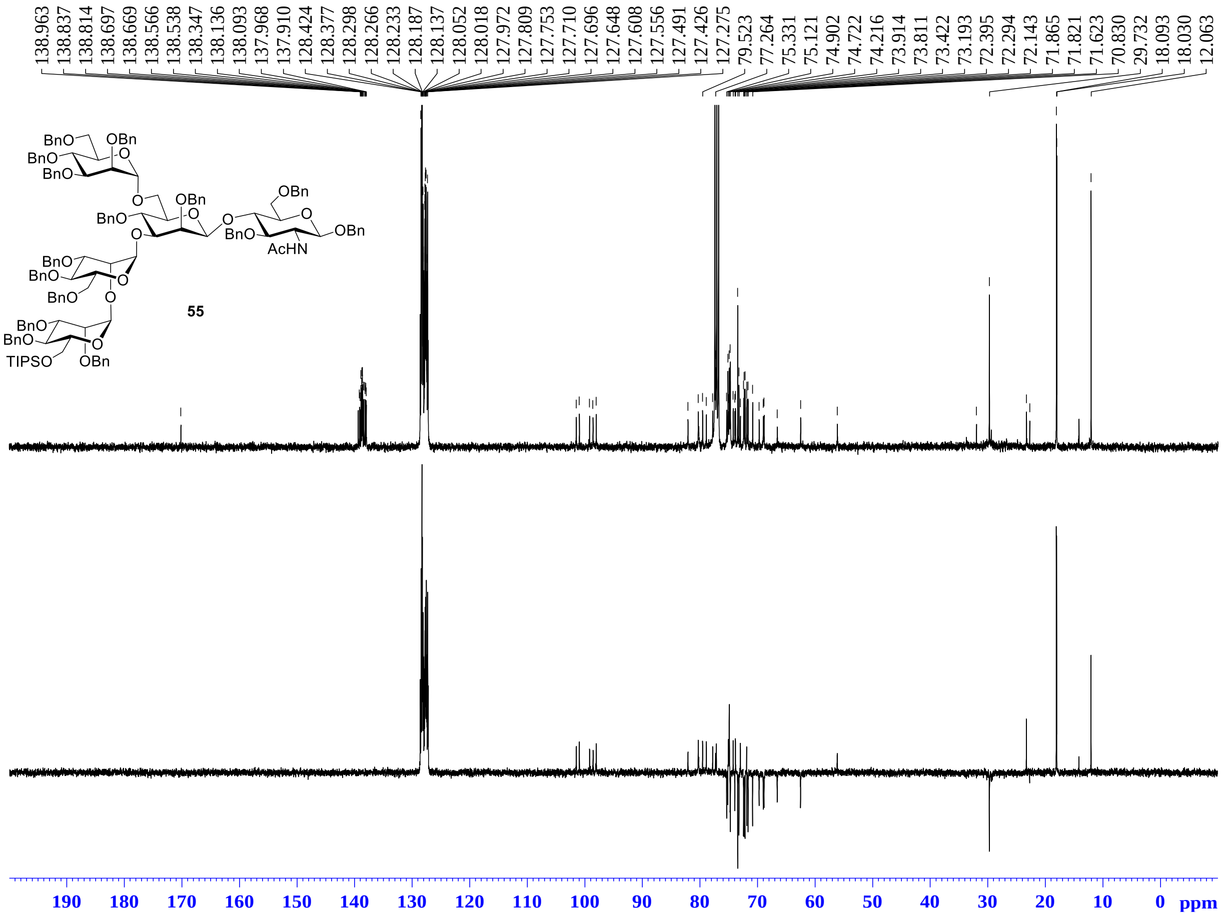

Compound 55: ^{13}C and Dept-135 NMR (CDCl$_3$, 100 MHz)

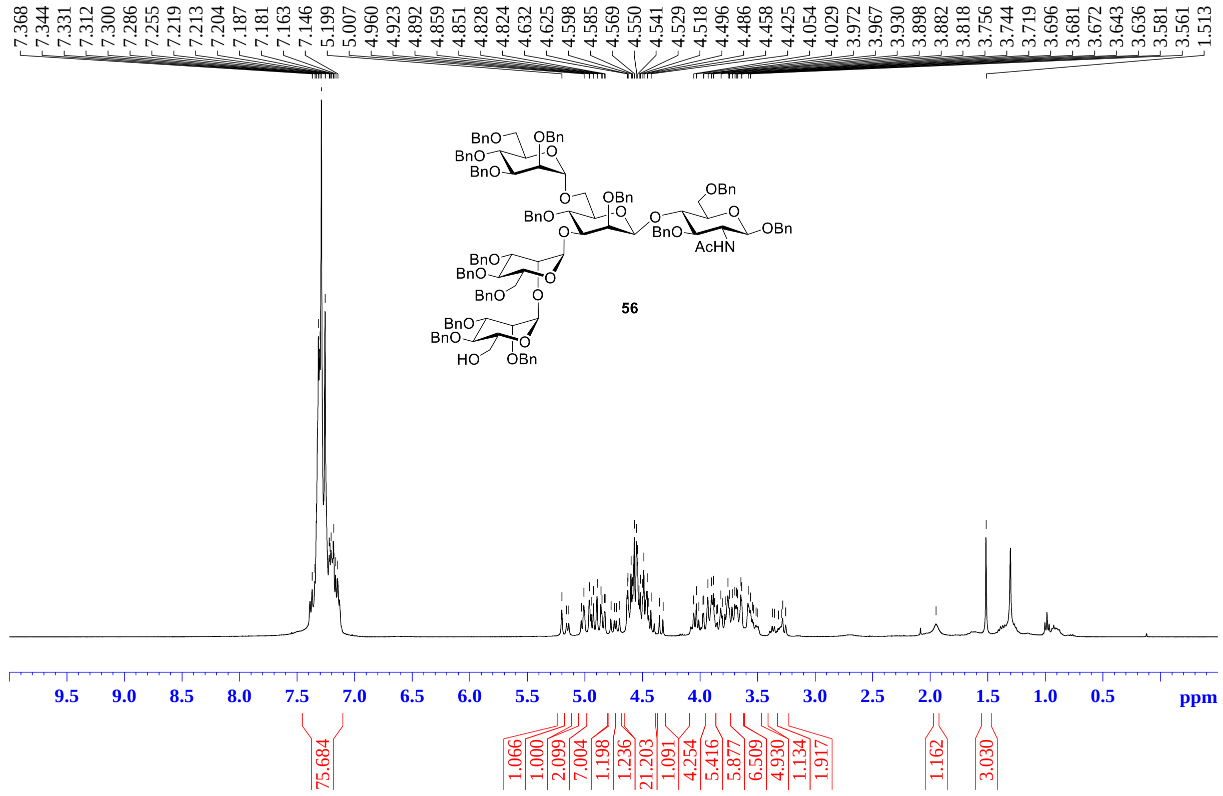

Compound 56: ^{1}H NMR (CDCl$_3$, 400 MHz)

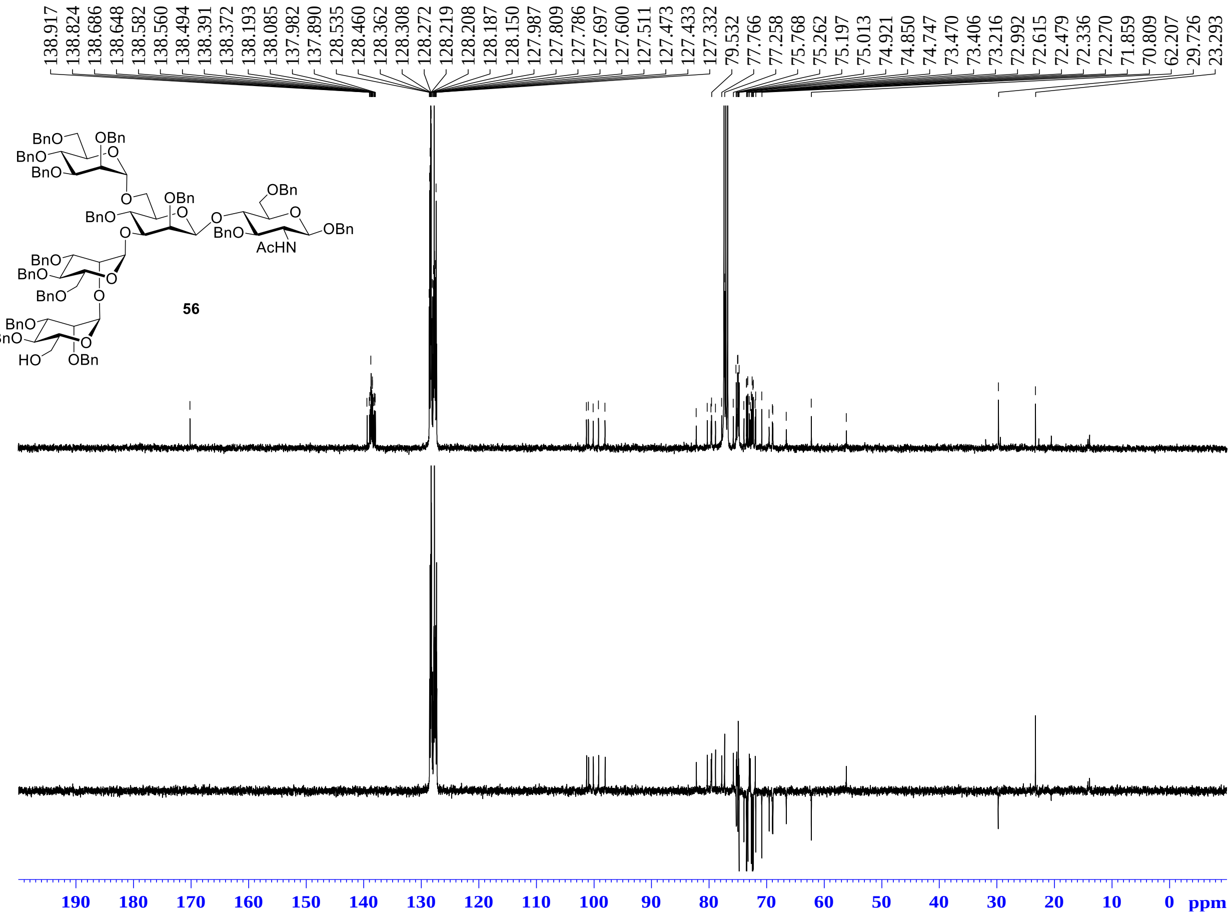

Compound 56: ^{13}C and Dept-135 NMR (CDCl$_3$, 100 MHz)

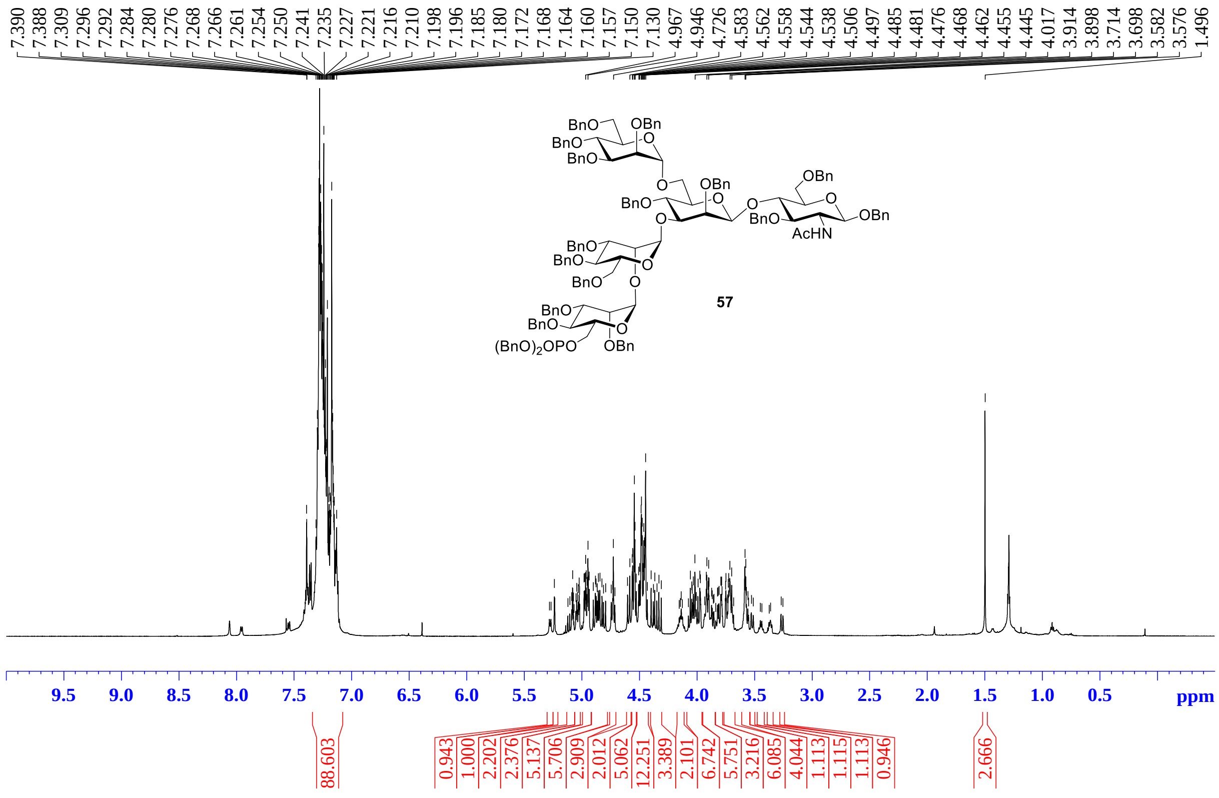

Compound 57: ^{1}H NMR (CDCl$_3$, 400 MHz)

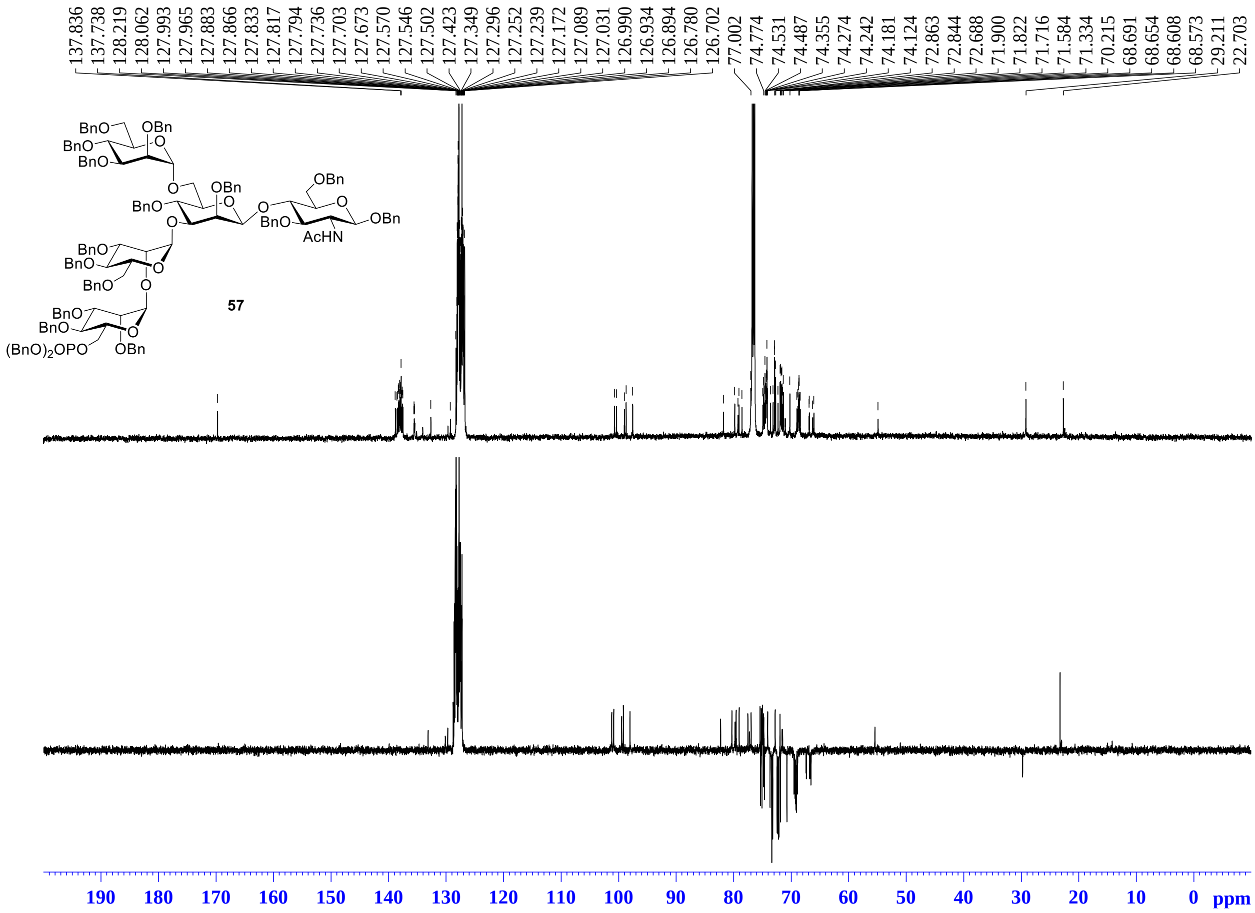

Compound 57: ^{13}C and Dept-135 NMR ($CDCl_3$, 100 MHz)

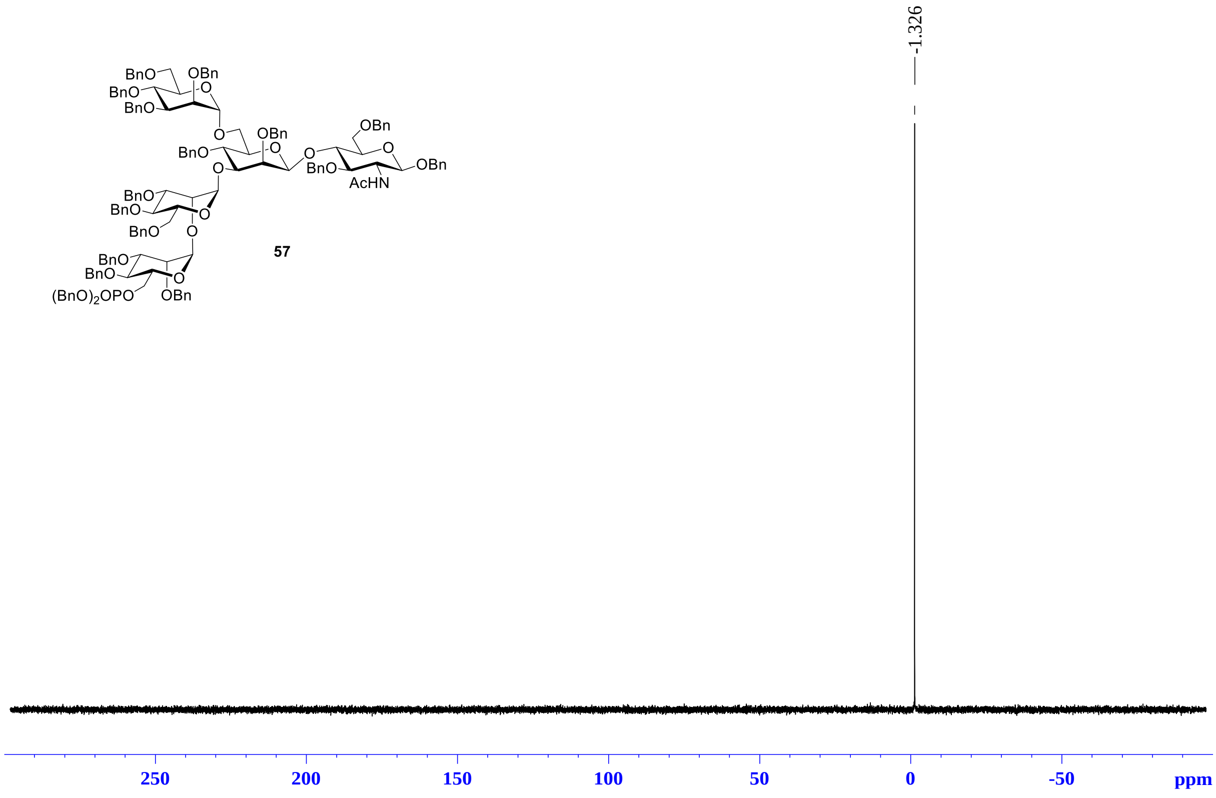

Compound 57: ^{31}P NMR ($CDCl_3$, 146 MHz)

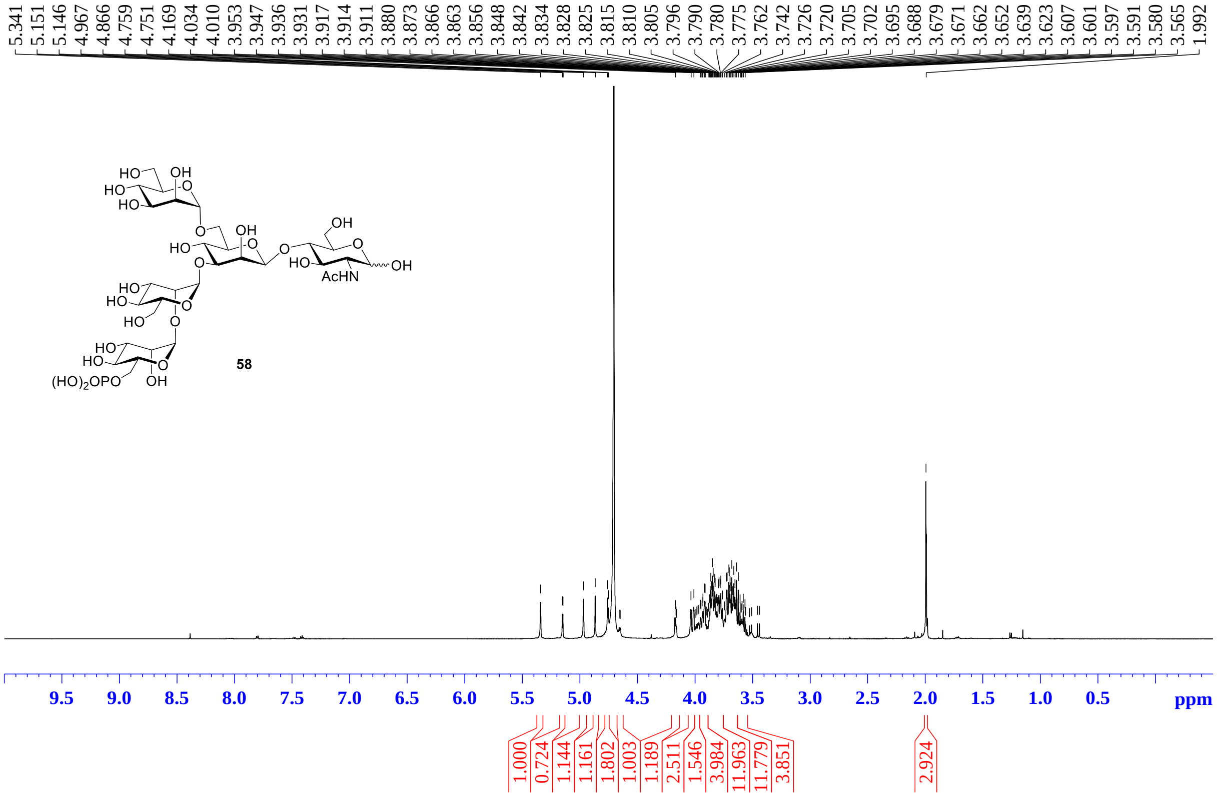

Compound 58: ^{1}H NMR (D_2O, 400 MHz)

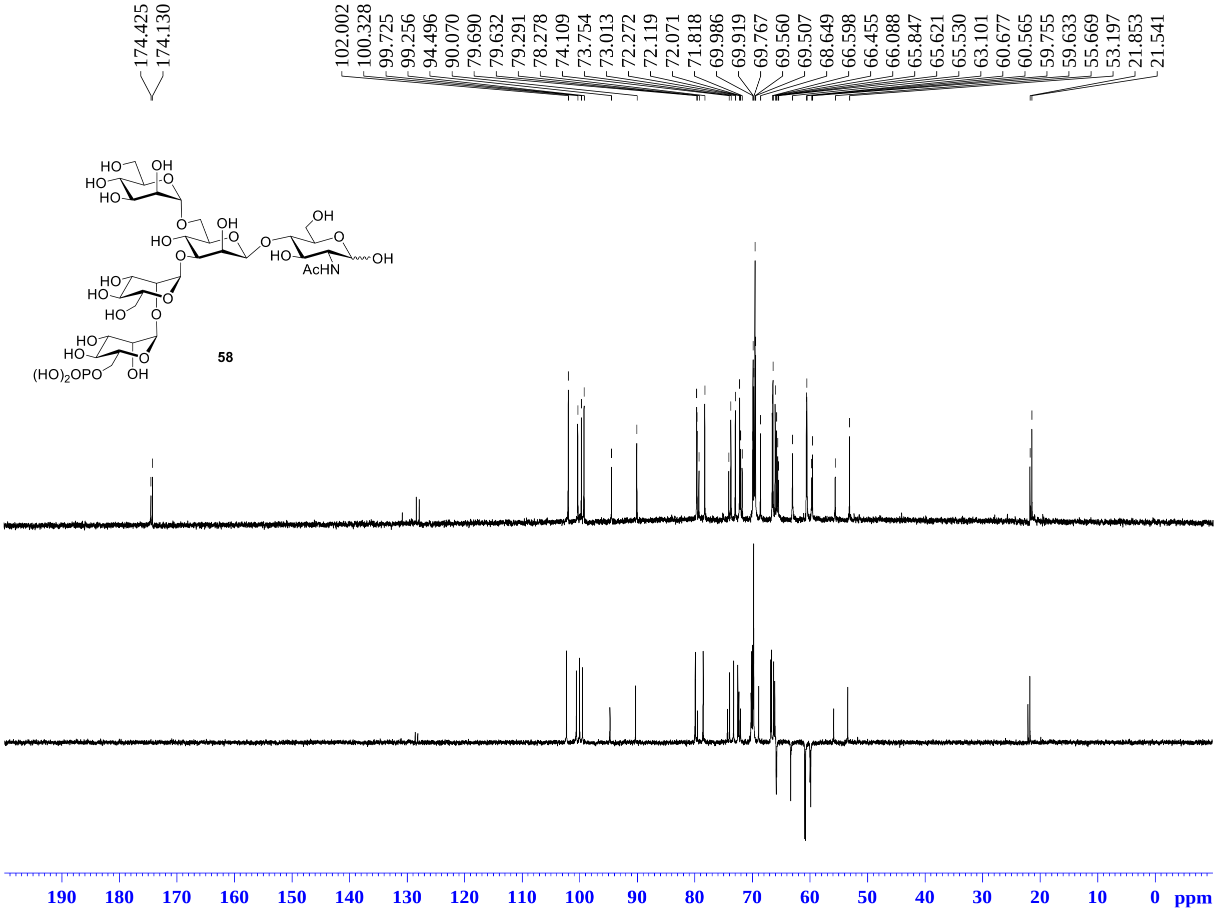

Compound 58: ^{13}C and Dept-135 NMR (D_2O, 400 MHz)

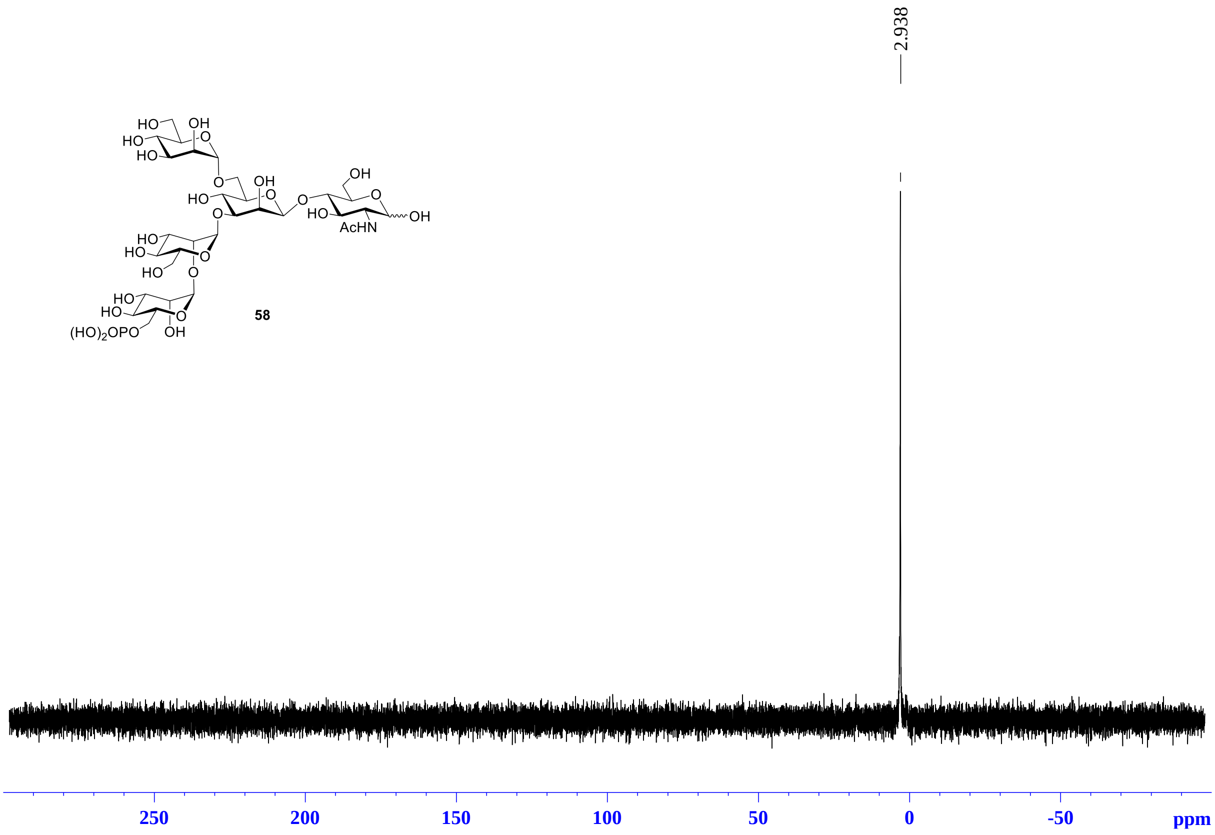

Compound 58: ^{31}P NMR (D_2O, 146 MHz)

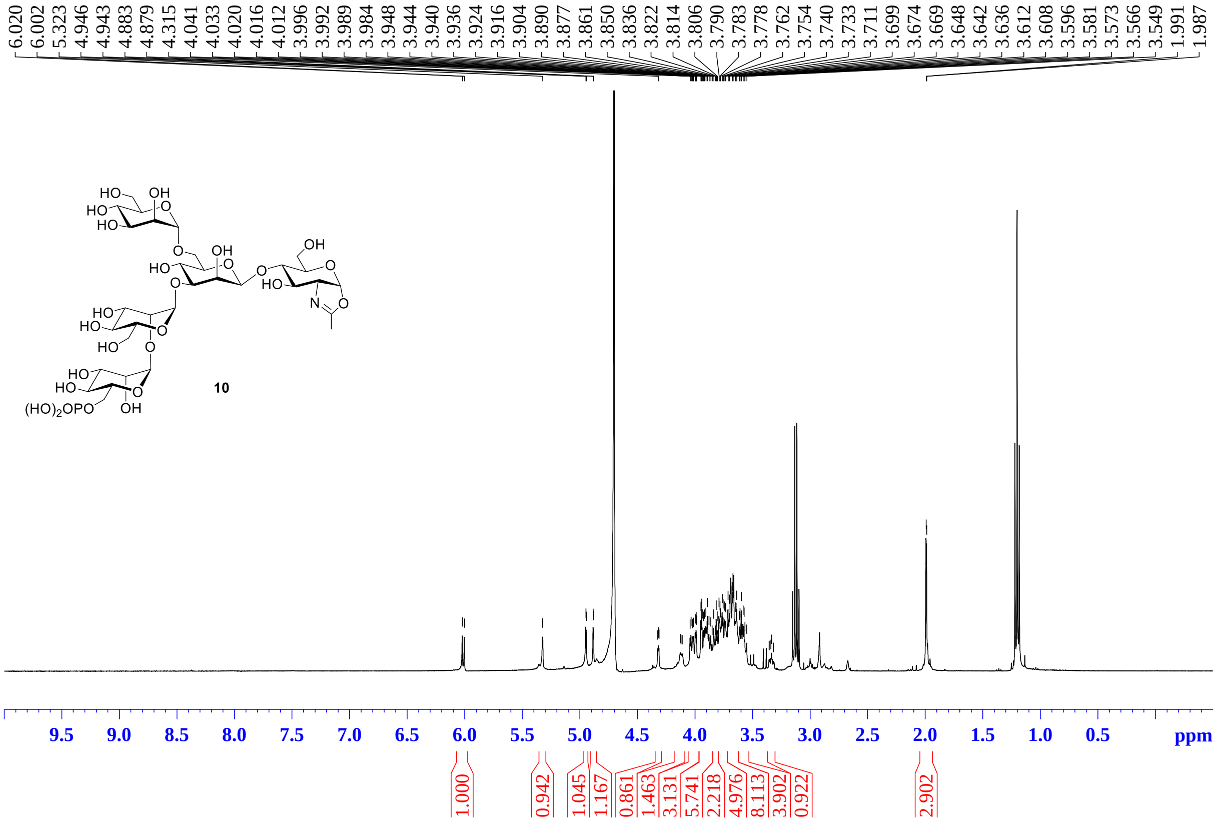

Compound 10: ^{1}H NMR (D_2O, 400 MHz)

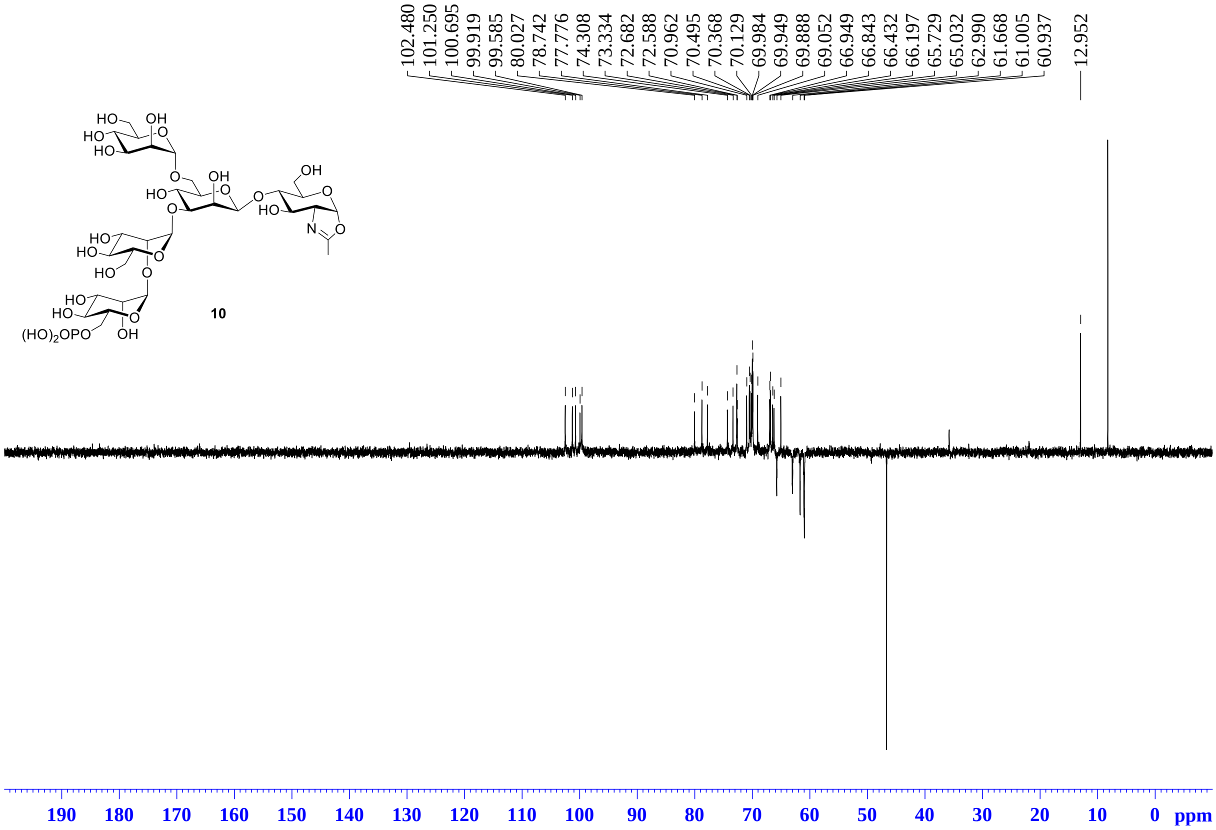

Compound 10: Dept-135 NMR (D_2O, 100 MHz)

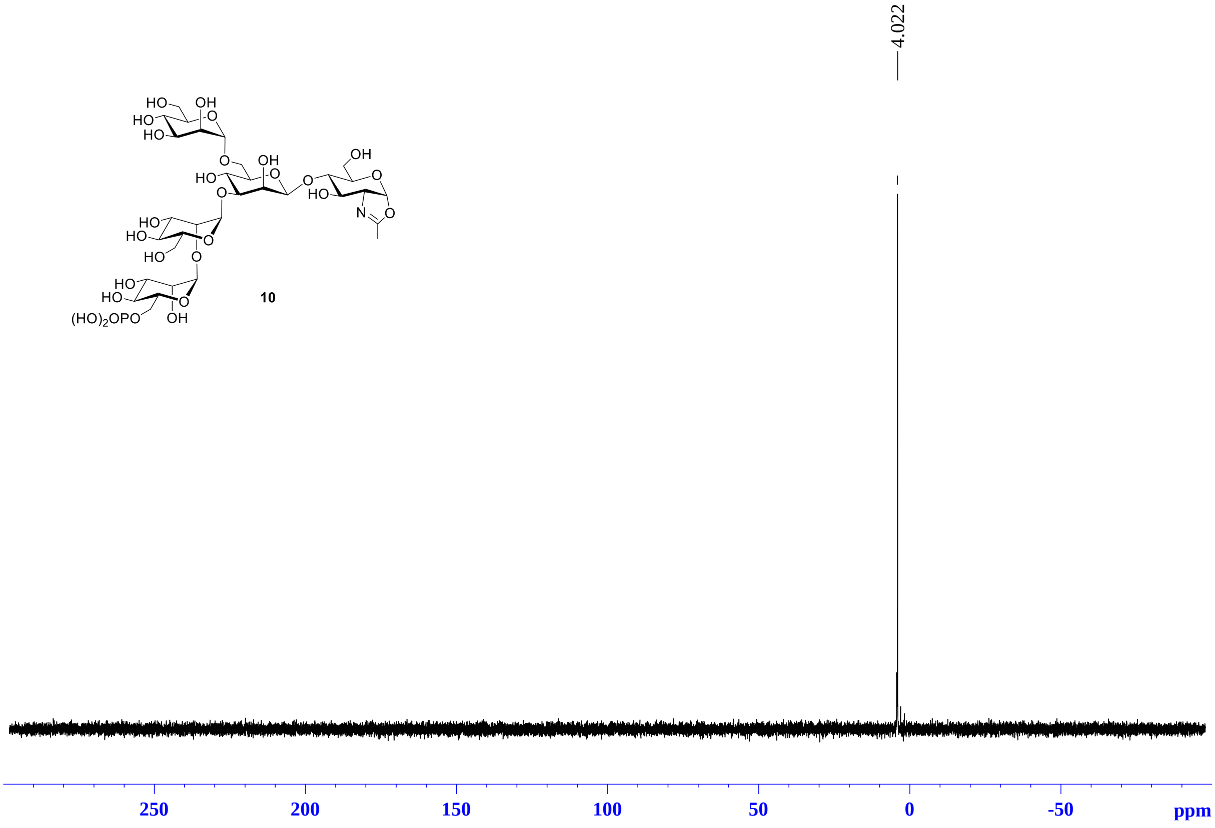

Compound 10: ^{31}P NMR (D_2O, 146 MHz)
